# Flexible organic thin-film transistors for all-in-one retinomorphic acceleration enabled by inorganic-organic heterogeneous dielectrics

**DOI:** 10.1038/s41467-026-76116-z

**Published:** 2026-07-27

**Authors:** Yilin Zhao, Dongyang Zhu, Ting Jiang, Le Wang, Ruiheng Wang, Guanran Wang, Yu Duan, Haifeng Ling, Deyang Ji, Wenping Hu

**Affiliations:** 1 Institute of Molecular Aggregation Science, Tianjin University, Tianjin, China; 2 State Key Laboratory of Flexible Electronics & Jiangsu Key Laboratory of Neuromorphic Electronics, Institute of Advanced Materials, Nanjing, University of Posts & Telecommunications, Nanjing, China; 3 State Key Laboratory of Integrated Optoelectronics, College of Electronic Science and Engineering, Jilin University, Changchun, China; 4 Key Laboratory of Organic Integrated Circuit, Ministry of Education, Tianjin University, Tianjin, China; 5 State Key Laboratory of Advanced Materials for Intelligent Sensing, Tianjin University, Tianjin, China; 6 Tianjin Key Laboratory of Molecular Optoelectronic Sciences, Department of Chemistry, School of Science, Tianjin University, Tianjin, China

**Keywords:** Electronic devices, Electronic properties and materials, Sensors

## Abstract

Organic neuromorphic visual sensors promise to overcome the limitations of conventional image sensors, but a trade-off between efficient charge transport and charge trapping has hindered the development of interfaces that combine efficient optical sensing and non-volatile memory. Here we show that an inorganic-organic heterogeneous dielectric (Al_2_O_3_/PAA), fabricated by low-temperature plasma-enhanced atomic layer deposition and spin-coating, enables flexible thin-film transistors with integrated sensing-memory-processing functions. The devices exhibit an average mobility of 22.65 cm^2 ^V^−1^ s^−1^, a 30 µs optical response to 450 nm light, charge retention exceeding ten years, write/erase endurance over 10^4^ cycles, an electrical response down to 130 ns, and stable multilevel programming at 10 µs. They emulate synaptic plasticity and achieve recognition accuracies of 99.49% (gesture), 94.78% (digit) and 92.56% (face). When integrated with a convolutional neural network accelerator, the system demonstrates real-time, interference-resistant face detection, highlighting the potential of this dielectric architecture for flexible neuromorphic vision systems.

## Abstract (web summary)

Organic neuromorphic visual sensors are often limited by the trade-off between electronic transport properties and non-volatility. Through dielectric layer optimization, Zhao et al. develop a flexible organic transistor with balanced performance that integrates sensing-memory-processing, supporting real-time face detection.

## Introduction

With aerospace technology progressively transitioning towards intelligence, the deployment of intelligent object detection systems on spaceborne platforms has garnered significant attention^1^. However, constrained by the severe scarcity of onboard computing resources, researchers are developing machine vision systems that can perceive, analyze, and predict their surroundings through visual sensing^2,3^. However, traditional artificial vision systems typically employ physically separated units for sensing, memory, and processing, resulting in structural complexity, high redundancy, and substantial energy consumption^4,5^. To overcome these limitations, neuromorphic visual sensing systems have emerged. By integrating sensing with in-memory computing, such systems can accelerate data transmission, perform autonomous image analysis and rapid edge computation, thereby significantly lowering energy consumption and operational costs^6–10^. At the core of such systems lie integrated sensing-memory-processing devices, which unify multiple functions within a single structure and have thus attracted considerable research attention^11–13^. In recent years, integrated devices based on inorganic materials and operating with electrical pulse inputs have achieved remarkable performance: nanosecond operation speeds, retention exceeding 10 years, endurance over 10^6^ cycles, and recognition accuracy above 95%^14–16^. In contrast, devices responsive to optical pulse signals—which are essential for visual sensing—lag significantly behind, typically offering only millisecond detection speeds, retention on the order of 10^4 ^s, endurance below 10^3^ cycles, and recognition accuracy around 90%^8,9,17–20^. Moreover, inorganic semiconductors and dielectrics often require high-temperature processing, entail high fabrication costs, and exhibit limited mechanical flexibility, constraining their applicability in flexible and versatile sensing scenarios^8,10,21–24^.

Organic semiconductor/dielectric materials, with their inherent flexibility, low cost, low-temperature processability, and tunable optoelectronic properties, offer a promising platform for developing flexible sensing-memory-processing devices that operate under photoelectric signal input^25–27^. While advances have been reported in this direction^28–30^, a fundamental challenge persists: the intrinsic trade-off between charge transport efficiency in the semiconductor channel and charge trapping capability in the gate dielectric layer^31–33^. For instance, optimizing the organic semiconductor for high carrier mobility (>10 cm^2 ^V^−1^ s^−1^) and fast switching typically leads to volatile memory retention (<1000 s)^34,35^. Conversely, engineering the dielectric for enhanced charge trapping—often reflected in a large memory window and long retention time (>100 h)—usually results in severely degraded field-effect mobility and, consequently, slower device operating speed^36–38^. Therefore, designing key interfacial architectures that simultaneously achieve efficient charge transport and non-volatile charge trapping represents a critical scientific hurdle toward high-performance organic neuromorphic vision sensors.

In this work, we develop an inorganic-organic heterogeneous dielectric layer by utilizing low-temperature plasma-enhanced atomic layer deposition (LT-PEALD) to grow aluminum oxide (Al_2_O_3_) onto the spin-coated poly (amic acid) (PAA) film. By optimizing the dielectric layer architecture and thickness ratios, we demonstrated the critical role of the polar polymer PAA in bottom-up modulation of interfacial charge trapping efficiency and charge transport kinetics. The fabricated flexible thin-film transistor with a 30 nm Al_2_O_3_/180 nm PAA interface exhibited a carrier mobility exceeding 20 cm^2 ^V^−1^ s^−1^. For sensing performance, the device exhibits detection sensitivity for monochromatic illumination at 450 nm and an optical response time (10–90% rise time) of 30 μs. In memory applications, it demonstrates an electrical pulse response speed down to 130 ns, a stable multilevel programming speed of 10 μs, a memory window of 22.2 V, long-term retention stability over 10 years, and reliable cycling endurance exceeding 10^4^ cycles. Notably, in neuromorphic computing applications, the device achieves 99.49% gesture recognition, 94.78% handwritten digit recognition accuracy, and 92.56% facial recognition accuracy, which is a leading position among flexible organic thin-film field effect transistors. The successful integration of our synaptic devices with a convolutional neural network hardware accelerator enables real-time, robust face detection with strong interference resistance even in complex environments.

## Results

### Characterization of heterogeneous dielectric layer

**Fig. 1:**
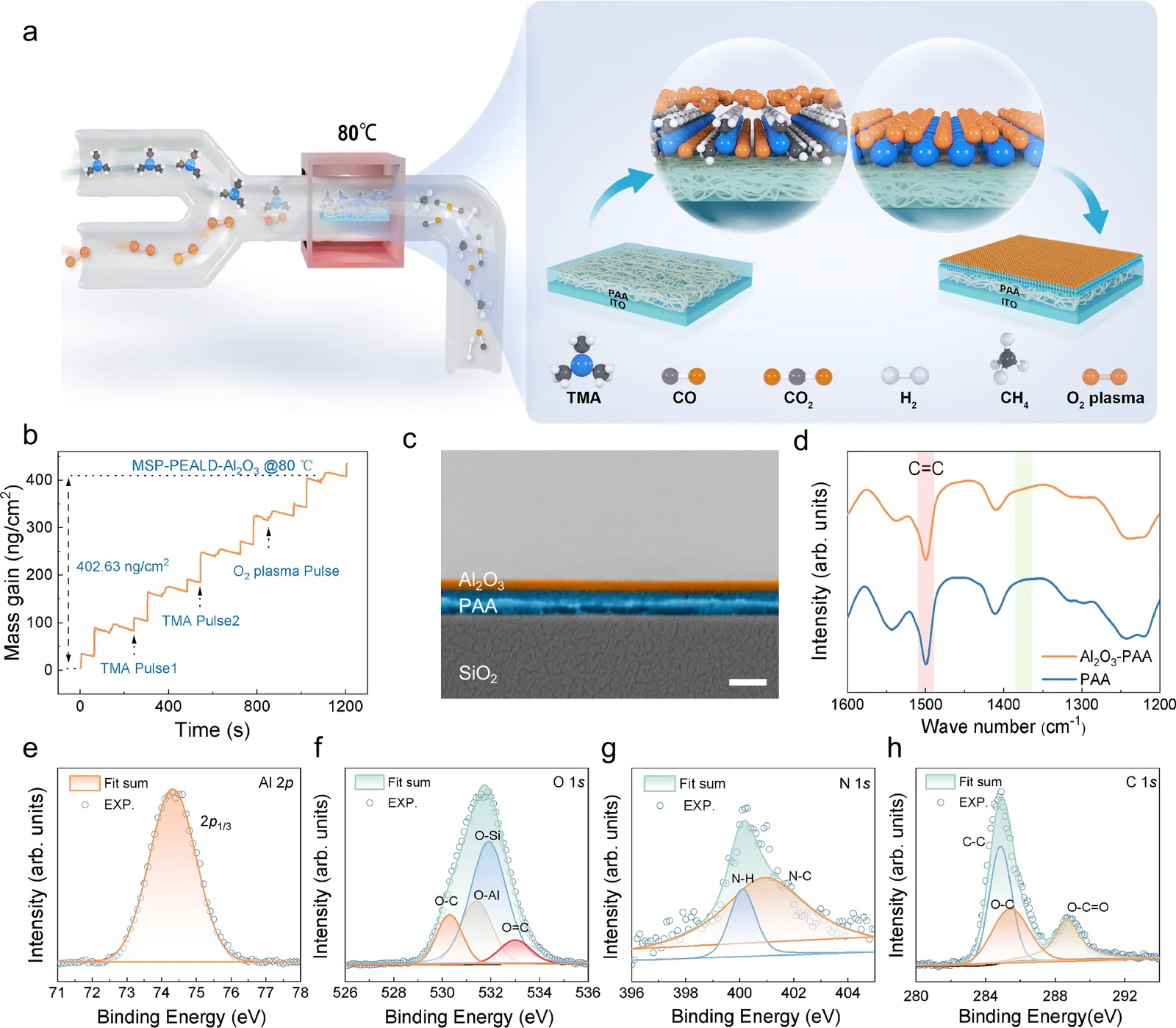
Construction and characterization of heterogeneous dielectric layers. **a** Schematic diagram of the process of growing Al_2_O_3_ on PAA film using PEALD at 80 °C. **b** Mass gain of Al_2_O_3_ during PEALD. **c** Cross-sectional scanning electron microscope images of inorganic-organic heterogeneous dielectric layers. Scale bar: 200 nm. **d** IR spectra of PAA and Al_2_O_3_/PAA films. Use x-ray photoelectron spectroscopy (XPS) to analyze the Al_2_O_3_/PAA film for Al 2*p* (**e**), O 1*s* (**f**), N 1*s* (**g**), C 1*s* (**h**), orbitals.

Conventional integration of inorganic-organic dielectric layers typically requires depositing the organic component atop an inorganic substrate, due to the high-temperature processing constraints of inorganic dielectric materials^30,39,40^. In this work, we invert this sequence by depositing Al_2_O_3_ via LT-PEALD directly onto spin-coated PAA film, forming an inorganic-organic heterogeneous dielectric stack. This architecture is designed to concurrently address the inherent trade-off between efficient charge transport and non-volatile charge trapping: the LT-PEALD grown Al_2_O_3_ layer provides a high-quality dielectric with abundant and stable charge-trapping sites^41–43^, while the underlying PAA layer, with its intrinsic self patterned nanogrooves, facilitates efficient in-plane charge transport^44^. As schematically shown in Fig. 1a, the deposition uses trimethylaluminum (TMA) and O_2_ plasma at a substrate temperature of 80 °C, ensuring complete precursor reaction while preserving the integrity of the organic underlayer^38,45–48^. The multi-step short-pulse process promotes saturable adsorption and minimizes physical adsorption, yielding a conformal Al_2_O_3_ film at low temperature^49^. The growth was monitored in situ by quartz crystal microbalance (QCM), which confirmed a stable and reproducible deposition process, indicative of high-quality Al_2_O_3_ film formation (Fig. 1b).

The interfacial morphology of the Al_2_O_3_/PAA heterostructure was characterized by high-resolution scanning electron microscopy (HR-SEM) (Fig. 1c), which shows excellent adhesion between the layers. The smooth surface of the underlying PAA film (Supplementary Fig. 1a) results in a reduced roughness for the Al_2_O_3_/PAA stack compared to bare Al_2_O_3_ (Supplementary Fig. 1b, c). FTIR spectroscopy (Fig. 1d) confirms that the PAA layer does not undergo imidization during the LT-PEALD process at 80 °C, as evidenced by the absence of the characteristic C-N-C absorption band near 1375 cm^−1^ and the preservation of the C=C stretching vibration at ~1500 cm^−1^ ^44^. XPS analysis further elucidates the chemical states at the interface (Fig. 1e–h). The Al 2*p* peak at 74.3 eV confirms the formation of Al_2_O_3_, while the O 1*s* spectrum reveals distinct contributions from O-Al (531.4 eV), C-O (531.5 eV), and C=O (533.0 eV) bonds. The N 1*s* spectrum shows doublets corresponding to N-H (400.1 eV) and N-C (400.9 eV), and the C 1*s* spectrum displays peaks for C-C (284.8 eV), C-O (285.5 eV), and O-C=O (288.6 eV). Critically, no residual peaks indicating chemical interaction between PAA and Al_2_O_3_ are observed, confirming the absence of interfacial reaction. The dielectric properties of Al_2_O_3_/PAA layers with varying thicknesses are summarized in Supplementary Fig. 2. Furthermore, the heterogeneous dielectric layer comprising 30-nm-thick Al_2_O_3_ on 180 nm-thick PAA exhibits a breakdown voltage of 58.8 V and a leakage current density of 13.92 mA cm^−2^, outperforming both single-layer PAA and Al_2_O_3_ films of equivalent thickness. (Supplementary Fig. 3).

### Flexible organic thin-film transistor array fabrication

**Fig. 2:**
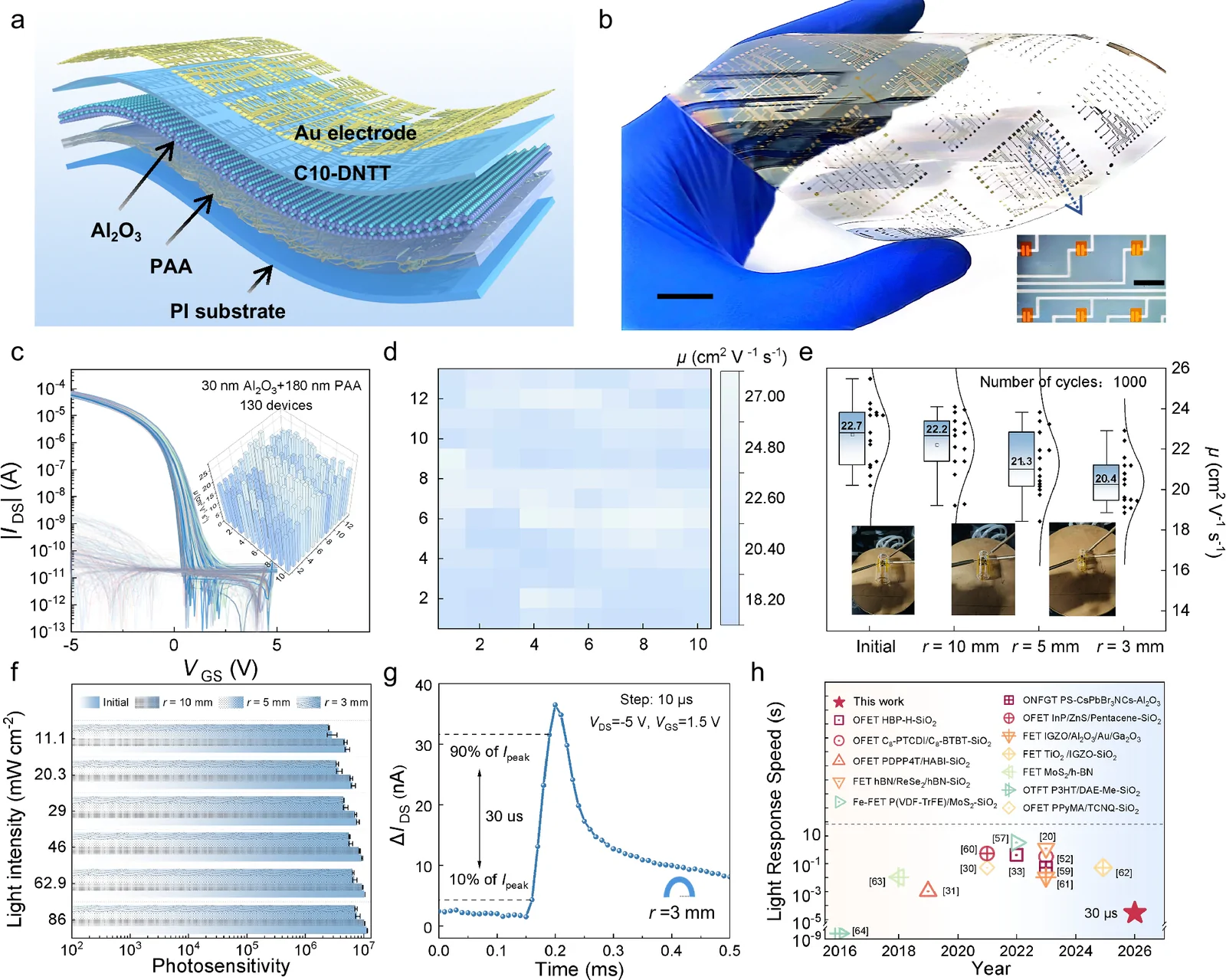
Flexible OTFT device preparation, characterization and optical sensing capability. **a** Schematic diagram of a flexible organic thin film transistor. **b** Array and localized demonstration of large-area flexible thin-film transistors fabricated on ITO-coated polyimide substrates with Al_2_O_3_/PAA dielectric and C10-DNTT semiconductor. The main panel (scale bar: 1 cm) shows the entire device array across the flexible substrate, while the inset (scale bar: 1 mm) provides a magnified view of the source and drain contact electrodes, clearly revealing their patterning and interconnection details. **c** Comparison of transfer characteristic curves, leakage current curves, and steric distribution of mobility for 130 devices on flexible substrates. **d** Mobility distribution map of 130 devices on a flexible substrate (*f* = 0.1 Hz). **e** Comparison of the mobility of the devices on flexible substrate bending for 1000 cycles at different bending radius (*f* = 0.1 Hz). Error bars represent the standard deviation (SD) of the mobility values obtained from 15 devices. **f** Variation of device *P*-value with light intensity at different bending radius on flexible substrates (450 nm). **g** Response current of devices to 50 μs laser pulse on flexible substrate. **h** Photoresponse velocity statistics of organic phototransistors.

To assess the potential of Al_2_O_3_/PAA films for flexible electronics (Fig. 2a), we first fabricated organic thin-film transistors (OTFTs) with nine distinct dielectric configurations on rigid ITO/glass substrates to systematically investigate the effects of layer thickness and deposition sequence (Supplementary Figs. 4–6). All heterojunction dielectric OTFTs exhibited uniform p-type transport with a stable on/off current ratio of ~10^7^ and negligible leakage (Supplementary Fig. 4). Comparative analysis revealed that the configuration with Al_2_O_3_ deposited on PAA yielded lower threshold voltages and higher on-state currents than alternative sequences (Supplementary Fig. 5a). An optimal Al_2_O_3_ thickness of 30 nm was identified from the transfer characteristics (Supplementary Fig. 5b). Statistical analysis of carrier mobility across the configurations confirmed that increasing PAA thickness enhances charge transport under a fixed Al_2_O_3_ thickness (Supplementary Fig. 6). Among 120 devices, the 30 nm Al_2_O_3_/180 nm PAA configuration achieved an average mobility of 20.21 cm^2 ^V^−1^ s^−1^ at *V*_DS_ = −5 V on ITO/glass. The electrical properties of different dielectric layers are compared as shown in Supplementary Table 1. To exclude contact-resistance artefacts, we performed transmission line method (TLM) measurements on devices with the optimized dielectric stack (Supplementary Figs. 7–9). The extracted channel-width-normalized contact resistance is R_c_W = 420 Ω·cm, which is within the typical range (10–10^3^ Ω·cm) reported for high-mobility transistors^23,50,51^. Using this optimized dielectric stack with a C10-DNTT semiconductor, we fabricated large-area device arrays on flexible ITO/polyimide (PI) substrates (Fig. 2b). Characterization of 130 devices (Fig. 2c) yielded an average mobility of 22.65 cm^2 ^V^−1^ s^−1^ and a maximum mobility of 29.11 cm^2 ^V^−1^ s^−1^ (Fig. 2d, Supplementary Fig. 10), representing advanced performance in previously reported organic field-effect transistors (Supplementary Fig. 11)^29,30,33,52–56^.

To assess the mechanical robustness of flexible thin-film transistors under continuous mechanical deformation, as depicted in Fig. 2e, 1000 bending cycles were conducted at bending radius of *r* = 10 mm, *r* = 5 mm, and *r* = 3 mm. The charge carrier mobility of 15 devices was measured and analyzed. Statistically, the devices retained 90% of their initial mobility even at the smallest bending radius (*r* = 3 mm) after 1000 bending cycles. This establishes a reliable foundation for the development of bend-stable, high-performance “sensing-memory-processing” integrated device arrays for practical applications. The optoelectronic response of the device (30 nm Al_2_O_3_/180 nm PAA) to 450 nm monochromatic illumination, demonstrating a pronounced positive current response across varying light intensities from 0.22 mW cm^−2^ to 86 mW cm^−2^, as depicted in Supplementary Fig. 12a. The primary photoresponsive parameters of the device, derived from the photoresponse transfer curves, include a photosensitivity (*P*) value exceeding 10^7^, a peak responsivity (*R*) value of 2.01 × 10^3 ^A W^−1^, and a maximum specific detectivity (D*) value approaching 10^14^ Jones, as illustrated in Supplementary Fig. 12b–e. This value is obtained from the directly measured noise current after noise correction (Supplementary Fig. 13). To further validate the multi-wavelength capability, extended the spectral response to 290 nm, 365 nm and 515 nm (Supplementary Figs. 14 and 15). The noise-corrected D* values at these wavelengths reach up to 4.4 × 10^14^ Jones (365 nm), indicating that the device can operate across a multi-wavelength spectral range, with a similar level of performance at 450 nm. After enduring 1000 bending cycles at a 3 mm bending radius, the device maintained a *P* value near 10^7^ with increasing light intensity, indicating robust photoelectric conversion efficiency and excellent monochromatic light detection at 450 nm (Fig. 2f, Supplementary Fig. 16). When subjected to laser pulses of 2 ms, 1 ms and 100 μs (Supplementary Fig. 17), the device exhibits consistent photoresponse down to 50 μs pulses (Fig. 2g). The optical response time, defined by the standard 10–90% rise time of the transient photocurrent, is approximately 30 μs. This experimental device achieves the highest response speed among comparable organic neuromorphic transistors (Fig. 2h^20,30–33,52,57–65^). When the human eye encounters intense light, the pupil constricts rapidly as a protective mechanism. Here, we exploit the ultrafast photoresponse of our device to control pupil dynamics. Optical signals are captured and transduced by a device array positioned behind the pupil (Supplementary Fig. 18a, b), then relayed via an external circuit (Supplementary Fig. 18c, d) to actuate pupil closure. Under dark conditions, the pupil maintains an open configuration (Supplementary Fig. 18e). Upon exposure to a 50 μs light pulse, the pupil executes rapid constriction (Supplementary Fig. 18f and Supplementary Movie 1), demonstrating biomimetic reflexive behavior with microsecond temporal resolution.

### Electrical characterization and storage mechanism

**Fig. 3:**
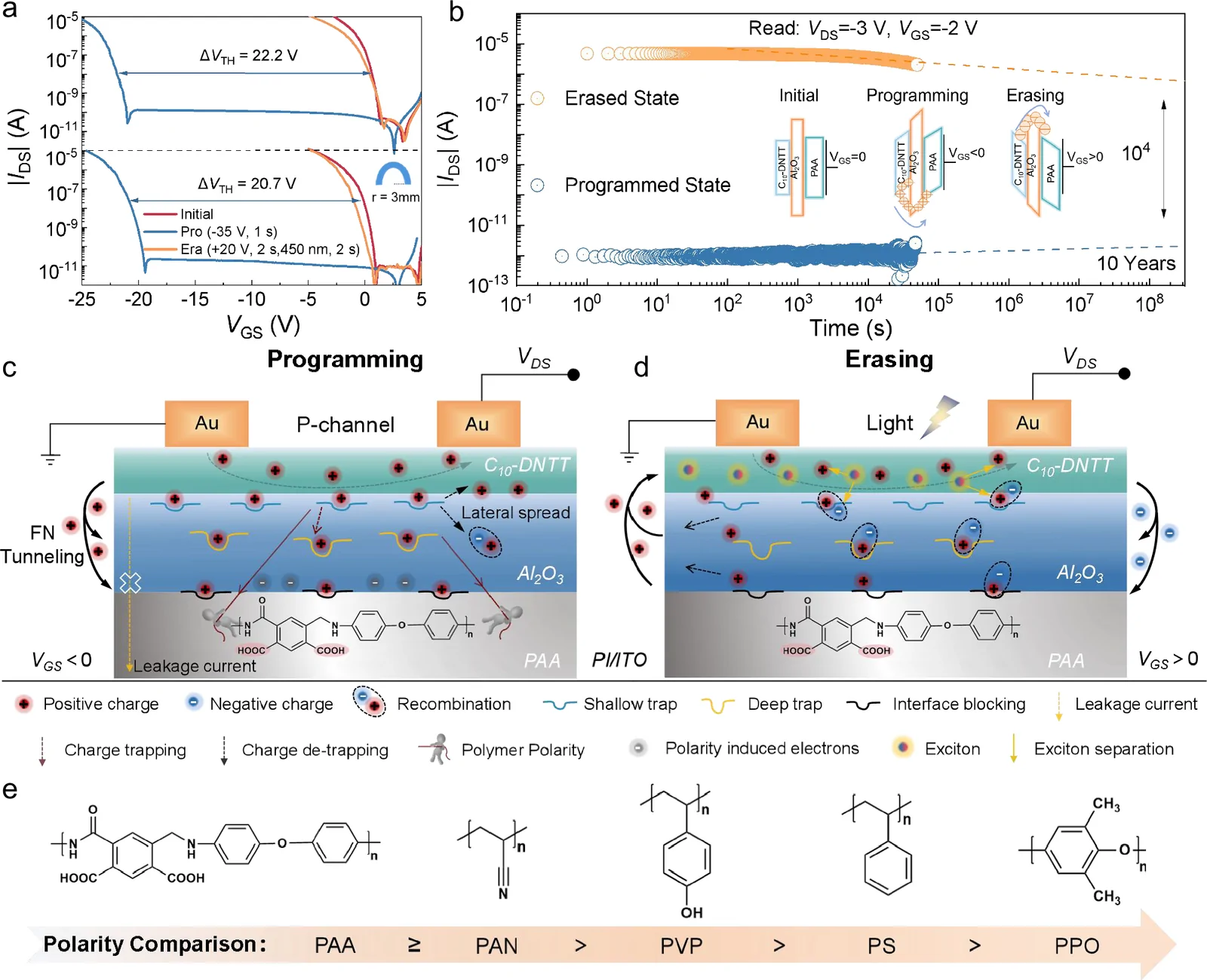
Storage capacity and mechanism of Al_2_O_3_/PAA heterogeneous dielectric layers. **a** Transfer curves of a flexible transistor based on a 30 nm Al_2_O_3_/180 nm PAA film on a flexible substrate, unbent and with a bending radius of 3 mm, after programming (*V*_GS_ = −35 V, 1 s) and erasing (450 nm light, 2 s) operations. **b** Device programming state and erase state currents for linear extrapolation. (Currents are read at *V*_DS_ = −3 V and *V*_GS_ = −2 V) and operating schematic of Al_2_O_3_/PAA based memory devices. Schematic illustrations of the **c** charge storage mechanism and **d** erasure mechanism for the Al_2_O_3_/PAA heterostructure dielectric. **e** Polarity comparison among polymers with varying dielectric strengths.

The memory operation of the organic neuromorphic transistor is driven by combined electrical and optical stimuli. As shown in Fig. 3a, programming with a −35 V gate pulse shifts the transfer curve negatively, while erasure under +20 V with 450 nm illumination restores it, yielding a memory window (Δ*V*_TH_) of 22.2 V. This window remains stable (20.7 V) under bending (*r* = 3 mm), demonstrating mechanical robustness. The charge-trapping density (Δ*n*) of the device can be calculated according to the following equation^64^, where *e* is the element charge and *C*_i_ (22.5 nF cm^−2^) is the capacitance per unit area of the gate insulating layer. The charge-trapping density of the device is calculated to be 3.12 × 10^12^ cm^−2^.

1 $$\Delta n=\frac{\Delta {V}_{{TH}}{C}_{i}}{e}$$

**Fig. 4:**
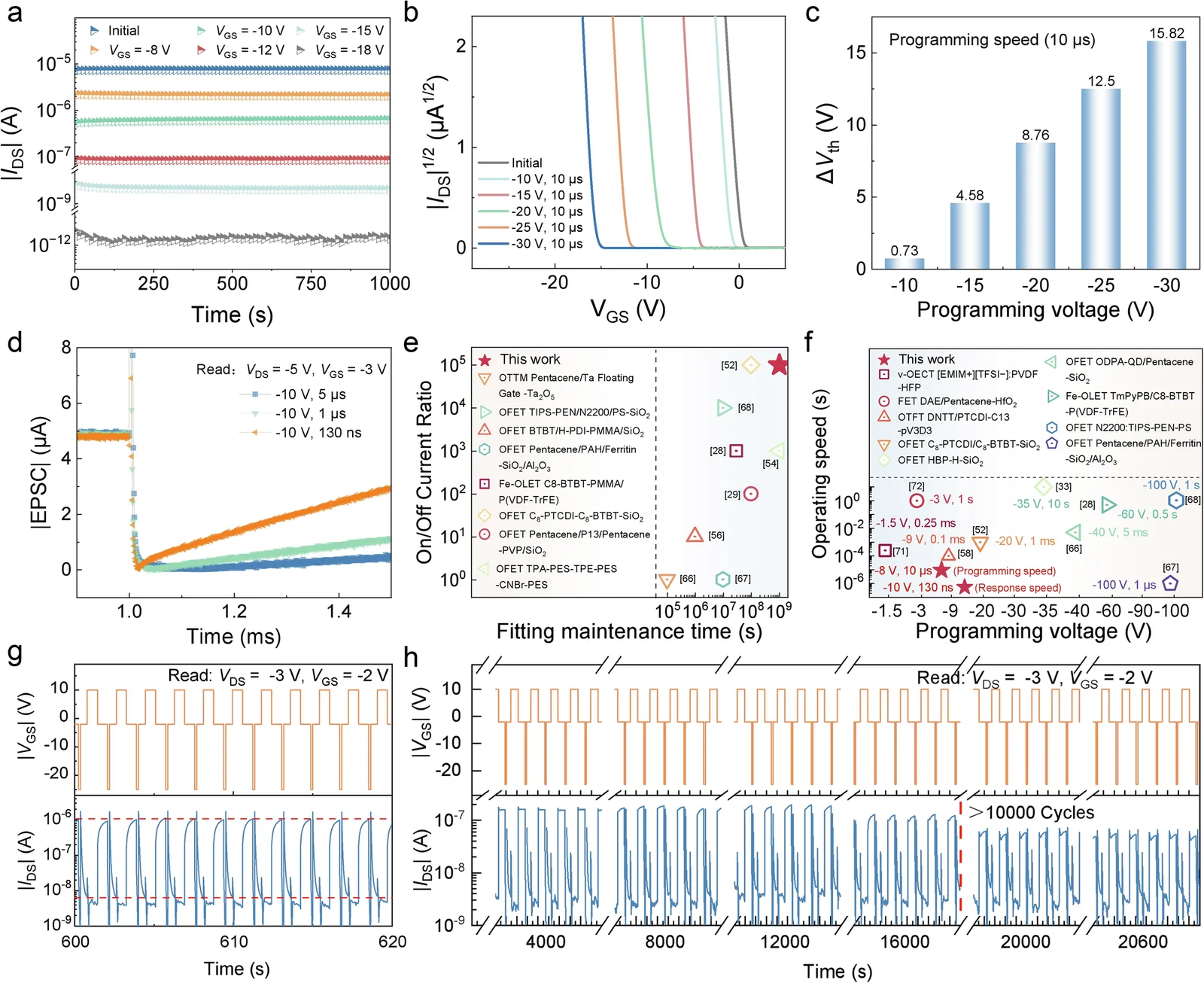
Photoelectric operation characteristics of flexible OTFTs. **a** Retention characteristics of the device under different programming voltages. The read current is monitored as a function of time after applying 10 μs programming pulses at *V*_GS_ = −8 V, −10 V, −12 V, −15 V and −18 V (read at *V*_DS_ = −3 V, *V*_GS_ = −2 V). **b** Fast storage transfer curves of the device at different voltages (−10 V, −15 V, −20 V, −25 V, and −30 V for 10 μs). **c** Storage window at 10 μs for different programming voltages. **d** The OTFT device demonstrates electrical inhibition behavior under a −10 V gate programming voltage across varying pulse widths (5 μs, 1 μs and 130 ns). **e** Comparison with the stabilization maintenance time of organic field effect transistors reported in recent years. **f** Statistical comparison of the electrical response speed of our device with previously reported organic memory transistors. Our device achieves an electrical pulse response speed of 130 ns (at –10 V) and a stable multilevel programming speed of 10 μs (at –8 V). **g** Initial programming-reading-erasing-reading cycles of the OTFT device on flexible substrate. (Programming: *V*_DS_ = 0 V, *V*_GS_ = −25 V, *t* = 0.1 s; Reading: *V*_DS_ = − 3 V, *V*_GS_ = −2 V, *t* = 0.6 s; Erasing: *V*_DS_ = 0 V, *V*_GS_ = 10 V, while applying 450 nm light with an intensity of 29.0 mW cm^-2^, *t* = 0.6 s; Reading: *V*_DS_ = −3 V, *V*_GS_ = −2 V, *t* = 0.4 s) **h** Long-term stability over more than 12,000 consecutive programming-reading-erasing-reading cycles (cycle period 1.7 s) on the same devices. The device states are shown at selected time points (4000, 8000, 12,000, 16,000, 20,000, and 20,600 s). All bias and illumination conditions are identical to those described in (**g**).

Retention was evaluated by monitoring the read current after programming/erasing (Fig. 3b). The device sustains an on/off current ratio >10^6^ for nearly 50,000 s. Linear extrapolation projects a retention time exceeding 10 years with a ratio >10^4^ meeting commercial standards and outperforming most reported organic memory transistors (Fig. 4e)^28,29,52,54,56,66–68^.

To clarify the storage mechanism, we compared devices with different dielectric stacks. A single Al_2_O_3_ layer shows a larger memory window (11.24 V; Supplementary Fig. 19) and better retention (~10^7^ on/off ratio over 10^4 ^s; Supplementary Fig. 20). EPR signals^69^ (*g* = 2.003; Supplementary Fig. 21) confirm oxygen vacancies in Al_2_O_3_ as deep traps, which are present in both the single Al_2_O_3_ layer and the Al_2_O_3_/PAA heterostructure but absent in the single PAA layer (Supplementary Figs. 22–24). This assignment is further corroborated by XPS O 1 s spectra, where a distinct shoulder peak at ~531.8 eV, characteristic of defect-related oxygen species, is observed exclusively in Al_2_O_3_-containing dielectrics (Supplementary Fig. 25). In PAA/Al_2_O_3_ stacks (PAA on top), thinner PAA gives larger memory windows (~12.8 V; Supplementary Fig. 26) and longer retention (~10^4 ^s; Supplementary Fig. 27a). As the PAA layer thickness increased, the retention time decreased to approximately 10^3^ s (Supplementary Fig. 27b–d). This trend originates from the charge-blocking effect of PAA: a thicker PAA layer attenuates the effective gate field, reducing the number of charges injected and captured by the deep traps in the underlying Al_2_O_3_. Instead, charges are primarily trapped at the PAA/Al_2_O_3_ interface, leading to shortened non-volatile retention (Supplementary Fig. 28). For the Al_2_O_3_/PAA heterostructure (Al_2_O_3_ on top), with PAA fixed at 180 nm, a programming pulse of *V*_GS_ = −20 V (1 s) yielded a memory window of ~10 V for devices with a 30 nm Al_2_O_3_ layer, significantly surpassing the Δ*V*_TH_ of ~6.7 V obtained with a 10 nm Al_2_O_3_ layer (Supplementary Figs. 29, 30d). Current–time (I–T) measurements confirmed excellent non-volatile retention exceeding 10^4^ s for the 30 nm Al_2_O_3_/PAA stack (Supplementary Fig. 30a–c). To directly probe the trap-induced energy level shift and the charge trapping role of oxygen vacancies, we performed Kelvin probe force microscopy (KPFM) on devices with different dielectric configurations. For single Al_2_O_3_ devices (Supplementary Figs. 31 and 32), the surface potential increases stepwise from 22.7 mV to 1.70 V as the programming voltage increases from −10 V to −20 V, confirming strong charge trapping; in contrast, single PAA devices (Supplementary Figs. 33) show negligible change (from 36.1 mV to 36.7 mV) under the same conditions. The Al_2_O_3_/PAA heterostructure (Supplementary Figs. 34 and 35) exhibits a similar stepwise increase (from 26.0 mV to 1.67 V), confirming that it inherits the strong trapping capability of Al_2_O_3_. These KPFM results provide direct evidence of trap-induced Fermi-level shifts and corroborate the charge storage mechanism inferred from the electrical measurements.

This performance arises from two complementary mechanisms: charge trapping in deep-level defects of Al_2_O_3_, and additional charge accumulation at the Al_2_O_3_/PAA interface via Fowler–Nordheim tunneling^70^. The wide bandgap of Al_2_O_3_ further suppresses charge leakage, ensuring long term non-volatility. Moreover, the polar carboxyl (–COOH) groups in PAA create an electron withdrawing interface, which establishes a negative potential that attracts and stabilizes trapped holes, thereby enhancing charge capture efficiency under negative gate pulses. These results demonstrate the charge storage capability of the top Al_2_O_3_ heterostructure design. The storage mechanism is illustrated in Fig. 3c. Erasure is achieved by applying a positive gate voltage together with 450 nm illumination; Fig. 3d depicts the synergistic role of photogenerated holes and the gate field in promoting interfacial detrapping. A summary of optoelectronic metrics for all dielectric configurations is provided in Supplementary Table 2.

To assess the bottom-up modulation role of the polar polymer in the Al_2_O_3_/PAA heterostructure, we compared PAA against four other polymers of decreasing polarity—polyacrylonitrile (PAN), poly(4-vinylphenol) (PVP), polystyrene (PS), and polyphenylene ether (PPO). Each was spin-coated into a lower dielectric layer of comparable thickness (~180 nm) (Fig. 3e, Supplementary Figs. 36–38). Using C10-DNTT as the semiconductor, we systematically characterized and statistically analyzed the electrical performance of the resulting devices. All multilayer structures exhibited p-type transport behavior, with mobility statistics summarized in Supplementary Figs. 39–43. Under identical programming pulse conditions (*V*_GS_ = −50 V, 1 s; Supplementary Figs. 44–49), both the memory window and carrier mobility systematically decreased as the polymer polarity weakened (PAA > PAN > PVP > PS > PPO). directly correlating with the interfacial electron-withdrawing strength. Replacing C10-DNTT with DNTT preserved the same polarity-dependent trends, confirming the generality of this bottom-up modulation mechanism (Supplementary Figs. 50–59). This polarity screening guided us to identify the 30 nm Al_2_O_3_/PAA heterostructure as the optimal interface, which synergistically enhances charge trapping efficiency through PAA’s strongly polar interface while maintaining high carrier mobility—providing an ideal platform for high-performance sensing–memory–processing integrated devices.

Building on the optimized 30 nm Al_2_O_3_/PAA interface, we further evaluated key performance metrics for the heterogeneous dielectric transistors, focusing on multilevel storage, operational speed, and cycling endurance. Applying distinct programming voltages (from −8 V to −18 V) with 10 μs pulses enables six well-separated storage states, which persist for over 10^3^ s, demonstrating potential for multilevel memory applications (Fig. 4a). Fast programming at 10 μs is further corroborated by the well-defined memory windows formed under programming voltages ranging from −10 V to −30 V (Fig. 4b, c). To evaluate the device’s ultra-fast signal detection limits, we performed sub-microsecond pulse measurements using a high-speed pulse module (comprising a signal generator and amplifier) in combination with the semiconductor parameter analyzer. Under −10 V pulses with widths of 5 μs, 1 μs and 130 ns, the device exhibits tunable suppression behavior (Fig. 4d). However, due to the technical limitation of our high-speed pulse module, which cannot provide gate voltages exceeding −10 V at sub-microsecond pulse widths, the −10 V, 1 μs condition yields only a small memory window of 0.38 V (Supplementary Fig. 60). From another perspective, we characterized the rea-time I–T changes before and after applying 1μs electrical pulses (Supplementary Fig. 61), which reveal a retention ability on the second scale, together with oscilloscope readout at 10 ns resolution to capture the instantaneous conductance change (Supplementary Fig. 62). Both measurements confirm the device’s responsiveness to 1 μs electrical signals. Nevertheless, this response is more indicative of transient current suppression, because at such short pulse widths and with the limited voltage amplitude available, stable charge trapping cannot be established. Therefore, it is essential to distinguish the fastest electrical pulse response from stable non-volatile programming. Based on our results, we define 130 ns as the fastest electrical response speed and 10μs as the programming speed for stable multilevel storage. Compared to previously reported organic devices, our device shows a significant advantage in operating speed (Fig. 4f^28,33,52,58,66–68,71,72^), with a distinction between the fastest electrical response and stable multilevel programming. A comprehensive performance comparison is provided in Supplementary Table 3. Moreover, the device shows robust cycling endurance, sustaining an on/off ratio >10 after over 10,000 “program-read-erase-read” cycles (Fig. 4g, Supplementary Fig. 63).

### Neuromorphic recognition based on synaptic properties

**Fig. 5:**
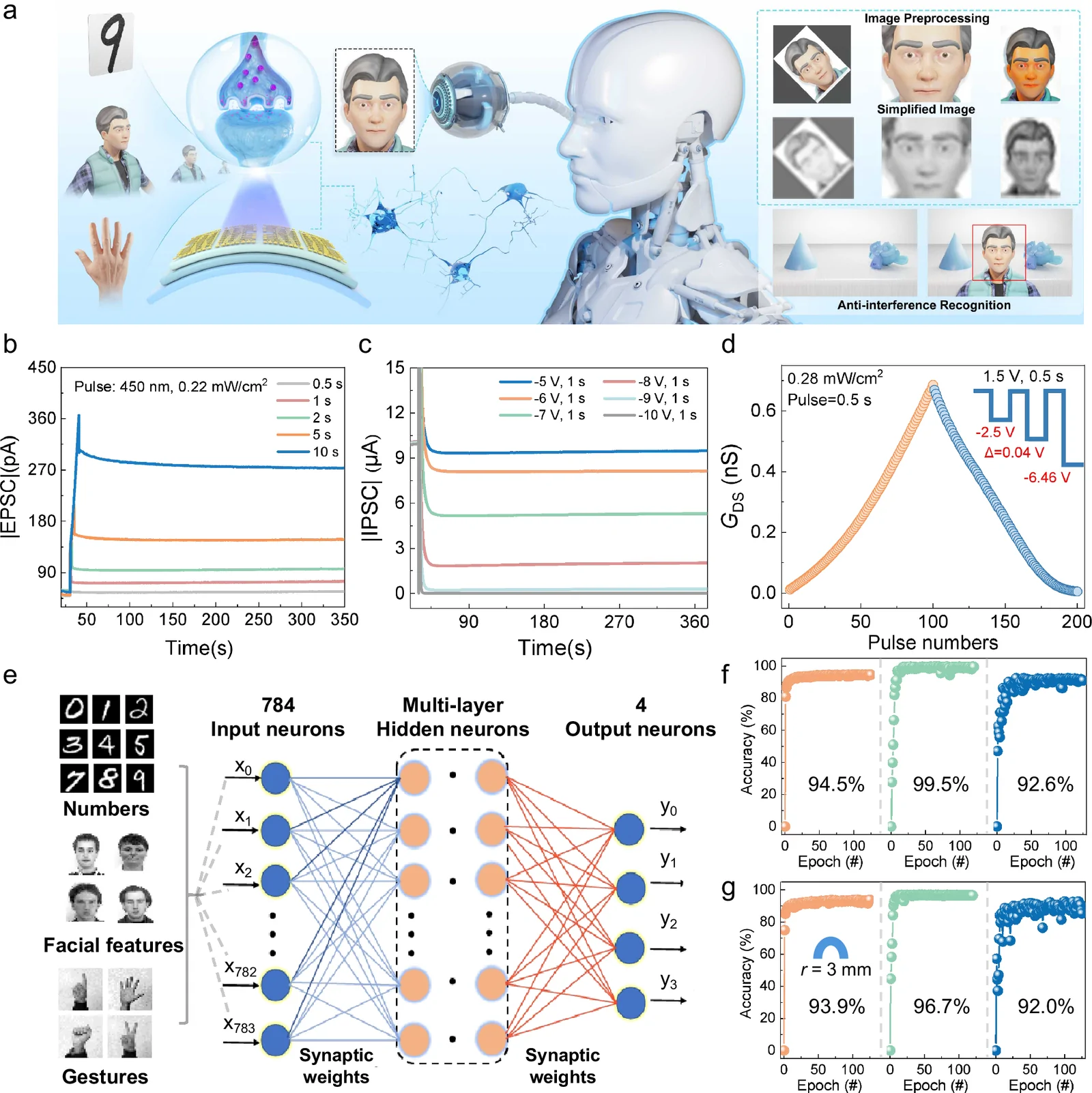
Data processing and recognition based on flexible neuromorphic devices. **a** Integrated sensing-memory-processing vision system: Recognizes and classifies handwritten digits, gestures, and faces. For face recognition, it applies preprocessing (rotation, center cropping, color enhancement) and scales images to 28 × 28 grayscale pixels for neural network training, reducing computation and speeding up training—ideal for resource-constrained scenarios. Based on this, a neural network hardware accelerator is developed, enabling real-time, robust face recognition. **b** Transformation of synaptic plasticity in Al_2_O_3_/PAA-based memory devices trained with increasing light pulse intensity. **c** Inhibitory postsynaptic current (IPSC) of Al_2_O_3_/PAA-based memory devices with increasing pulse voltage of pulses. **d** Light enhancement and electrical depression behavior of Al_2_O_3_/PAA-based memory devices. **e** Schematic of the three-layer artificial neural network (ANN). The representative digit, face, and gesture images used for the neural-network demonstration were obtained from the MNIST database (https://github.com/cvdfoundation/mnist), the Yale Face Database (http://cvc.cs.yale.edu/cvc/projects/yalefaces/yalefaces.html), and the Hand Posture and Gesture Datasets from Idiap Research Institute (https://www.idiap.ch/webarchives/sites/www.idiap.ch/resource/gestures/), respectively. **f** Recognition accuracy for handwritten digits (yellow, left), gestures (green, middle), and faces (blue, right) under non-bending conditions. **g** Recognition accuracy for handwritten digits (yellow, left), gestures (green, middle), and faces (blue, right) when bent at a radius of 3 mm.

Having validated the transistor as an integrated sensing-memory-processing unit, we next explored its neuromorphic computing capabilities (Fig. 5a). The device successfully emulated key synaptic plasticity rules. Short-term plasticity phenomena, including paired-pulse facilitation (PPF) and depression (PPD), were elicited using sequences of optical and electrical stimuli, with their dynamics quantified against inter-pulse intervals (Supplementary Fig. 64). Under extended 450 nm optical training (0.22 mW cm^−2^), the excitatory postsynaptic current (EPSC) transitioned from short-term to long-term plasticity (LTP), as evidenced by increased EPSC amplitude and slowed decay with longer pulses (Fig. 5b). This plasticity could also be modulated by varying light intensity and pulse number (Supplementary Fig. 65). Beyond 450 nm, similar pulse-number-dependent EPSC enhancement was observed at 290 nm, 365 nm and 515 nm (Supplementary Figs. 66), confirming that the optical synaptic plasticity is a general feature across the tested spectral range. The device further emulated a brain-like learning-forgetting-relearning process. After initial potentiation with 100 light pulses, the EPSC naturally decayed. Remarkably, only two pulses were required in a subsequent session to re-reach the same EPSC level, demonstrating efficient memory reconsolidation (Supplementary Fig. 67). This property was leveraged for optical image sensing and memory. Using an array, the character J was sensed and stored, where EPSC levels (represented by pixel intensity) increased with pulse count during learning and faded during forgetting, while retaining a discernible state even after 1000 s (Supplementary Fig. 68). A key advantage for neuromorphic hardware is low energy consumption^73^. The device demonstrated robust synaptic responses at an ultralow operating voltage of −1 mV, with an energy consumption of just 0.06 fJ per synaptic event (Supplementary Fig. 69), significantly outperforming most reported counterparts.

Beyond optical modulation, the synaptic weights were also effectively tuned by electrical gate pulses. A negative presynaptic pulse (*V*_GS_ = −5 V, 1 s) induced a sharp decrease in the postsynaptic current, signifying inhibitory synaptic activity with no recovery over 300 s, confirming long-term depression (LTD) under electrical suppression (Fig. 5c). The degree of this modulation was finely controllable by the pulse amplitude, duration, and number (Supplementary Fig. 70). Critically, the device conductance (*G*_DS_ = EPSC/*V*_DS_) exhibited symmetrical and reversible LTP and LTD, which is essential for artificial synaptic weight updating. As shown in Fig. 5d, channel conductance could be potentiated by 100 consecutive light pulses and subsequently gradually depressed by 100 gate pulses, a stable modulation retained even under mechanical bending (*r* = 3 mm; Supplementary Fig. 71). Since optical stimuli can directly induce nonvolatile potentiation of the device conductance, the sensing input can be locally converted into a synaptic memory state, providing a device-level basis for sensor-memory-computing integration and future in-sensor neuromorphic computing. To further evaluate this potential at the system level, a multilayer perceptron (MLP) network was simulated using experimentally calibrated synaptic-device parameters (Fig. 5e, Supplementary Fig. 72)^74^, was deployed for classification on the MNIST, a hand gesture, and the Yale face datasets (Supplementary Note 1)^75^. For all recognition tasks, the training and testing datasets were strictly separated. The test samples/subjects for handwritten digit, gesture and face recognition were excluded from the training process and were not used for parameter optimization. The system achieved high recognition accuracies of 94.78%, 99.83%, and 92.56% (Fig. 5f), respectively, for the flat device, and retained robust performance (93.88%, 96.67%, 91.96%) under mechanical bending (*r* = 3 mm; Fig. 5g). These results validate the system’s capability for complex visual pattern recognition and highlight its significant potential for practical, flexible neuromorphic computing applications.

### Real-time face detection based on hardware accelerator

While convolutional neural networks (CNNs) have achieved considerable success in civilian domains such as facial recognition and autonomous driving, their deployment in aerospace applications has relied extensively on field-programmable gate arrays (FPGAs), which offer inherent parallelism, reconfigurability, and low power consumption for energy-efficient convolutional acceleration in spaceborne environments. However, the limited capacity of FPGA on-chip memory poses a bottleneck for meeting the large data requirements of convolutional processing—a limitation we address by incorporating a device-aware synaptic weight model derived from experimentally measured OTFT conductance plasticity into an FPGA-based inference framework. By minimizing control logic and retaining only essential arithmetic and storage units, our dataflow-driven architecture establishes a hardware-oriented inference and evaluation pipeline that substantially reduces off-chip memory access overhead. This design offers high intrinsic energy-efficiency potential within an ultra-low power envelope, offering a scalable solution for resource-constrained embedded and edge computing applications. It should be noted that the energy-efficiency analysis in this work mainly concerns the core computing module and does not explicitly include the overhead of peripheral circuits; therefore, the practical system-level efficiency may be lower and remains to be evaluated in future hardware implementations.

**Fig. 6:**
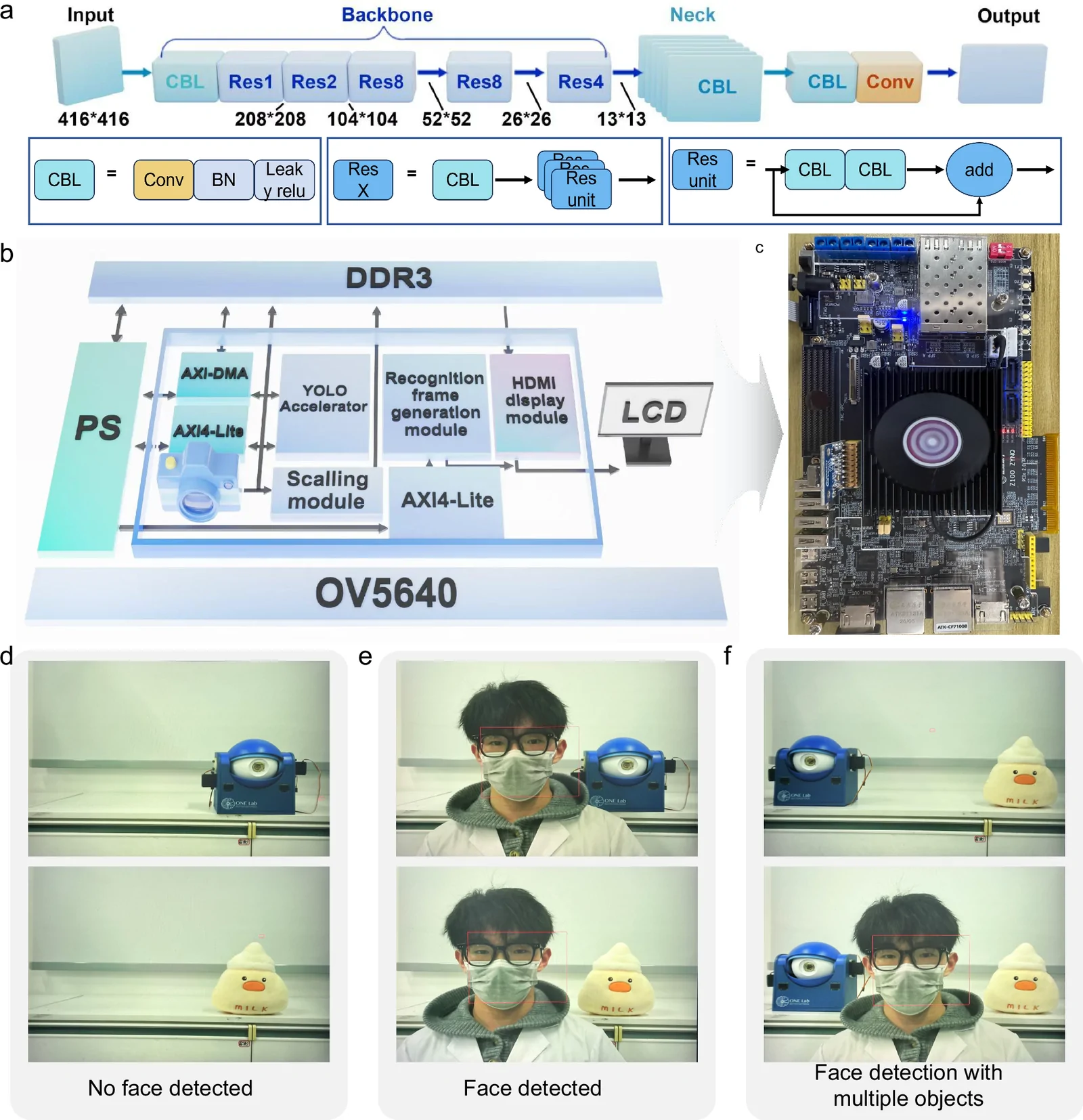
Implementation and demonstration of the hardware-accelerated system for real-time face detection. **a** Schematic diagram of the YOLO neural network architecture, illustrating the hierarchical structure and feature map dimension evolution from the input stage through the Backbone feature extraction network and Neck network to the Output layer. **b** Hardware emulation flow based on experimentally measured OTFT synaptic characteristics. Experimentally characterized OTFT conductance plasticity is first converted into a device-aware synaptic model, which is then mapped onto the FPGA-based inference engine for system-level neural network evaluation. The OTFT devices provide the physical basis for synaptic weight modeling, whereas the actual inference operations are executed on the Zynq FPGA platform. **c** Photograph of the experimental hardware platform constructed using a Zynq SoC. **d**–**f** Validation results of real-time system detection performance. **d** Negative control tests on non-face targets (e.g., dolls and mechanical eyes) demonstrating no false positive detections. **e** Positive detection of face targets in mask-wearing scenarios showing successful recognition. **f** Demonstration of multi-target detection in a complex background containing various interfering objects, including zoomed-in views of the detected face regions.

As a demonstration of this paradigm, we developed a specialized hardware accelerator tailored for real-time face detection, implementing a lightweight YOLOv3-tiny architecture (Fig. 6a). The accelerator leverages the intrinsic LTP/LTD characteristics of the experimentally measured OTFT synaptic characteristics are used to construct a device-aware weight model, while the actual convolutional and fully connected operations are executed on FPGA, thereby demonstrating the feasibility of mapping algorithmic workloads onto synaptic hardware. The system is orchestrated on a Zynq SoC platform using a hardware-software co-design paradigm (Fig. 6b, c), where the Programmable Logic (PL) executes intensive convolution and pooling kernels while the Processing System (PS) manages data scheduling and output parsing. High-definition video streams are down sampled and buffered in DDR3 memory, processed through the FPGA-based device-aware accelerator, and the resulting coordinates are transmitted via the AXI4-Lite protocol for low-latency visual overlay, establishing a complete pipeline for edge-based intelligence. The practical efficacy and robustness of this synaptic hardware accelerator were substantiated through rigorous real-time inference benchmarks (Fig. 6d–f and Supplementary Movie 2). We evaluated discriminatory specificity by introducing non-face artifacts with morphological resemblances to human features, such as dolls and mechanical eyes; the system exhibited high precision by successfully suppressing these false positives (Fig. 6d). Furthermore, the accelerator demonstrated robustness in occlusion scenarios, accurately localizing targets wearing medical masks by leveraging high-level semantic features (Fig. 6e). Even in cluttered environments containing simultaneous distractors and multiple targets (Fig. 6f), the system maintained zero-false-positive and zero-false-negative performance, confirming that can provide reliable and accurate performance for complex visual detection tasks.

## Discussion

This work fabricates an Al_2_O_3_/PAA heterostructure dielectric and reveals the critical bottom-up modulation effect of polar polymers in OTFTs. Through systematic comparison of different dielectric configurations and sequences, the heteroelectric dielectric layer structure with Al_2_O_3_ as the top layer is optimized, achieving an balance between efficient charge trapping (via deep defects and an electron-withdrawing interface) and high carrier mobility (22.65 cm^2 ^V^–1^ s^−1^). The resulting flexible device integrates “sensing-memory-processing” capabilities: a fast optical response (30 μs), electrical response with a fast-switching speed (130 ns) and a stable programming speed (10 μs), stable non-volatile memory (retention >10 years, endurance >10^4^ cycles), and robust neuromorphic functions (PPF/PPD, LTP/LTD). These attributes enabled the construction of an accurate artificial neural network for visual pattern recognition. Furthermore, by integrating the synaptic devices with a dedicated hardware accelerator on a Zynq SoC platform, we demonstrated a complete edge-intelligence pipeline for real-time, occlusion-robust face detection with high precision and reliability. This study provides a foundational hardware component and a validated system architecture for next-generation energy-efficient edge computing in applications such as intelligent surveillance and autonomous systems.

## Methods

### Materials

Substrate Preparation: Glass/ITO or PI/ITO substrates were ultrasonic cleaned in deionized water, acetone and isopropanol, respectively, and then dried with nitrogen, then treated with 50 W O_2_ plasma for 5 min (plasma treatment was carried out using NAEN TECH). PAA thick thin films were prepared by spin coating in the N_2_ environment provided by the glove box. PAA dielectric layer preparation: Poly (amic acid) (PAA) was synthesized by Beijing University of Chemical Technology. On the cleaned ITO conductive glass (20 mm × 20 mm × 1.1 mm, square resistance <10 Ω, transmittance >83%) surface spin coating PAA solution (PAA and DMAC according to the 1: 3 mass ratio of mixing and become), spinning speed of 5000 r/min, time of 120 s, spinning and coating, drying a few hours after the solvent DMAC to be completely evaporated, to obtain 180 nm-thick PAA. The growth of Al_2_O_3_ on PAA layer by PEALD: the substrate temperature was heated to 80 °C, the pipeline temperature was heated to 180 °C, and the precursor pipeline was heated to 120 °C during growth. The pressure inside the chamber was maintained at 0.25 Torr during the reaction, and high-purity Ar (99.999%) was used as the carrier gas and precursor cleaning gas for the reaction, with a carrier gas flow rate of 45 sccm (std. ml/min) for trimethylaluminium (TMA, 99.9999%), and a carrier gas flow rate of 90 sccm for the O_2_ plasma. throughout the PELAD process, the TMA was maintained at room temperature. O_2_ plasma was generated at 15 sccm O_2_ (99.999%) at 100 W RF power. The PEALD (Plasma Enhanced Atomic Layer Deposition System) model is PEALD-150A, produced from Jiaxing Kemin, Zhejiang Province. Trimethylaluminium (TMA) was obtained from Shanghai Titan Technology Co., Ltd, high-purity argon (Ar) was obtained from Tianjin Liufang Industrial Gases Distribution Co., Ltd, and oxygen (O_2_) was obtained from Dongrun Gas Sales (Tianjin) Co.

### Device fabrication

20 nm C10-DNTT (Purchased from Hangzhou Aude Technology) was vapor-deposited as a semiconductor layer on an Al_2_O_3_-PAA heterogeneous dielectric layer using a ZD-400 single-chamber, high-vacuum, resistive vacuum deposition equipment at a rate of 0.04 Å/s under a vacuum of 2 × 10^−4 ^Pa. The C10-DNTT surface was then covered with a metal mask, and 20 nm Au was vapor-deposited as a source/drain at a rate of 0.07 Å/s under a vacuum of 2 × 10^−4^ Pa. Completed the preparation of “sensing, memory and computing” integrated transistors.

### Electrical characterization

The electrical properties of the equipment were tested by Keithley 2636B System in air environment and the mobility was calculated by using the saturation zone equation [*I*_DS_ = *μ C*_i_ (*W*/2 *L*) (*V*_GS_-*V*_th_)^2^]. Capacitance-voltage measurements were performed with a DEMKIT E4980AL LCR meter. For phototransistor and photonic synapse characterization, a Keithley 2636B semiconductor parameter analyzer was used, with illumination from a Zolix MLED4-3 LED and a xenon lamp. In the fast optical response test, a function signal generator (Tektronix) and a laser were used to provide fast laser pulses, and the current signals were acquired using the PDA FS-Pro semiconductor parameter analyzer. For electrical pulse response down to 130 ns, the signal was applied using a combination of instruments from PRIMARIUS: a semiconductor parameter analyzer, a signal amplifier, and a fast pulse module (model FS-ProFS430-PAMUHP-FWGMK). All electrical measurements and characterizations were performed in ambient air at 25 °C and with a relative humidity of 20%–30%.

### Informed consent for publication of identifiable images

The authors affirm that human research participants provided informed consent for publication of the images in Figs. 6e, f. No images of children or minors are included in this study.

## Supplementary information

Supplementary Information

Description of Additional Supplementary Files

Supplementary Movie 1

Supplementary Movie 2

Transparent Peer Review file

## Data Availability

The public datasets used for the neural-network demonstrations in Fig. 5e and Supplementary Fig. 72 are available from the following sources: the MNIST handwritten digit dataset, originally derived from the National Institute of Standards and Technology database and accessed via the CVDF mirror (https://github.com/cvdfoundation/mnist); the Hand Posture and Gesture Datasets from Idiap Research Institute (https://www.idiap.ch/webarchives/sites/www.idiap.ch/resource/gestures/); and the Yale Face Database from Yale University (http://cvc.cs.yale.edu/cvc/projects/yalefaces/yalefaces.html). All source data generated in this study and underlying the figures/charts are provided in the Source Data files. The raw data for the main figures are available in Figshare (10.6084/m9.figshare.32957597) under CC BY 4.0.

## Code availability

The code that supports the findings of this study have been deposited in Figshare under the DOI (10.6084/m9.figshare.32957597) and are freely accessible under the Creative Commons Attribution 4.0 International (CC BY 4.0) license. The repository contains all raw data underlying the main figures and the code used for data analysis and neural network simulations. In addition, the neural network simulation for Fig. 5e, f was performed using the NeuroSim framework (version 1.0), which was developed by Prof. Shimeng Yu’s group at the Georgia Institute of Technology and is publicly available for non‑commercial use (https://github.com/neurosim/neurosim).
