# Supplementary material for: Flexible organic thin-film transistors for all-in-one retinomorphic acceleration enabled by inorganic-organic heterogeneous dielectrics
Source: Nat Commun. 2026 Jul 27;17:9124. doi: 10.1038/s41467-026-76116-z (PMC13518895; doi:10.1038/s41467-026-76116-z)
Supplement: Supplementary file 1 [file 41467_2026_76116_MOESM1_ESM.pdf]

Flexible Organic Thin-Film Transistors for All-in-One Retinomorphic Acceleration Enabled by Inorganic-Organic Heterogeneous Dielectrics

Yilin Zhao,[1#] Dongyang Zhu,[1#] Ting Jiang,[1#] Le Wang,[2#] Ruiheng Wang,[2] Guanran Wang,[3] Yu Duan,[3*] Haifeng Ling,[2*] Deyang Ji,[1, 4*] Wenping Hu[5, 6]

[1] Institute of Molecular Aggregation Science, Tianjin University, Tianjin 300072, China.

[2] State Key Laboratory of Flexible Electronics & Jiangsu Key Laboratory of Neuromorphic Electronics, Institute of Advanced Materials, Nanjing University of Posts & Telecommunications, Nanjing 210023, China.

[3] State Key Laboratory of Integrated Optoelectronics, College of Electronic Science and Engineering, Jilin University, Changchun 130012, China.

[4] Key Laboratory of Organic Integrated Circuit, Ministry of Education, Tianjin University, Tianjin 300072, China.

[5] State Key Laboratory of Advanced Materials for Intelligent Sensing, Tianjin University, Tianjin, 300072, China.

[6] Tianjin Key Laboratory of Molecular Optoelectronic Sciences, Department of Chemistry, School of Science, Tianjin University, Tianjin 300072, China.

Corresponding authors: Deyang Ji (jideyang@tju.edu.cn), Yu Duan (duanyu@jlu.edu.cn) and Haifeng Ling (iamhfling@njupt.edu.cn).

# These authors contributed equally to this work.

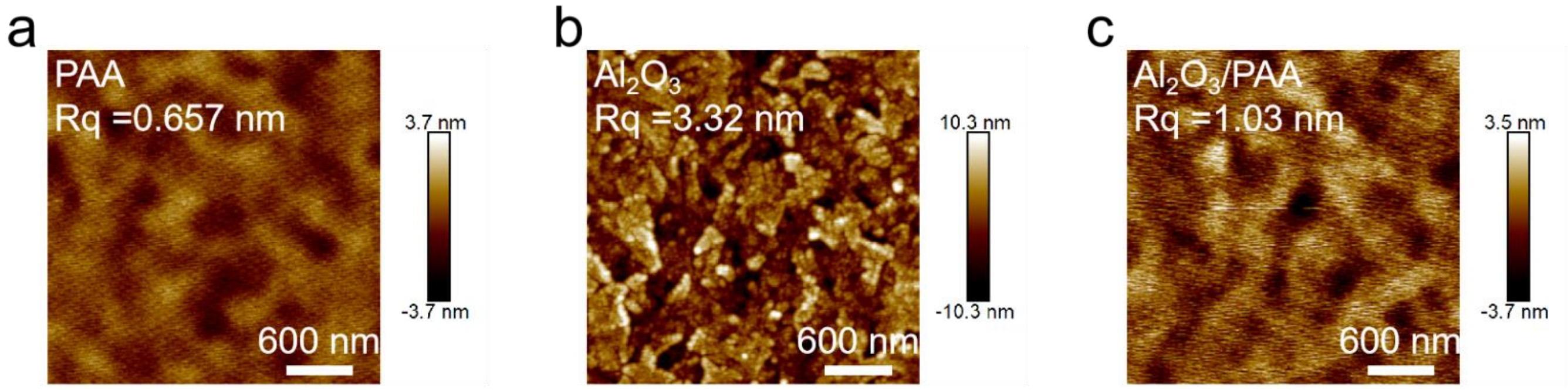

Supplementary Fig. 1 | AFM topography images of PAA, Al_2O_3, and Al_2O_3/PAA films on ITO substrates. **a**, PAA film. **b,** Al_2O_3 film. **c**, Al_2O_3/PAA bilayer film.

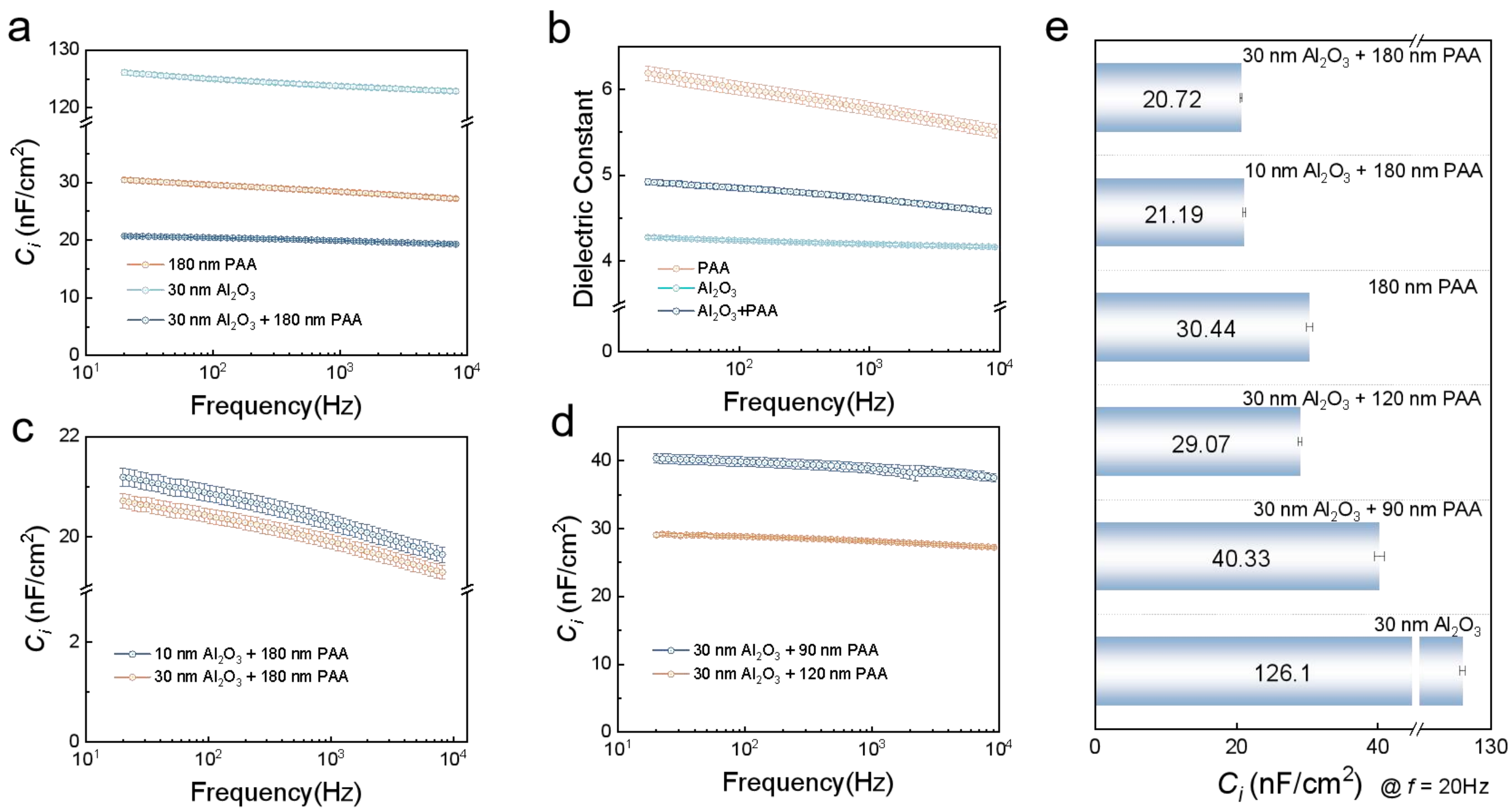

Supplementary Fig. 2 | Capacitance and dielectric constant of Al_2O_3/PAA dielectric layers as a function of frequency. a, C_i of PAA, Al_2O_3, and Al_2O_3/PAA bilayers. **b,** ε_r of PAA, Al_2O_3, and Al_2O_3/PAA bilayers. **c,** C_i of Al_2O_3/PAA with 10-nm and 30-nm Al_2O_3 (fixed 180-nm PAA). **d,** C_i of Al_2O_3/PAA with 90 nm and 120 nm PAA (fixed 30 nm Al_2O_3). **e,** C_i summary at 20 Hz for all thickness combinations.

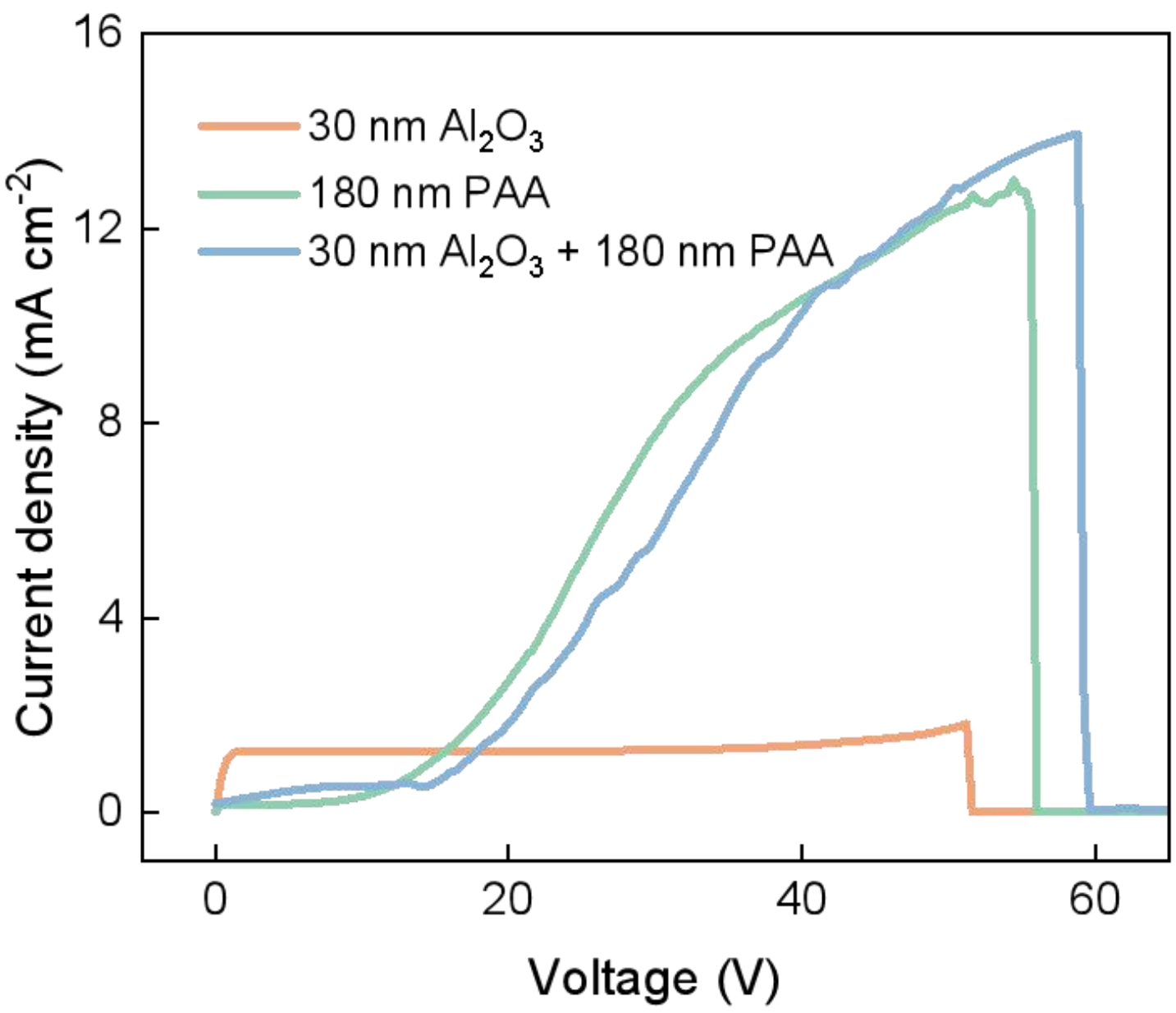

Supplementary Fig. 3 | Breakdown voltage and current density of 30nm Al_2O_3, 180nm PAA and 30nm Al_2O_3/180nm PAA heterogeneous dielectric layers. The heterogeneous dielectric layer exhibits a breakdown voltage of 58.8 V and a leakage current density of 13.92 mA cm^{-2}, outperforming both single-layer PAA and single-layer Al_2O_3 films of equivalent thickness.

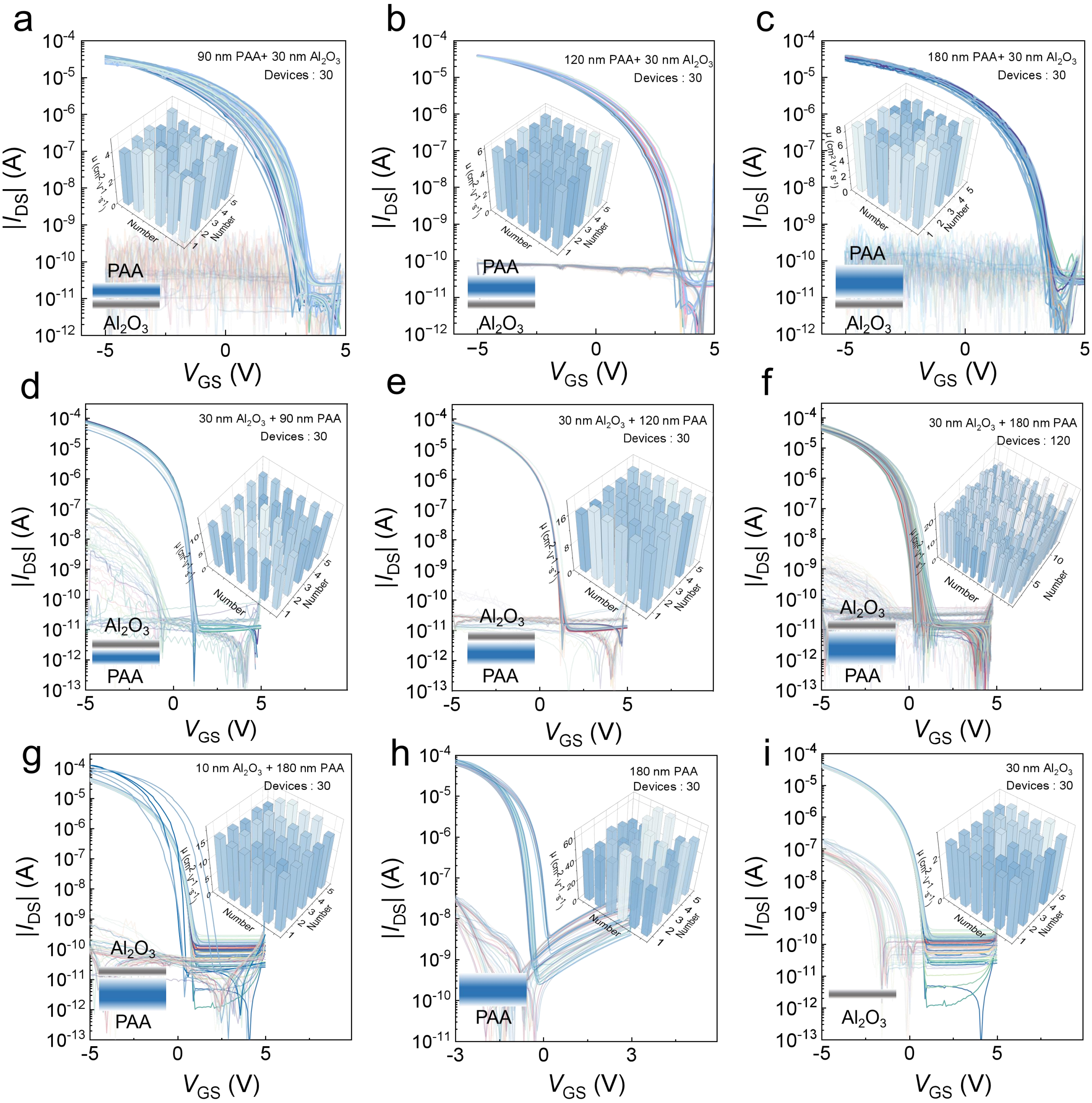

Supplementary Fig. 4 | OTFTs performance with Al_2O_3/PAA dielectrics of different thicknesses and stacking orders. a–c, PAA/Al_2O_3 with PAA thickness of 90, 120, and 180 nm, respectively (Al_2O_3 fixed at 30 nm). **d–f,** Al_2O_3/PAA with PAA thickness of 90, 120, and 180 nm, respectively (Al_2O_3 fixed at 30 nm). **g,** Al_2O_3/PAA (10 nm/180 nm). **h,** PAA only (180 nm). **i,** Al_2O_3 only (30 nm).

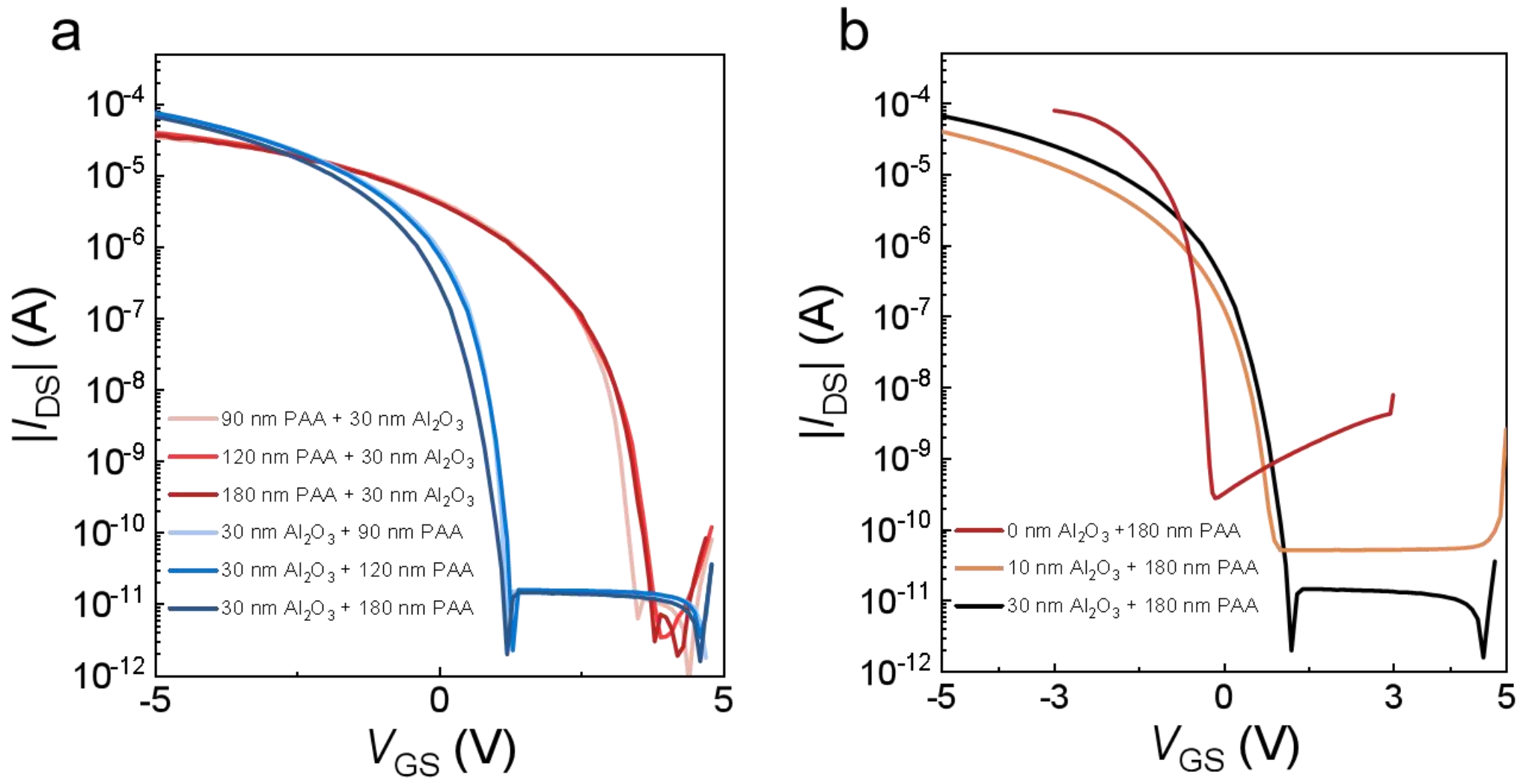

Supplementary Fig. 5 | Transfer characteristics of OTFTs with Al_2O_3/PAA heterostructure dielectrics under different layer engineering conditions. a, Effect of varying PAA thickness and layer order (Al_2O_3/PAA vs. PAA/Al_2O_3). **b,** Effect of varying Al_2O_3 thickness.

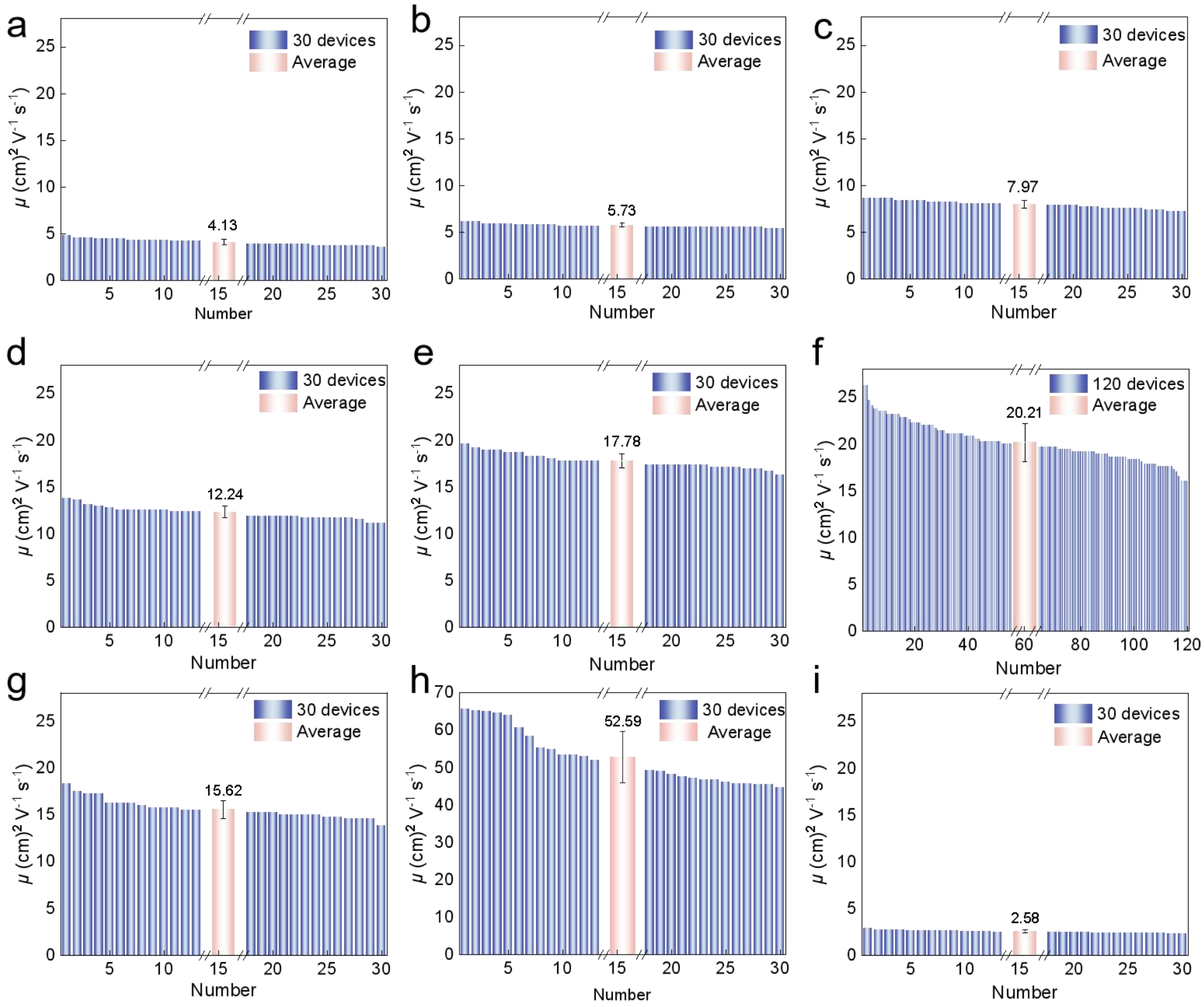

Supplementary Fig. 6 | Statistical summary of OTFTs mobility for each dielectric configuration in Supplementary Fig. 4, with capacitance measured at *f*= 20 Hz. a, 90 nm PAA/30 nm Al_2O_3. **b,** 120 nm PAA/30 nm Al_2O_3. **c,** 180 nm PAA/30 nm Al_2O_3. **d,** 30 nm Al_2O_3/90 nm PAA. **e,** 30 nm Al_2O_3/120 nm PAA, **f,** 30 nm Al_2O_3/180 nm PAA. **g,** 10 nm Al_2O_3/180 nm PAA. **h,** 180 nm PAA. **i,** 30 nm Al_2O_3.

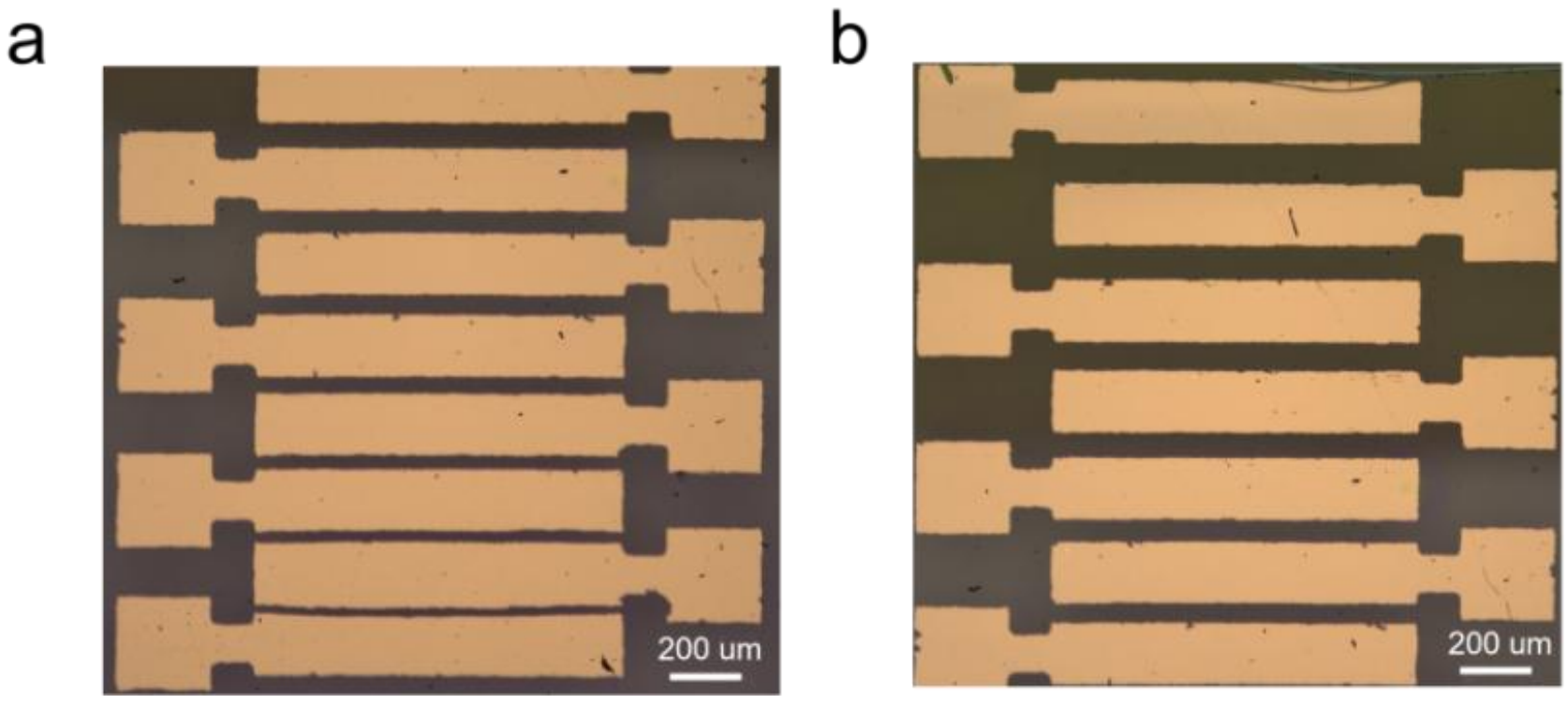

Supplementary Fig. 7 | Optical microscopy image of the TLM electrode array. Channel widths are fixed at W=1 mm, with channel lengths varying from L=30 μm to 130 μm (30, 40, 50, 60, 70, 80, 90, 110 and 130 μm) for contact resistance extraction.

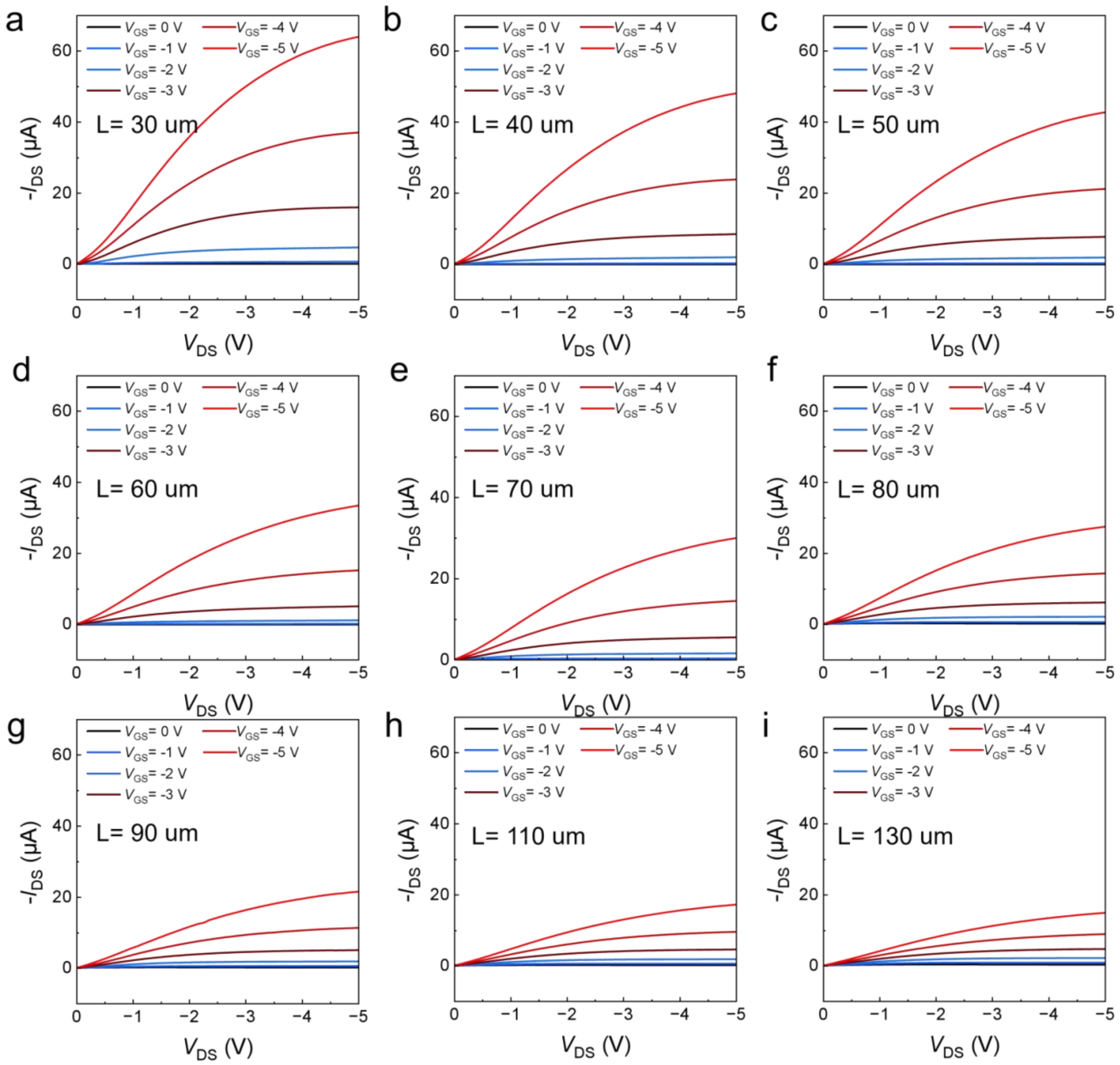

Supplementary Fig. 8 | Output characteristics of TLM devices with varying channel lengths (L=30–130 μm, W=1 mm). a-i, I_{DS}-V_{DS} curves measured at different V_{GS} for devices with channel lengths of 30 μm (**a**), 40 μm (**b**), 50 μm (**c**), 60 μm (**d**), 70 μm (**e**), 80 μm (**f**), 90 μm (**g**), 110 μm (**h**), and 130 μm (**i**). The linear behaviour in the low-bias regime confirms ohmic contacts, validating the subsequent TLM extraction of contact resistance.

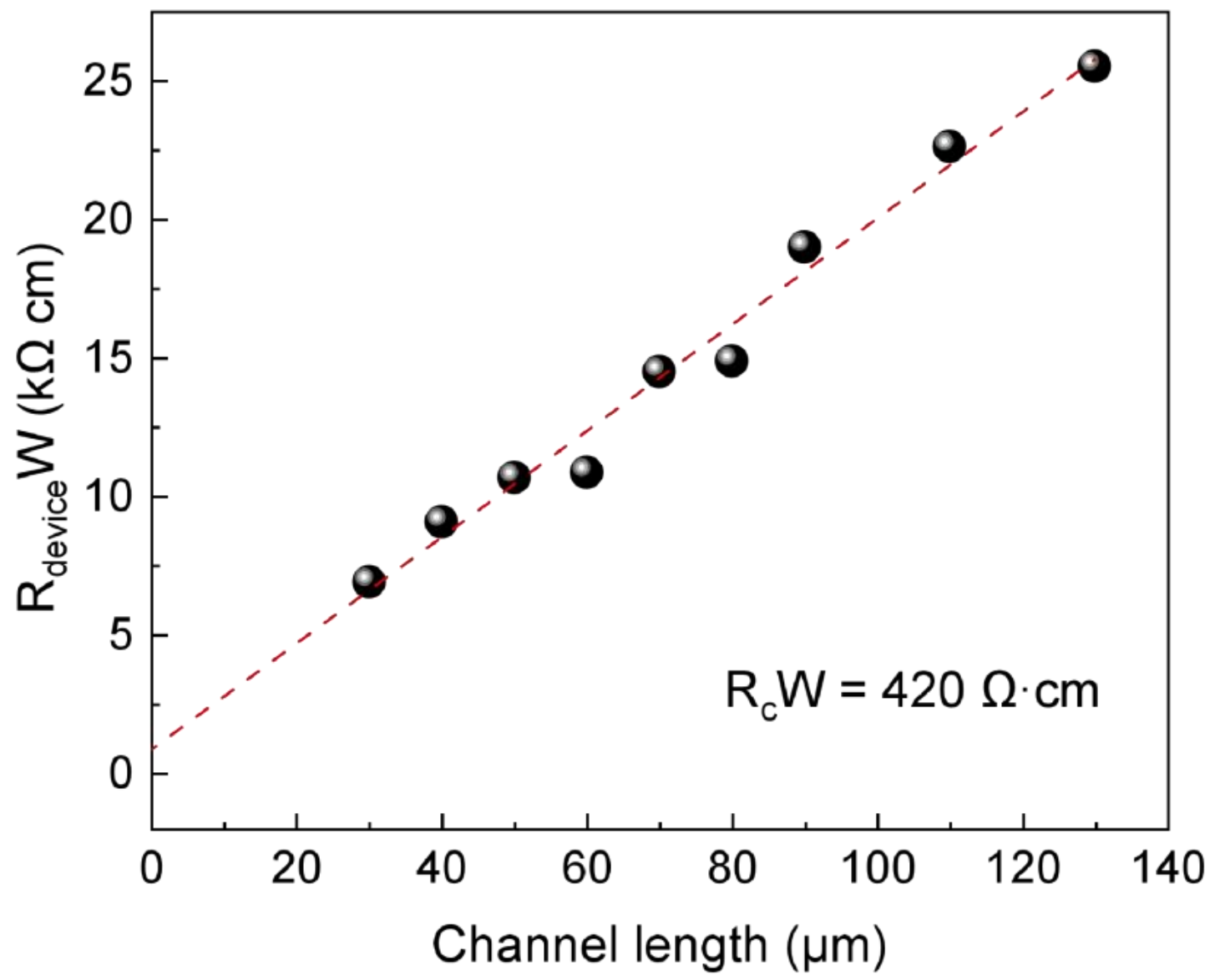

Supplementary Fig. 9 | Total on-resistance R_{ON} as a function of channel length L extracted from TLM measurements. The linear fit of R_{ON} versus L yields a channel-width-normalized contact resistance of R_{ON} = 420 Ω cm, confirming ohmic contacts and validating the extracted mobility values.

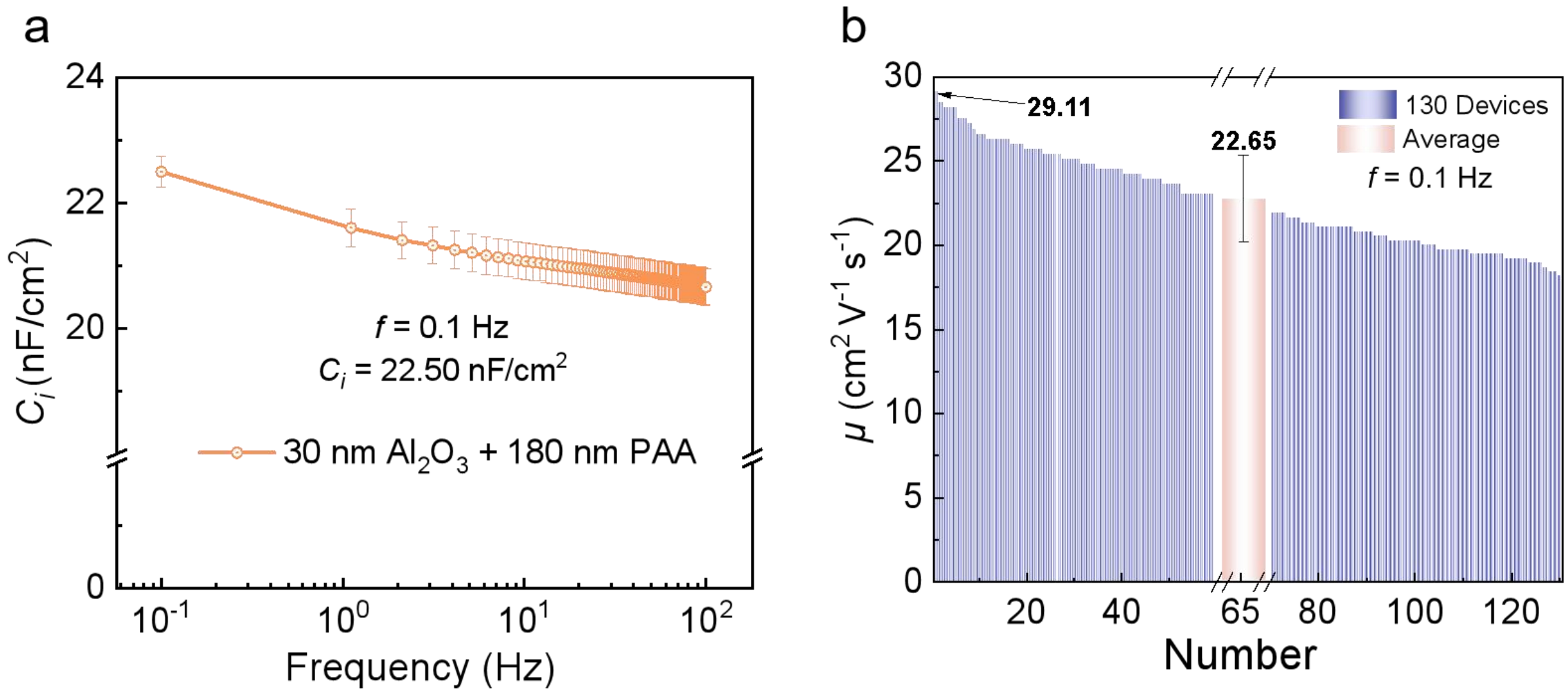

Supplementary Fig. 10 | Electrical characterization of Al_2O_3/PAA bilayer dielectrics on flexible substrates. a, Capacitance–frequency (C–f) characteristics measured at low frequencies. **b,** Mobility distribution of 130 devices extracted from transfer curves (tested at 0.1 Hz).

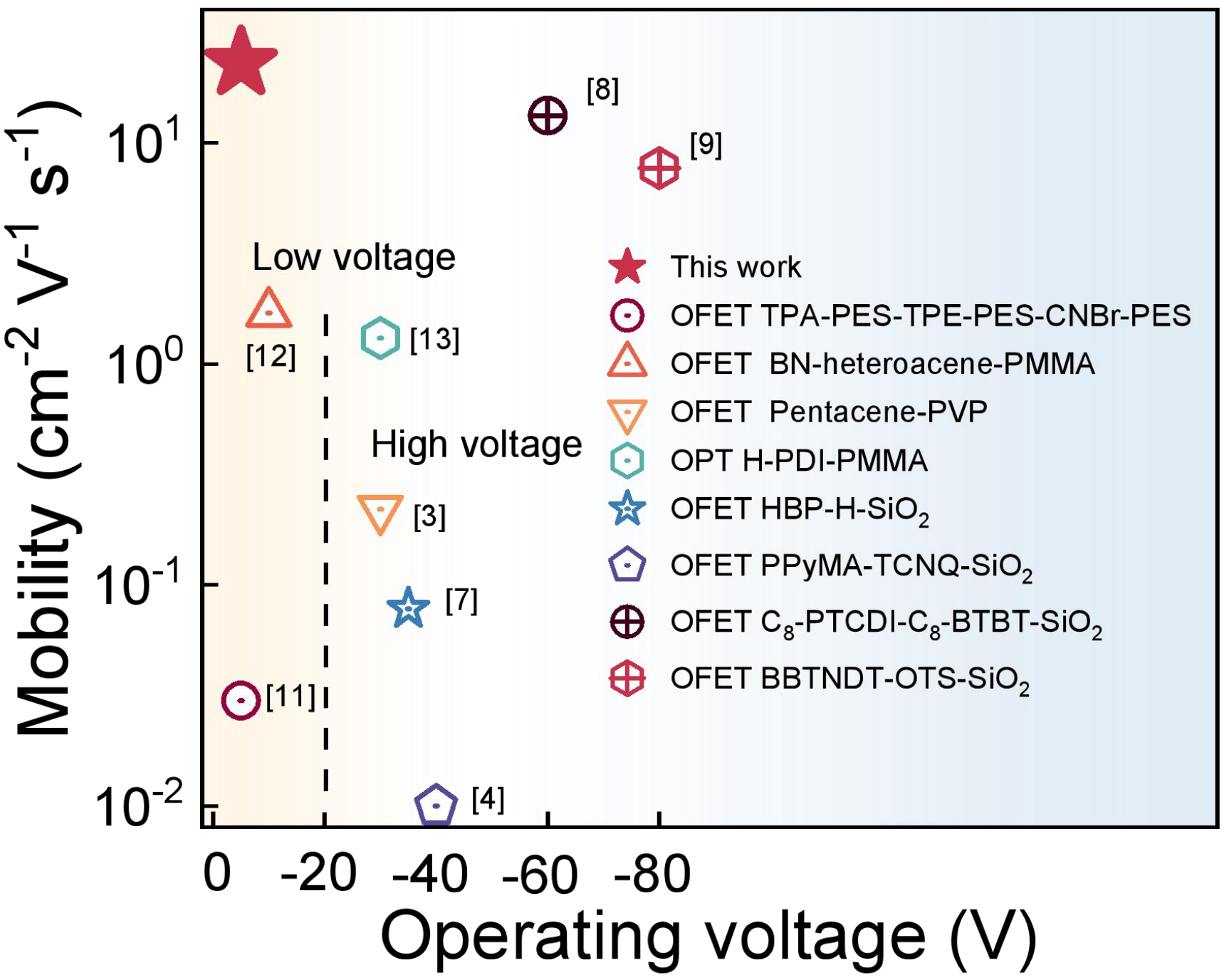

Supplementary Figure 11 | Comparison of carrier mobility for representative organic transistor memory devices. Statistical distribution of mobility values reported for organic transistors. For reference, the present work achieves an average mobility of 22.65 cm^2 V^{-1} s^{-1} at an operating voltage of −5 V, as indicated by the red data point in the distribution.

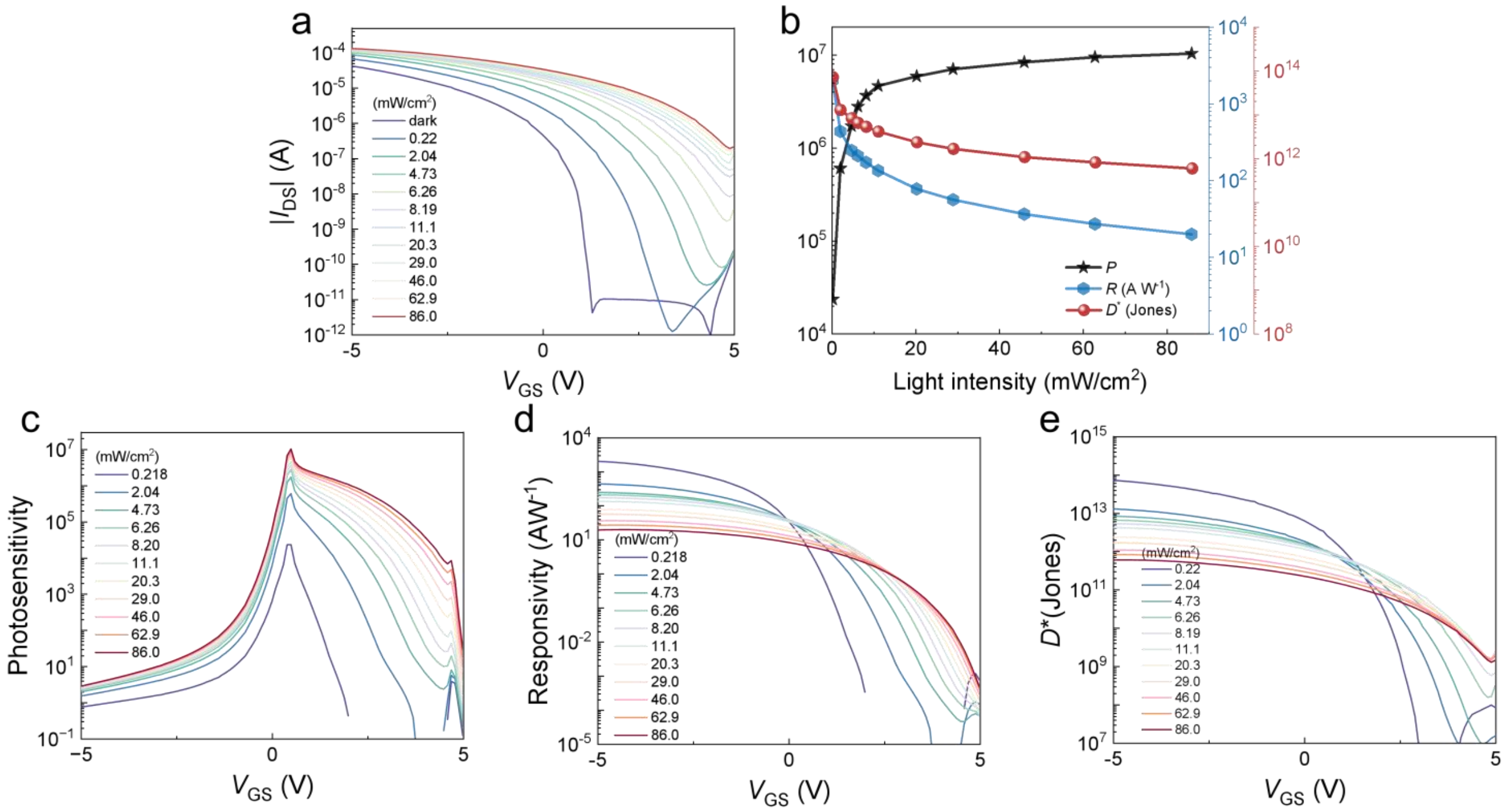

Supplementary Fig. 12 | Vacuum deposition of 20 nm C10-DNTT semiconductor on 30 nm Al_2O_3/180 nm PAA film based on flexible substrate, followed by deposition of 20 nm Au electrodes to prepare OTFTs and test optical response. (**a**) Transfer curves of devices at different optical power densities, (**b**) Statistics of *P*, *R*, *D** values of the device at different optical power densities, (**c**) *P*-value of the device at different optical power densities, (**d**) *R*-value of the device at different optical power densities, and (**e**) *D**-value of the device at different optical power densities.

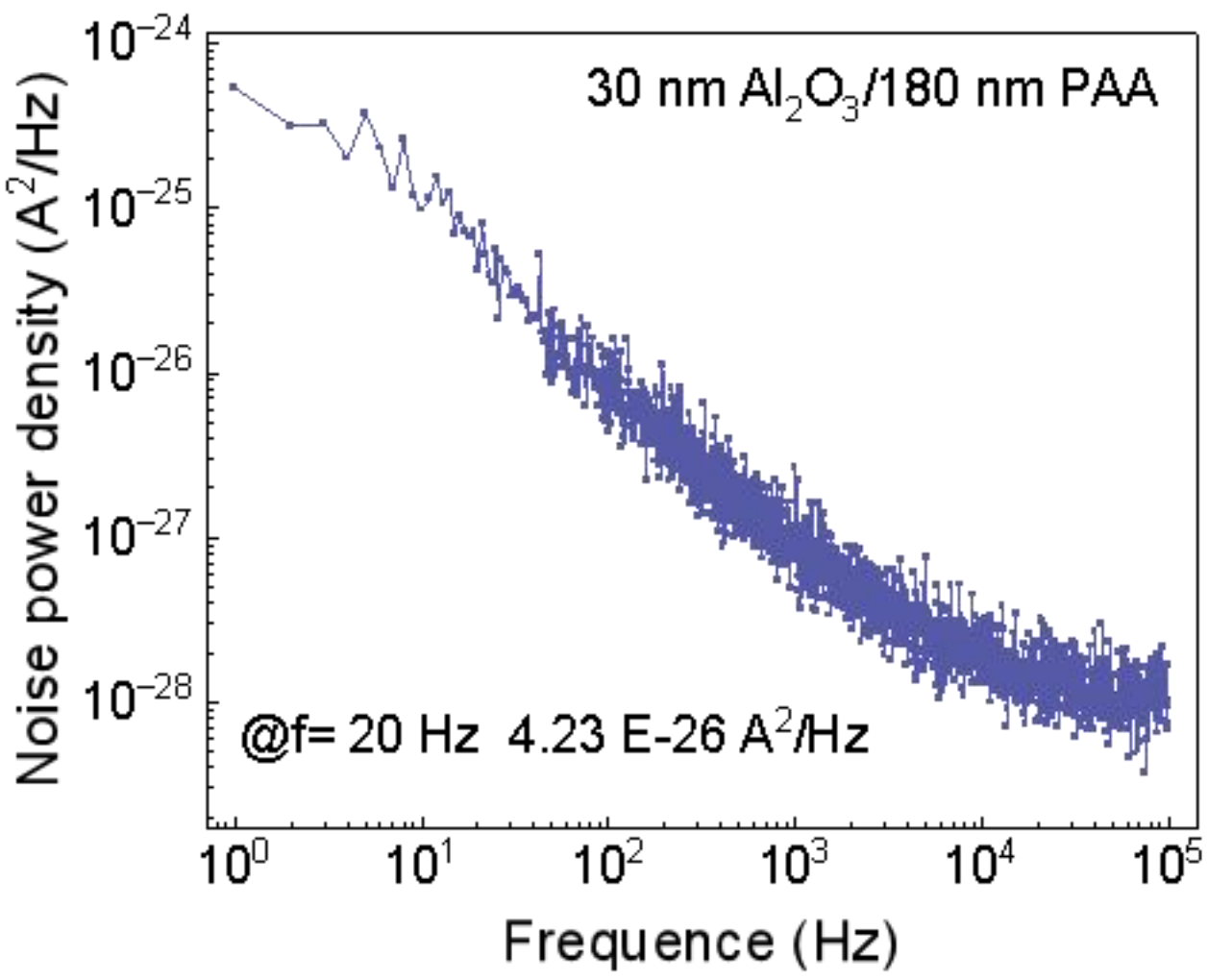

Supplementary Fig. 13 | Frequency-dependent noise current spectral density of the device measured under dark conditions. The noise power spectral density (S_i) is plotted as a function of frequency. The measurement was performed at a fixed bias of V_{DS}= -5 V and V_{GS}= 0 V in the dark. The noise current (i_n) used for detectivity calculations was extracted at 20 Hz, yielding a value of i_n= 2.05×10^{-13} A. (corresponding to $S_n = i_n^2$=4.23×10^{-26} A^2/Hz)

Based on the measured noise current, we calculated the noise-equivalent power (NEP) and D^* using the following standard formulae:

$$NEP = \frac{i_n}{R} \quad (1)$$

$$D^* = \frac{\sqrt{A}}{NEP} \quad (2)$$

where: i_n is the RMS noise current (A), R is the responsivity (A W^{-1}), A is the effective channel area (cm^2). The measured dark-state noise current of our device is i_n =2.05×10^{-13} A. Using this value, we recalculated the specific detectivity for all measured wavelengths to ensure a rigorous and consistent comparison.

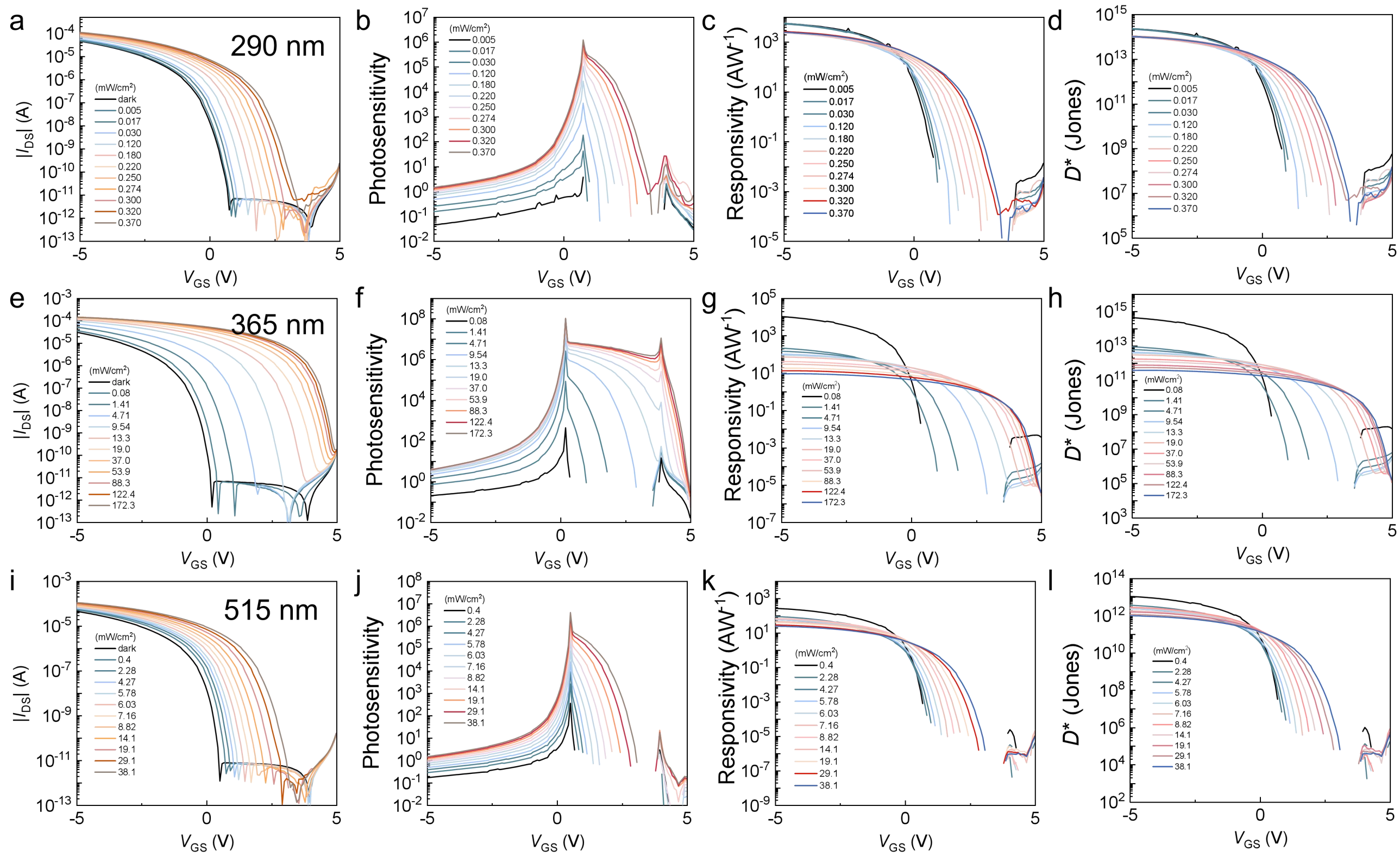

Supplementary Fig. 14 | Multi-wavelength photoresponse characteristics of the 30 nm Al_2O_3/180 nm PAA device. Photoresponse performance was systematically characterized under monochromatic illumination at 290 nm, 365 nm, and 515 nm. **a,e,i,** Transfer curves measured in the dark and under illumination at 290 nm (**a**), 365 nm (**e**), and 515 nm (**i**). **b,f,j,** Corresponding *P* values extracted from the transfer curves. **c,g,k**, *R* values. **d,h,l,** Noise-corrected *D** values derived from the directly measured dark-state noise current (i_n= 2.05×10^{-13} A) The device exhibits clear photoresponse across all tested wavelengths.

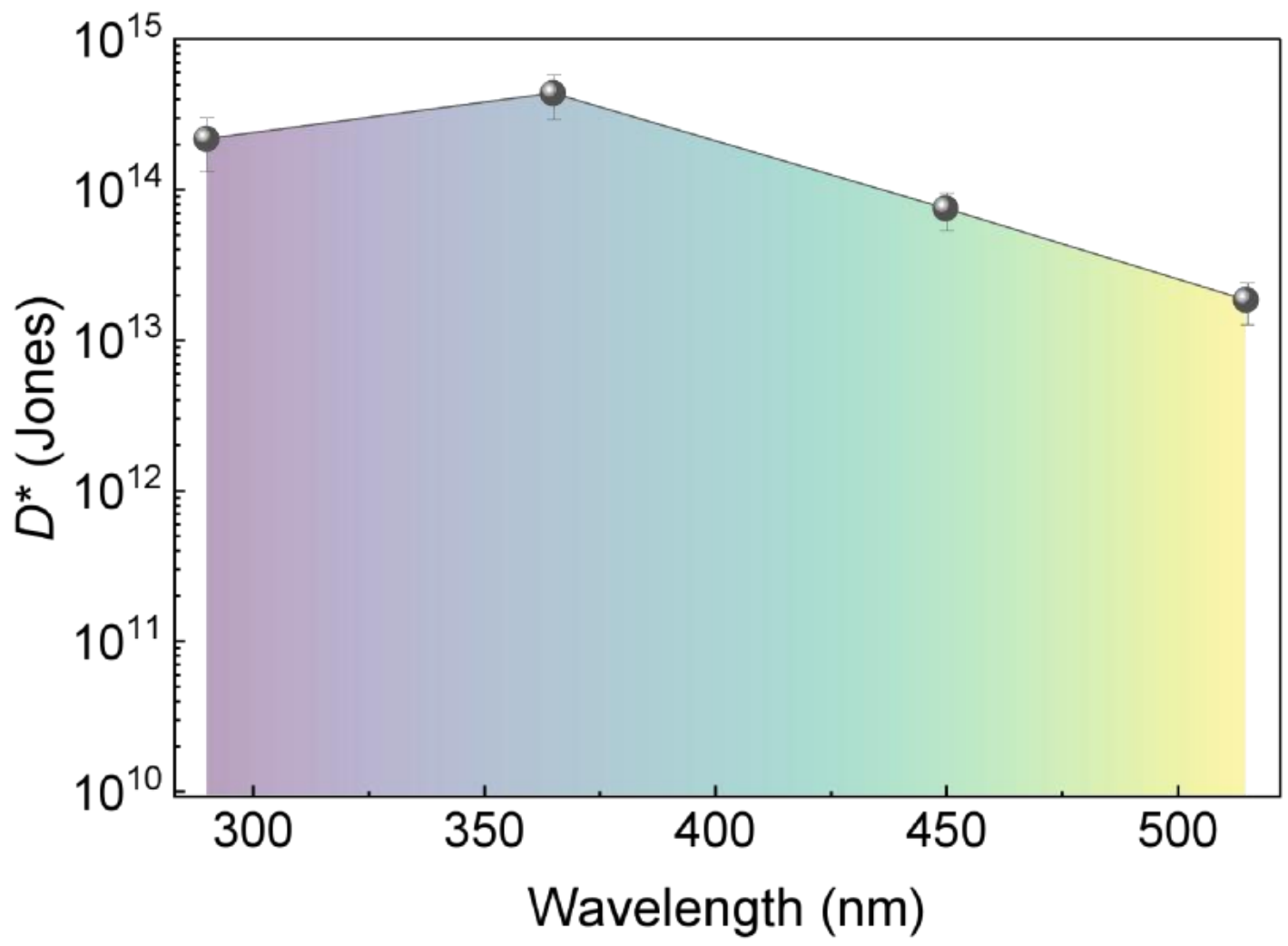

Supplementary Fig. 15 | Statistical distribution of *D at different wavelengths for the 30 nm Al_2O_3/180 nm PAA device.** The chart summarizes the device-to-device variation of noise-corrected *D** values measured at 290 nm, 365 nm, 450 nm and 515 nm, compiled from multiple devices. Error bars represent the standard deviation of the extracted *D** values. The device exhibits the highest *D** of 4.4×10^{14} Jones at 365 nm, with consistent performance across the tested spectral range. All measurements were performed at an operating voltage of −5 V.

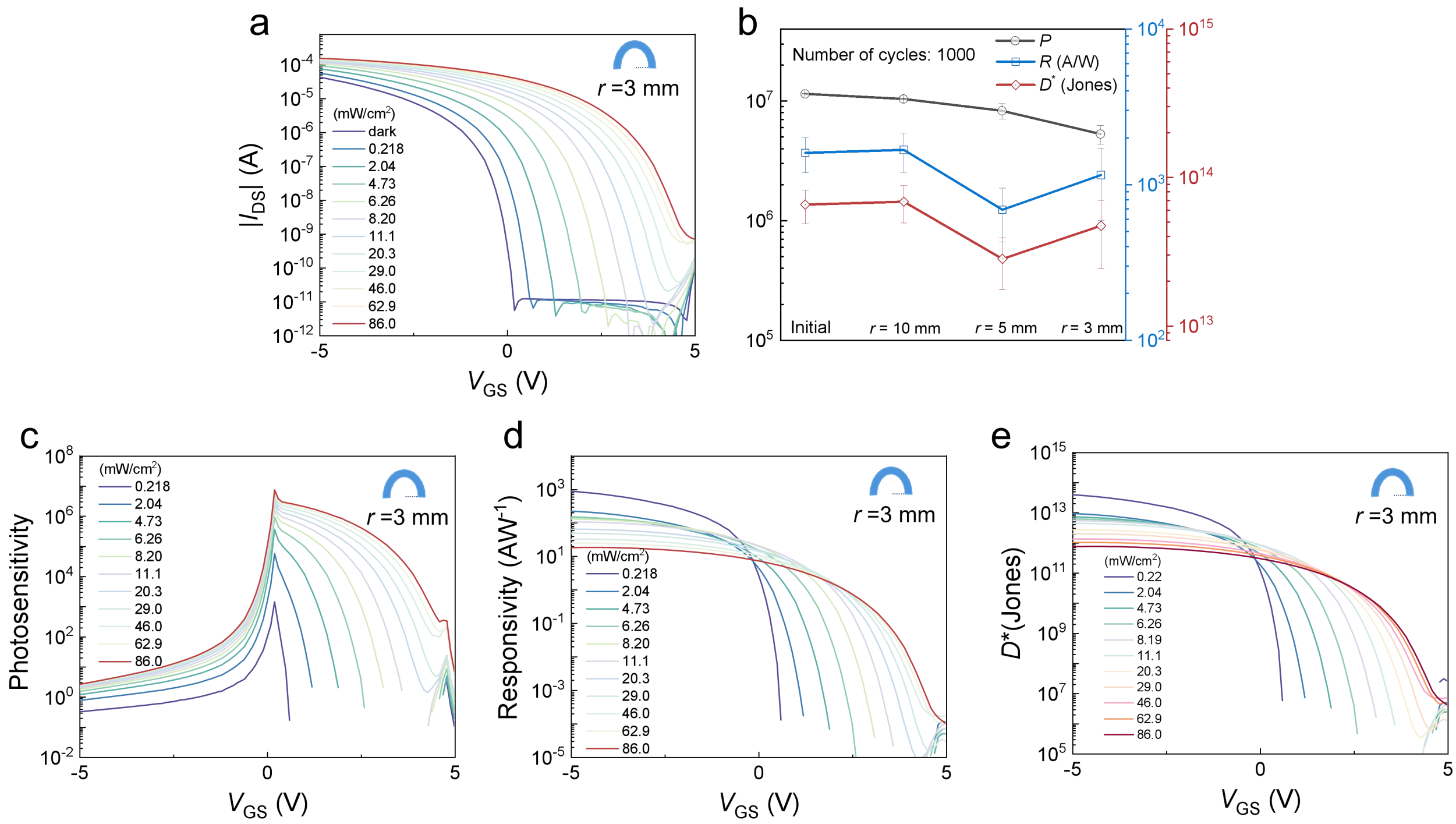

Supplementary Fig. 16 | Mechanical flexibility and optoelectronic performance of flexible OTFTs based on 30 nm Al_2O_3 / 180 nm PAA bilayer films. a, Transfer curves measured after 1,000 bending cycles at a 3-mm radius, under various optical power densities. **b**, Statistical summary of P-value, R-value, and D-value as a function of bending radius (after 1,000 cycles). **c**, P-value after 1,000 bending cycles at a 3-mm radius, under different optical power densities. **d**, R-value after 1,000 bending cycles at a 3-mm radius, under different optical power densities. **e**, *D**-value after 1,000 bending cycles at a 3-mm radius, under different optical power densities.

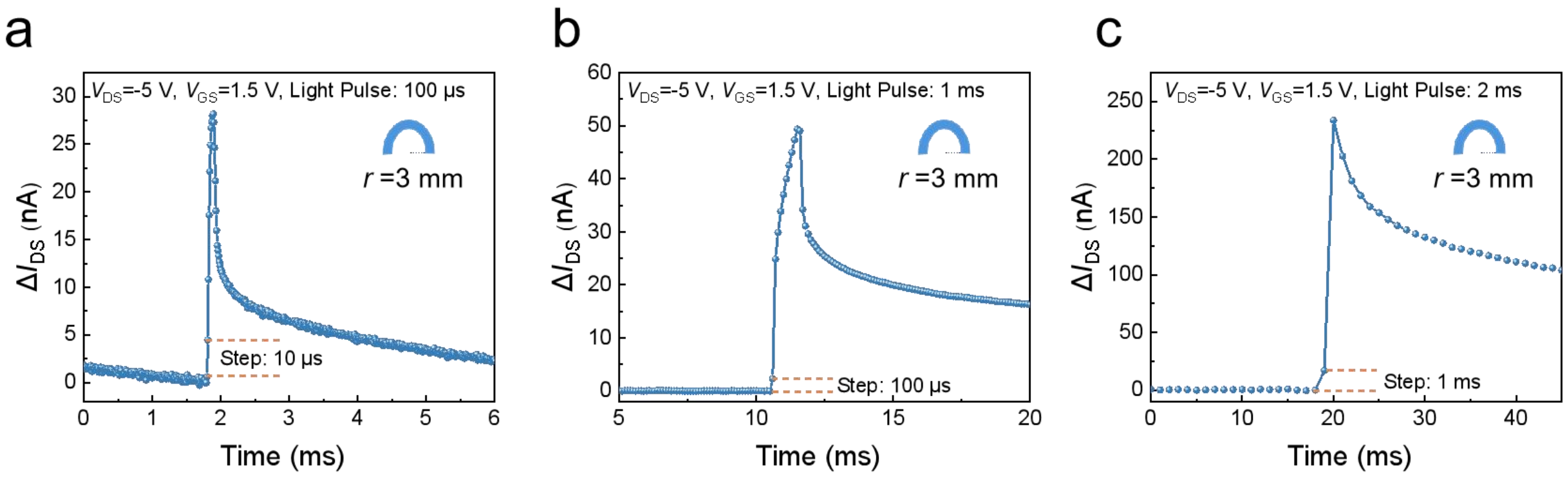

Supplementary Fig. 17 | Testing OTFTs for fast response to optical stimulation at 3mm bend radius based on 30 nm Al_2O_3/180 nm PAA films. a, 100 μs. **b**, 1 ms. **c**, 2 ms.

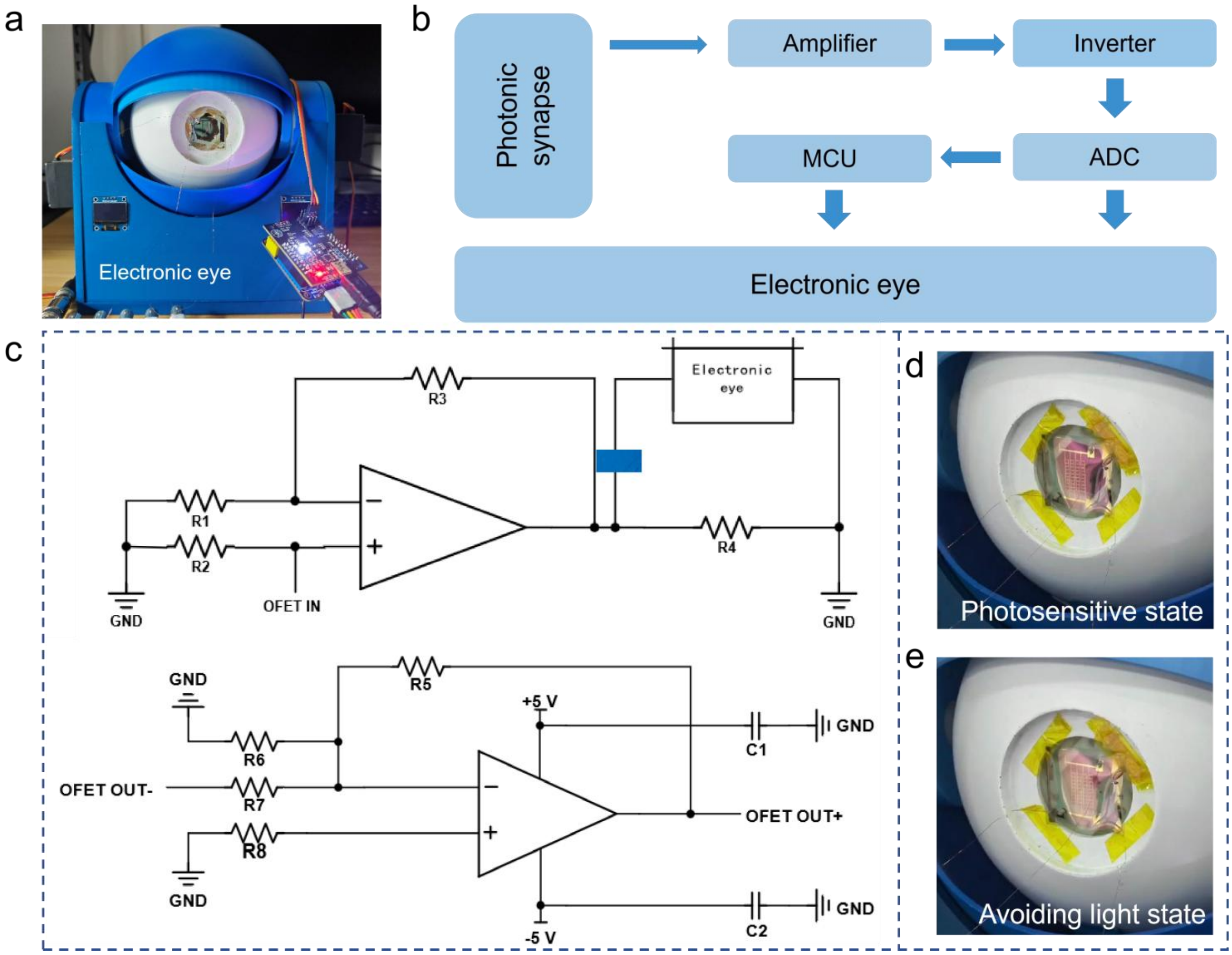

Supplementary Fig. 18 | OTFT array-driven electronic eye. a, Physical image of the device array covering the electronic eye. **b**, External circuit structure for driving the electronic eye with the device array. **c**, Schematic of the driving circuit for pupil constriction in the eye using the device array. **d**, Pupil remains dilated in a dark environment. **e**, Pupil remains constricted under bright light stimulation.

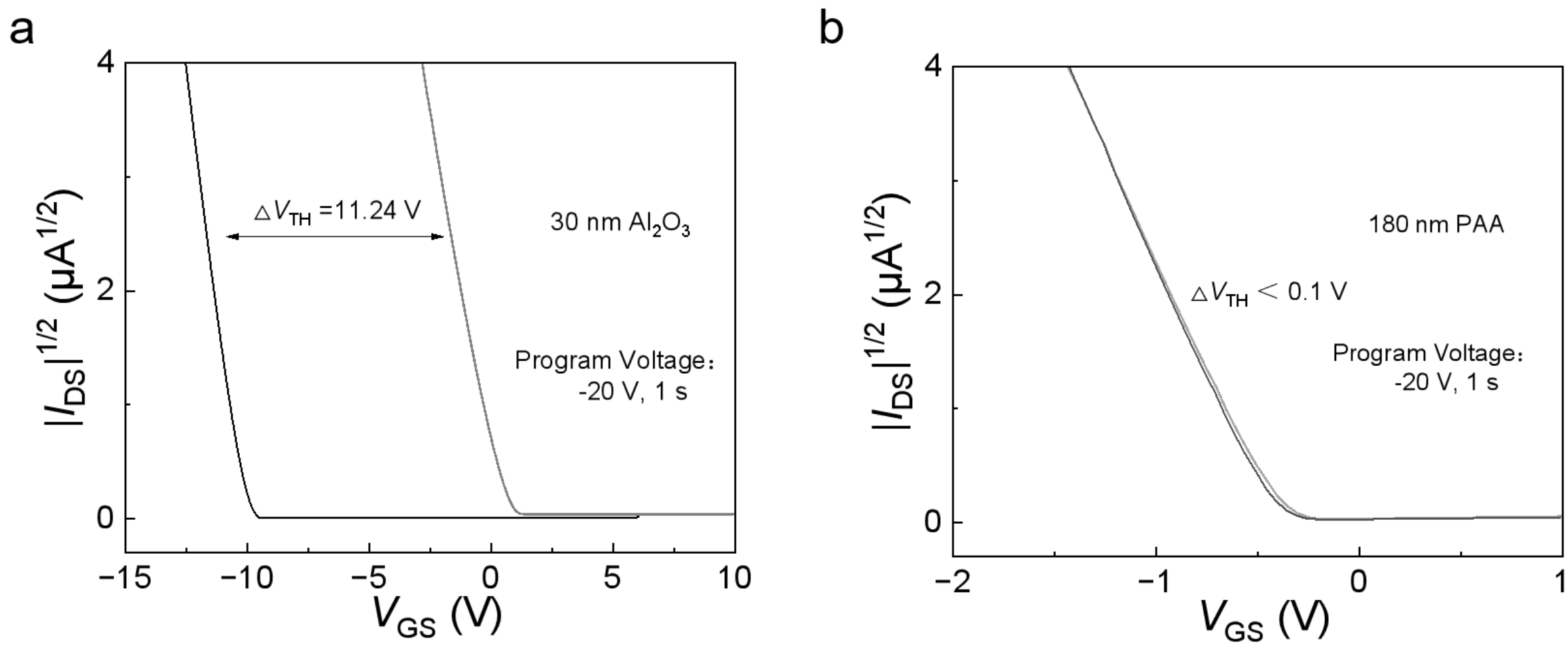

Supplementary Fig. 19 | Storage window comparison of OTFTs with single-layer dielectrics under a –20 V programming voltage (1 s pulse). a, Single Al_2O_3 dielectric. **b**, Single PAA dielectric.

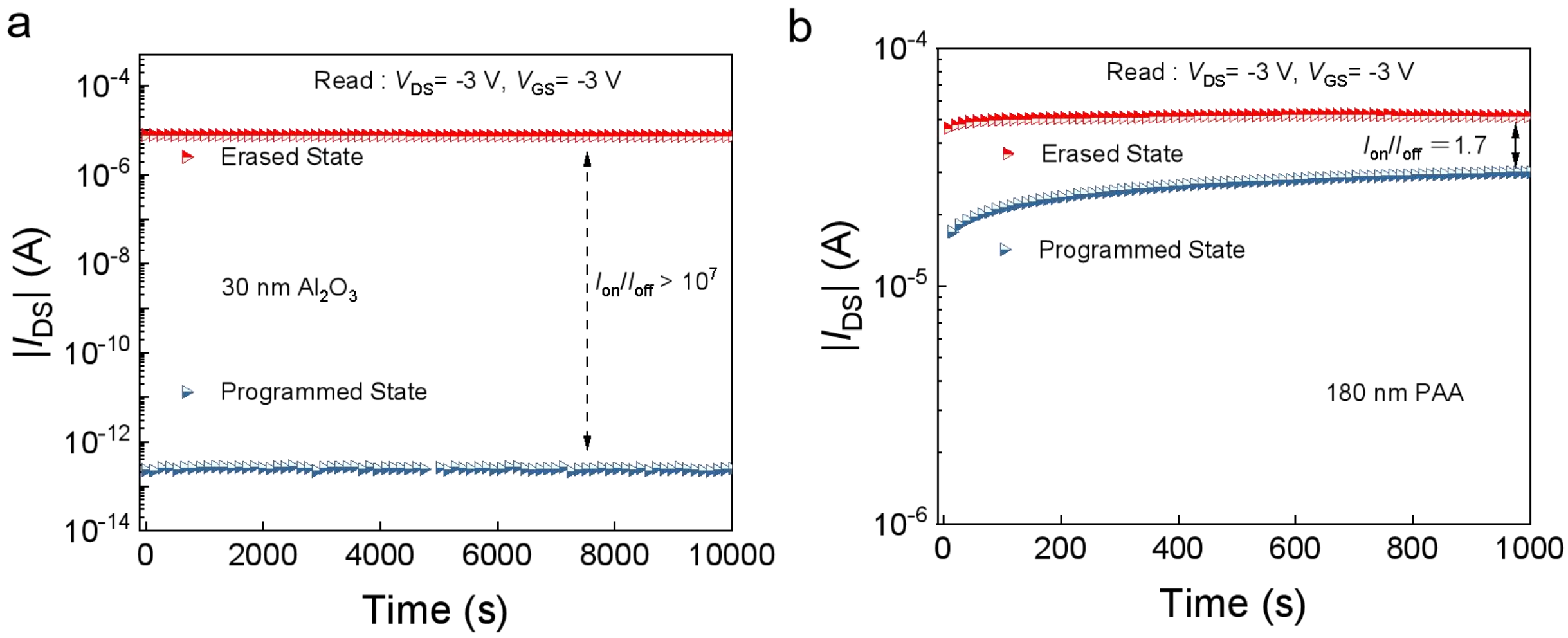

Supplementary Fig. 20 | Retention time comparison of OTFTs with single-layer dielectrics under a –20 V programming voltage (1 s pulse). a, Single Al_2O_3 dielectric. **b**, Single PAA dielectric.

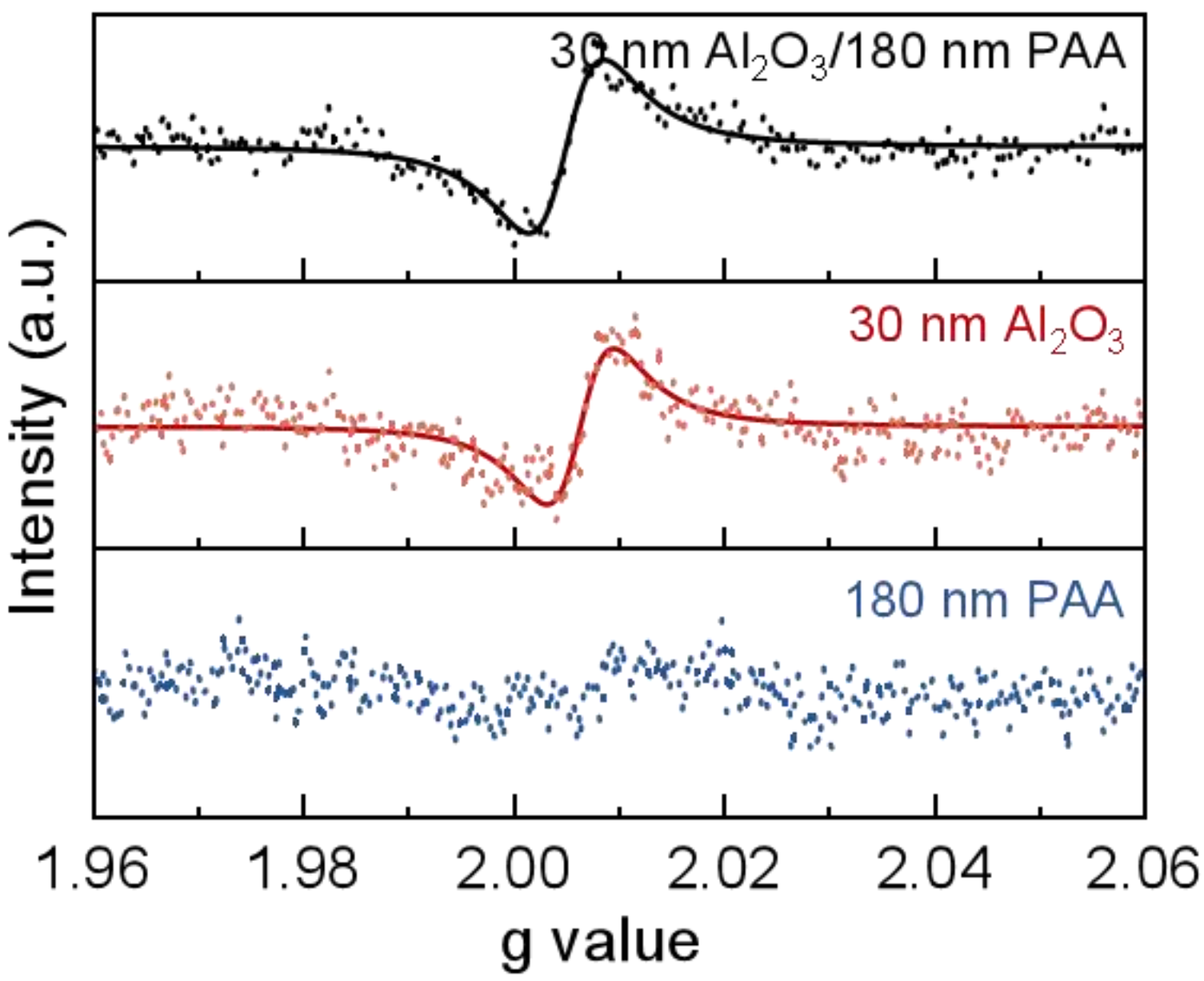

Supplementary Fig. 21 | Electron paramagnetic resonance (EPR) spectra of single PAA, single Al_2O_3 and Al_2O_3/PAA heterostructure. A clear signal at g≈2.003 is observed only for Al_2O_3-containing layers, confirming the presence of oxygen vacancies introduced by LT-PEALD Al_2O_3.

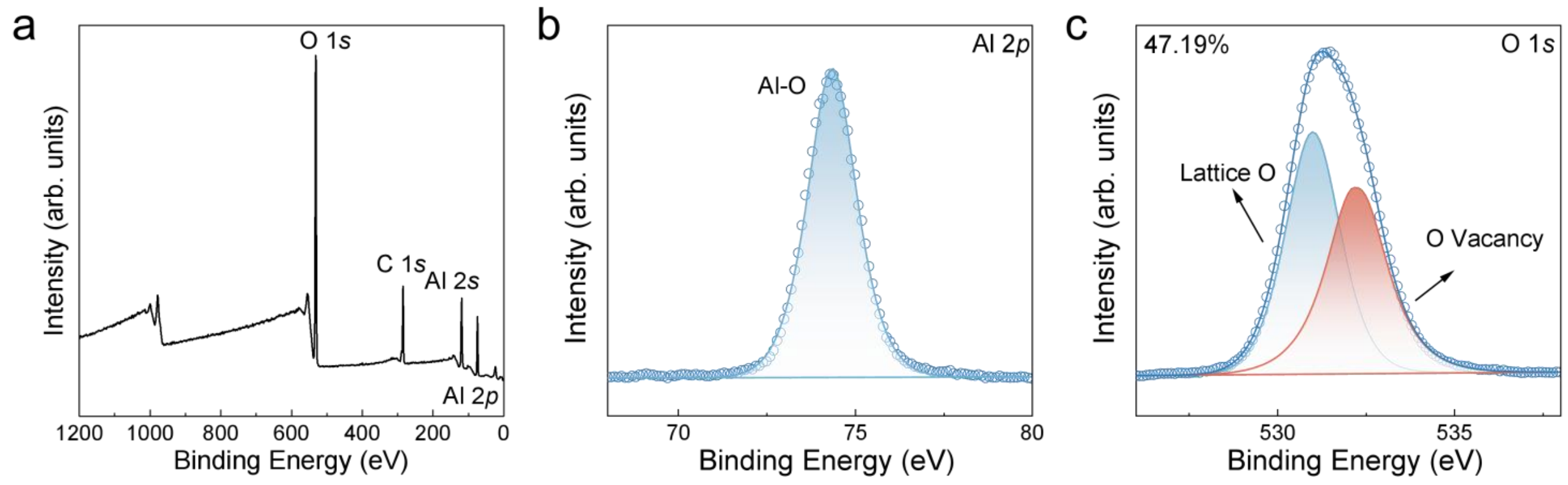

Supplementary Fig. 22 | XPS analysis of the single Al_2O_3 layer. a, Survey XPS spectrum of the Al_2O_3 layer, confirming the presence of Al, O and C elements. **b**, High-resolution Al 2*p* core-level spectrum, showing a single peak at 74.3 eV characteristic of Al–O bonding in Al_2O_3. **c**, High-resolution O 1*s* core-level spectrum with peak deconvolution. The main peak at 530.9 eV (lattice oxygen in Al–O), and a shoulder peak at approximately 531.8 eV (oxygen vacancies or defect-related oxygen species). The distinct shoulder peak at ~531.8 eV confirms the presence of oxygen vacancies in the Al_2O_3 layer, providing deep trapping sites for charge storage.

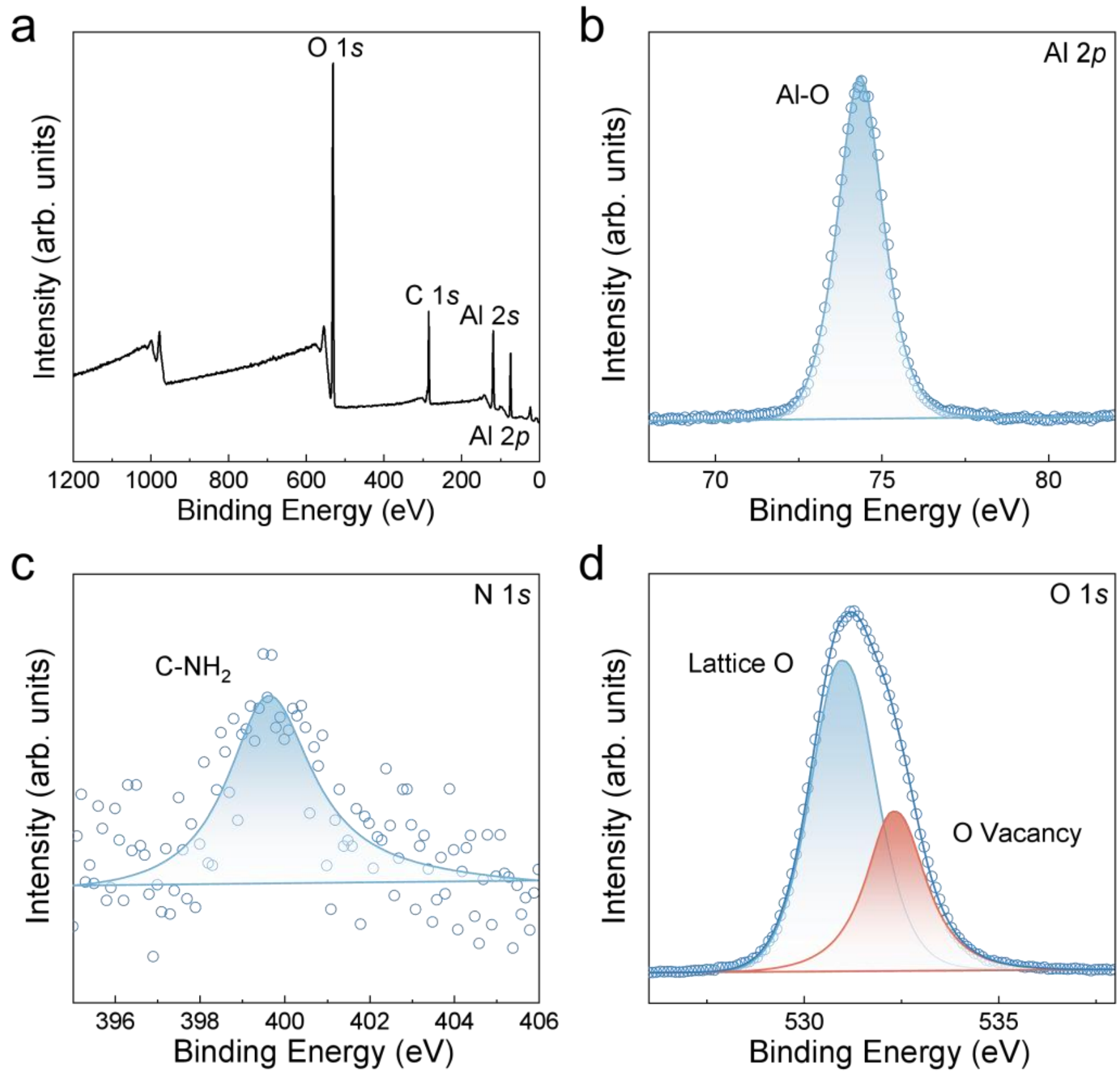

Supplementary Fig. 23 | XPS analysis of the single Al_2O_3/PAA layer. a, Survey XPS spectrum of the Al_2O_3 layer, confirming the presence of Al, O and C elements. **b**, High-resolution Al 2*p* core-level spectrum, showing a single peak at 74.3 eV characteristic of Al–O bonding in Al_2O_3. **c**, High-resolution N 1*s* core-level spectrum. A weak signal is observed due to the limited probing depth of XPS (typically 3-10 nm), which primarily samples the top Al_2O_3 layer rather than the underlying PAA. **d**, High-resolution O 1*s* core-level spectrum with peak deconvolution. The main peak at 530.9 eV (lattice oxygen in Al–O), and a shoulder peak at approximately 531.8 eV (oxygen vacancies or defect-related oxygen species).

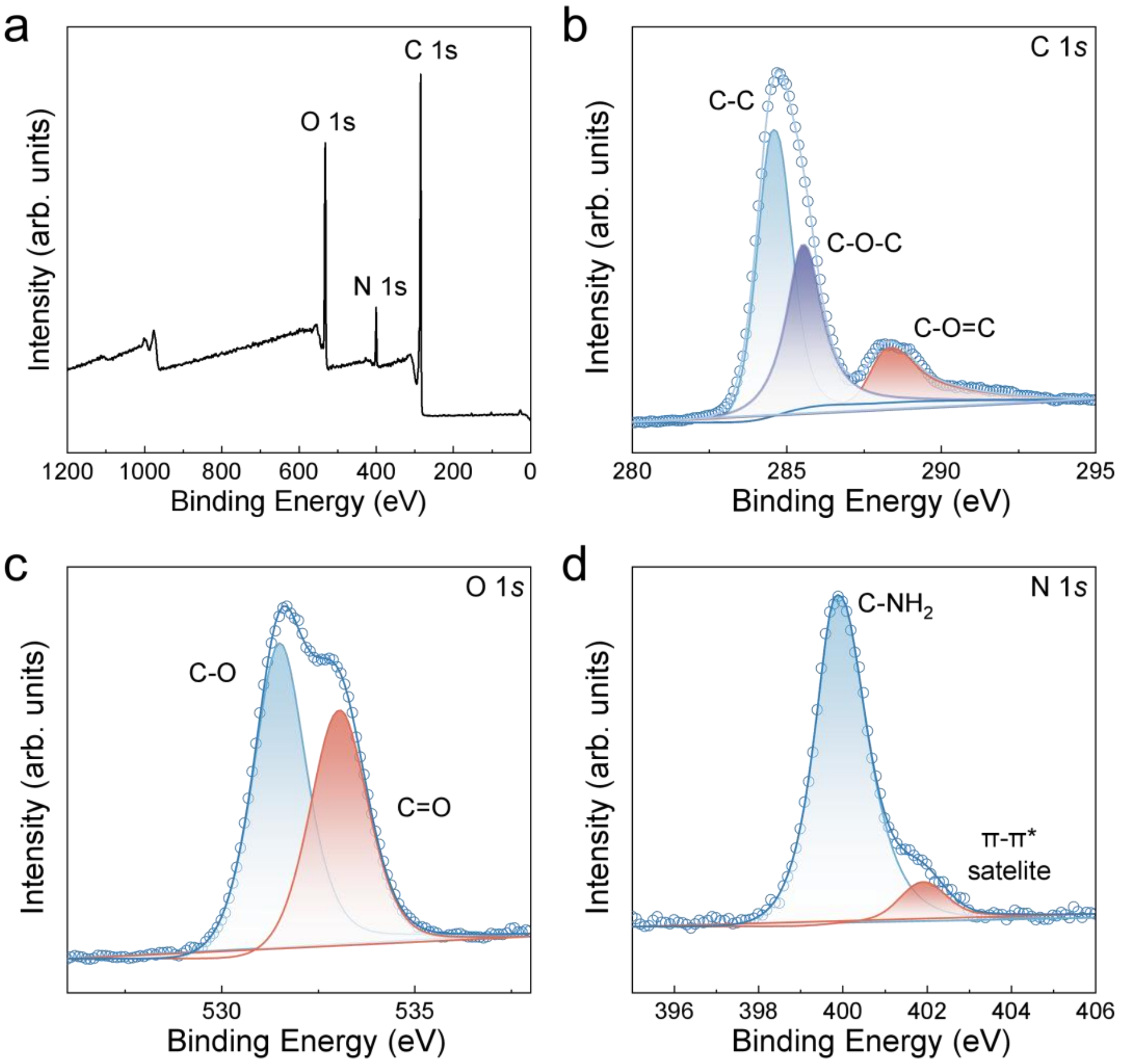

Supplementary Fig. 24 | XPS analysis of the single PAA layer. a, Survey XPS spectrum of the PAA layer, confirming the presence of C, O and N elements. **b**, High-resolution C 1*s* core-level spectrum with peak deconvolution. The C 1*s* spectrum can be fitted to three components at 284.8 eV (C–C/C–H), 286.0 eV (C–O), and 288.6 eV (O–C=O), characteristic of the poly (amic acid) structure. **c**, High-resolution O 1*s* core-level spectrum, fitted to two components at 531.5 eV (C–O) and 533.0 eV (C=O), consistent with the carboxyl and carbonyl groups in PAA. **d**, High-resolution N 1*s* core-level spectrum, showing a peak at approximately 400.1 eV corresponding to the amide groups (–NH–) in PAA. No signal at ~531.8 eV (oxygen vacancies) is observed in the O 1*s* spectrum, consistent with the EPR result confirming the absence of oxygen vacancies in the single PAA layer.

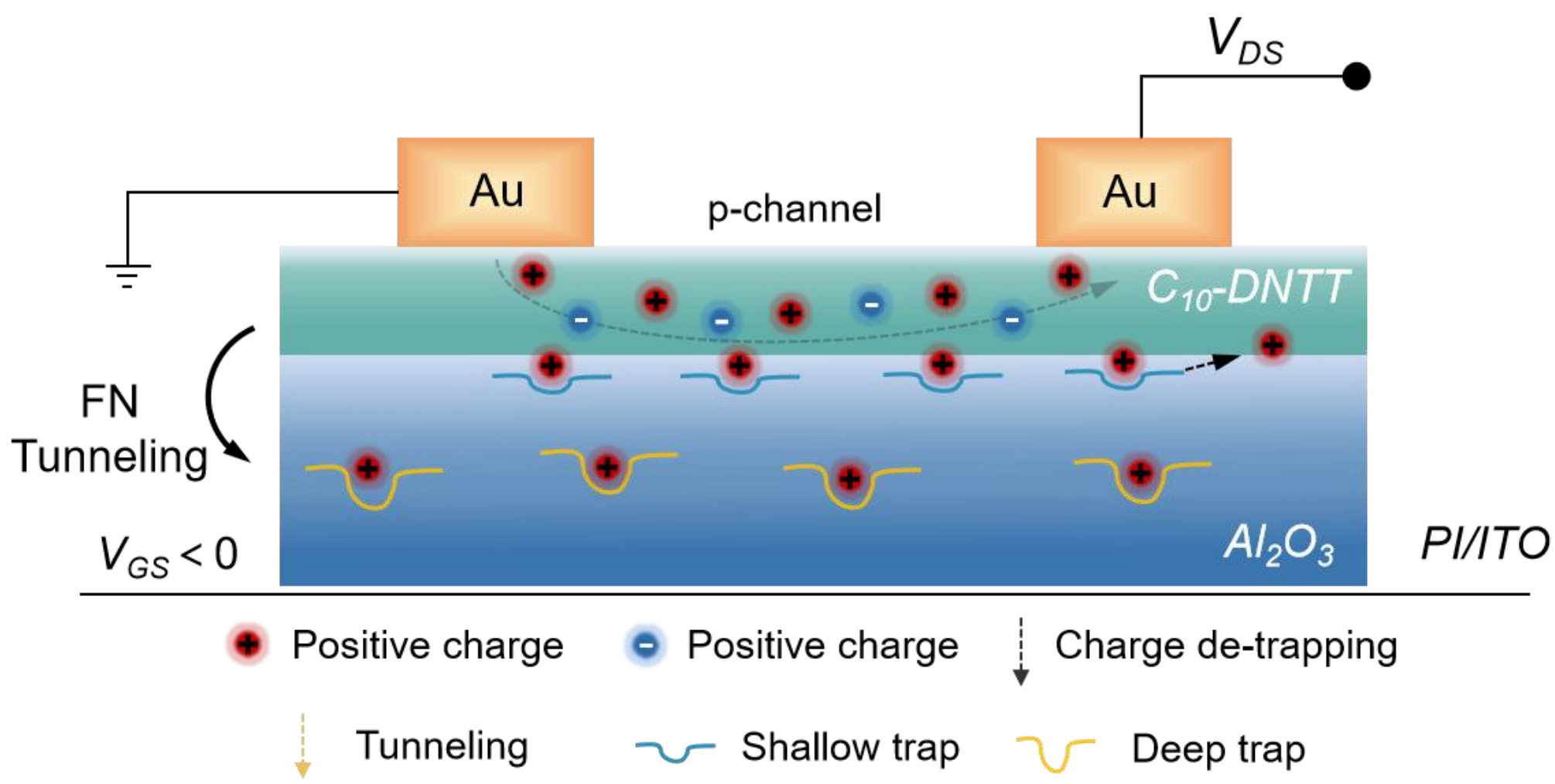

Supplementary Fig. 25 | Schematic of OTFT storage mechanism when single Al_2O_3 is used as dielectric layer. The diagram depicts the process of hole trapping in deep-level defects of the Al_2O_3 layer upon application of a negative gate voltage pulse.

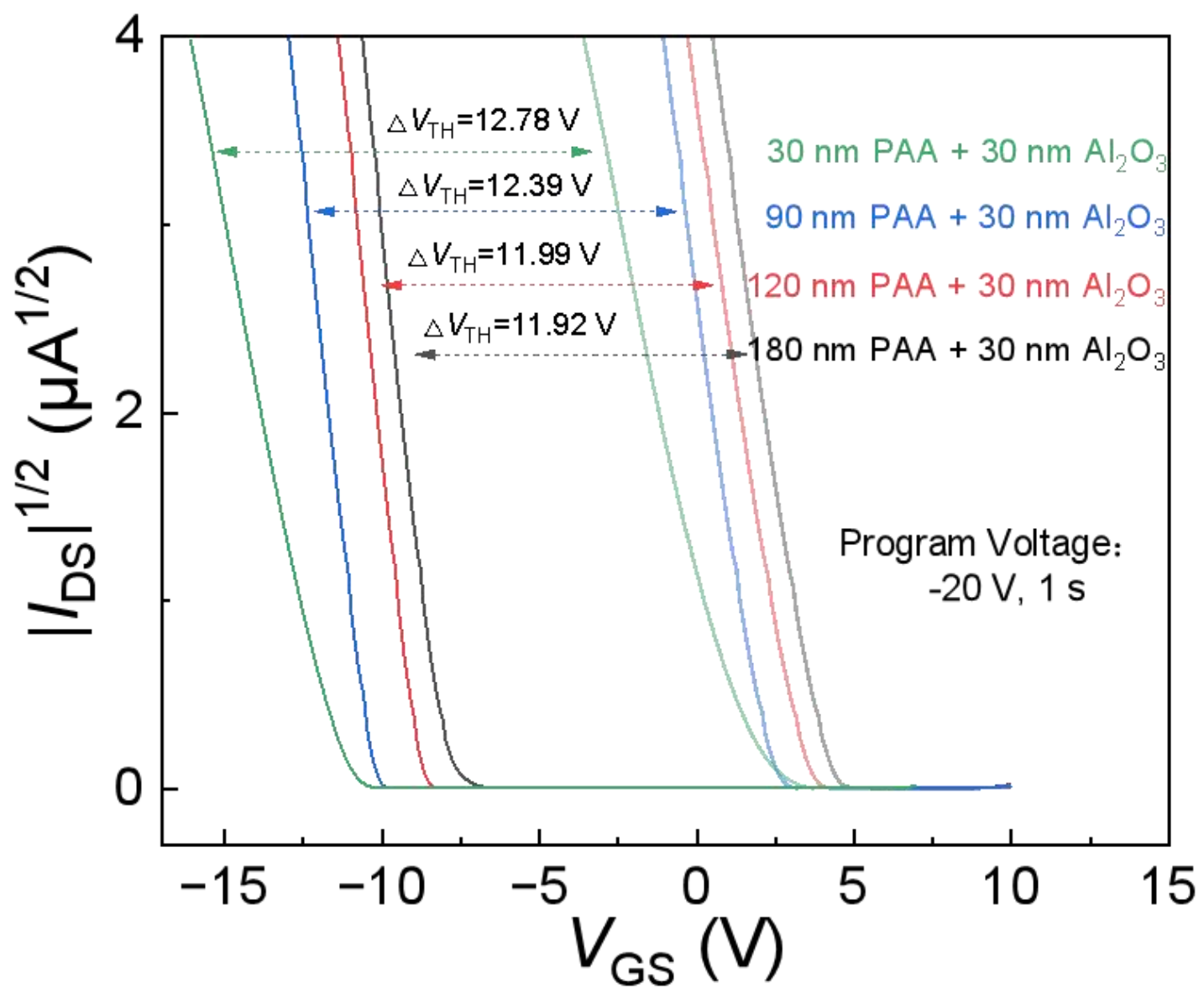

Supplementary Fig. 26 | Storage window comparison in PAA/Al_2O_3 heterostructure-based OTFTs under –20 V programming voltage (1 s pulse) with varied PAA thicknesses. The obtained storage windows are: 12.78 V for 30 nm PAA / 30 nm Al_2O_3, 12.39 V for 90 nm PAA / 30 nm Al_2O_3, 11.99 V for 120 nm PAA / 30 nm Al_2O_3, and 11.92 V for 180 nm PAA / 30 nm Al_2O_3.

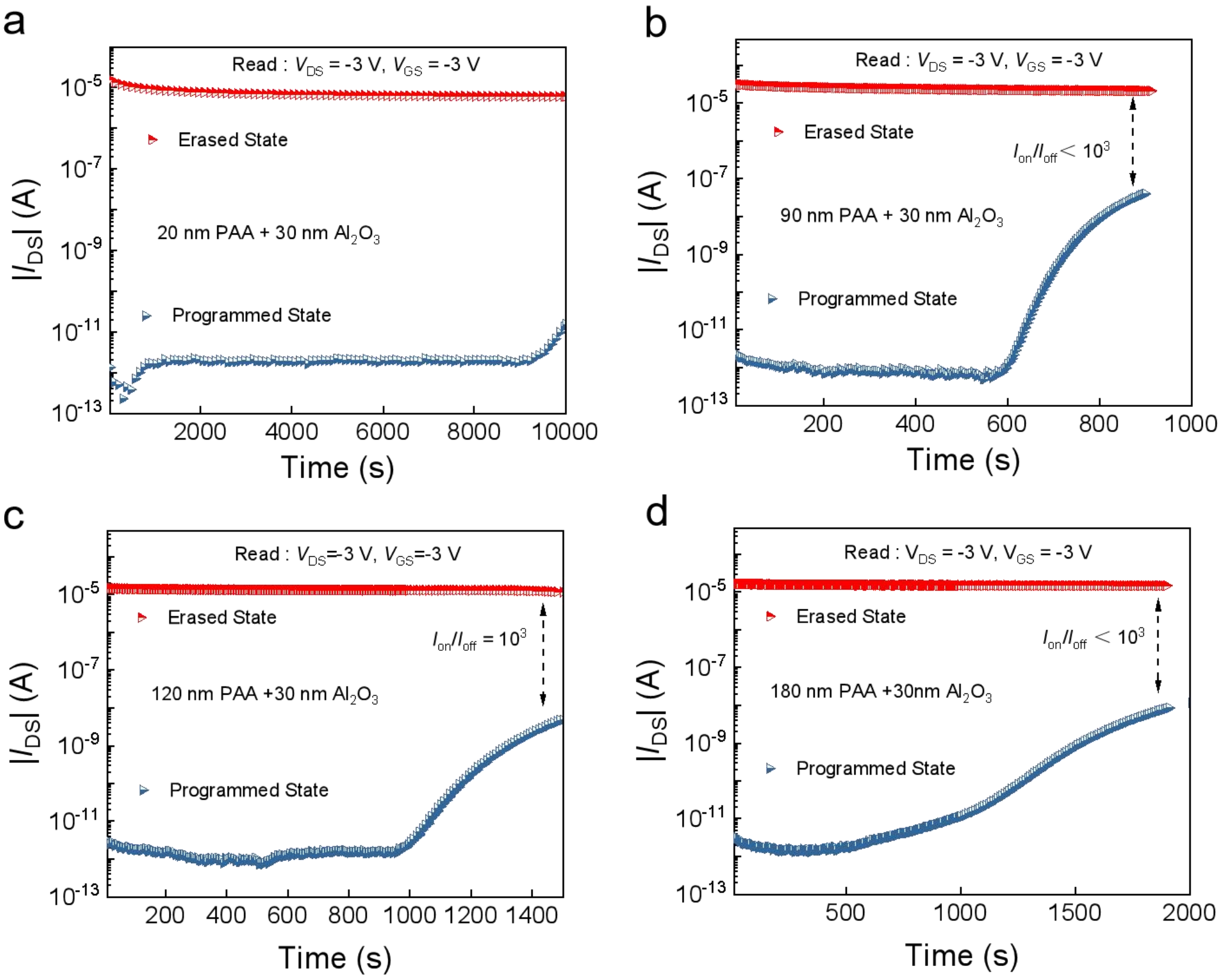

Supplementary Fig. 27 | Retention time comparison of PAA/Al_2O_3 heterostructure-based OTFTs with varied PAA thicknesses, measured under a –20 V programming voltage (1 s pulse). a, 20nm PAA + 30nm Al_2O_3. **b**, 90nm PAA + 30nm Al_2O_3. **c**, 120nm PAA + 30nm Al_2O_3. **d**, 180nm PAA + 30nm Al_2O_3

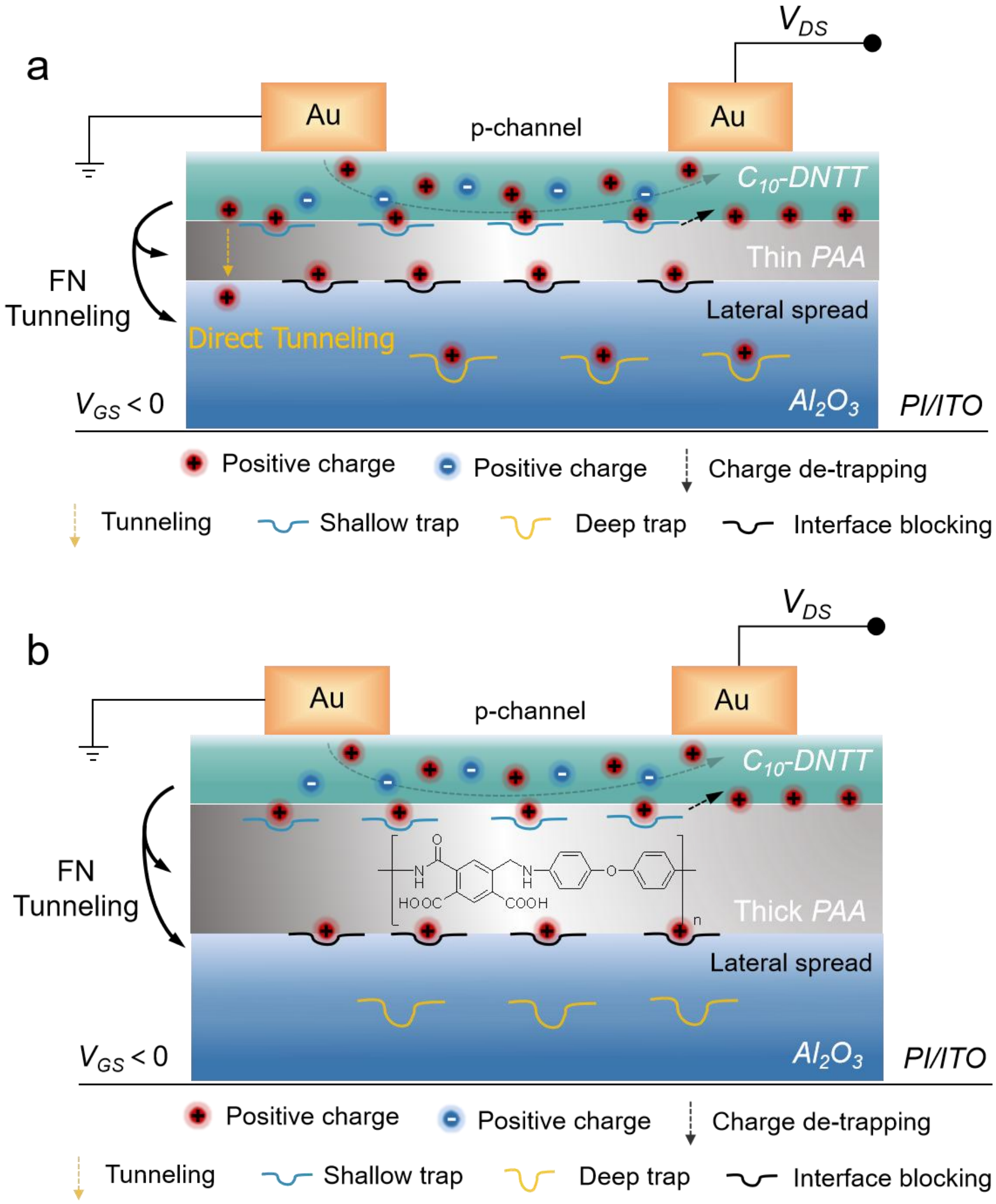

Supplementary Fig. 28 | Schematic illustration of charge storage mechanisms in PAA/Al_2O_3 heterostructure-based OTFTs with different PAA thicknesses. **a**, Thin PAA configuration. **b**, Thick PAA configuration.

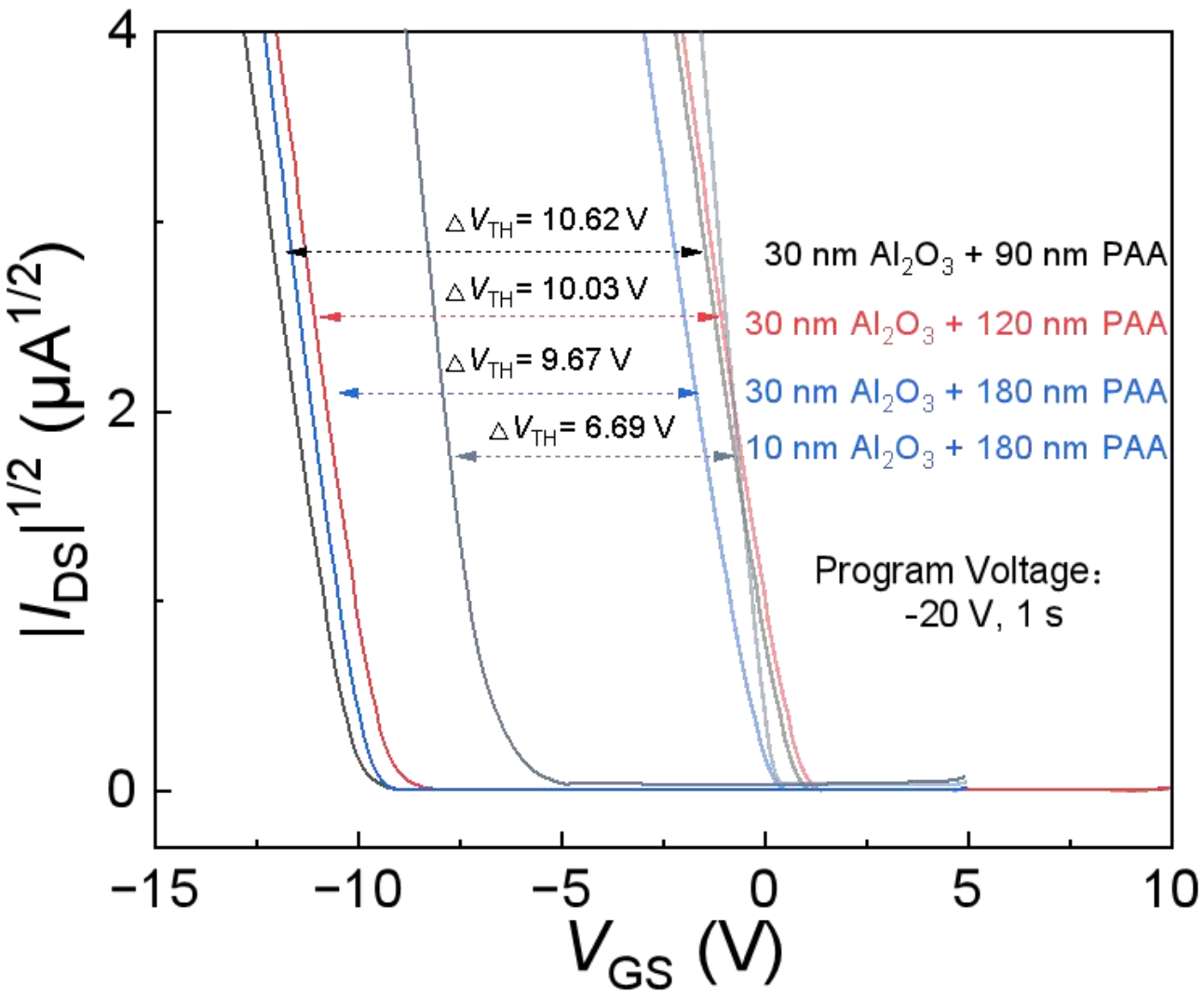

Supplementary Fig. 29 | Storage window comparison in Al_2O_3/PAA heterostructure-based OTFTs under a programming voltage of –20 V (1 s pulse) with varied Al_2O_3 and PAA thicknesses. The obtained storage windows are: 10.62 V for 30 nm Al_2O_3 / 90 nm PAA, 10.03 V for 30 nm Al_2O_3 / 120 nm PAA, 9.67 V for 30 nm Al_2O_3 / 180 nm PAA, and 6.69 V for 10 nm Al_2O_3 / 180 nm PAA.

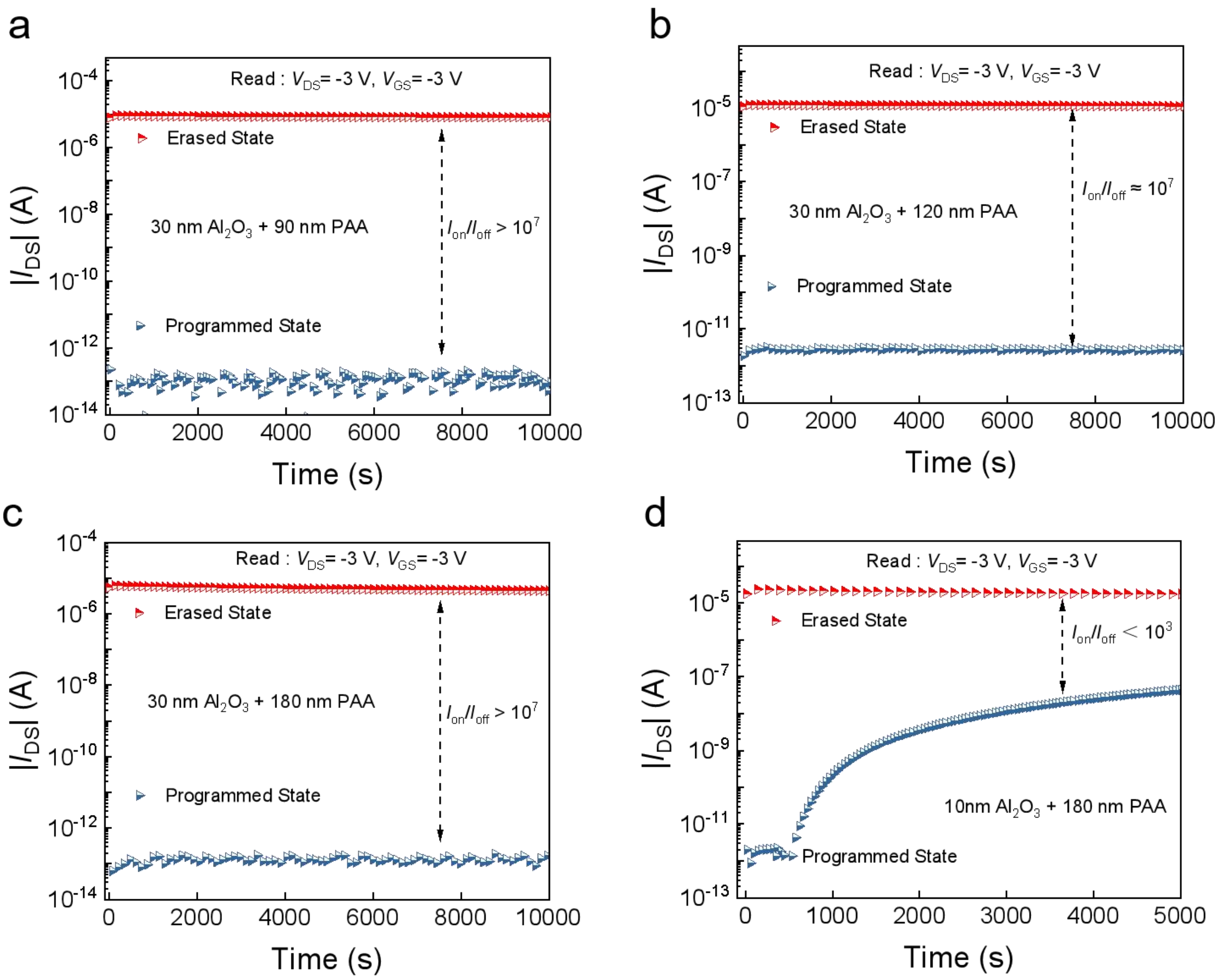

Supplementary Fig. 30 | Comparison of retention time in Al_2O_3/PAA heterostructure-based OTFTs under a –20 V programming voltage (1 s pulse) with various Al_2O_3 and PAA thicknesses. a, 30 nm Al_2O_3 / 90 nm PAA. **b,** 30 nm Al_2O_3 / 120 nm PAA. **c,** 30 nm Al_2O_3 / 180 nm PAA. **d,** 10 nm Al_2O_3 / 120 nm PAA.

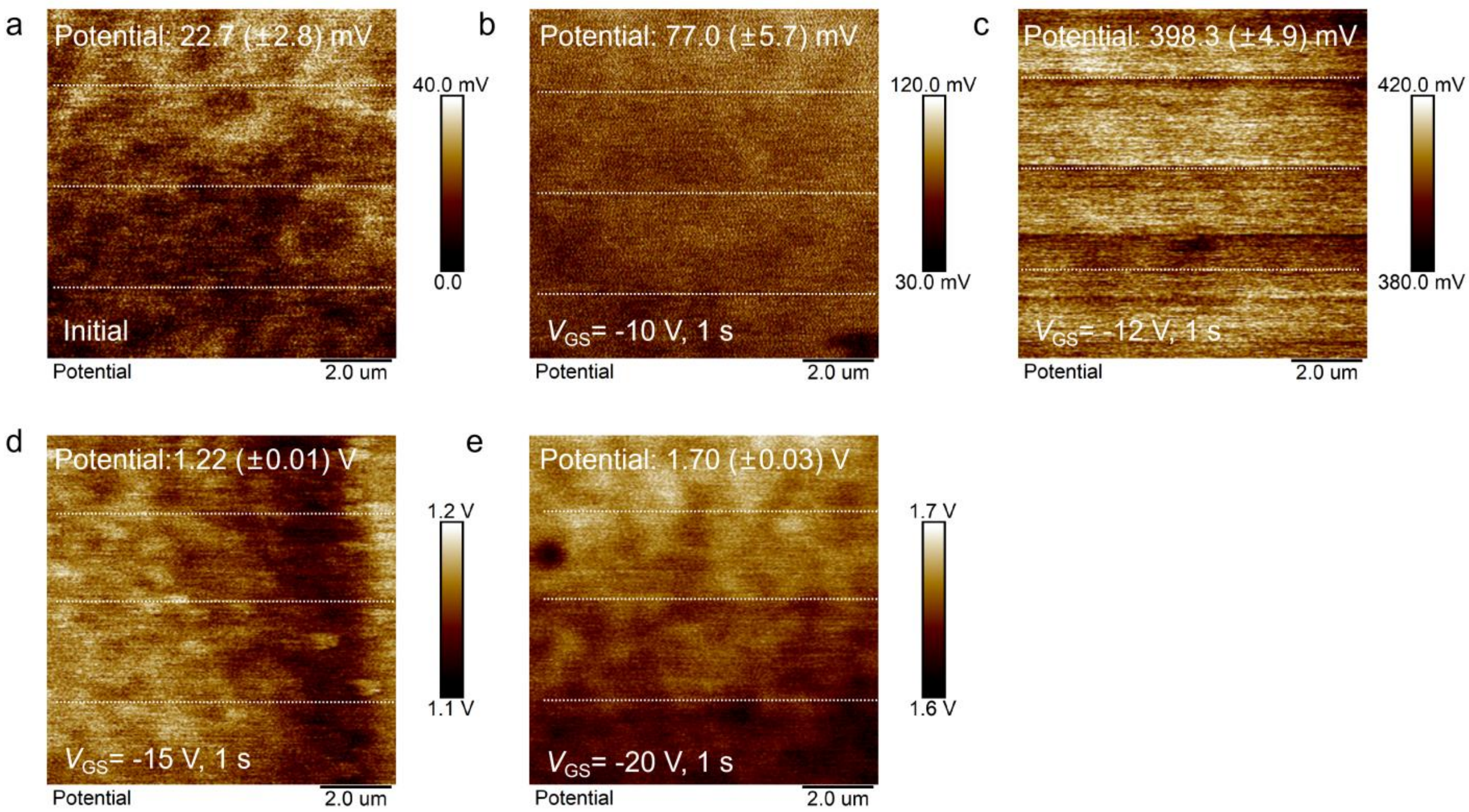

Supplementary Fig. 31 | Surface potential mapping of the transistor channel for a device with a 30 nm Al_2O_3 dielectric layer under different gate pulse conditions. a, KPFM surface potential image of the semiconductor channel in the pristine state (without gate voltage application). **b–e**, KPFM surface potential images after applying gate pulses of –10 V (**b**), –12 V (**c**), –15 V (**d**), and –20 V (**e**) for 1 s each. The pulse was removed prior to KPFM scanning.

The KPFM measurements were carried out using a conductive tip with a work function of ϕ_{tip}=5.1 eV (calibrated against HOPG). The contact potential difference V_{CPD} is given by:

$$V_{CPD}=\frac{\phi_{sample}-\phi_{tip}}{e} \quad (3)$$

Where ϕ_{sample} is the work function of the semiconductor surface. The Fermi level of the sample is $E_{F,sample}$ = -ϕ_{sample}. Hence:

$$E_{F,sample}=-5.1\,eV-eV_{CPD} \quad (4)$$

For a p-type semiconductor (C10-DNTT), the hole concentration is

$$p=N_v exp(\frac{E_V-E_F}{k_B T}) \quad (5)$$

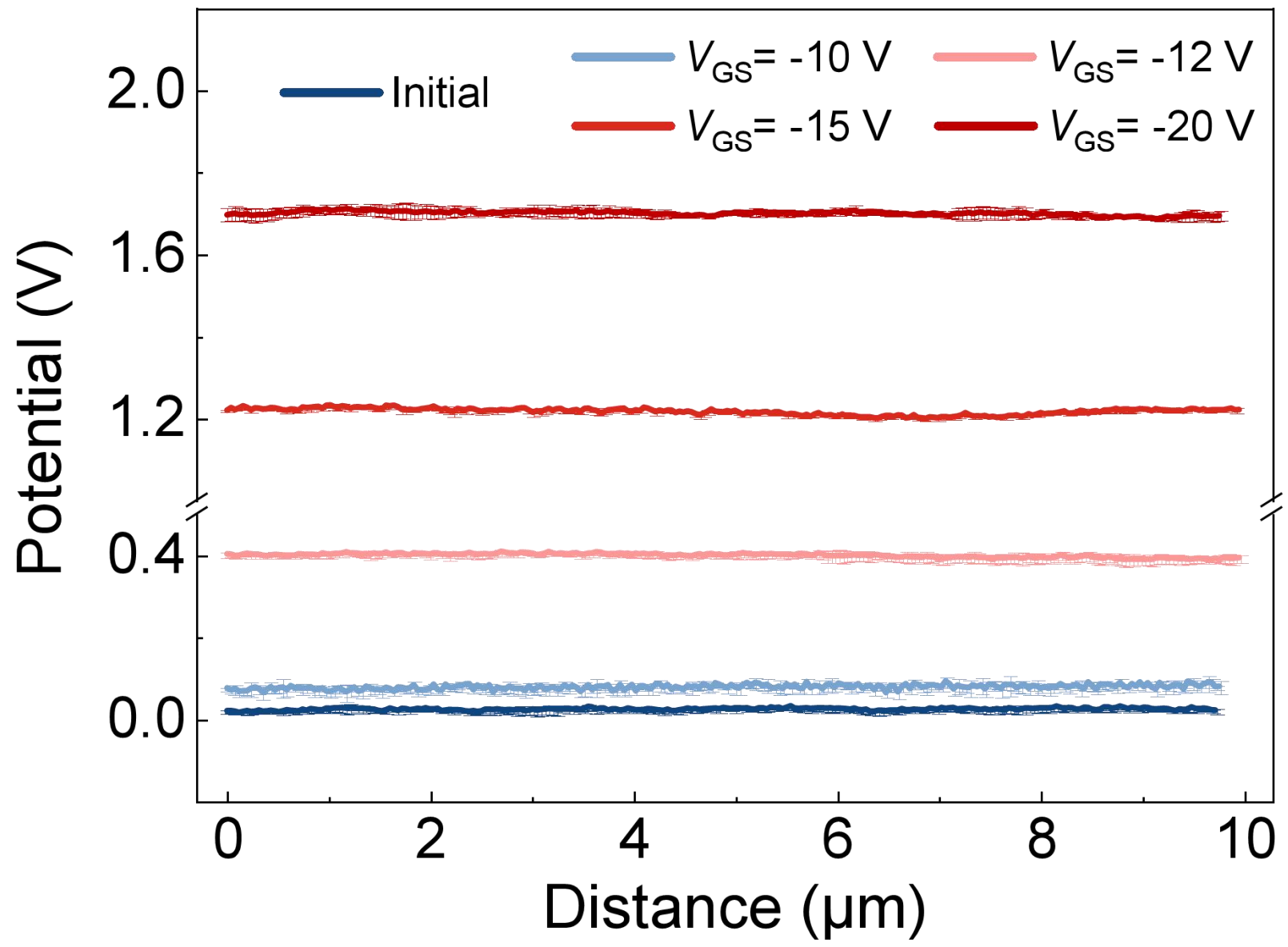

Supplementary Fig. 32 | Statistical summary of the average surface potential for transistors with an Al_2O_3 dielectric layer under various gate pre-bias conditions. The data points represent the mean surface potential values extracted from three distinct measurement positions for each condition; error bars denote the standard deviation.

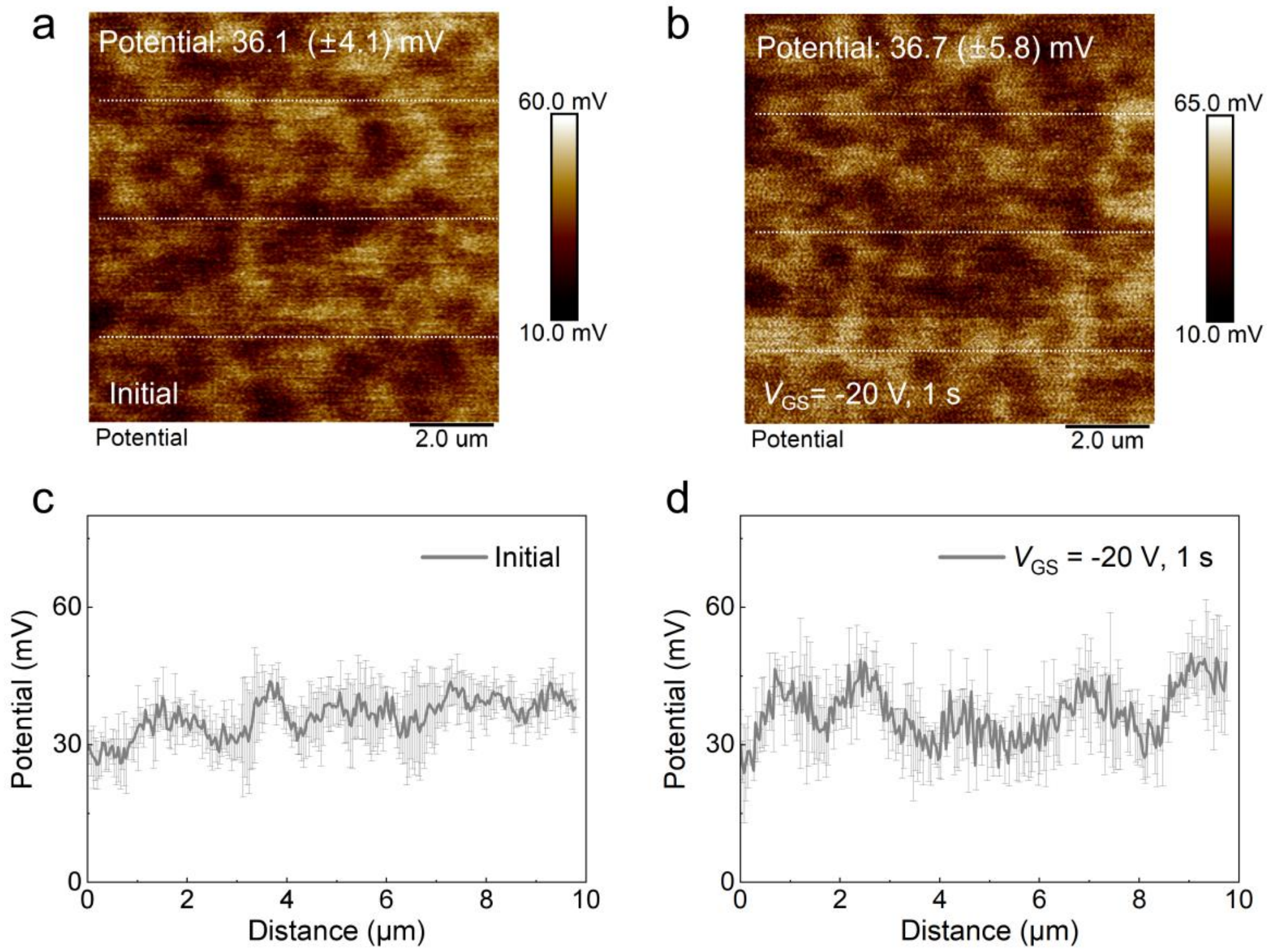

Supplementary Fig. 33 | Surface potential characteristics of transistors based on a 180 nm PAA dielectric layer before and after gate voltage pulsing. a, KPFM surface potential mapping of the semiconductor channel before gate voltage application. **b**, KPFM surface potential mapping after applying a –20 V, 1 s gate pulse. **c**, Statistical distribution of the initial surface potential (mean value: 36.1 ± 4.1 mV). **d**, Statistical distribution of the surface potential after pulsing (mean value: 36.7 ± 5.8 mV). The negligible change confirms that the single PAA layer exhibits very weak charge trapping capability.

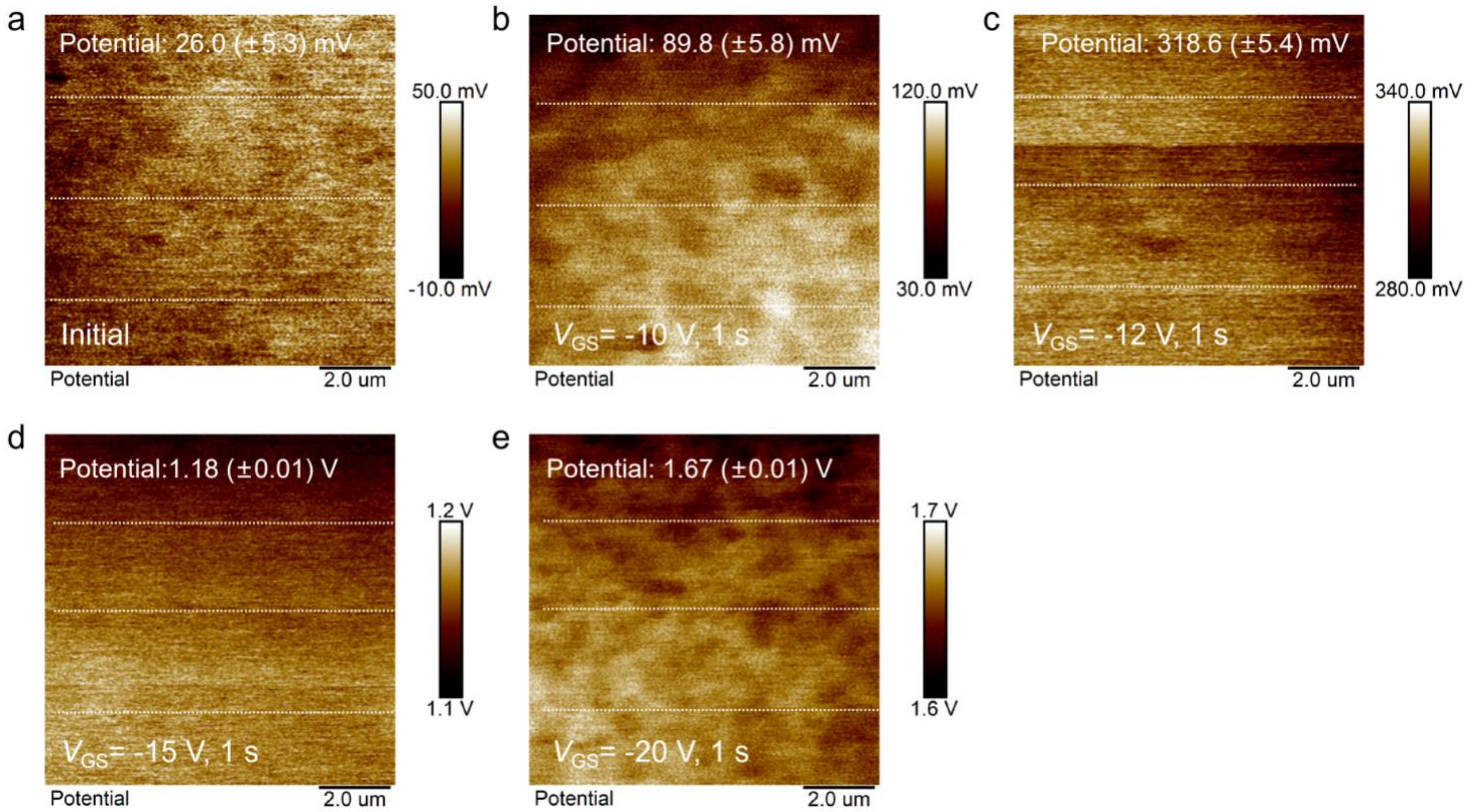

Supplementary Fig. 34 | Surface potential mapping of the transistor channel for a device with a 30 nm Al_2O_3/180 nm PAA dielectric layer under different gate pulse conditions. a, KPFM surface potential image of the semiconductor channel in the pristine state (without gate voltage application). **b–e**, KPFM surface potential images after applying gate pulses of –10 V (**b**), –12 V (**c**), –15 V (**d**), and –20 V (**e**) for 1 s each. The pulse was removed prior to KPFM scanning.

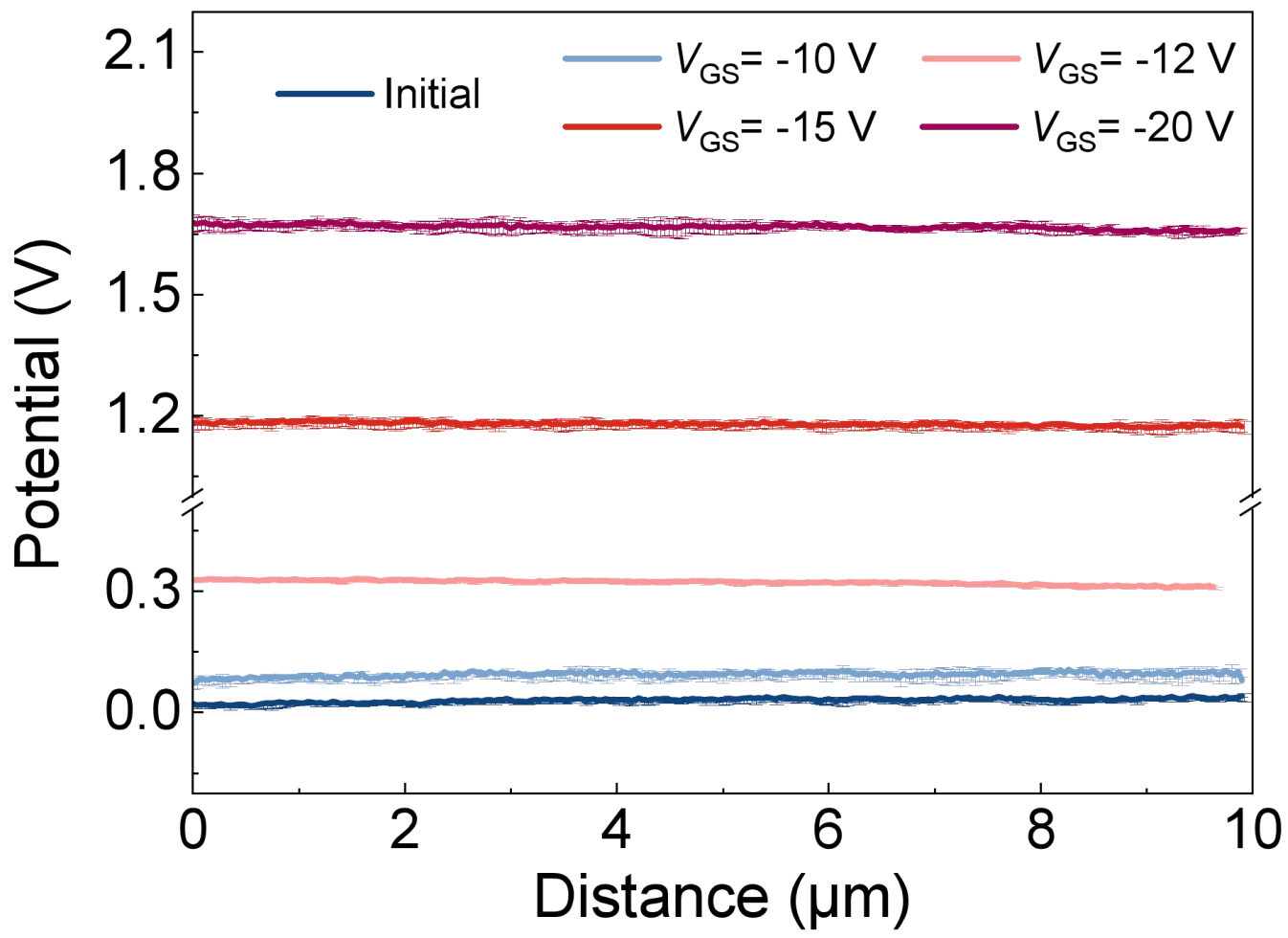

Supplementary Fig. 35 | Statistical summary of the average surface potential for transistors with a 30 nm Al_2O_3/180 nm PAA dielectric layer under various gate pre-bias conditions. The data points represent the mean surface potential values extracted from three distinct measurement positions for each condition; error bars denote the standard deviation.

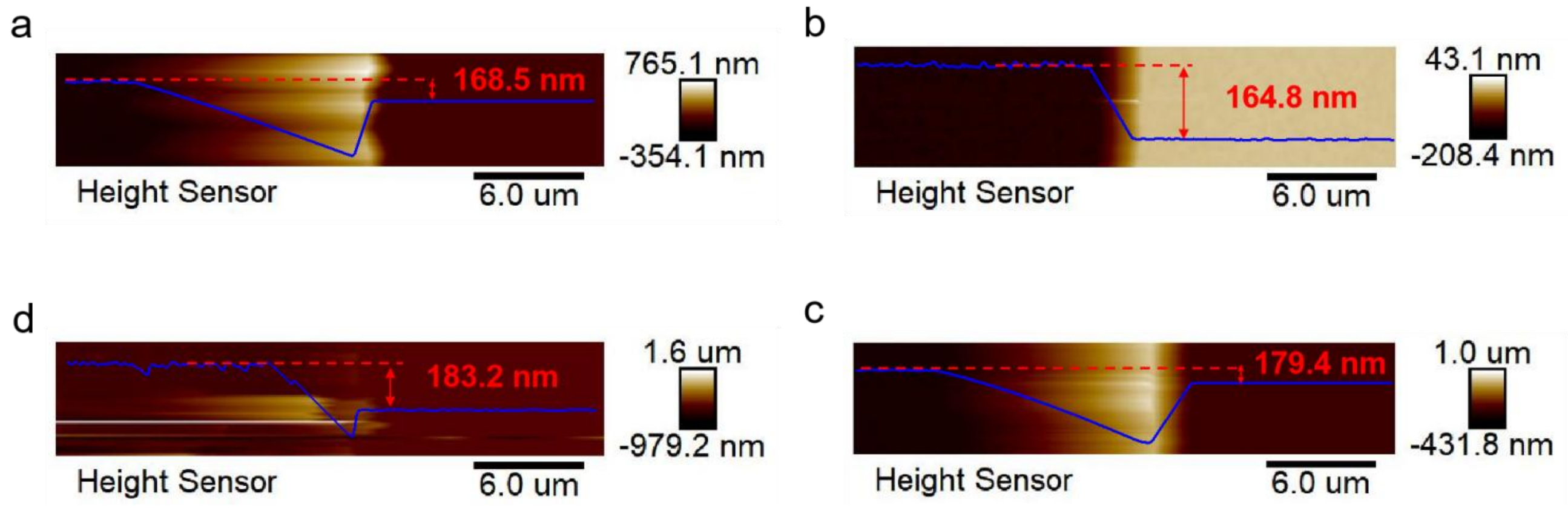

Supplementary Fig. 36 | AFM-based thickness measurement of polymer dielectric films. a, PAN. **b**, PS. **c**, PVP. **d**, PPO.

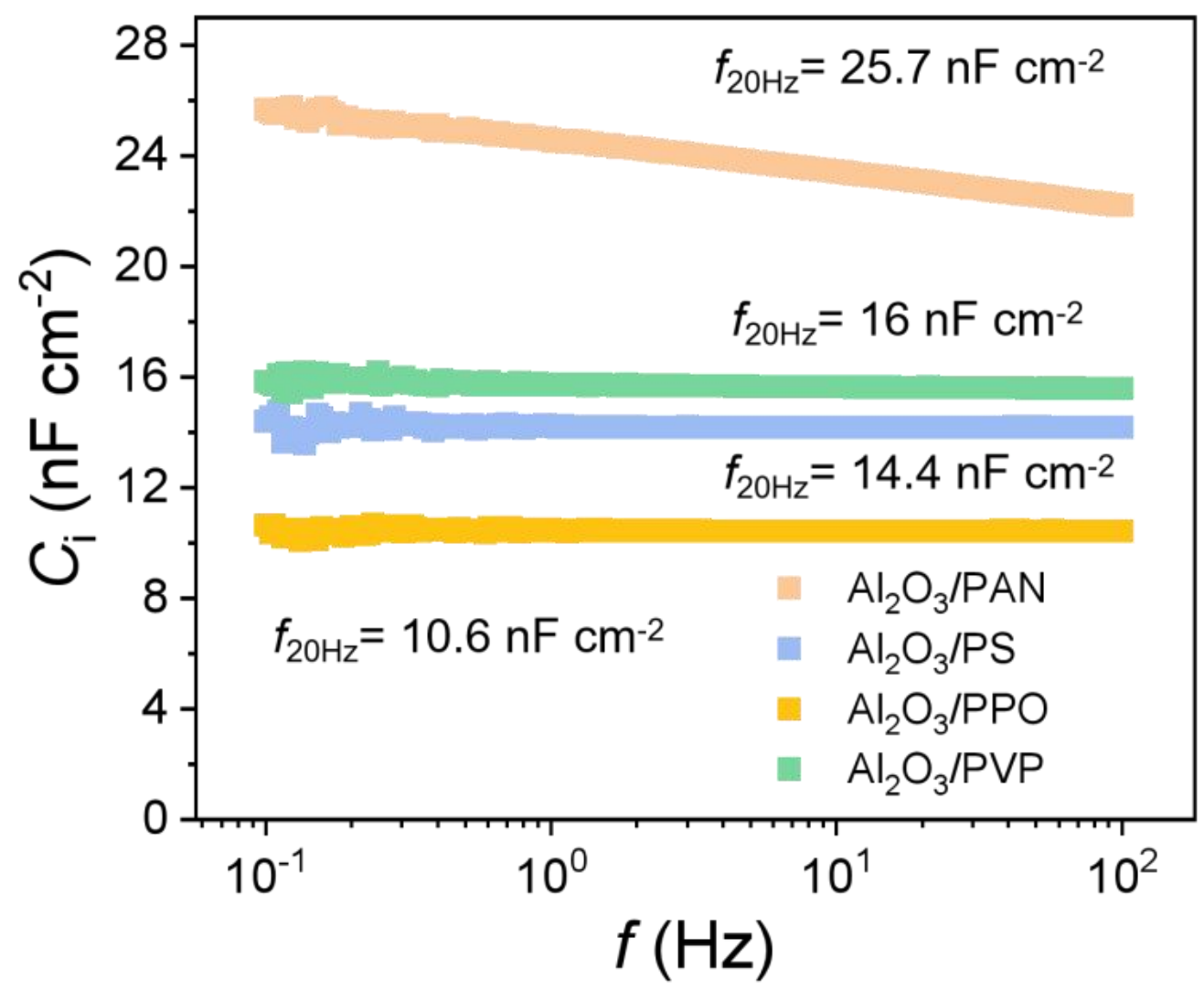

Supplementary Fig. 37 | Capacitance characterisation of Al_2O_3/polymer heterostructure dielectrics. At 20 Hz, the capacitances of the four heterostructure dielectrics were Al_2O_3/PAN (25.7 nF cm^{-2}), Al_2O_3/PS (14.4 nF cm^{-2}), Al_2O_3/PPO (10.6 nF cm^{-2}), and Al_2O_3/PVP (16.0 nF cm^{-2}).

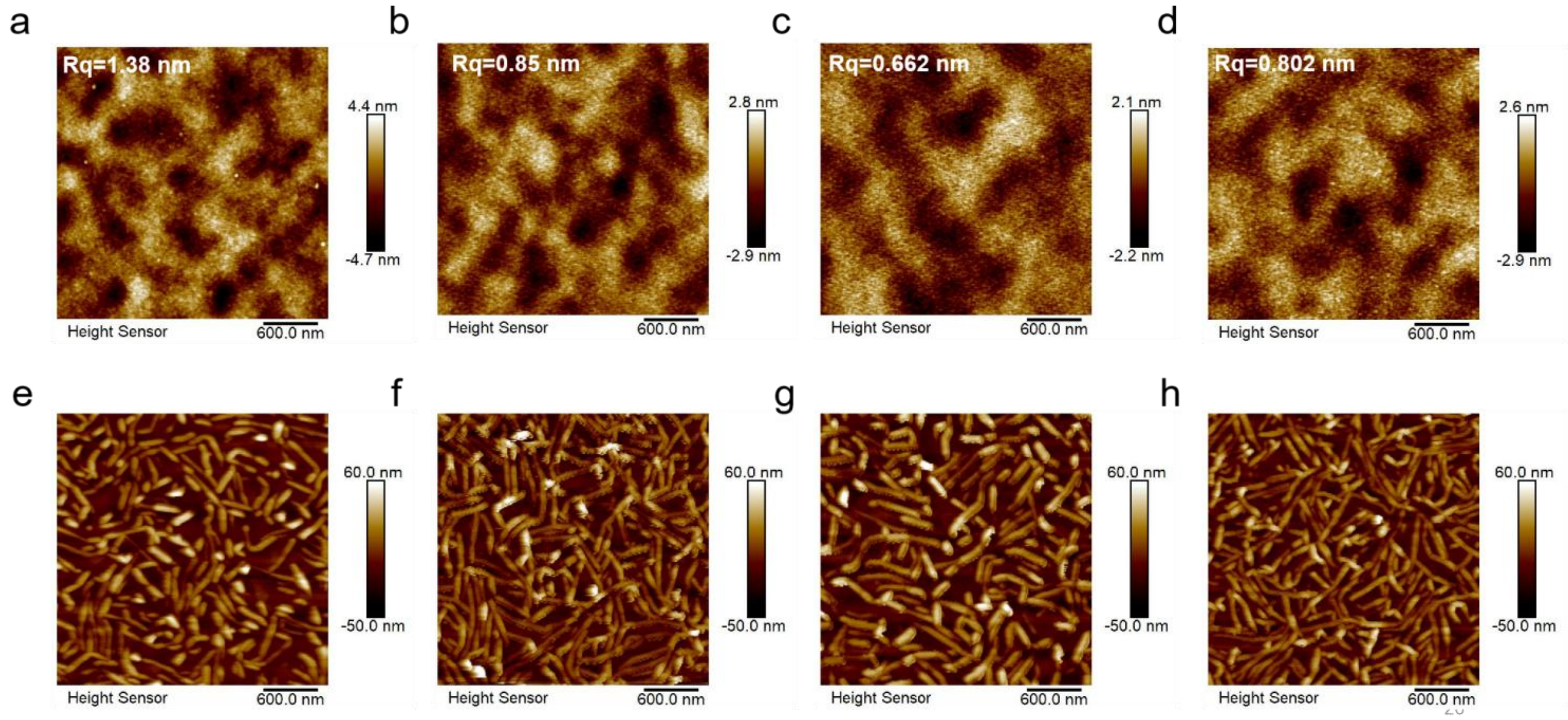

Supplementary Fig. 38 | Surface morphologies of Al_2O_3/polymer heterostructure dielectrics and the corresponding C10-DNTT semiconductor layers. a, Al_2O_3/PAN. b, Al_2O_3/PS. **c**, Al_2O_3/PVP. **d**, Al_2O_3/PPO. **e**, Al_2O_3/PAN/C10-DNTT. **f**, Al_2O_3/PS/C10-DNTT. **g**, Al_2O_3/PVP/C10-DNTT. **h**, Al_2O_3/PPO/C10-DNTT.

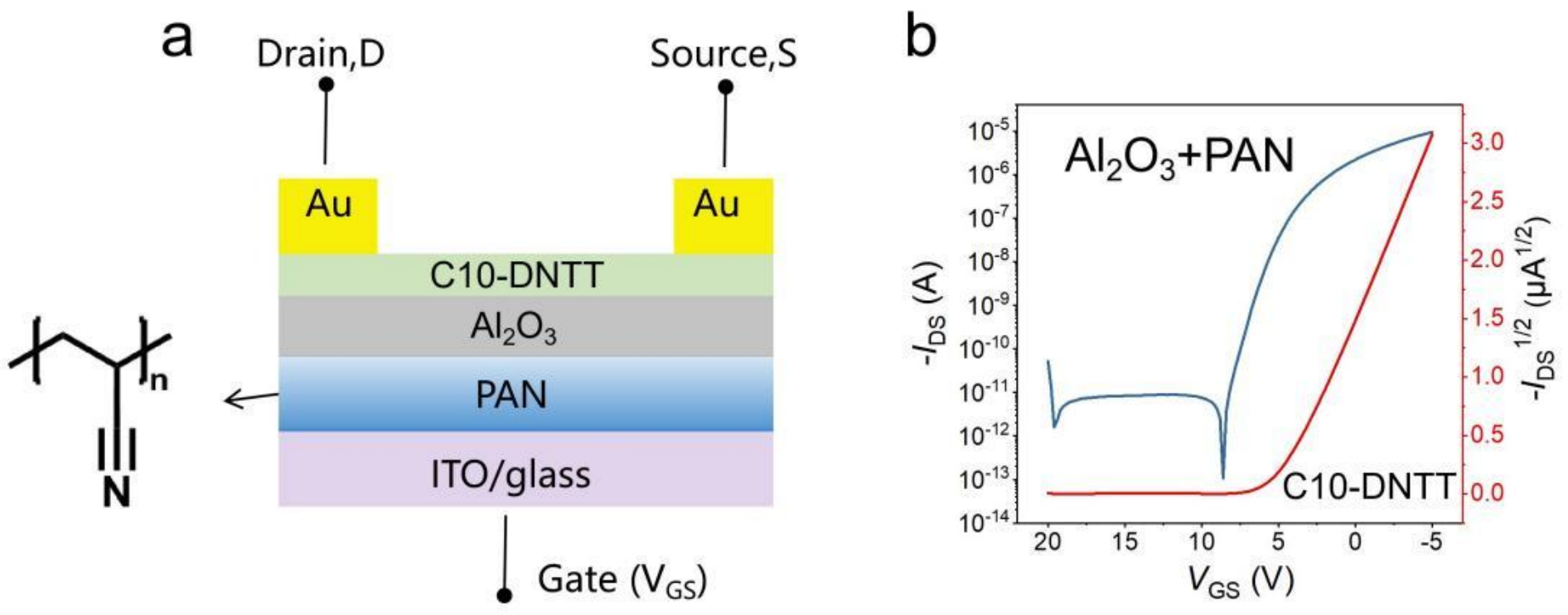

Supplementary Fig. 39 | OTFTs with C10-DNTT semiconductor on Al_2O_3/PAN dielectric. a, Device schematic. **b**, Transfer curves.

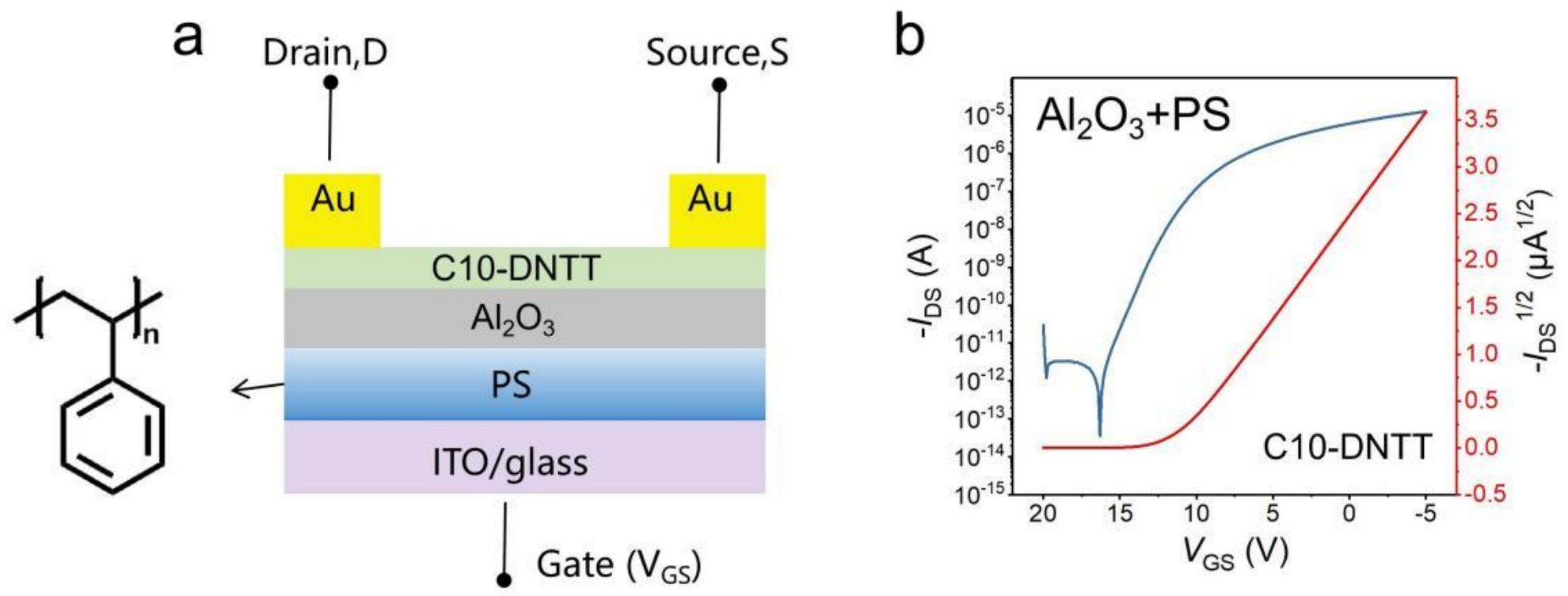

Supplementary Fig. 40 | OTFTs with C10-DNTT semiconductor on Al_2O_3/PS dielectric.

a, Device schematic. **b**, Transfer curves.

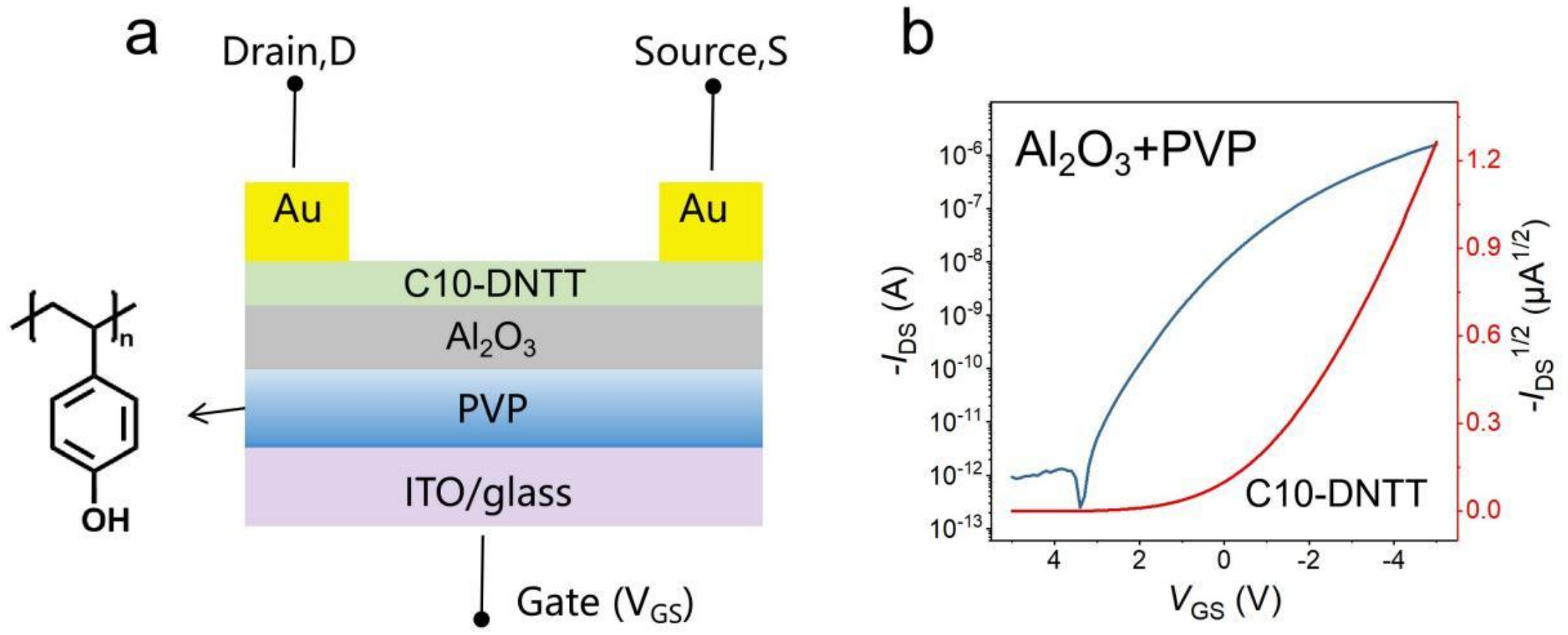

Supplementary Fig. 41 | OTFTs with C10-DNTT semiconductor on Al_2O_3/PVP dielectric.

a, Device schematic. **b**, Transfer curves.

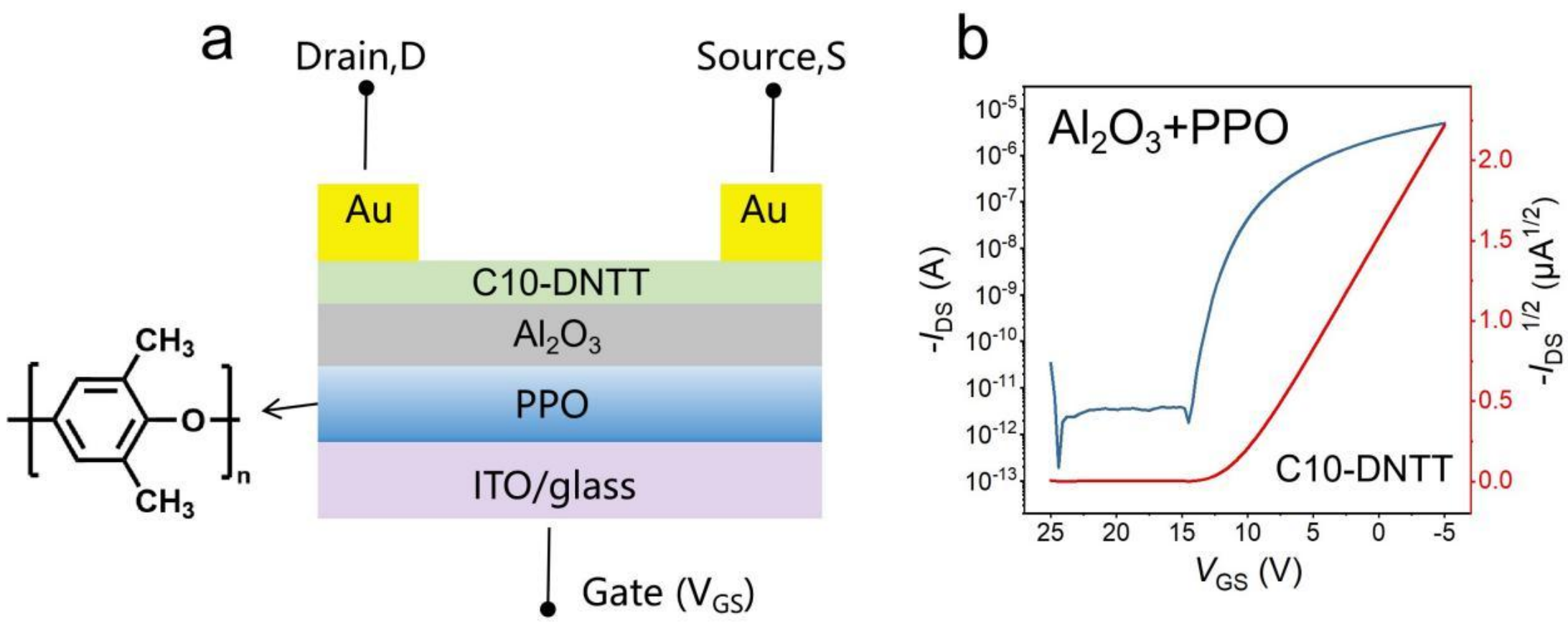

Supplementary Fig. 42 | OTFTs with C10-DNTT semiconductor on Al_2O_3/PPO dielectric. a, Device schematic. **b**, Transfer curves.

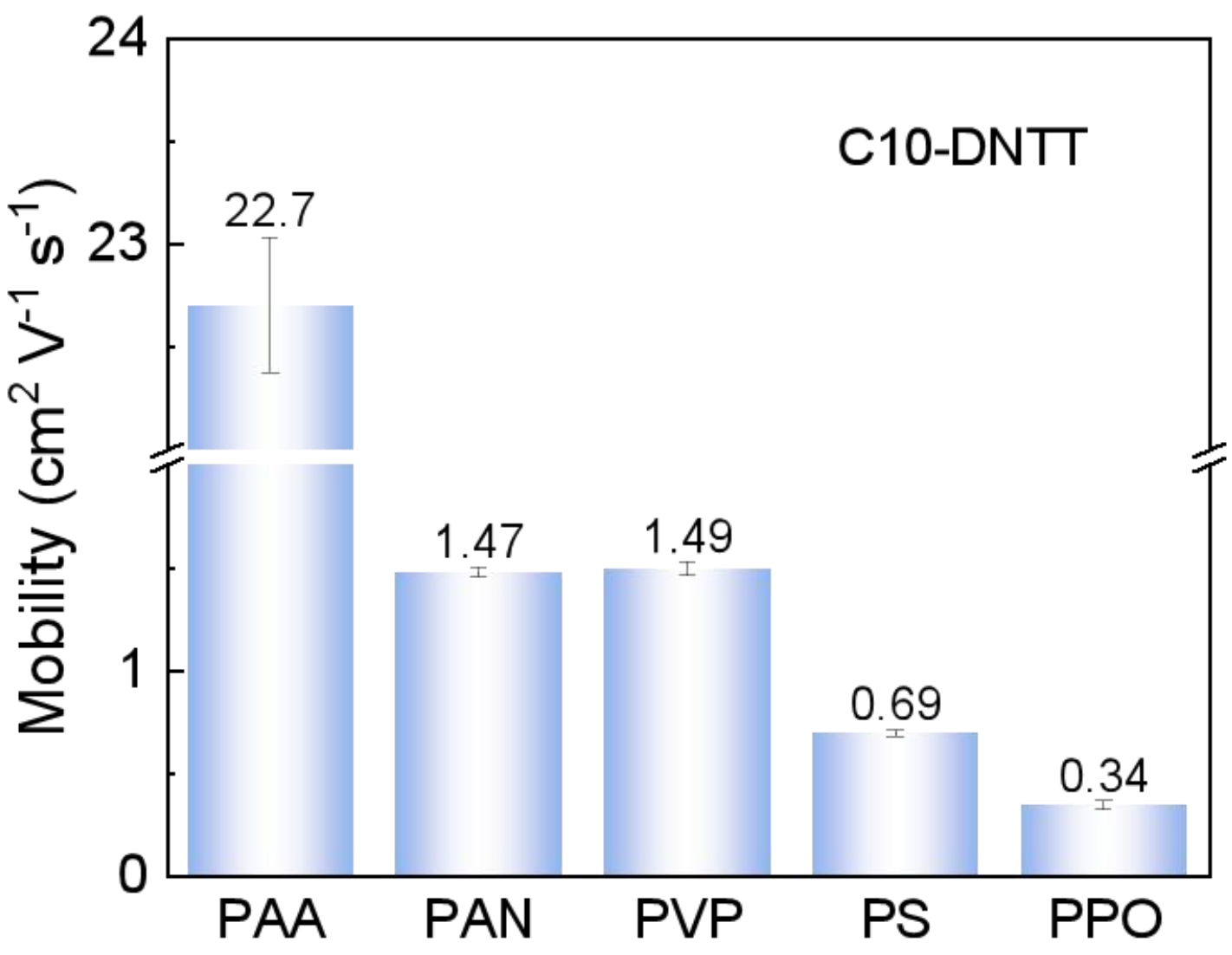

Supplementary Fig. 43 | Statistical comparison of field-effect mobility in Al_2O_3/polymer heterostructure dielectric-based OTFTs with C10-DNTT semiconductor, across different polymer configurations (*f* = 20 Hz). The devices with different dielectric layers exhibit a polarity-dependent trend in carrier mobility variation.

.

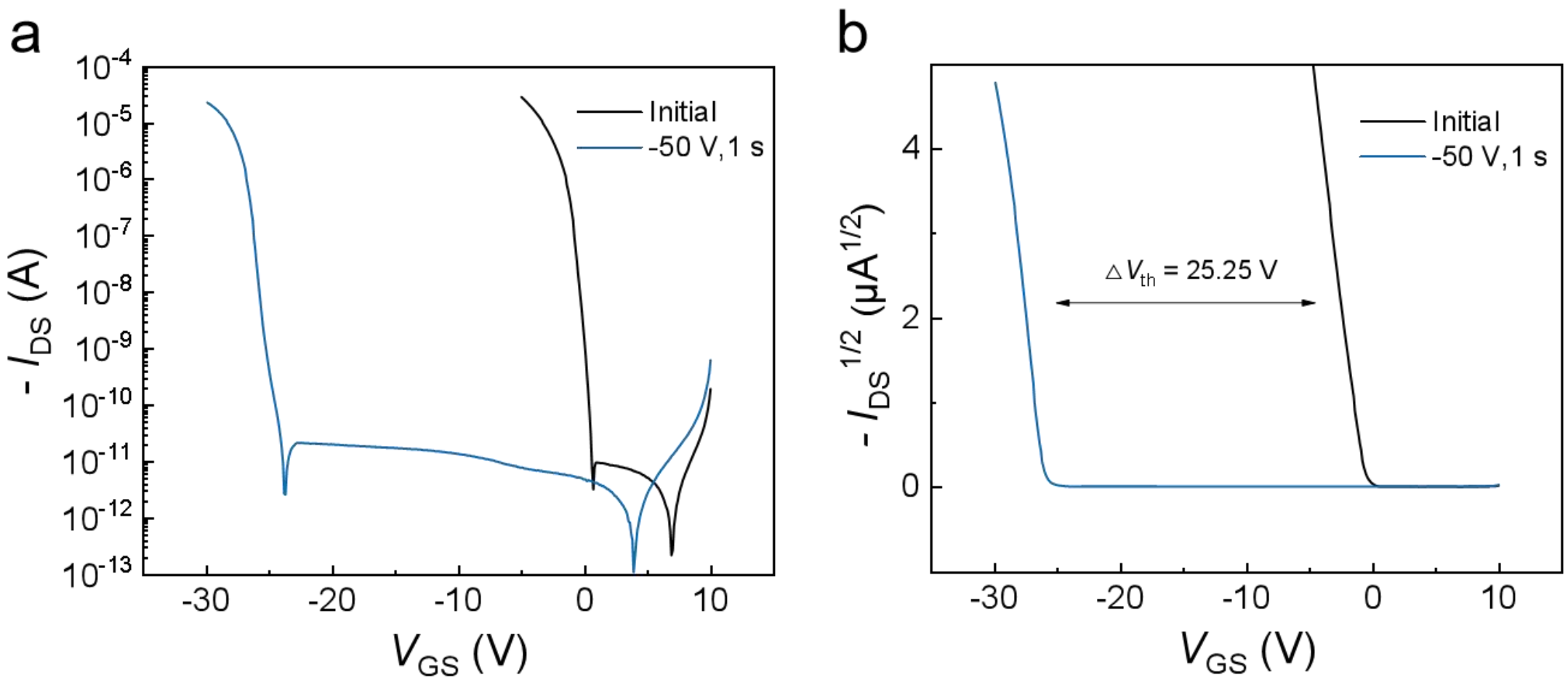

Supplementary Fig. 44 | Electrical performance of Al_2O_3/PAA-based C10-DNTT OTFTs under –50 V programming pulses (1 s). a, Transfer characteristics. **b**, Memory window (ΔV_{th}).

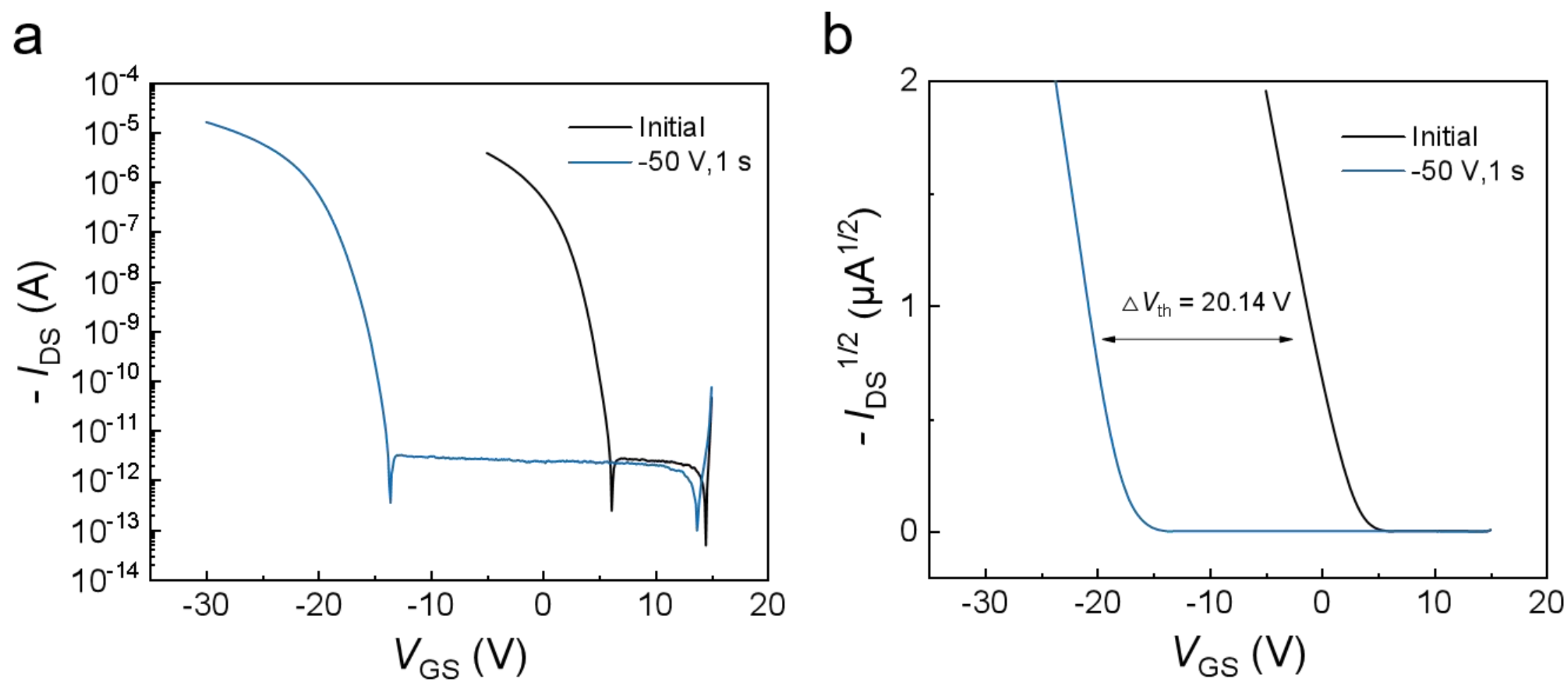

Supplementary Fig. 45 | Electrical performance of Al_2O_3/PAN-based C10-DNTT OTFTs under –50 V programming pulses (1 s). a, Transfer characteristics. **b**, Memory window (ΔV_{th}).

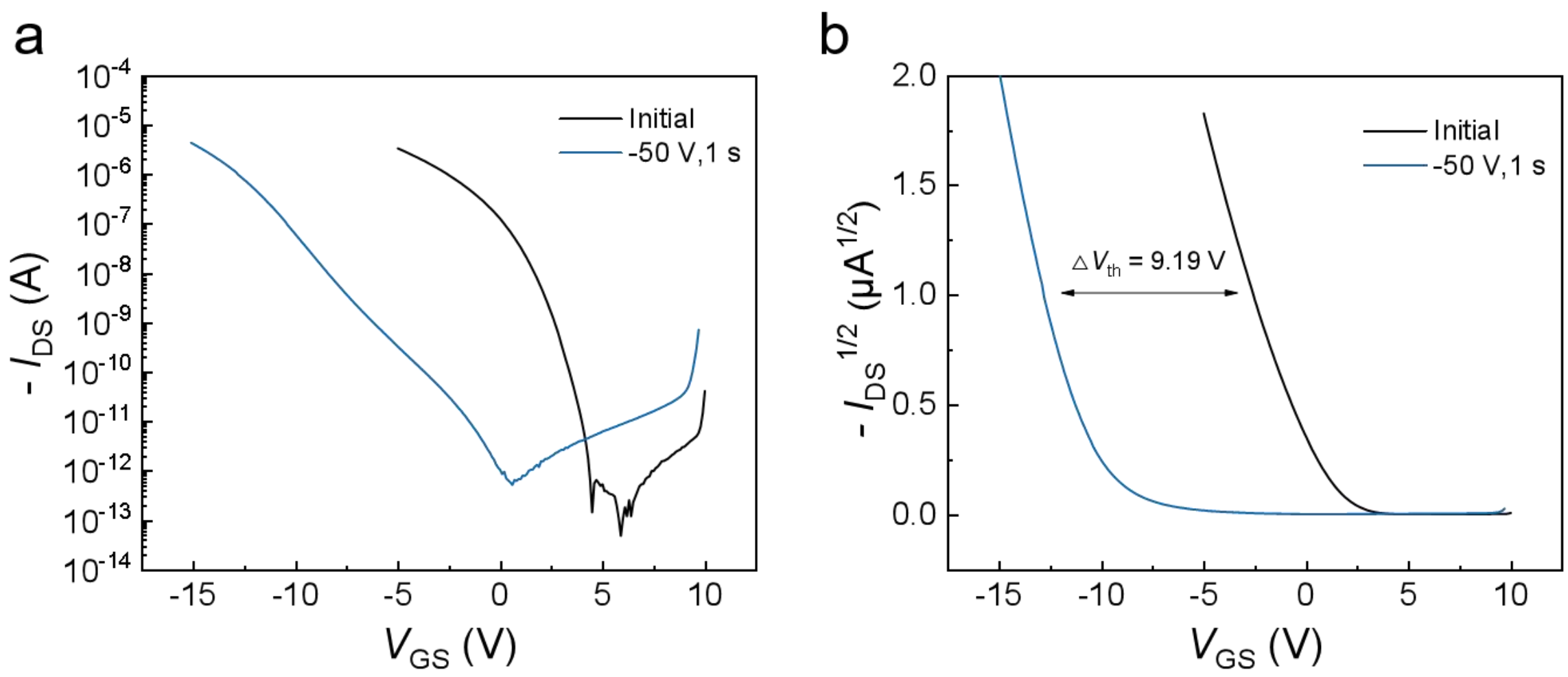

Supplementary Fig. 46 | Electrical performance of Al_2O_3/PVP-based C10-DNTT OTFTs under –50 V programming pulses (1 s). a, Transfer characteristics. **b**, Memory window (ΔV_{th}).

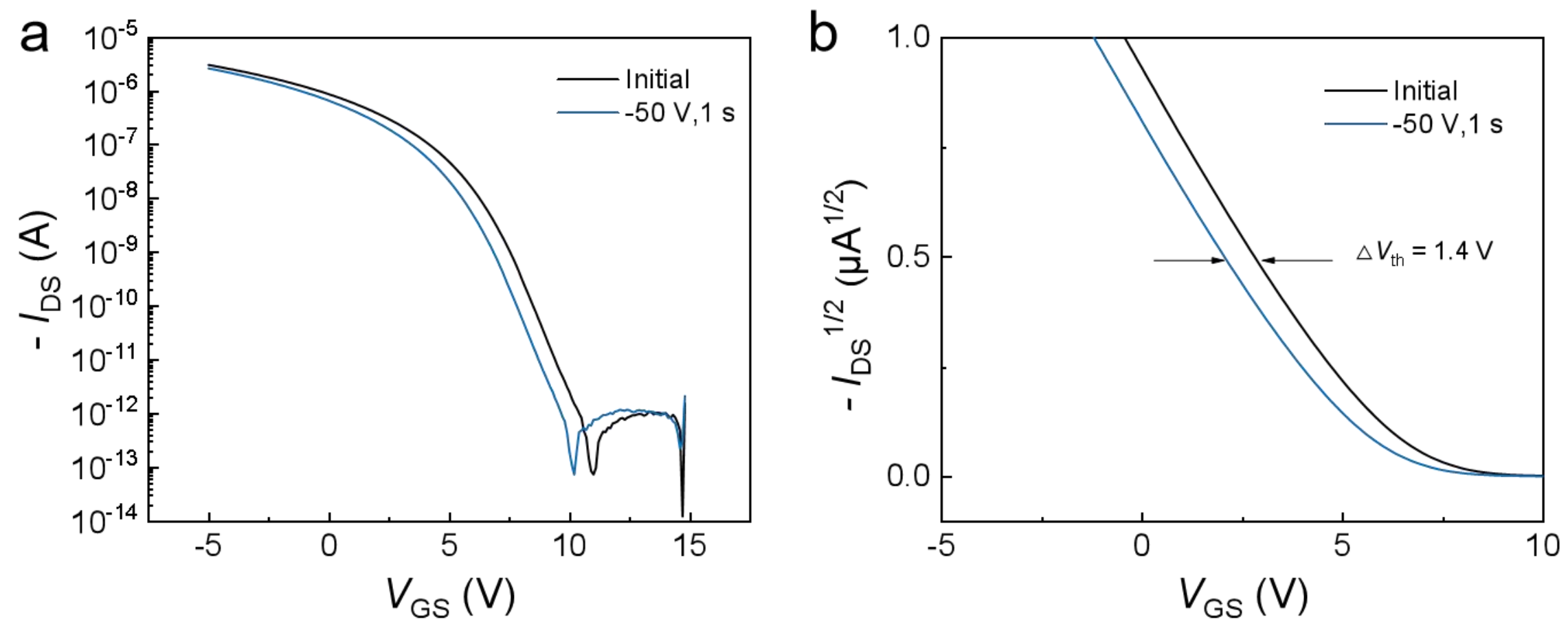

Supplementary Fig. 47 | Electrical performance of Al_2O_3/PS-based C10-DNTT OTFTs under –50 V programming pulses (1 s). a, Transfer characteristics. **b**, Memory window (ΔV_{th}).

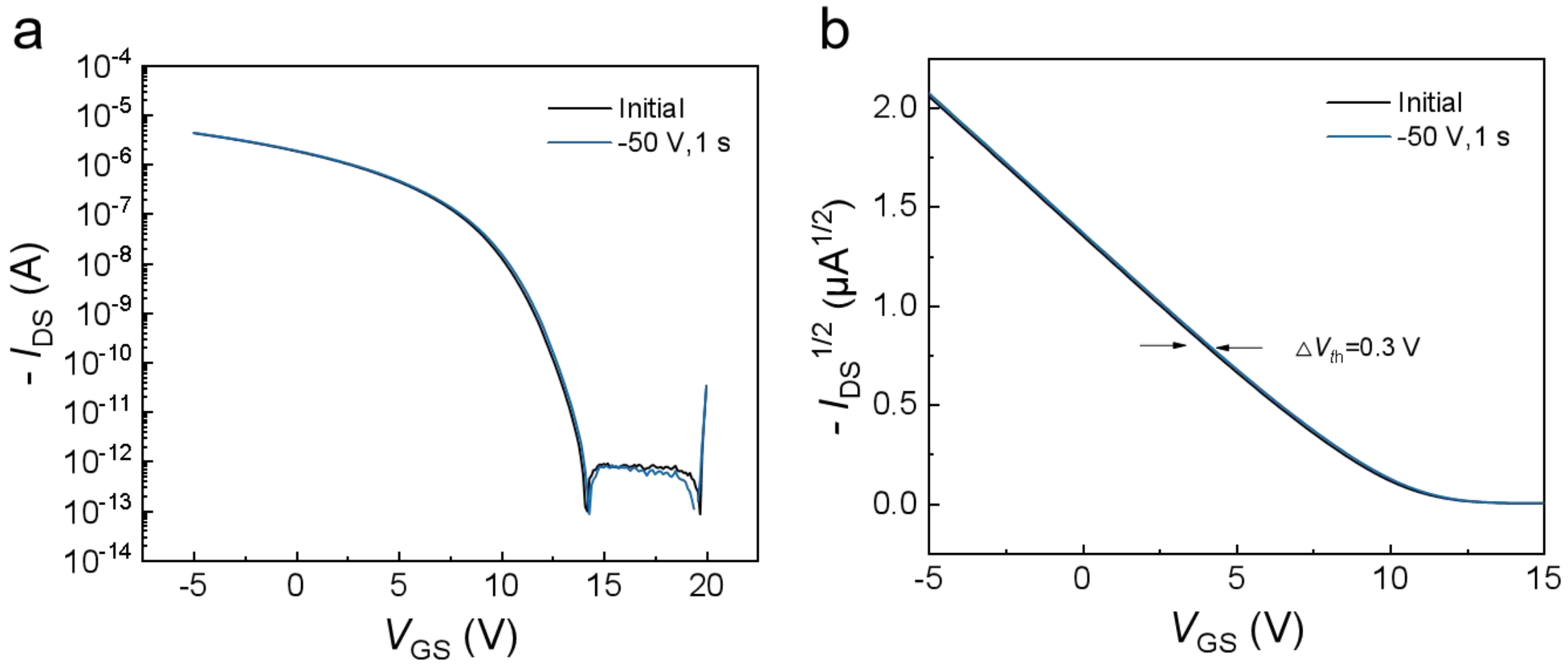

Supplementary Fig. 48 | Electrical performance of Al_2O_3/PPO-based C10-DNTT OTFTs under –50 V programming pulses (1 s). a, Transfer characteristics. **b**, Memory window (ΔV_{th}).

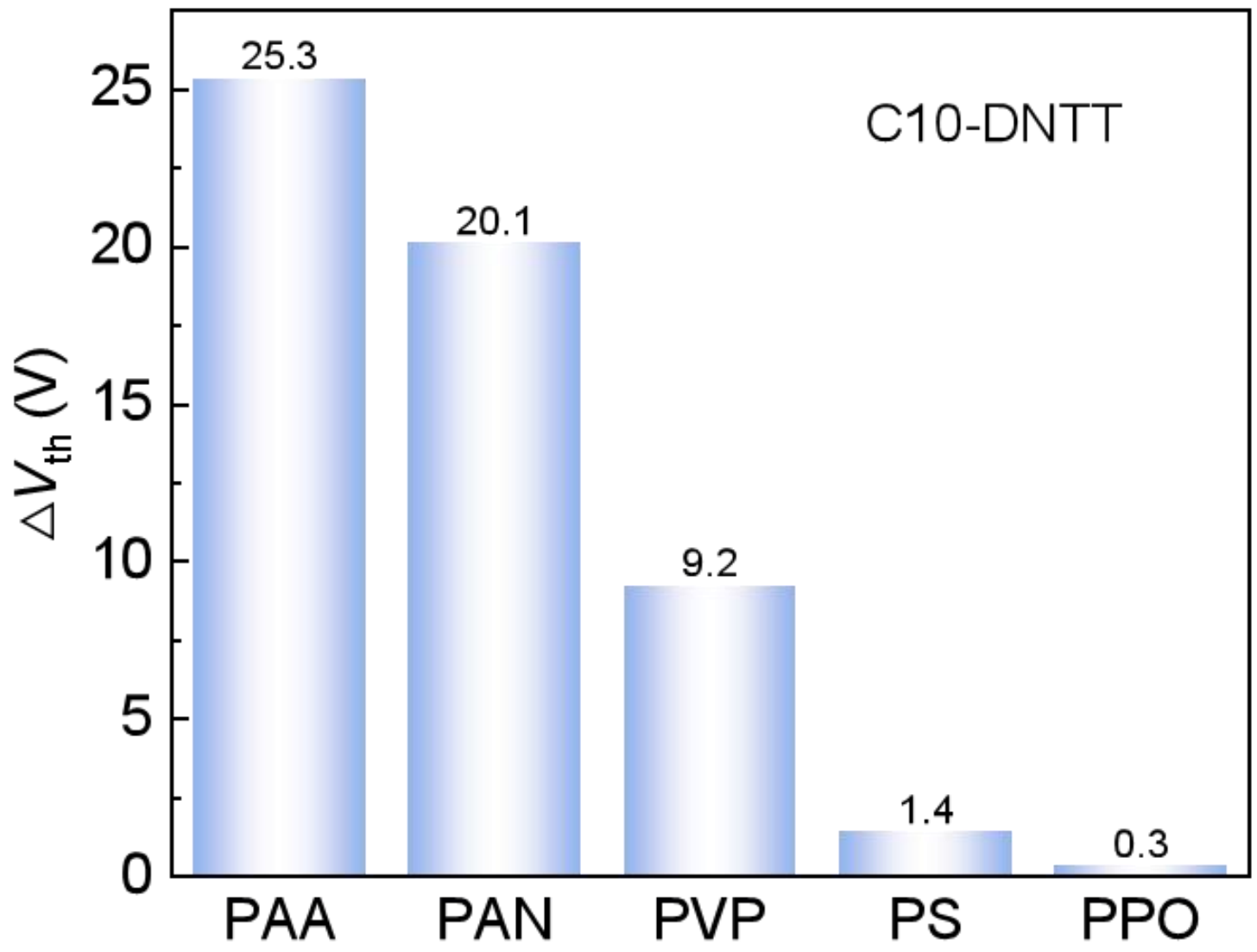

Supplementary Fig. 49 | Statistical comparison of storage window in Al_2O_3/polymer heterostructure dielectric-based OTFTs with C10-DNTT semiconductor, across different polymer configurations. The devices with different dielectric layers exhibit a polarity-dependent trend in storage window variation.

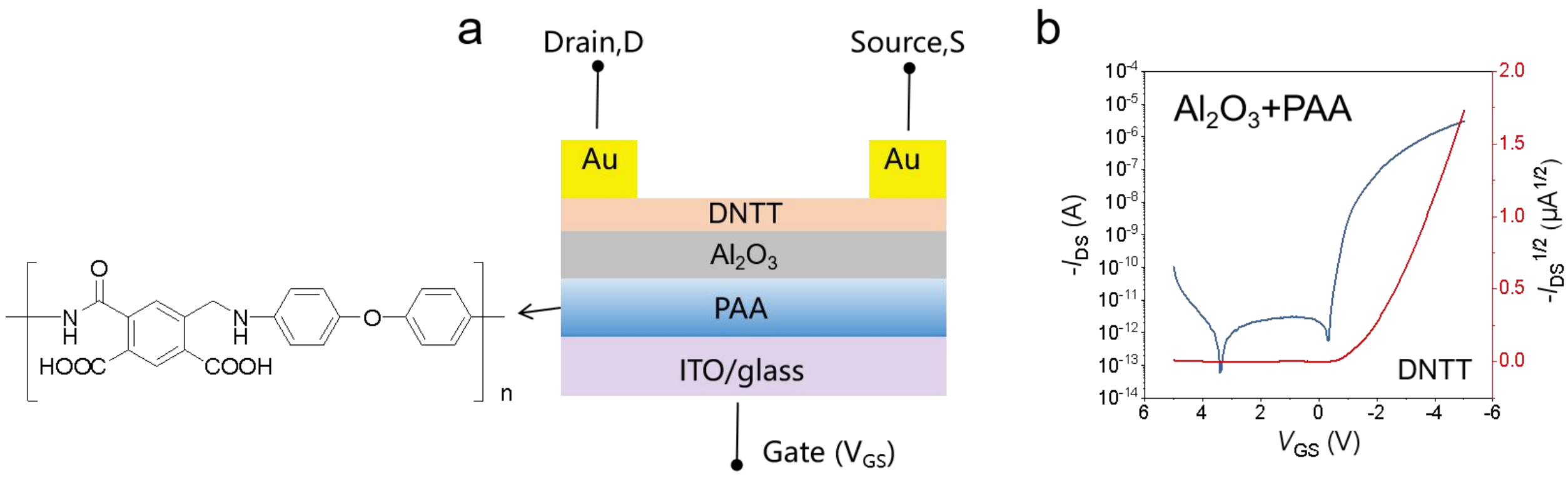

Supplementary Fig. 50 | OTFTs with DNTT semiconductor on Al_2O_3/PAA dielectric (replacing C10-DNTT). a, Device schematic. **b**, Transfer curves.

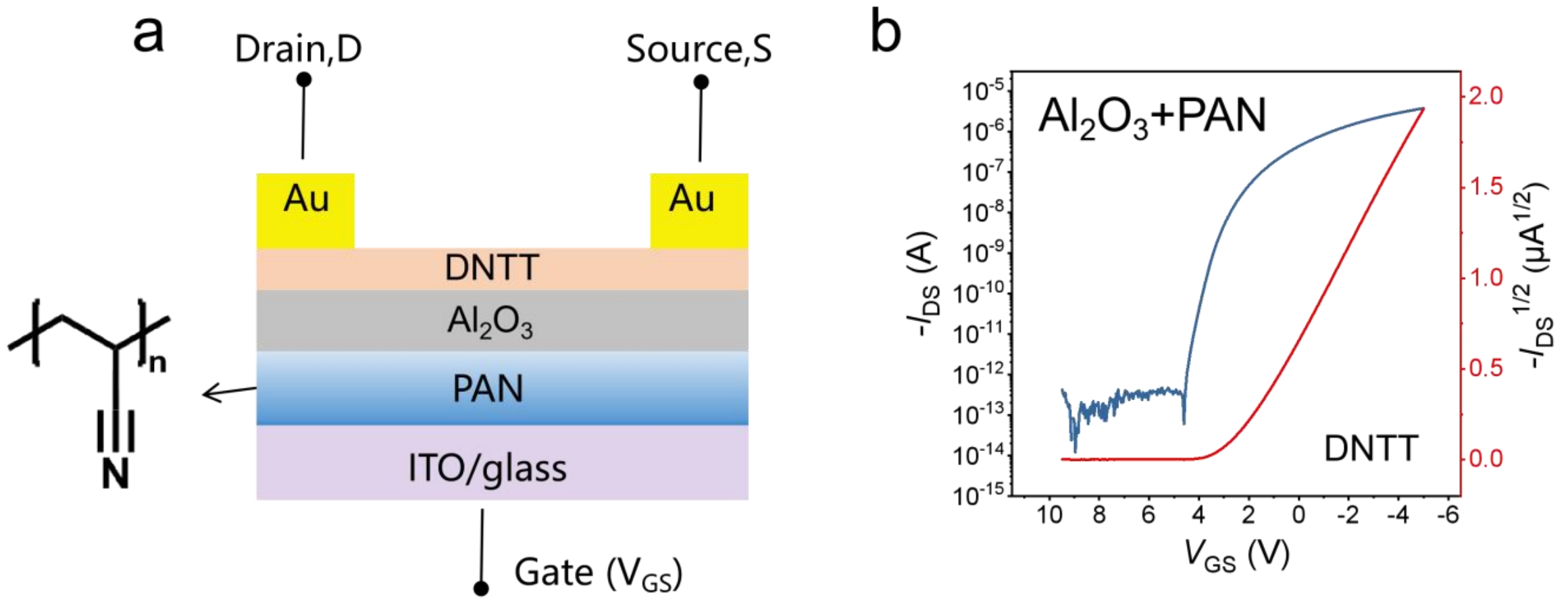

Supplementary Fig. 51 | OTFTs with DNTT semiconductor on Al_2O_3/PAN dielectric (replacing C10-DNTT). a, Device schematic. **b**, Transfer curves.

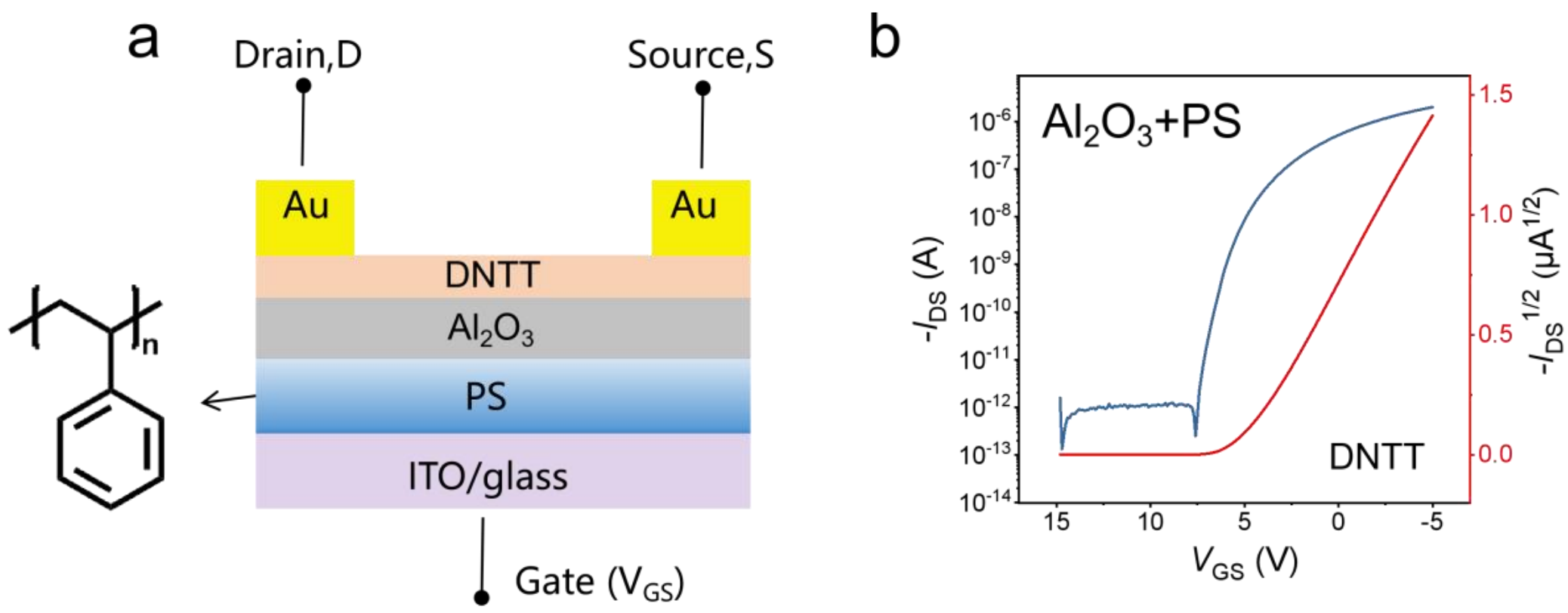

Supplementary Fig. 52 | OTFTs with DNTT semiconductor on Al_2O_3/PS dielectric (replacing C10-DNTT). a, Device schematic. **b**, Transfer curves.

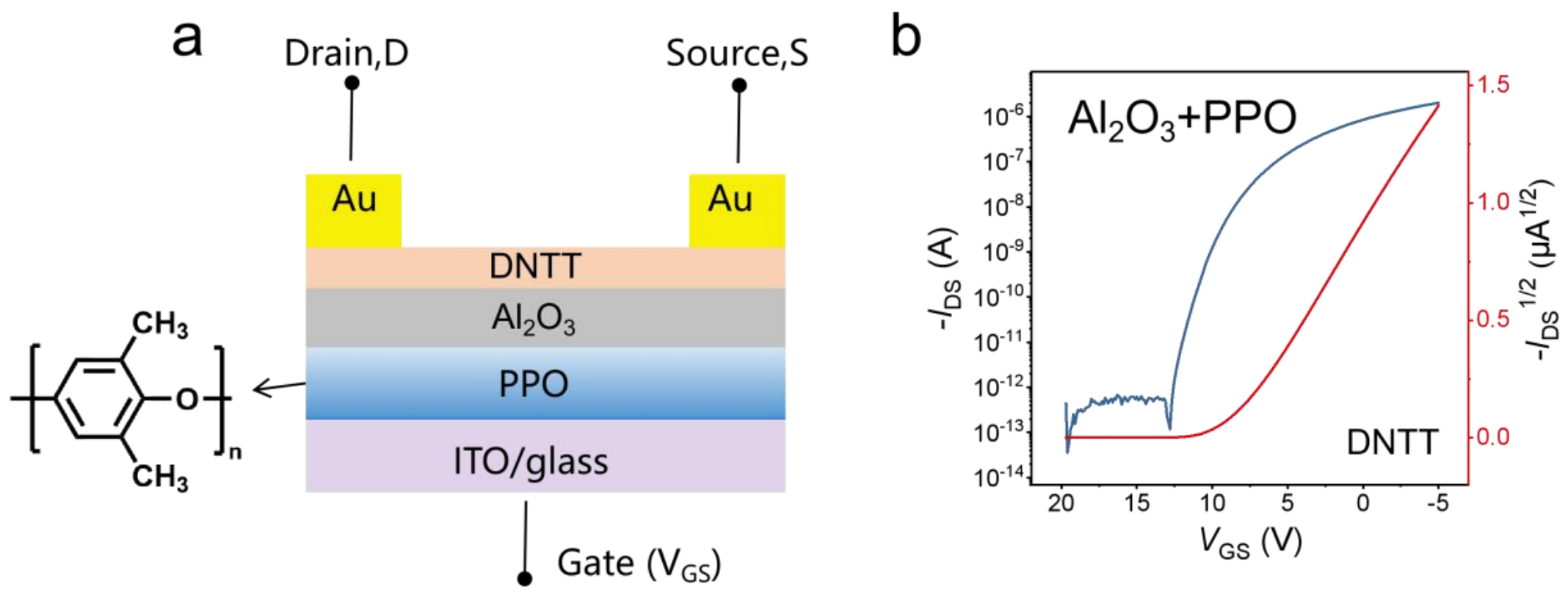

Supplementary Fig. 53 | OTFTs with DNTT semiconductor on Al_2O_3/PPO dielectric (replacing C10-DNTT). a, Device schematic. **b**, Transfer curves.

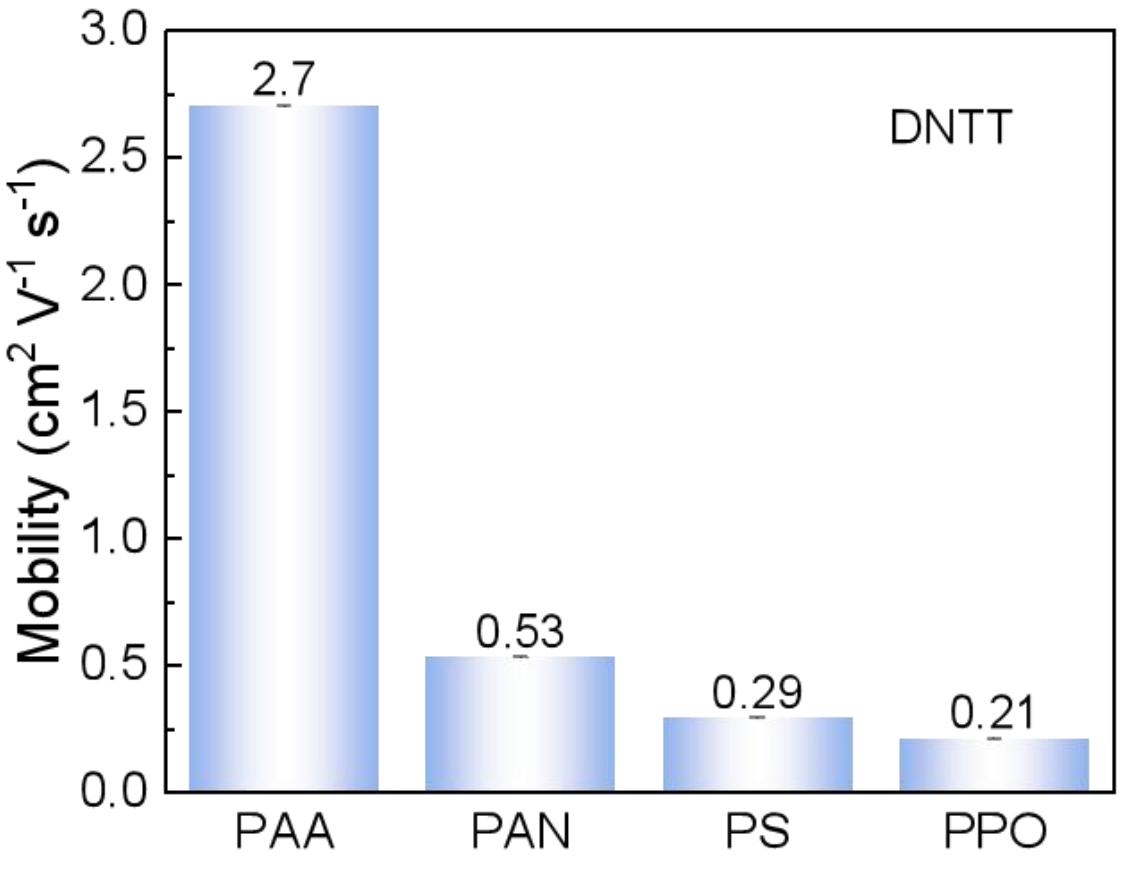

Supplementary Fig. 54 | Statistical comparison of field-effect mobility in Al_2O_3/polymer heterostructure dielectric-based OTFTs with DNTT semiconductor, across different polymer configurations (*f* = 20 Hz). The devices with different dielectric layers exhibit a polarity-dependent trend in carrier mobility variation.

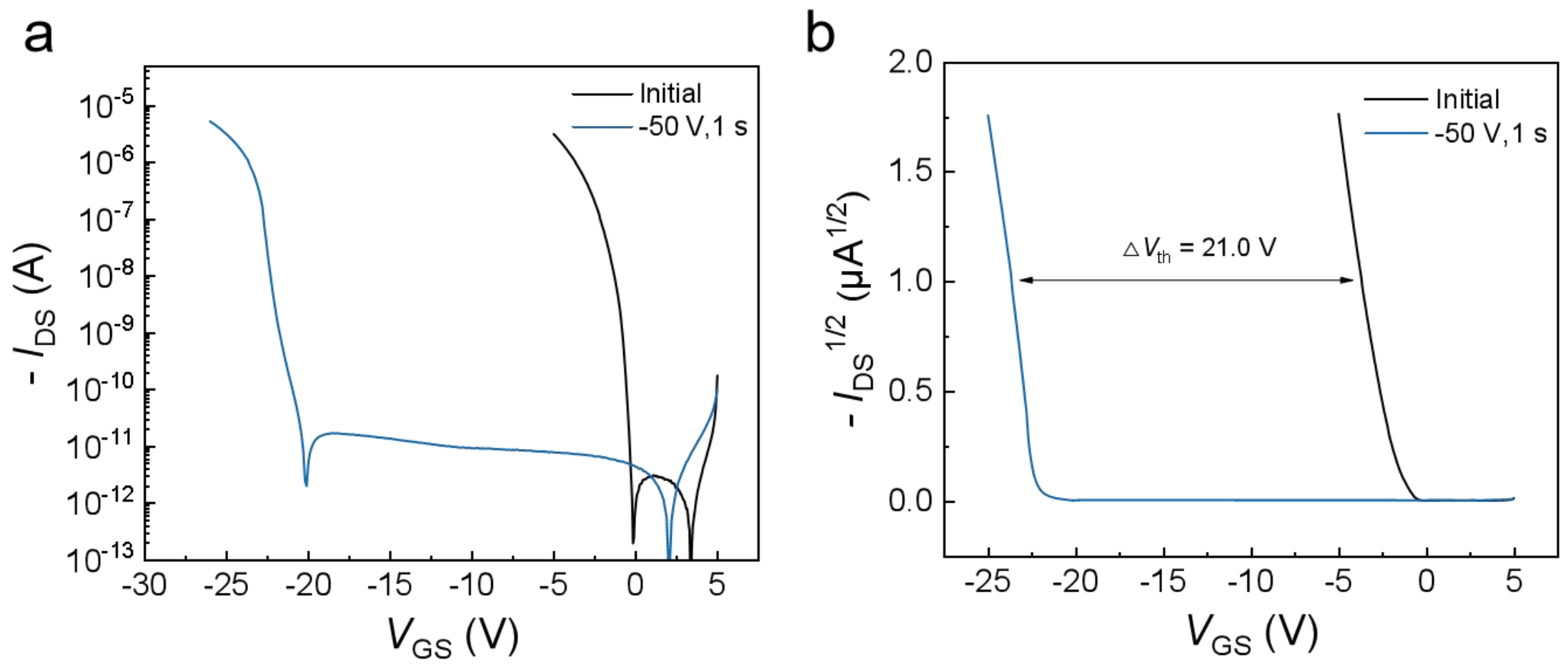

Supplementary Fig. 55 | Electrical performance of Al_2O_3/PAA-based DNTT OTFTs under –50 V programming pulses (1 s). a, Transfer characteristics. **b**, Memory window (ΔV_{th}).

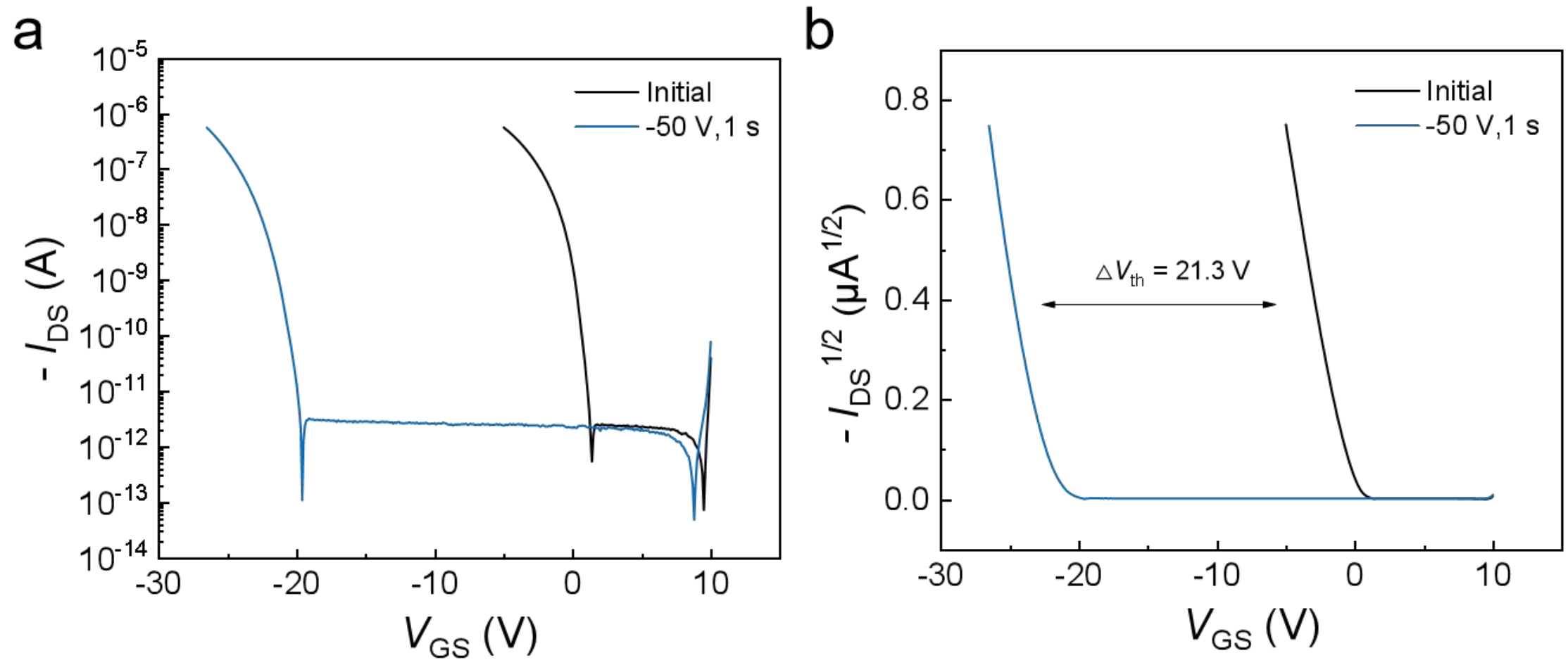

Supplementary Fig. 56 | Electrical performance of Al_2O_3/PAN-based DNTT OTFTs under –50 V programming pulses (1 s). a, Transfer characteristics. **b**, Memory window (ΔV_{th}).

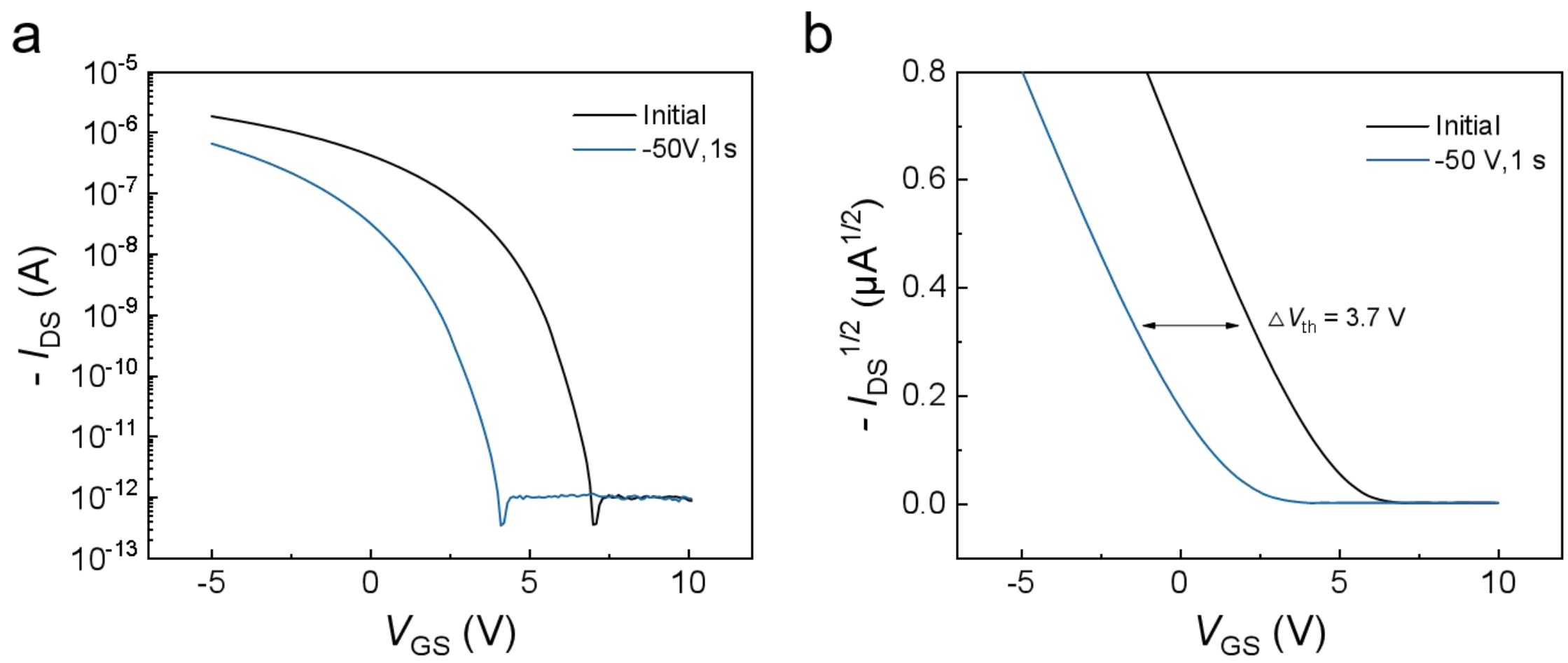

Supplementary Fig. 57 | Electrical performance of Al_2O_3/PS-based DNTT OTFTs under –50 V programming pulses (1 s). a, Transfer characteristics. **b**, Memory window (ΔV_{th}).

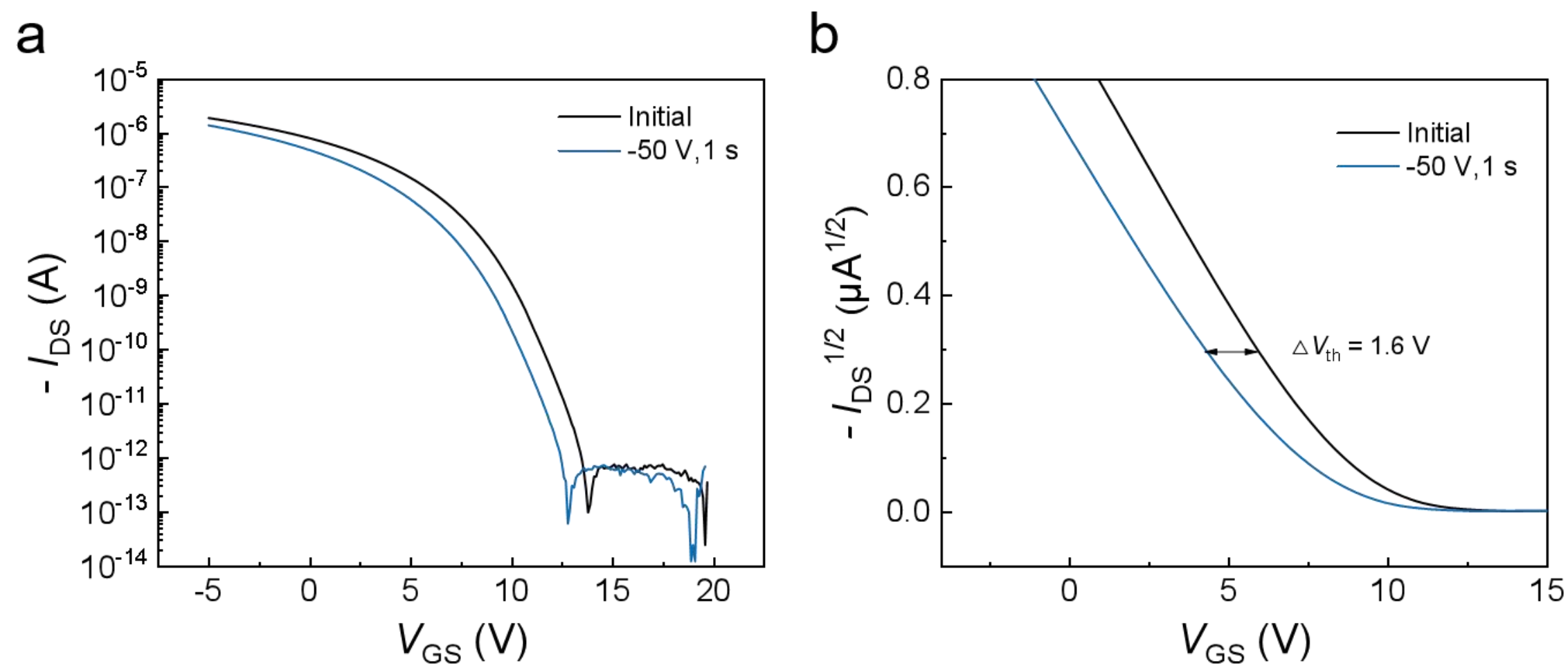

Supplementary Fig. 58 | Electrical performance of Al_2O_3/PPO-based DNTT OTFTs under –50 V programming pulses (1 s). a, Transfer characteristics. **b**, Memory window (ΔV_{th}).

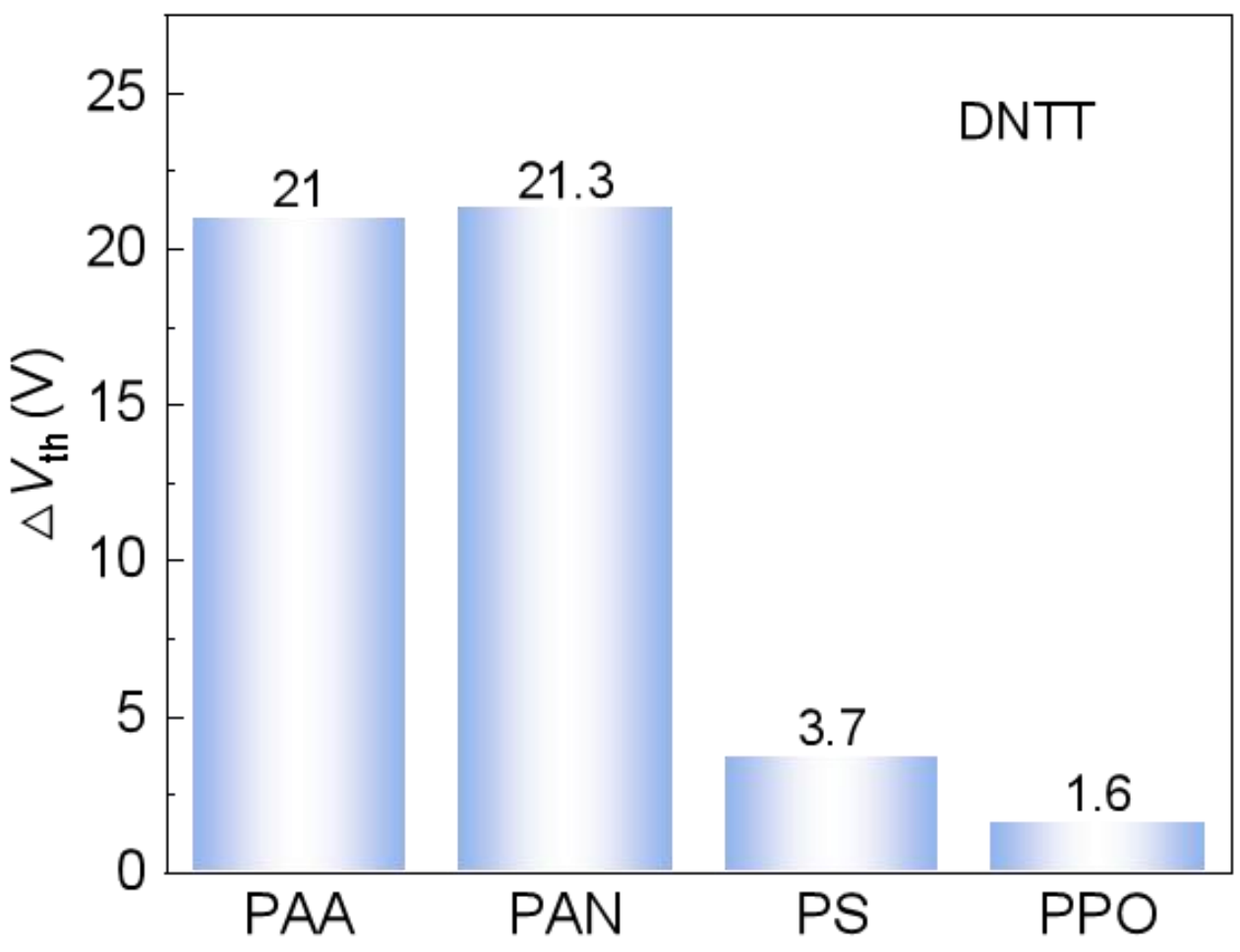

Supplementary Fig. 59 | Statistical comparison of storage window in Al_2O_3/polymer heterostructure dielectric-based OTFTs with DNTT semiconductor, across different polymer configurations. The devices with different dielectric layers exhibit a polarity-dependent trend in storage window variation.

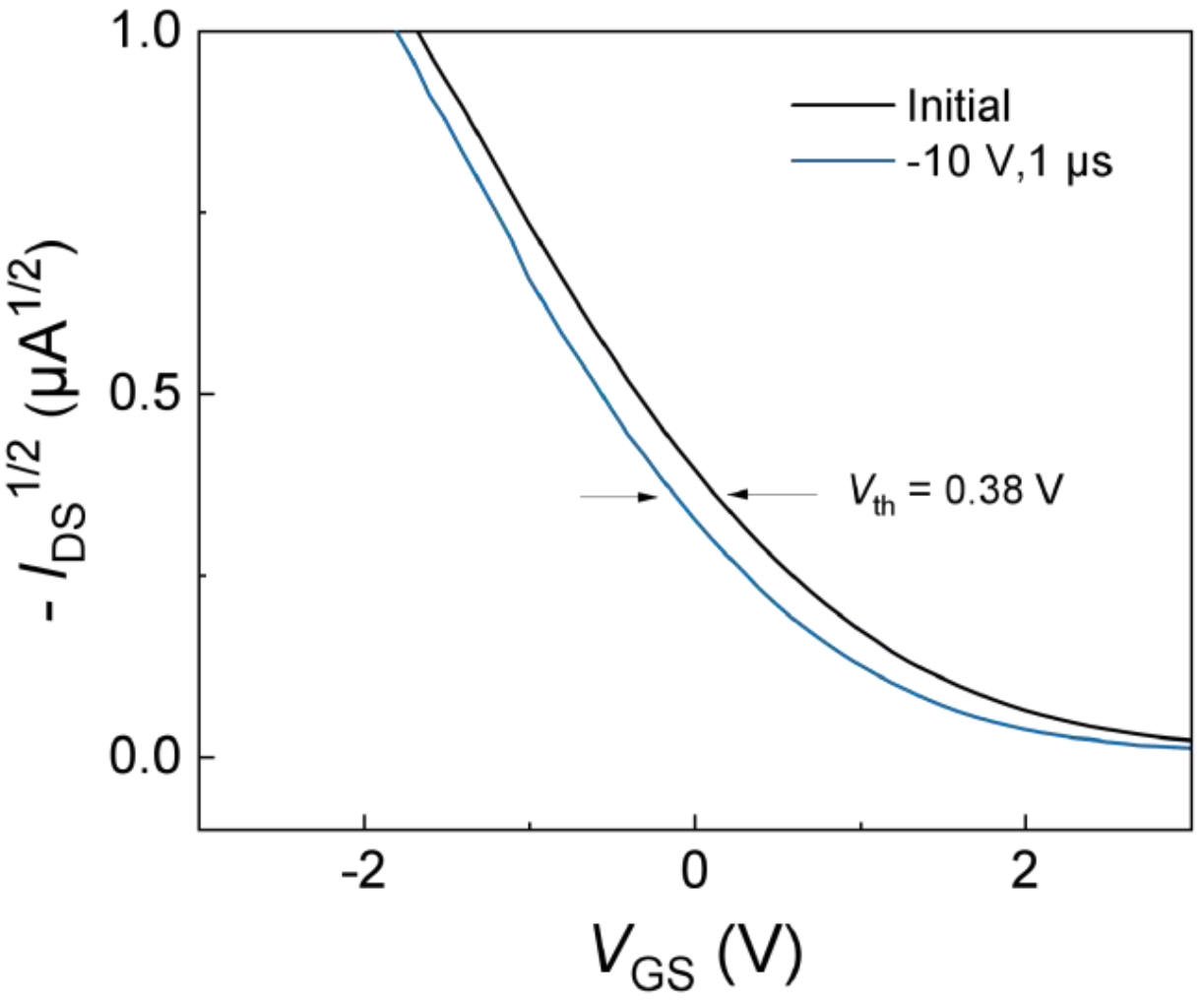

Supplementary Figure 60 | The storage window of the device following the application of a –10 V, 1 μs programming pulse. A slight negative shift in the transfer curve was observed, indicating limited charge retention capability over this short time scale.

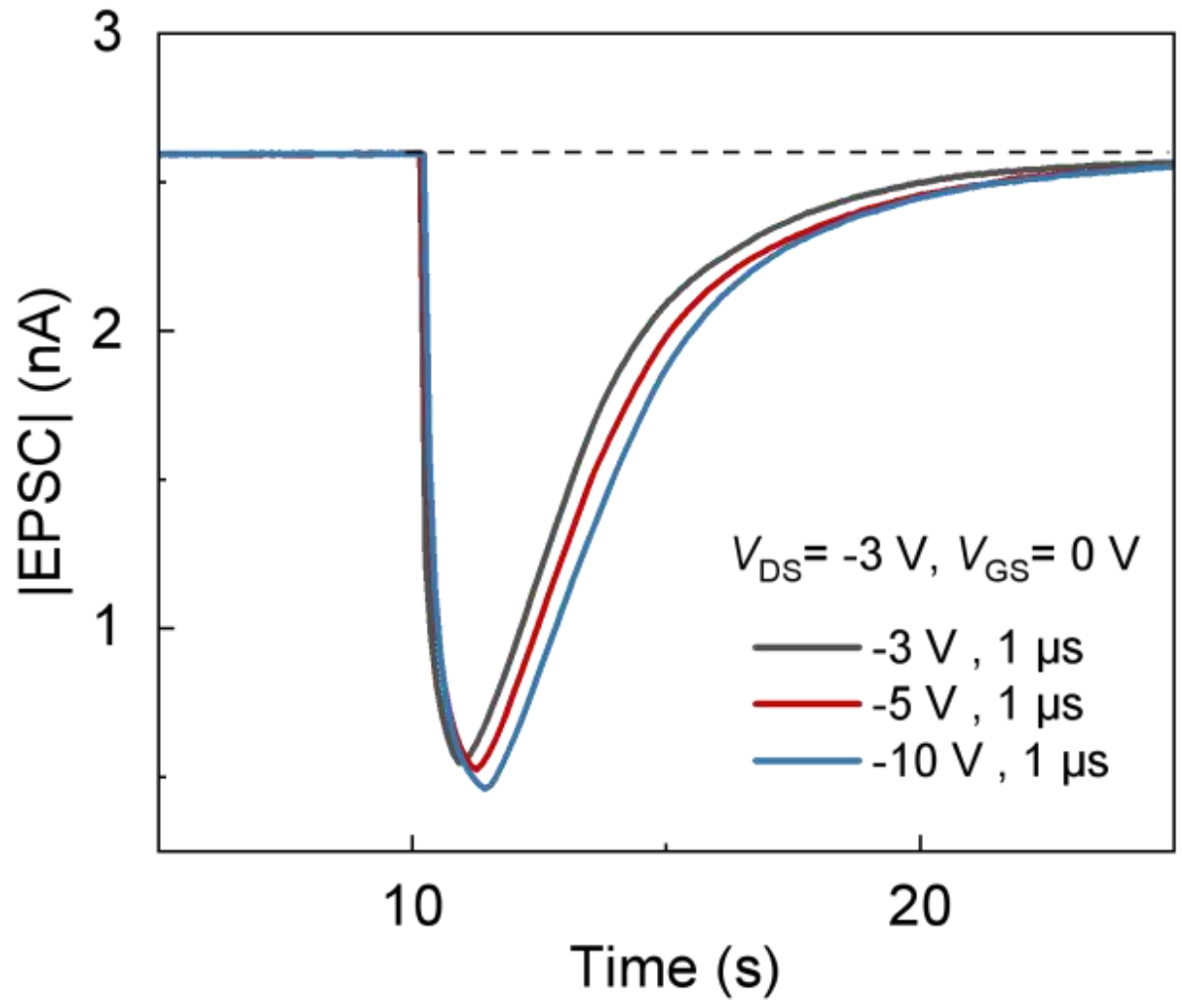

Supplementary Fig. 61 | Real-time drain current modulation under 1 μs gate pulses. The device exhibits clear current responses to –3 V, –5 V and –10 V pulses (1 μs width), confirming electrical modulation at sub-microsecond timescales, though the changes are transient rather than non-volatile.

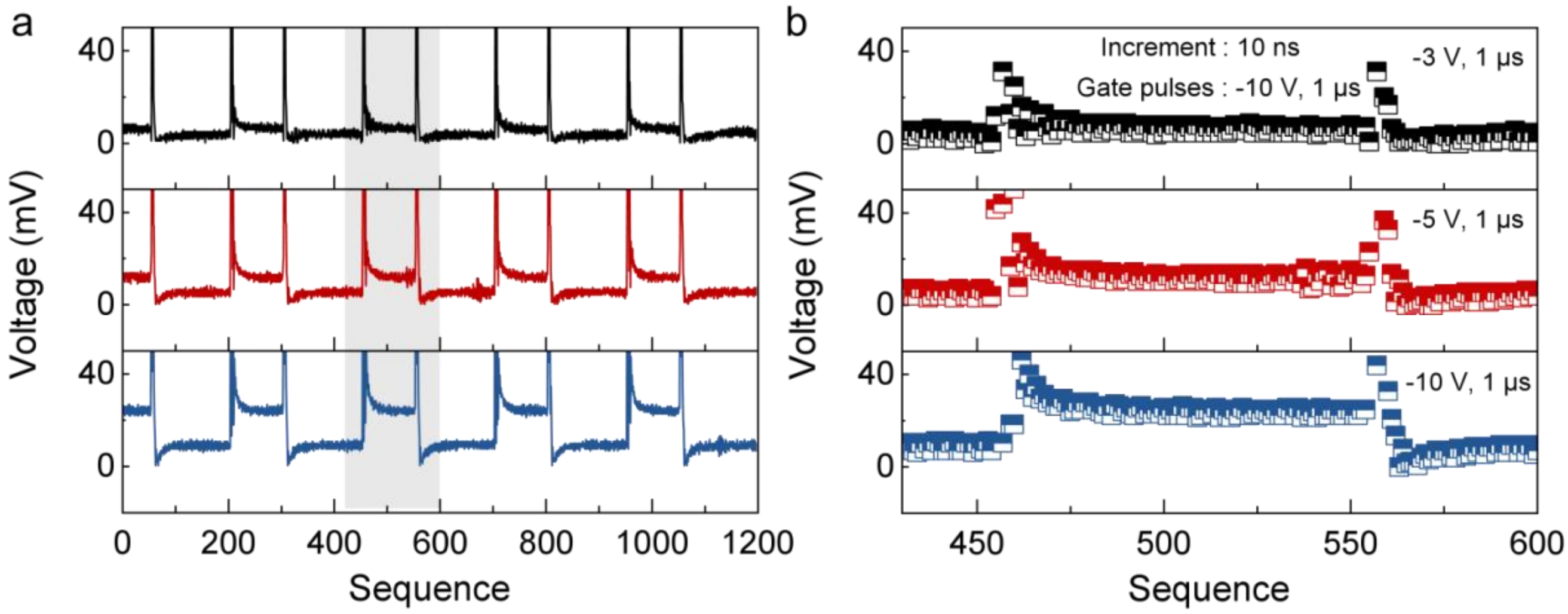

Supplementary Fig. 62 | Real-time electrical response to 1 μs gate pulses measured with a current pre-amplifier and oscilloscope (10 ns sampling interval). a, Transient drain current response to multiple 1 μs pulses at –3 V, –5 V and –10 V. **b**, Enlarged single-pulse view, resolving the current dynamics on the sub-microsecond scale. The clear modulation confirms the device's fast electrical responsiveness, though the response is transient rather than non-volatile.

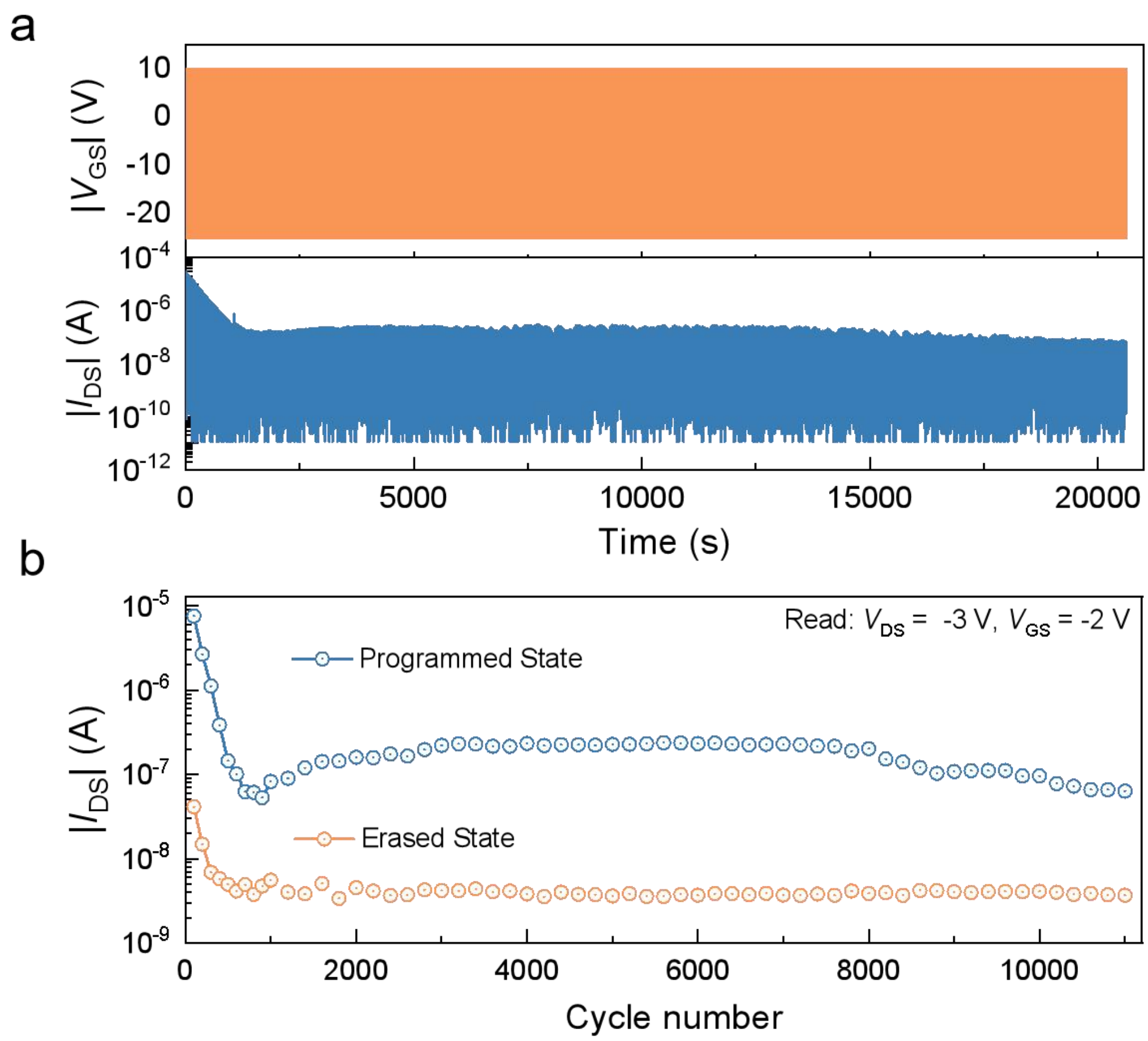

Supplementary Fig. 63 |Cyclic endurance of flexible Al_2O_3/PAA-based OTFT memory devices. a, Full programming–read–erase–read sequence under repeated voltage pulses. **b,** Programming and erasure currents extracted from the endurance test (partial data for >10,000 cycles).

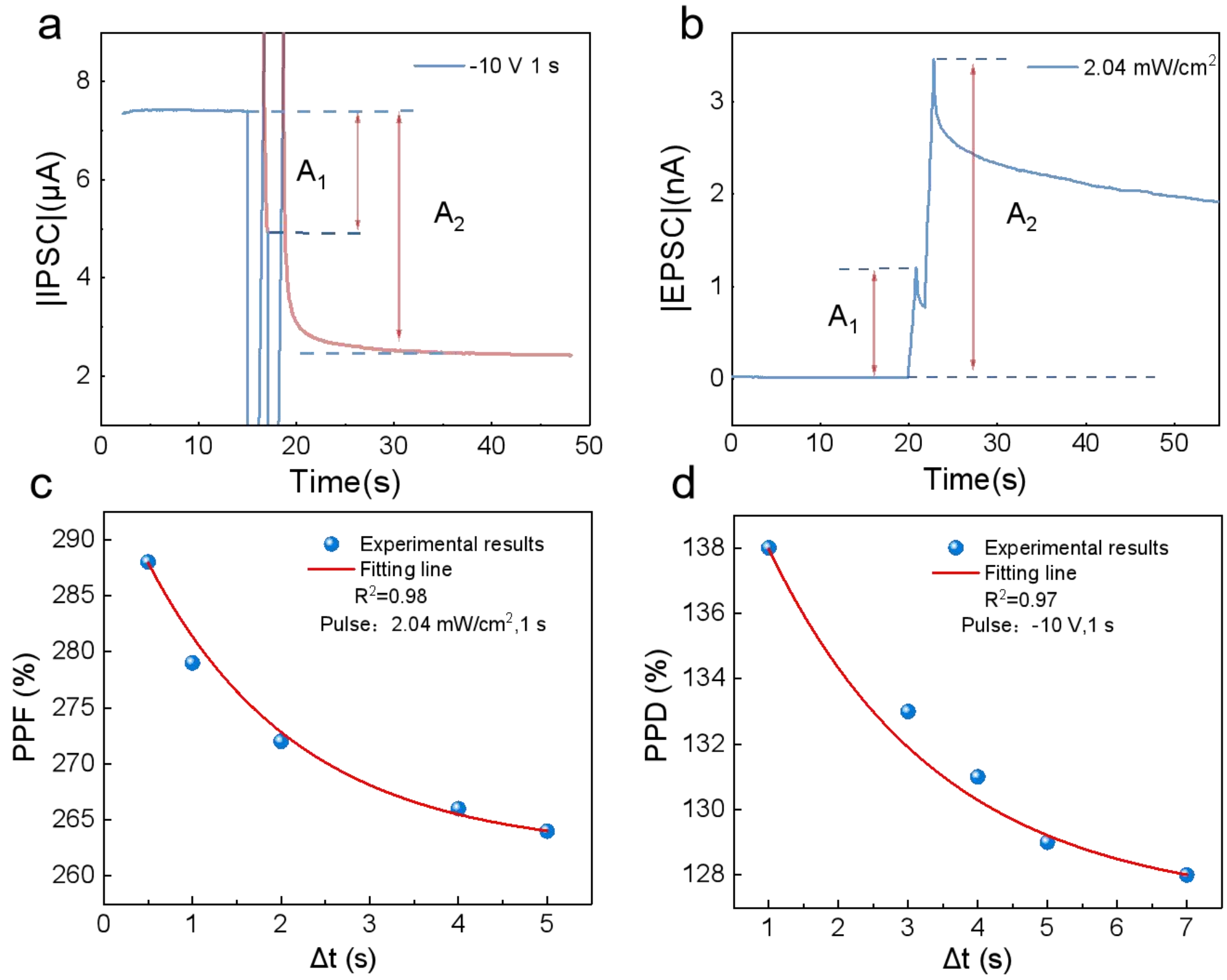

Supplementary Fig. 64 | Electrical and optical paired- pulse plasticity in Al_2O_3/PAA-based OTFTs. a, PPD negative voltage pulses (-10 V, 1 sec) were applied to the OTFTs. **b,** PPF behavior triggered by applying a pair of light pulses (450 nm, 2.04 mW/cm^2, 1 s). **c,** PPF index as a function of light pulse time interval (Δ*t*) with a light intensity of 0.22 mW/cm^2 and a pulse width of 1 s. **d,** PPD exponential as a function of the negative voltage pulse time interval (Δ*t*). The red solid line is obtained by fitting the exponential function.

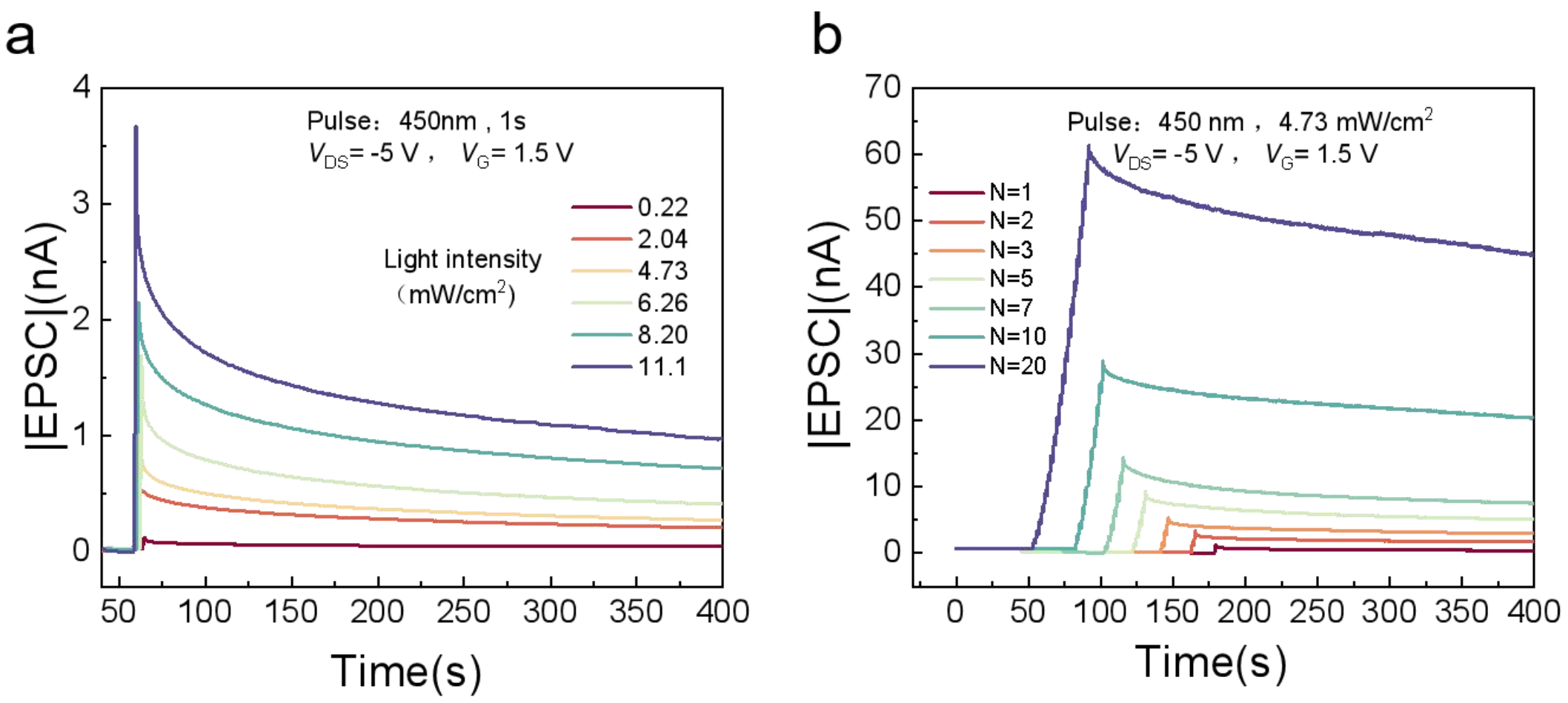

Supplementary Fig. 65 | STP-to-LTP transition of Al_2O_3/PAA-based OTFT devices triggered by light pulses. a, Response to increasing pulse intensity. **b**, Response to increasing pulse number.

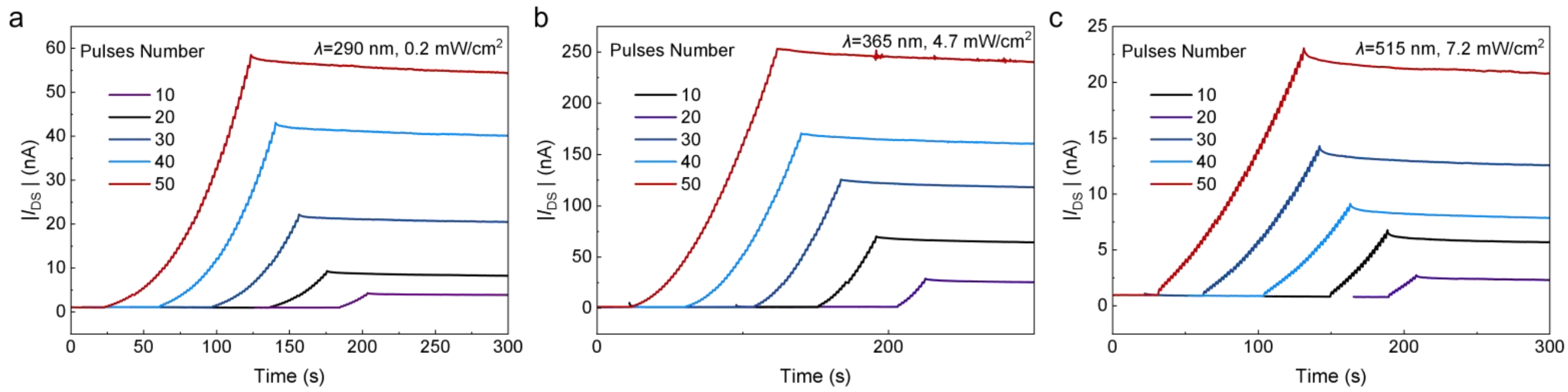

Supplementary Fig. 66 | Multi-pulse photoresponse behaviour of the 30 nm Al_2O_3/180 nm PAA device at different wavelengths. a–c, Excitatory postsynaptic current responses measured under consecutive light pulses (10, 20, 30, 40 and 50 pulses) at 290 nm (**a**), 365 nm (**b**) and 515 nm (**c**). At all tested wavelengths, the device exhibits reproducible pulse-number-dependent EPSC enhancement, mirroring the synaptic behaviour observed at 450 nm.

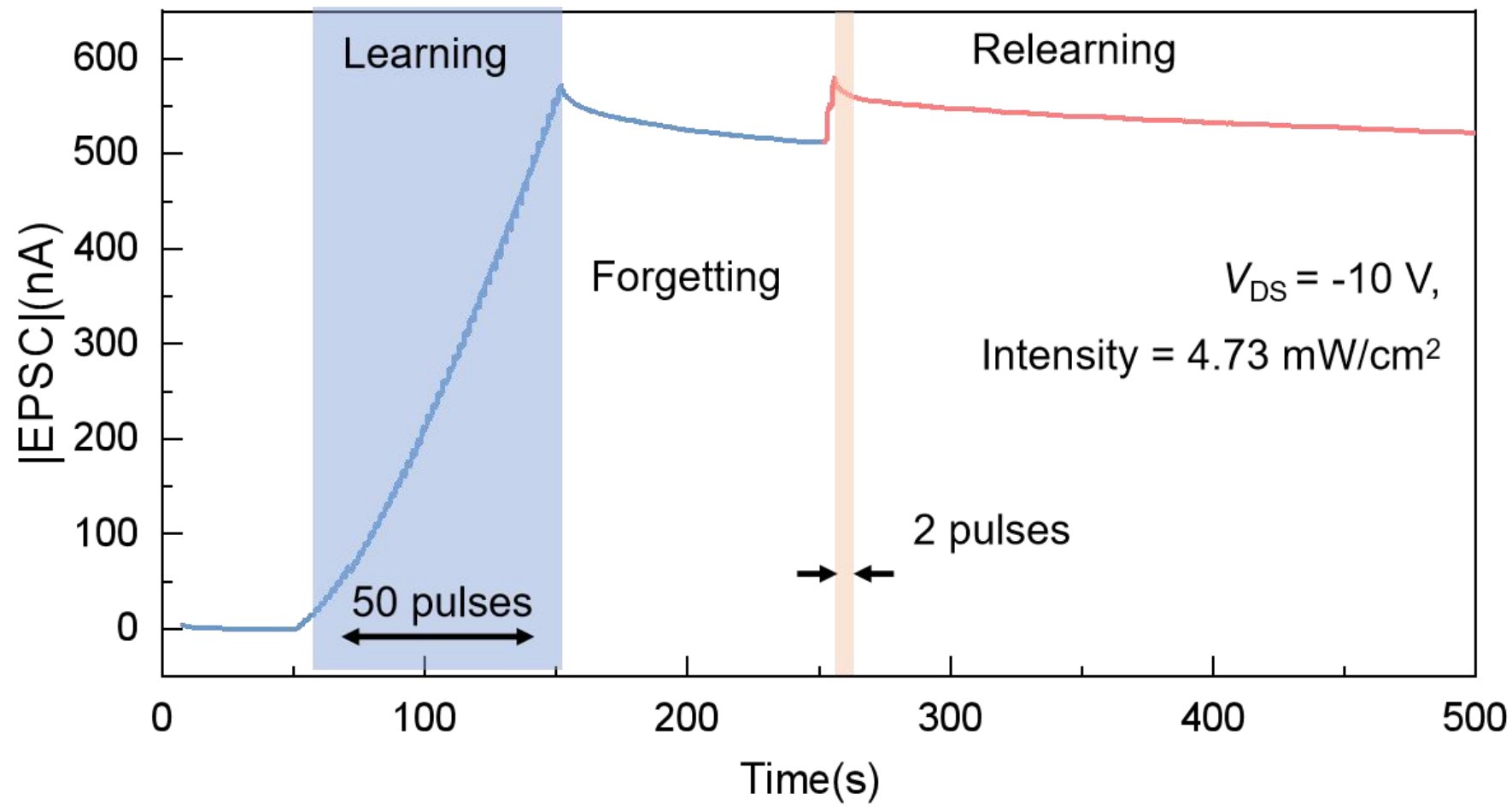

Supplementary Fig. 67 | Learning-forgetting-relearning behavior under light pulse stimulation training. Light pulse: 450 nm, 2.04 mW/cm^2 for 1 s.

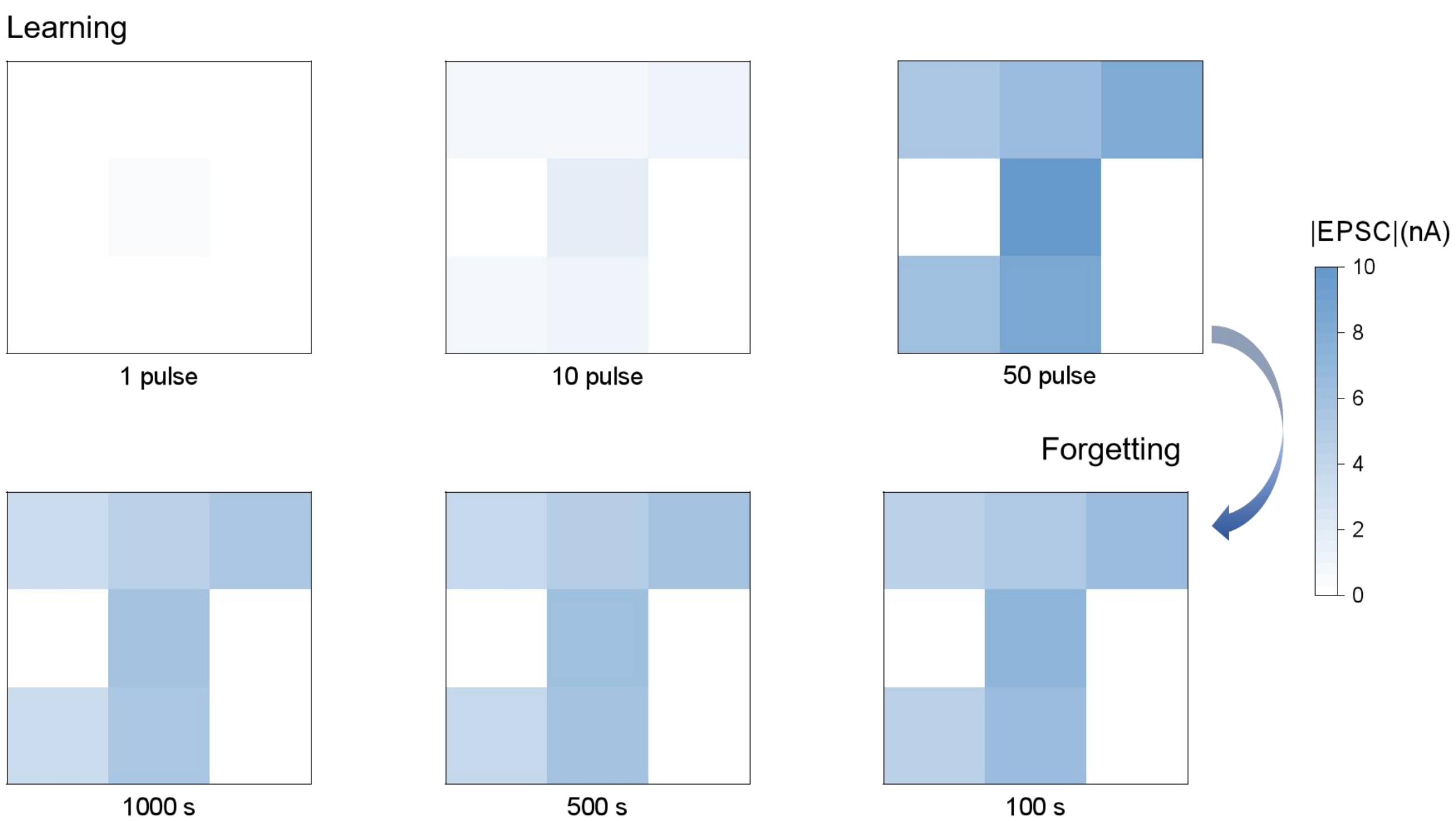

Supplementary Fig. 68 | Learning and forgetting processes of Al_2O_3/PAA-based memory devices stimulated by continuous light pulses (450 nm, 2.04 mW cm^{-2}, 1 s). The device retains well-resolved current levels throughout the 1000 s forgetting phase.

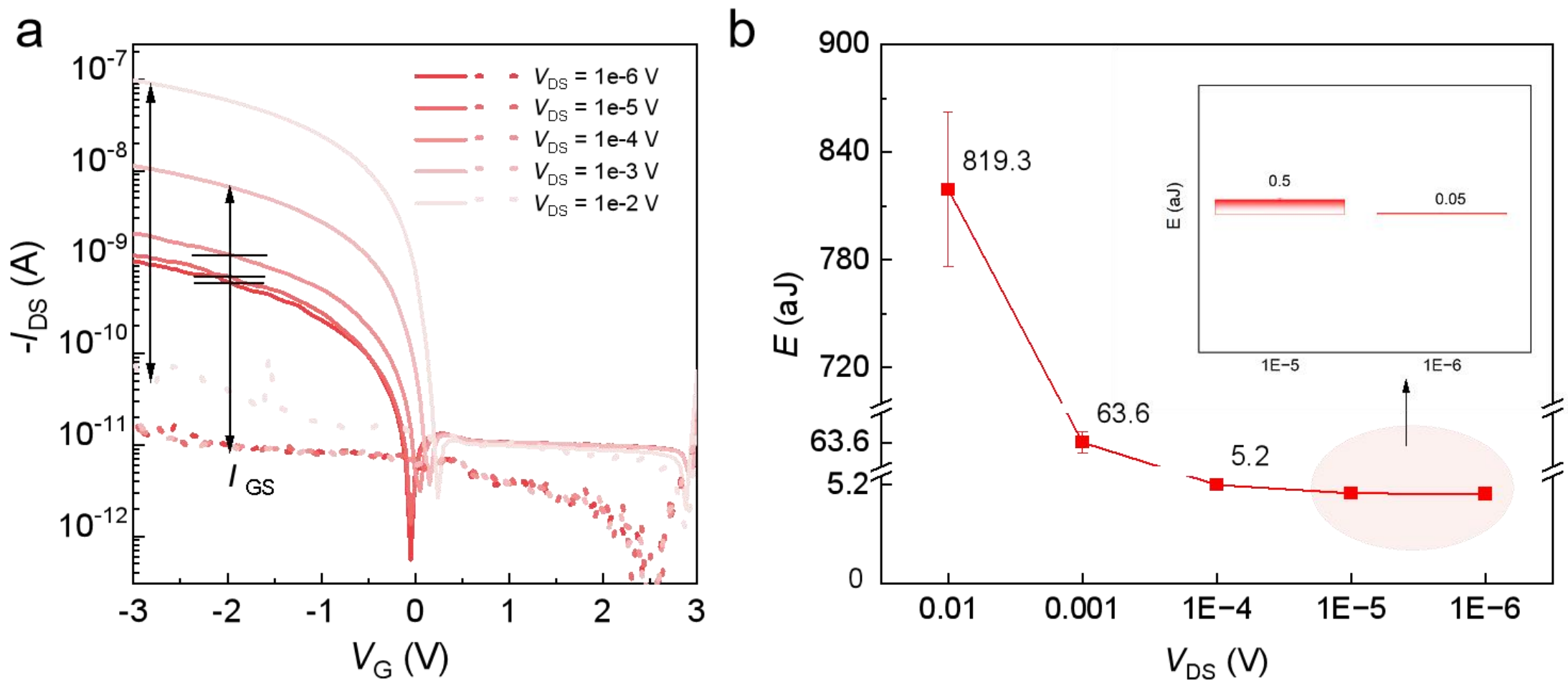

Supplementary Fig. 69 | Operating-voltage dependence of transfer characteristics and energy consumption for flexible devices. a, Transfer curves at different bias voltages. **b,** Energy consumption at different bias voltages.

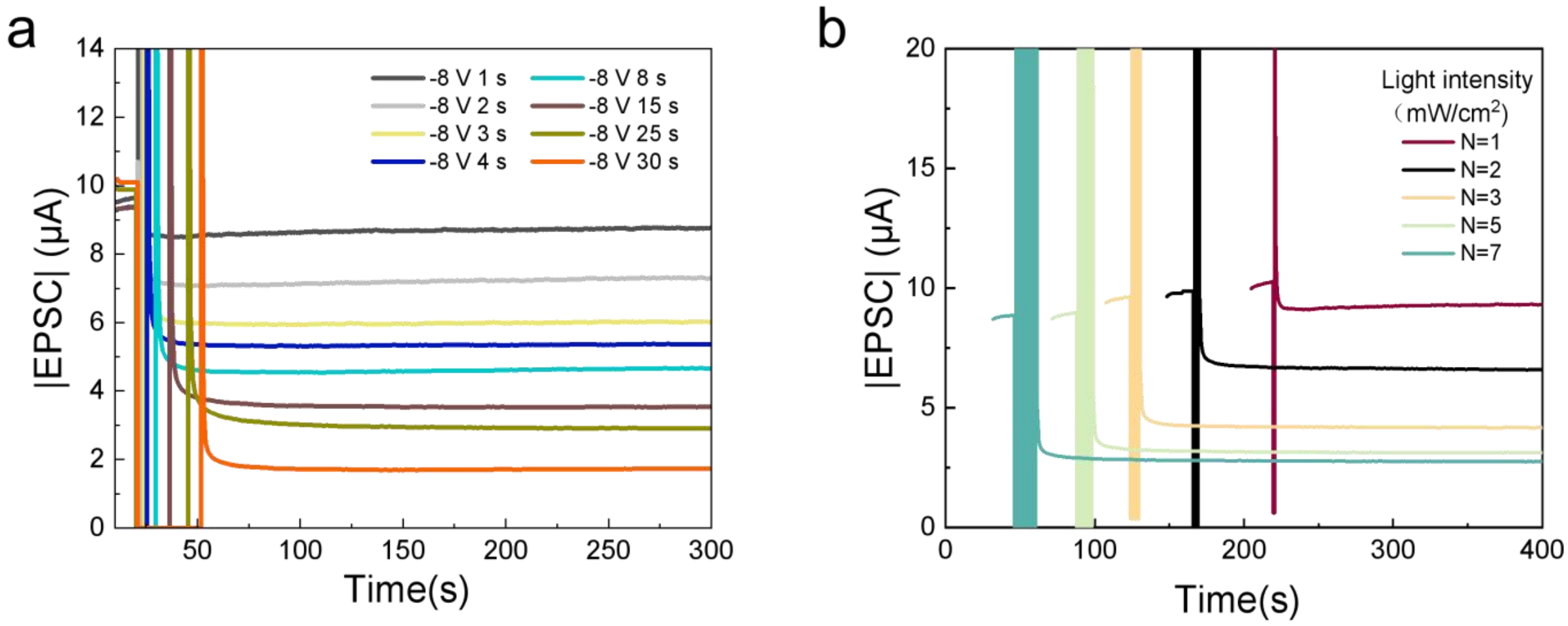

Supplementary Fig. 70 | IPSC of Al_2O_3/PAA-based memory devices measured at V_{DS} = –5 V and V_{GS} = –2 V. a, Response to increasing pulse duration. **b,** Response to increasing number of pulses. All data were acquired under the same bias conditions.

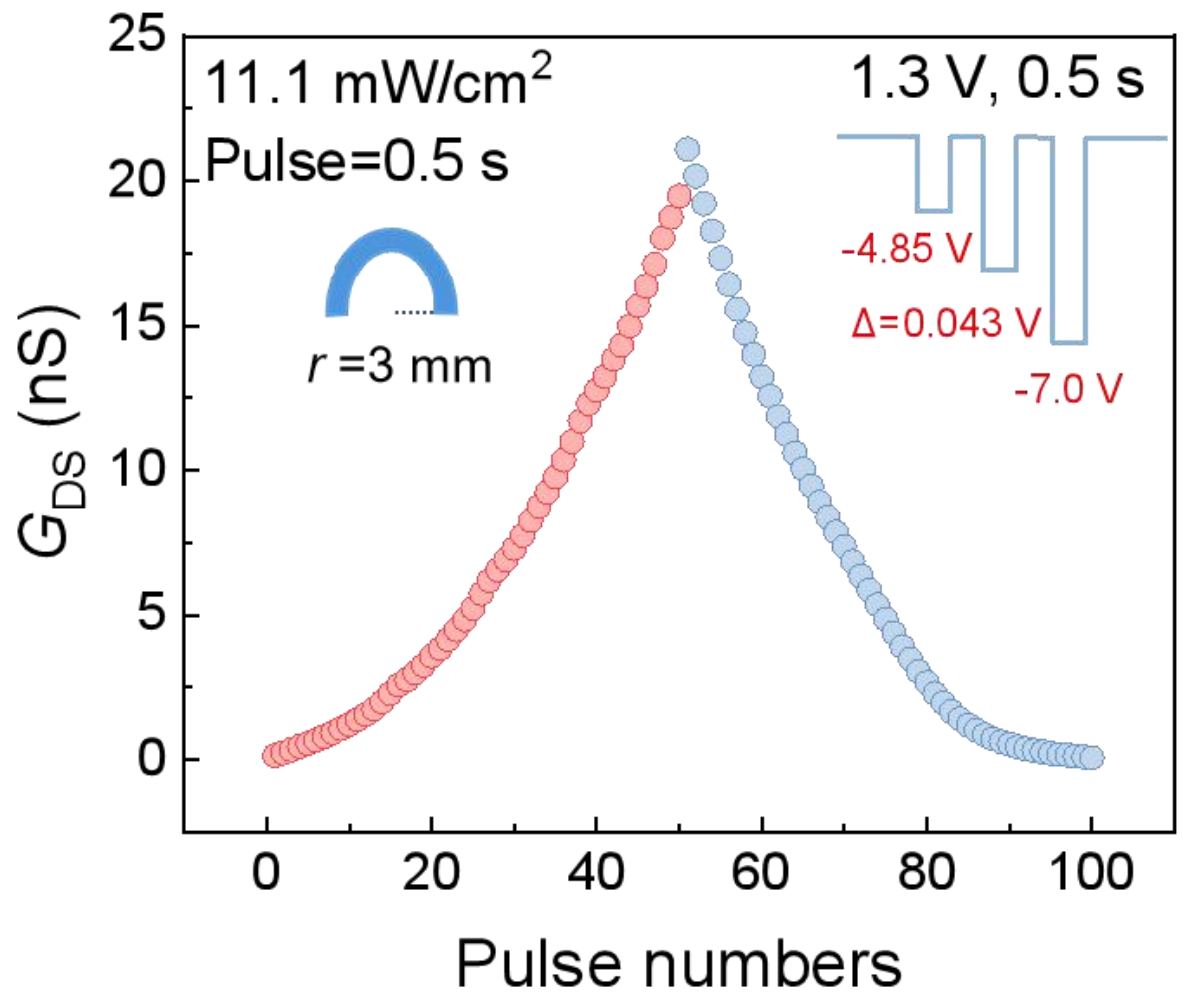

Supplementary Fig. 71 | Optical enhancement and electrical suppression behaviour of flexible OTFTs based on 30 nm Al_2O_3 /180 nm PAA films at 3 mm bending radius. The application of 50 optical pulses and 50 electrical pulses respectively induces photocurrent enhancement and voltage suppression, thereby demonstrating robust LTP and LTD behavior.

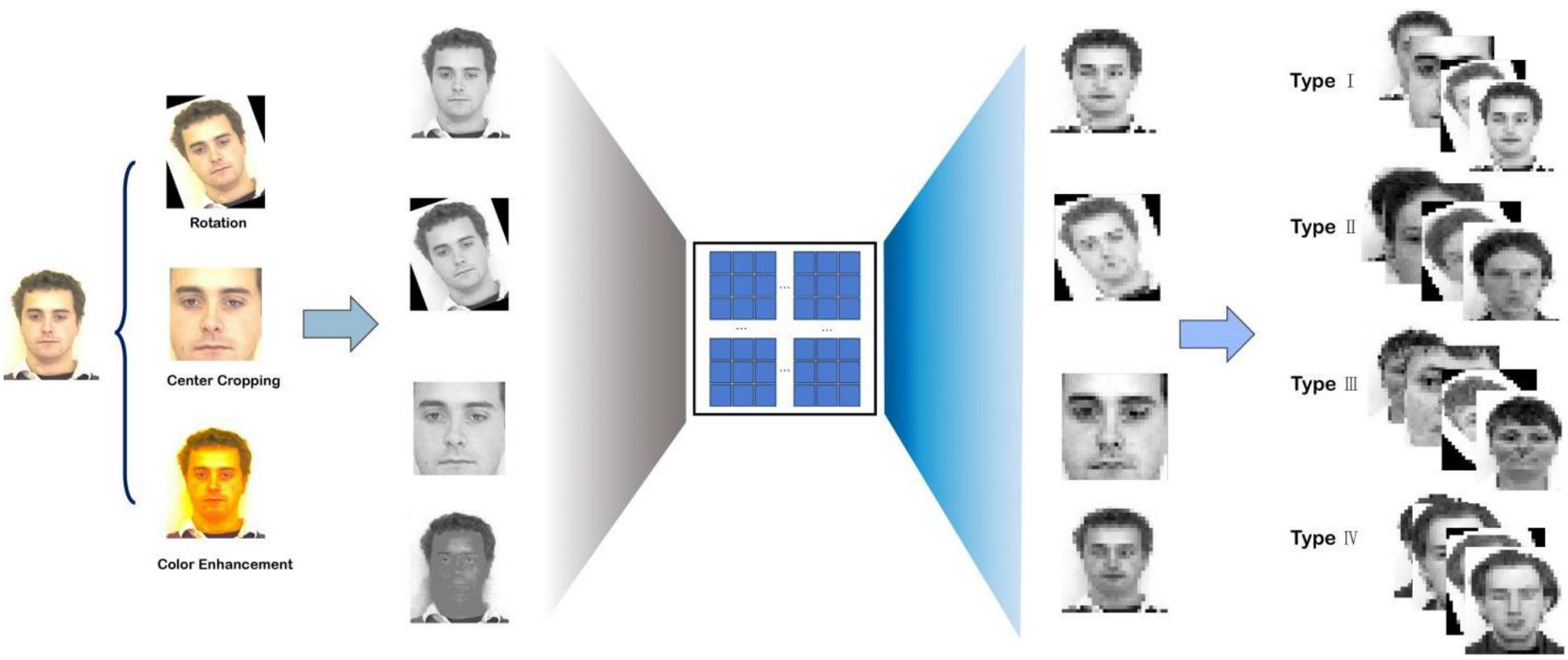

Supplementary Fig. 72 | An array of neuromorphic devices applied to the preprocessing step of a face dataset senses the storage process of a face image and demonstrates the image enhancement process. The database is provided by Yale University and is stated on the official Yale Face Database webpage to be publicly available for non-commercial use.

Supplementary Table 1: Electrical characteristics of transistors fabricated with different dielectric layers (C10-DNTT Semiconductor)

**Dielectric Layer Type**	**μ (cm^2 V^{-1} s^{-1}) @ f = 20 Hz**	**V_{TH} (V)**	**SS(V dec^{-1})**
30nm PAA+30nm Al_2O_3	4.01	1.25	1.32
90nm PAA+30nm Al_2O_3	4.13	2.19	1.05
120nm PAA 30nm Al_2O_3	5.73	2.23	1.15
180nm PAA 30nm Al_2O_3	7.97	2.34	1.12
30nm Al_2O_3	2.58	0.06	0.63
30nm Al_2O_3+90nm PAA	12.2	0.49	0.55
30nm Al_2O_3+120nm PAA	17.8	0.38	0.56
**30nm Al_2O_3+180nm PAA**	**20.2**	**0.14**	**0.58**
10nm Al_2O_3+180nm PAA	15.6	0.08	0.59

Supplementary Table 2: Fabrication of transistor memory windows and retention times using different dielectric layers (programming at a voltage of -20 V for 1 second, C10-DNTT Semiconductor)

**Dielectric Layer Type**	**ΔV_{TH} (V) (-20 V, 1 s)**	**Sustain time (s)**
30nm PAA/30nm Al_2O_3	12.8	$> 10^4$
90nm PAA/30nm Al_2O_3	12.4	$\approx 10^3$
120nm PAA/30nm Al_2O_3	11.9	$\approx 10^3$
180nm PAA/30nm Al_2O_3	11.7	$\approx 10^3$
30nm Al_2O_3	11.2	$> 10^4$
30nm Al_2O_3/90nm PAA	10.6	$> 10^4$
30nm Al_2O_3/120nm PAA	9.99	$> 10^4$
**30nm Al_2O_3/180nm PAA**	**9.85**	**$> 10^4$**
10nm Al_2O_3/180nm PAA	15.6	$\approx 10^3$

Supplementary Table 3: Performance comparison of representative non-volatile organic/2D optoelectronic memory devices.

**Device Type**	**Semiconductor**	**Dielectric Layer**	**Operating Voltage**	**Mobility ($cm^2V^{-1}s^{-1}$)**	**Retention Time**	**Optical Response Speed**	**Electrical Response Speed**	**Ref.**
FET	$ReSe_2$	hBN / SiO_2	0.5 V	—	>5,000 s (~3 months)	0.5 s	~1 ms	1
Fe-OLET	C8-BTBT	P(VDF-TrFE) / PMMA	−40 V	8.1	>10^4 s	—	0.5 s	2
OHTM	Pentacene / P13 / Pentacene	PVP / SiO_2	−30 V	0.23	>10^4 s (extrapolated to 10 yr)	—	1 s	3
FET	Pentacene	SiO_2+ PPyMA/TCNQ	−40 V	~0.01	>10^4 s	50 ms	1 s	4
FET	PDPP4T + p-NO_2-HABI	SiO_2	−80 V	0.4	>10^4 s	1 ms	—	5
FET	$(POE)_2SnI_4$	SiO_2 + CL-PVP	−40 V	0.3	10^4 s	50 ms	5 s	6
OFET	HBP-H	SiO_2	−10 V	0.078	12 h	0.4 s	10 s	7
OFET	C8-BTBT + C8-PTCDI	FPS	−2 V	13.3	10^4 s (extrapolated to 30 yr)	300 ms	1 ms	8
OFET	BBTNDT	SiO_2 / Al_2O_3	−80 V	7.7	>$2×10^4$ s	10 s	10 s	9
OECT	PTBT-p	EMIMTFSI: PVDF-HFP	−0.8 V	—	>10^4 s	0.4 s	200 ns	10
OFET	Pentacene	SiO_2	−5 V	0.03	>10^5 s	1 s	5 s	11
OFET	Pentacene/BN-1	PMMA / SiO_2	-10 V	1.7	>10^4 s	1 s	1 s	12
OPT	BTBT	PMMA / SiO_2	−30 V	1.31	>10^4 s	1 s	10 μs	13
OFET	PTCDI-C13	pC1D1 / pV3D3	2.5 V	—	>10^5 s	—	100 μs	14
FET	IGZO	Ga_2O_3/Al_2O_3	−0.5 V	—	>72 h	50 ms	1 s	15
FET	IGZO	SiO_2	0.5 V	1.32	—	50 ms	—	16
OTFT	P3HT / DAE	SiO_2 / PMMA+PVP	−10 V	0.01	>$4.3×10^7$ s	3 ns	—	17
OFET	Pentacene	SiO_2 + PAH	−40 V	0.013	10^4 s	—	10 μs	18
OFET	N2200 : TIPS-PEN	PS-brush	20 V	0.12	10^4 s (extrapolated to 10 yr)	—	1 μs	19
OFET	Pentacene	HfO_2 + DAE SAM	−0.1 V	0.7	>$6×10^5$ s	—	1 s	20
OTFT (This work)	C10-DNTT	Al_2O_3 / PAA	−5 V	22.65	>$5×10^4$ s (extrapolated to 10 yr)	30 μs	Response: 130 ns Storage: 10 μs	This work

Supplementary Note 1:

In this paper, an image-aware computational recognition system was evaluated through NeuroSim-based simulations using experimentally measured/fitted conductance parameters of the synaptic devices. The simulations were intended to assess the potential system-level inference performance under device-realistic conductance-update constraints. The MNIST, gesture, and Yale face recognition results were obtained from simulations rather than from inference directly performed on a physical synaptic array or device-in-the-loop measurements. In this study, a multilayer perceptron (MLP) neural network model was designed using the NeuroSim platform for simulating a multiclassification task. The network consists of an input layer, a hidden layer, and an output layer, where the input layer is not counted in the number of network layers. The MLP network is fully connected, with neuron nodes in each layer connected via synapses and transferring information through a weight matrix. The MLP network is set up as 784-100-10, i.e., there are 784 neurons in the input layer, 100 neurons in the hidden layer, and 10 neurons in the output layer that correspond to a 28×28 pixel image and 10 number categories, respectively.

The training process comprises two key steps: feedforward and backpropagation. In the feedforward stage, the input data is processed through the network using weighted summation and an activation function to generate the prediction result of the output layer. This result is then compared with the actual label to calculate the prediction error. In the backpropagation phase, the error information is passed backward, and the network weights are adjusted to minimize the prediction error. The stochastic gradient descent method is employed to update the weights during the training process.

During backpropagation, the error was propagated backward, and the network weights were updated using stochastic gradient descent. In the NeuroSim simulation, the calculated weight updates were mapped to equivalent LTP or LTD conductance-update steps

according to the experimentally fitted device characteristics. These conductance updates were numerically simulated in NeuroSim, and no physical write pulses were applied to a real synaptic array during the recognition simulations.

The variation of conductance with the number of pulses in the MLP simulation model is described using the following equation:

$$G_{\mathrm{LTP}} = B\left(1 - e^{\left(\frac{\mathrm{P}}{\mathrm{A}}\right)}\right) + G_{\min} \tag{6}$$

$$G_{\mathrm{LTD}} = - B\left(1 - e^{\left(\frac{\mathrm{P}-\mathrm{P}_{max}}{\mathrm{A}}\right)}\right) + G_{\max} \tag{7}$$

$$B = \frac{G_{\max} - G_{\min}}{1 - e^{\frac{-\mathrm{P}_{\max}}{\mathrm{A}}}} \tag{8}$$

The equations model the nonlinear conductance update. Here, G_{LTP} and G_{LTD} represent the conductance of LTP and LTD, respectively. Other parameters, $G_{\max}$, $G_{\min}$, and $P_{\max}$, are obtained directly from experiments and represent the maximum conductance, the minimum conductance, and the maximum number of pulses required to switch between the minimum and maximum conductance states, respectively. A, determined by the nonlinearity of the weight update, can be either positive or negative.

Data obtained from experiments and normalized by Pmax can be fitted to obtain A_{LTP} = -0.85 and A_{LTD} = -0.75. B, on the other hand, is a function of A within the range defined by $G_{\max}$, $G_{\min}$, and $P_{\max}$.

For all recognition tasks, the training and testing datasets were strictly separated. The testing samples/subjects were not used during training or parameter optimization. For the Yale face recognition task, data augmentation was applied only to the training set because of the limited number of available samples. The augmentation operations included image rotation, center cropping, and color-space enhancement. The testing set remained independent and was used only for final performance evaluation. Representative augmented images are shown in Supplementary Fig. 72.

Supplementary References

1.Tsai M.-Y. et al. A reconfigurable transistor and memory based on a two-dimensional heterostructure and photoinduced trapping. *Nat. Electron.* **6**, 755-764 (2023).

2.Xu M. et al. Nonvolatile Memory Organic Light-Emitting Transistors. *Adv. Mater.* **35**, 2307703 (2023).

3.Li W. et al. High‐Performance Nonvolatile Organic Field‐Effect Transistor Memory Based on Organic Semiconductor Heterostructures of Pentacene/P13/Pentacene as Both Charge Transport and Trapping Layers. *Adv. Sci.* **4**, 1700007 (2017).

4.Yang Y.F. et al. Highly Efficient Photo-Induced Recovery Conferred Using Charge-Transfer Supramolecular Electrets in Bistable Photonic Transistor Memory. *Adv. Funct. Mater.* **31**, 2102174 (2021).

5.Liu Y. et al. Photo-/thermal-responsive field‐effect transistor upon blending polymeric semiconductor with hexaarylbiimidazole toward photonically programmable and thermally erasable memory device. *Adv. Mater.* **31**, 1902576 (2019).

6.Zhang F. et al. Photoinduced Nonvolatile Memory Transistor Based on Lead-Free Perovskite Incorporating Fused π-Conjugated Organic Ligands. *Adv. Mater.* **36**, 2307326 (2024).

7.Wang Y. et al. Robust radical cations of hexabenzoperylene exhibiting high conductivity and enabling an organic nonvolatile optoelectronic memory. *J. Am. Chem. Soc.* **144**, 16612-16619 (2022).

8.Li D. et al. Insulating Electrets Converted from Organic Semiconductor for High‐Performance Transistors, Memories, and Artificial Synapses. *Adv. Funct. Mater.* **33**, 2305012 (2023).

9.Pei K. et al. A high‐performance optical memory array based on inhomogeneity of organic semiconductors. *Adv. Mater.* **30**, 1706647 (2018).

10.Wang S. et al. An organic electrochemical transistor for multi-modal sensing, memory and processing. *Nat. Electron.* **6**, 281-291 (2023).

11.Ke C.-Y. et al. High-performance aggregation-induced emission active poly (ether sulfone) s as polymeric electret for energy-saving genuine photonic transistor memory.

Chem. Eng. J. **457**, 141209 (2023).
12.Yu Y. et al. A BN-Doped U-Shaped Heteroacene as a Molecular Floating Gate for Ambipolar Charge Trapping Memory. *Angew. Chem. Int. Ed.* **62**, e202303335 (2023).
13.Shen J. et al. Electret Molecular Configuration Induced Programmable Photonic Memory for Advanced Logic Operation and Data Encryption. *Adv. Funct. Mater.* **33**, 2213341 (2023).
14.Choi, J. et al. Vertically stacked, low-voltage organic ternary logic circuits including nonvolatile floating-gate memory transistors. *Nat. Commun.* **13**, 2305 (2022).
15.Zhu R. et al. Non-volatile optoelectronic memory based on a photosensitive dielectric. *Nat. Commun.* **14**, 5396 (2023).
16.Kim J. et al. Metal-Oxide Heterojunction Optoelectronic Synapse and Multilevel Memory Devices Enabled by Broad Spectral Photocarrier Modulation. *Small* **19**, 2301186 (2023).
17.Leydecker T. et al. Flexible non-volatile optical memory thin-film transistor device with over 256 distinct levels based on an organic bicomponent blend. *Nat. Nanotechnol.* **11**, 769-775 (2016).
18.Kim B.J. et al. Organic field-effect transistor memory devices using discrete ferritin nanoparticle‐based gate dielectrics. *Small* **9**, 3784-3791 (2013).
19.Li W. et al. Solution-Processed Wide-Bandgap Organic Semiconductor Nanostructures Arrays for Nonvolatile Organic Field-Effect Transistor Memory. *Small* **14**, (2018).
20.Chen H. et al. Design of a photoactive hybrid bilayer dielectric for flexible nonvolatile organic memory transistors. *ACS Nano* **10**, 436-445 (2016).
