# Supplementary material for: Flexible organic thin-film transistors for all-in-one retinomorphic acceleration enabled by inorganic-organic heterogeneous dielectrics
Source: Nat Commun. 2026 Jul 27;17:9124. doi: 10.1038/s41467-026-76116-z (PMC13518895; doi:10.1038/s41467-026-76116-z)
Supplement: Supplementary file 2 [file 41467_2026_76116_MOESM2_ESM.pdf]

Description of Additional Supplementary Files:

Supplementary Movie 1 | Demonstration of biomimetic pupillary light reflex driven by the flexible OTFT device. This movie showcases an artificial pupil system constructed by integrating the flexible organic thin-film transistor with an external driving circuit. Upon exposure to a 50 μs light pulse (450 nm), the device rapidly transduces the optical signal into an electrical response, which is relayed through the external circuit to actuate pupil constriction. Under dark conditions, the pupil remains fully open. The movie illustrates the device's capability to mimic the rapid protective reflex of the human eye, achieving microsecond-temporal-resolution photodetection and demonstrating its potential for biomimetic vision systems.

Supplementary Movie 2 | Realtime multi-target face detection with occlusion tolerance using the FPGA-based hardware accelerator. This movie demonstrates the practical efficacy of the synaptic device- integrated hardware accelerator for real-time face detection in complex visual environments. The system, implemented on a Zynq SoC platform, accurately localizes and tracks multiple face targets even under challenging conditions, including occlusions (e.g., medical masks) and cluttered backgrounds with various interfering objects.
