# Supplementary material for: Flexible organic thin-film transistors for all-in-one retinomorphic acceleration enabled by inorganic-organic heterogeneous dielectrics
Source: Nat Commun. 2026 Jul 27;17:9124. doi: 10.1038/s41467-026-76116-z (PMC13518895; doi:10.1038/s41467-026-76116-z)
Supplement: Supplementary file 3 [file 41467_2026_76116_MOESM5_ESM.pdf]

nature portfolio

Peer Review File

Flexible Organic Thin-Film Transistors for All-in-One Retinomorphic Acceleration Enabled by Inorganic-Organic Heterogeneous Dielectrics

Corresponding Author: Professor Deyang Ji

This file contains all reviewer reports in order by version, followed by all author rebuttals in order by version.

Version 0:

Reviewer comments:

Reviewer #1

(Remarks to the Author)
This manuscript explores low-temperature (80°C) plasma-enhanced atomic layer deposition (LT-PEALD) and spin-coating to fabricate an inorganic-organic heterogeneous dielectric layer (Al2O3-PAA) and further flexible organic thin-film transistors for all-in-one retinomorphic acceleration. The resulting flexible device array exhibits a mean mobility of 22.65 (29.11) cm2 V-1 s-1, a rapid response time of 10 μs to 450 nm illumination, a stable charge storage time of over ten years, a write/erase endurance exceeding 10,000 cycles, and a programming speed of 130 ns. Integrated with a convolutional neural network hardware accelerator, the synaptic device achieves real-time face detection with good interference resistance. The authors may want to address the following comments before consideration for publication.
1. Could the authors provide a direct measurement of trap distribution or energy levels?

2. It is also helpful to include direct comparison/evidence to support the claim that the trade-off between mobility and trapping is solved by the heterostructure.

3. Besides the 450 nm illumination, how about the spectral response and the corresponding noise analysis?

4. Please consider including a table to benchmark the complete performance parameters of the reported heterogeneous dielectric layer and the resulting transistor against those in the literature to clearly demonstrate the advantages of this work.

5. The use of machine learning models often faces the challenges of parameter overfitting, making them difficult to apply in new data sets or new human subjects. Could the authors provide relevant validation and demonstration for the recognition of handwritten digits/gestures/faces from unseen human subjects?

6. Besides the demonstration using a Zynq SoC, is it possible to provide relevant demonstrations on in-sensor computing?

7. The energy efficiency claim probably does not include the peripheral circuits (which often consume the most power). If so, the implication in the practical applications should be discussed.

8. It is less convincing to use linear extrapolation for projecting a retention time exceeding ten years.

9. The polymer polarity results are a bit under-leveraged in the demonstrations/applications.

Reviewer #2

(Remarks to the Author)
This manuscript reports flexible organic thin-film transistors (OTFTs) employing an Al2O3/PAA heterogeneous dielectric to achieve integrated optical sensing, nonvolatile memory, and synaptic functions. The device-level performance, particularly the high carrier mobility, robust bending stability, and the implementation of LTP/LTD through synergistic photoelectric modulation—is noteworthy and potentially impactful for the flexible electronics community.
However, the central claims of the manuscript significantly exceed the provided experimental evidence. Several headline

performance metrics appear to conflate qualitative observations or calibrated simulations with rigorous, direct device validation. In its current form, the manuscript lacks the technical depth and empirical certainty required for Nature Communications.

Comment # 1: The manuscript states that the synaptic devices “physically execute the computation-intensive convolutional and fully connected layers.” However, Fig. 6b indicates that convolution, activation and pooling are performed by the Programmable Logic of the Zynq SoC, while the OTFT devices are not clearly shown to be directly interfaced with the FPGA data path. The role of the devices in the inference pipeline is therefore unclear. The authors should provide the physical array size, weight-to-conductance mapping, device-in-the-loop MAC/VMM results, ADC/DAC specifications, latency, energy per inference, and comparison with a pure FPGA/software baseline.

Comment # 2: Supporting Note S1 states that the MLP network was designed using NeuroSim for simulation, with experimentally fitted conductance parameters. Thus, the MNIST, gesture and Yale face results appear to be device-parameter-calibrated simulations, not inference performed on a physical synaptic array. Describing these results as “implemented with synaptic devices” is misleading unless supported by device-in-the-loop measurements.

Comment # 3: Figs. 4b, c show memory-window formation under 10 μs gate pulses, whereas Fig. 4d shows “tunable suppression behavior” under 5 μs, 1 μs and 130 ns pulses. This appears to reflect short-pulse synaptic inhibition rather than stable nonvolatile programming. No post-pulse transfer-curve shift, retention trace, read-current state or device statistics after a 130 ns pulse are provided. To claim 130 ns programming, the authors must show post-pulse memory states, retention data, terminal-level pulse waveforms and RC/parasitic analysis.

Comment # 4: Fig. 2g shows current variation at 10 μs readout intervals during a 50 μs optical pulse, which does not by itself establish a sub-10 μs response time. Supplementary Fig. 11 only shows optical stimulation down to 100 μs. The manuscript does not provide 10–90% rise/fall times, optical pulse edge verification, amplifier bandwidth or RC-time analysis. The authors should provide bandwidth-limited transient photocurrent measurements and multi-device statistics. Without these data, the “<10 μs response” claim should be removed from the Abstract or replaced with the directly measured timescale.

Comment # 5: Supplementary Table 2 shows that the selected 30 nm Al2O3/180 nm PAA stack has a memory window of 9.85 V, whereas the 30 nm PAA/30 nm Al2O3 stack reaches 12.8 V under the same condition. Thus, the selected structure should be framed as a mobility–memory trade-off, not as the structure with superior charge-storage capability. In addition, the proposed mechanism—oxygen-vacancy trapping, interfacial charge accumulation and polar-PAA stabilization—is mainly inferred from polarity–memory correlations and EPR on single-layer Al2O3. More direct evidence, such as KPFM/Kelvin probe, temperature-dependent retention, C–V analysis or Fowler–Nordheim analysis of the Al2O3/PAA stack, is needed.

Comment # 6: The headline mobility values are difficult to assess because Supplementary Table 1 reports mobility at 20 Hz, Supplementary Fig. 7b reports 130-device statistics at 0.1 Hz, and Supplementary Fig. 2 shows strong frequency dependence of Ci. The authors should specify which Ci value was used for each mobility extraction, recalculate mobility using frequency-appropriate capacitance, and provide TLM or four-probe measurements to rule out contact-resistance artefacts. In addition, the basis of D* > 1014 Jones should be stated explicitly. If it is derived from dark-current or shot-noise assumptions rather than direct NEP/noise measurements, it should be reported as extrapolated detectivity, since such estimates can overstate actual faint-light detection performance.

Version 1:

Reviewer comments:

Reviewer #1

(Remarks to the Author)
Thank the authors for their efforts to address the comments and the manuscript is now recommended for publication.

Reviewer #2

(Remarks to the Author)
The authors have adequately addressed all of the concerns I raised. Their responses are satisfactory, and I recommend this manuscript for publication.

Open Access This Peer Review File is licensed under a Creative Commons Attribution 4.0 International License, which permits use, sharing, adaptation, distribution and reproduction in any medium or format, as long as you give appropriate credit to the original author(s) and the source, provide a link to the Creative Commons license, and indicate if changes were made.
In cases where reviewers are anonymous, credit should be given to 'Anonymous Referee' and the source.
The images or other third party material in this Peer Review File are included in the article's Creative Commons license, unless indicated otherwise in a credit line to the material. If material is not included in the article's Creative Commons license and your intended use is not permitted by statutory regulation or exceeds the permitted use, you will need to obtain permission directly from the copyright holder.
To view a copy of this license, visit https://creativecommons.org/licenses/by/4.0/

Dear Editor and Reviewer:

Enclosed please find our revised manuscript entitled "**Flexible Organic Thin-Film Transistors for All-in-One Retinomorphic Acceleration Enabled by Inorganic-Organic Heterogeneous Dielectrics**" (Manuscript No. NCOMMS-26-026639-T). The constructive and insightful suggestions from the reviewers have greatly contributed to improving the manuscript. We have carefully revised the manuscript according to the reviewers' comments. Our detailed response and the changes made to the manuscript are provided on the next page point by point. The revised sections of the manuscript have been highlighted in red.

Sincerely yours,

Deyang Ji

Point-by-point response to the reviewers' comments

Reviewer #1 (Remarks to the Author):

This manuscript explores low-temperature (80°C) plasma-enhanced atomic layer deposition (LT-PEALD) and spin-coating to fabricate an inorganic-organic heterogeneous dielectric layer (Al_2O_3-PAA) and further flexible organic thin-film transistors for all-in-one retinomorphic acceleration. The resulting flexible device array exhibits a mean mobility of 22.65 (29.11) $cm^2\,V^{-1}\,s^{-1}$, a rapid response time of 10 μs to 450 nm illumination, a stable charge storage time of over ten years, a write/erase endurance exceeding 10,000 cycles, and a programming speed of 130 ns. Integrated with a convolutional neural network hardware accelerator, the synaptic device achieves real-time face detection with good interference resistance. The authors may want to address the following comments before consideration for publication.

Response: Thank you for the positive and constructive assessment of our work. In response to all the comments, we have performed additional experiments—including Kelvin probe force microscopy (KPFM) measurements, multi-wavelength photoresponse characterization, direct noise current measurements, and extended retention tests—and have thoroughly revised the manuscript to address each concern. Specifically, we have: (1) provided direct KPFM evidence for trap-induced Fermi-level shifts across different dielectric configurations; (2) extended the spectral response to 290 nm, 365 nm, and 515 nm with noise-corrected detectivity calculations based on directly measured noise current; (3) compiled a comprehensive performance comparison table to benchmark our device against the state of the art; (4) clarified the strict separation of training and testing datasets for all machine learning tasks; (5) clarified the scope of our in-sensor computing demonstration as an experimentally calibrated system-level evaluation; (6) explicitly acknowledged the limitations of our energy-efficiency analysis regarding peripheral-circuit overhead; (7) extended retention measurements to 50,000 s to support the linear extrapolation to ten years; and (8) strengthened the narrative connecting polymer polarity screening to the subsequent device applications. We believe these revisions have substantially improved the manuscript and we are grateful for your insightful suggestions.

Q1. Could the authors provide a direct measurement of trap distribution or energy levels?

A1. We fully agree that a direct measurement of trap distribution or energy levels would provide stronger evidence for the charge trapping mechanism in our Al_2O_3-PAA heterostructure. In our original manuscript, we demonstrated that applying a negative gate voltage pulse shifts the transfer curve negatively (**Fig. 3a)**, and that as the programming voltage gradient increases, the turn-on position exhibits a corresponding negative shift. (**Fig. 4b, 4c**). Furthermore, current-time (I-T) measurements showed a stepwise decrease in the readout current with increasing programming voltage (**Fig. 4a**), indicating a reduction of hole density in the channel. These observations confirmed the effective charge storage capability of our device and established a clear correlation between the memory window and the applied gate voltage.

To directly probe the trap-induced energy level shift, we have now performed Kelvin probe force microscopy (KPFM) measurements on the semiconductor surface before and after applying gate voltage pulses. The KPFM measurements were carried out using a conductive tip with a work function of ϕ_{tip}=5.1 eV (calibrated against HOPG). The contact potential difference V_{CPD} is given by:

$$V_{CPD}=\frac{\phi_{sample}-\phi_{tip}}{e} \qquad (1)$$

Where ϕ_{sample} is the work function of the semiconductor surface. The Fermi level of the sample is $E_{F,sample} = -\phi_{sample}$. Hence:

$$E_{F,sample}=-5.1eV-eV_{CPD} \qquad (2)$$

For a p-type semiconductor (C10-DNTT), the hole concentration is

$$p=N_v exp(\frac{E_V-E_F}{k_BT}) \qquad (3)$$

A decrease in E_F (i.e., E_F becomes more negative) corresponds to a reduction in hole concentration, because the Fermi level moves away from the valence band edge. Specifically, we measured the surface potential of the semiconductor layer under the following conditions: (i) without any gate voltage application, and (ii) after applying gate pulses of –10 V, –12 V, –15 V, –17 V, and –20 V for 1 s each (the pulse was removed before KPFM scanning). The results are summarized in **Supplementary Figs. 34 and 35**, These

KPFM results clearly show a monotonic increase in V_{CPD}(i.e., an increase in ϕ_{sample} and a more negative $E_{F,sample}$) with increasing programming voltage. According to the p-type carrier statistics, a more negative Fermi level indicates a significant reduction of hole density at the semiconductor surface-exactly as expected when holes are trapped in the dielectric layer and removed from the channel. The stepwise increase in surface potential also corroborates the multilevel memory behavior observed in our electrical measurements. We have also added relevant statements on page 11 of the revised manuscript.

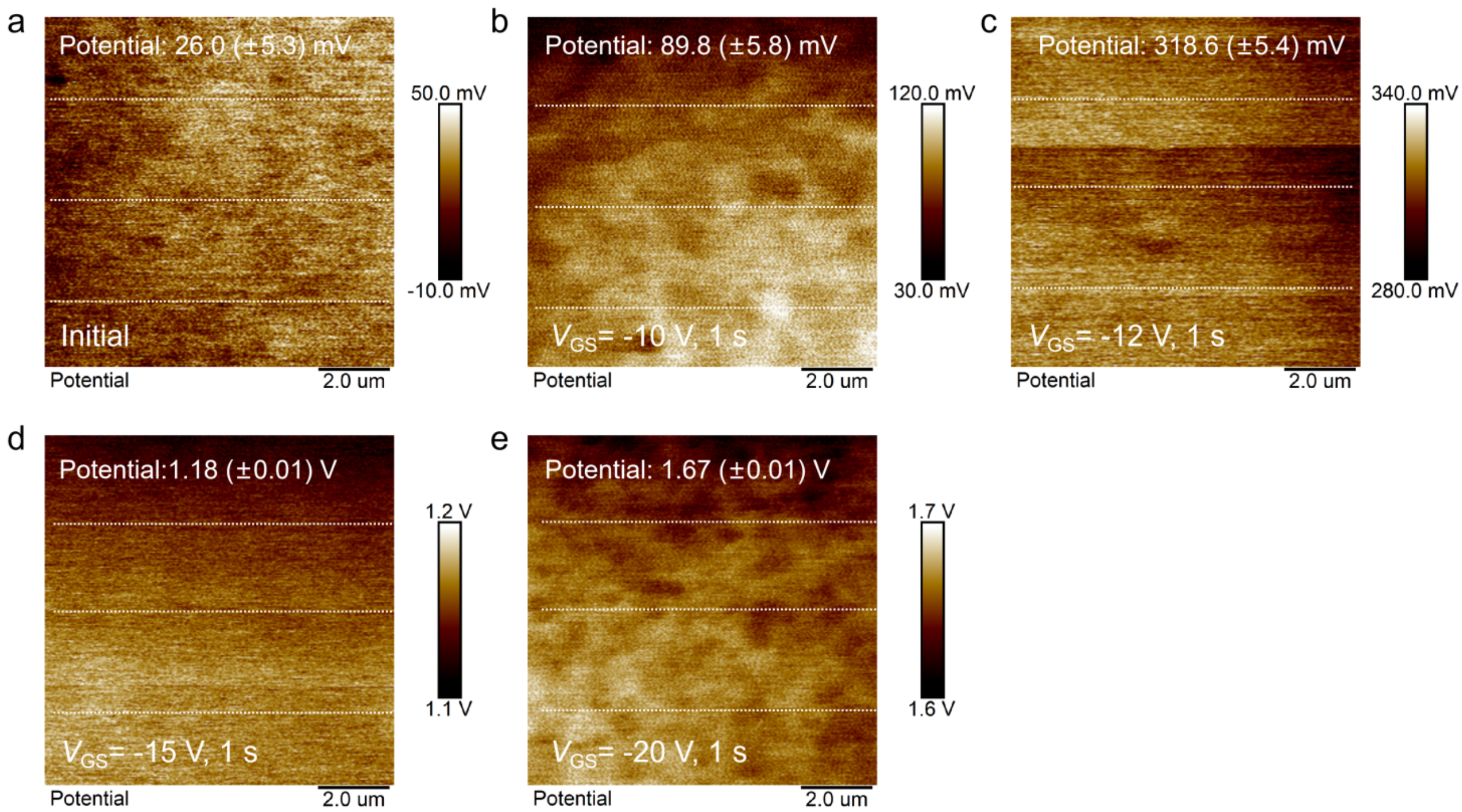

Supplementary Fig. 34 | Surface potential mapping of the transistor channel for a device with a 30 nm Al_2O_3/180 nm PAA dielectric layer under different gate pulse conditions. a, KPFM surface potential image of the semiconductor channel in the pristine state (without gate voltage application). **b–e**, KPFM surface potential images after applying gate pulses of –10 V (**b**), –12 V (**c**), –15 V (**d**), and –20 V (**e**) for 1 s each. The pulse was removed prior to KPFM scanning.

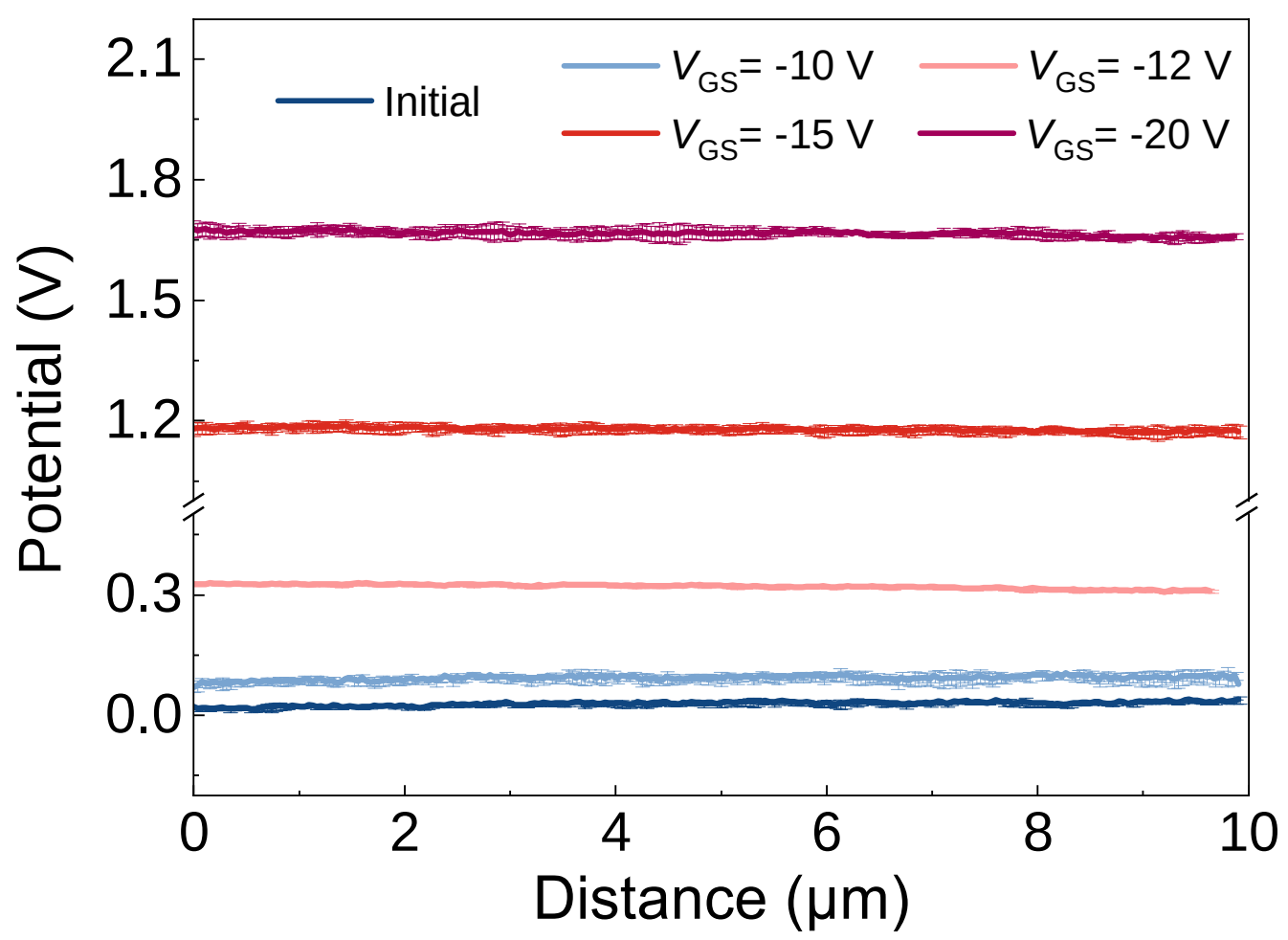

Supplementary Fig. 35 | Statistical summary of the average surface potential for transistors with an 30 nm Al_2O_3/180 nm PAA dielectric layer under various gate pre-bias conditions. The data points represent the mean surface potential values extracted from three distinct measurement positions for each condition; error bars denote the standard deviation.

Page 11: … The Al_2O_3/PAA heterostructure (Supplementary Figs. 34 and 35) exhibits a similar stepwise increase (from 26.0 mV to 1.67 V), confirming that it inherits the strong trapping capability of Al_2O_3. These KPFM results provide direct evidence of trap-induced Fermi-level shifts and corroborate the charge storage mechanism inferred from the electrical measurements...

Q2. It is also helpful to include direct comparison/evidence to support the claim that the trade-off between mobility and trapping is solved by the heterostructure.

A2. We agree that direct, multi-dimensional evidence is necessary to robustly support our claim that the Al_2O_3-PAA heterostructure successfully resolves the intrinsic trade-off between high carrier mobility and efficient charge trapping. In response, we have systematically compared transistors based on three dielectric configurations: single PAA, single Al_2O_3, and the Al_2O_3/PAA heterostructure, from both electrical performance and surface potential perspectives.

(1). Electrical performance comparison

Mobility: As shown in **Supplementary Figs. 4f, 4h, 4i and 6f, 6h, 6i**, the single PAA

dielectric yields higher carrier mobility than single Al_2O_3, which is beneficial for fast device response. However, due to the inherent aging tendency of polymer dielectrics, the operational stability of single PAA devices is unsatisfactory. In contrast, the Al_2O_3/PAA heterostructure exhibits a moderately high mobility (over 20 $cm^2 V^{-1} s^{-1}$) together with excellent stability. By systematically tuning the thickness and deposition sequence (**Supplementary Figs. 4-6**), we identified the 30 nm Al_2O_3-180 nm PAA stack as the optimal configuration.

Memory (trapping): As shown in **Supplementary Figs. 19 and 20**, single PAA devices show a negligible memory window (< 0.1 V) after a –20 V, 1 s programming pulse, with an on/off ratio below 10, indicating almost no non-volatile storage capability. In sharp contrast, single Al_2O_3 devices under the same condition exhibit a large memory window (> 11 V) and a stable retention exceeding 10^4 s with an on/off ratio > 10^7, demonstrating excellent charge trapping ability.

Heterostructure performance: As shown in **Supplementary Figs. 29 and 30,** the Al_2O_3/PAA heterostructure (30 nm Al_2O_3/180 nm PAA) achieves a memory window and retention time comparable to those of single Al_2O_3, while simultaneously maintaining the high carrier mobility that is unattainable with Al_2O_3 alone.

(2). Direct evidence from KPFM measurements

To further corroborate the above electrical observations, we performed KPFM measurements to directly monitor the surface potential (work function) changes under different programming conditions, which reflect the extent of charge trapping. For single PAA devices (**Supplementary Fig. 33**): Before programming, the surface potential is 36.1 (±4.1) mV. After applying a –20 V, 1 s gate pulse, the surface potential becomes 36.7 (±5.8) mV. The negligible change confirms that PAA alone has very weak charge trapping capability. For single Al_2O_3 devices (**Supplementary Figs. 31 and 32**): Before programming, the surface potential is 22.7 (±2.8) mV. After applying gate pulses of –10 V, –12 V, –15 V, and –20 V for 1 s each, the surface potentials increase stepwise to 77.0 (±5.7) mV, 398.3 (±4.9) mV, 1.22 (±0.01) V, and 1.70 (±0.03) V, respectively. This monotonic and substantial increase clearly demonstrates strong and tunable charge trapping in Al_2O_3. For the Al_2O_3/PAA heterostructure (as previously shown in response to Comment #1): A similar

stepwise increase in surface potential was observed (from 26.0 mV up to 1.67 V under –20 V programming), confirming that the heterostructure retains the excellent trapping capability of Al_2O_3.

Taken together, the electrical characterizations (mobility, memory window, retention) and direct KPFM evidence collectively demonstrate that the Al_2O_3/PAA heterostructure successfully overcomes the traditional mobility-trapping trade-off: It preserves the high carrier mobility enabled by the PAA layer (with its self-patterned nanogrooves), and inherits the strong and non-volatile charge trapping capability from the Al_2O_3 layer (deep-level defects). We have incorporated these new data and discussions into the revised manuscript Page 11 to make our claim more rigorous and convincing.

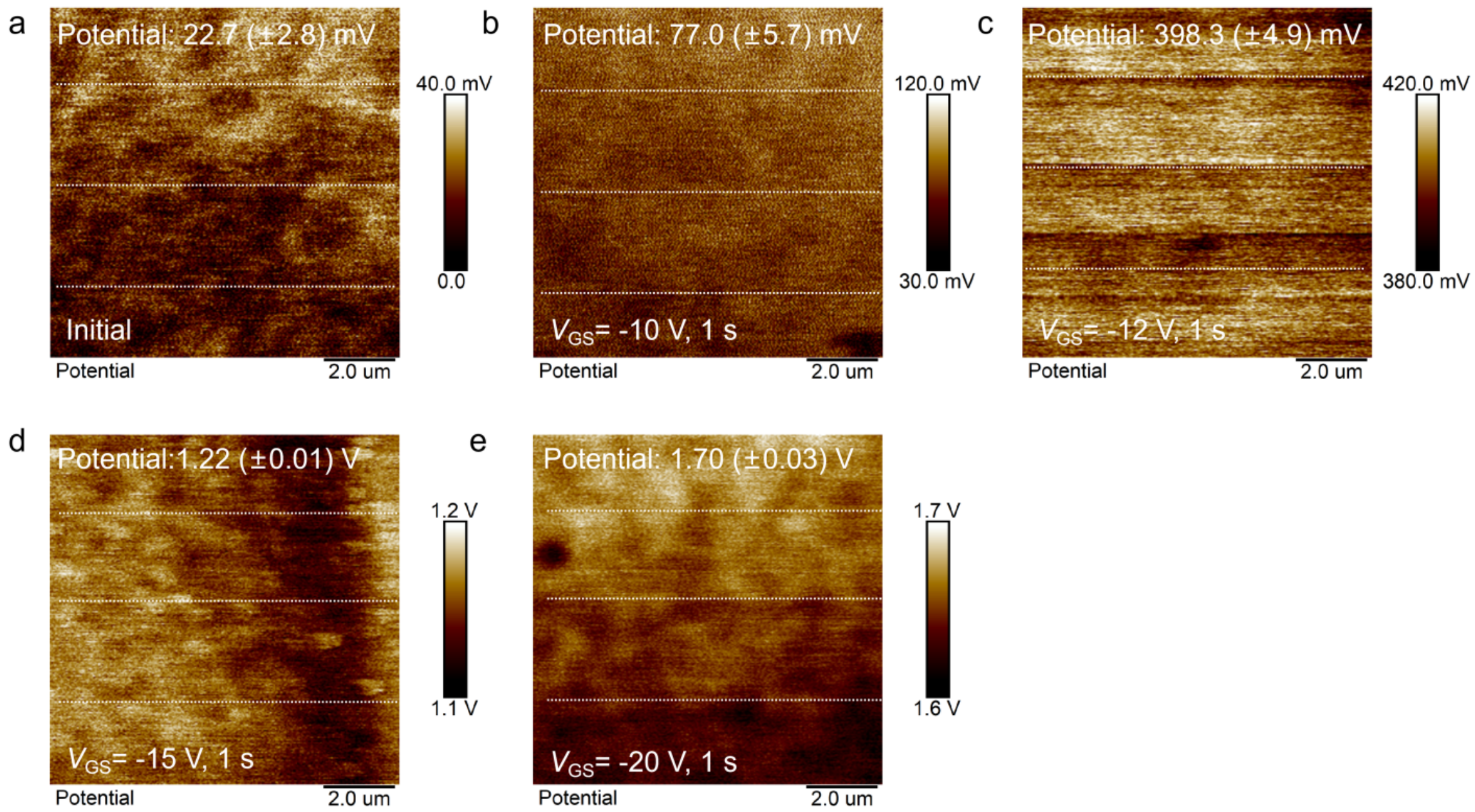

Supplementary Fig. 31 | Surface potential mapping of the transistor channel for a device with a 30 nm Al_2O_3 dielectric layer under different gate pulse conditions. a, KPFM surface potential image of the semiconductor channel in the pristine state (without gate voltage application). **b–e**, KPFM surface potential images after applying gate pulses of –10 V (**b**), –12 V (**c**), –15 V (**d**), and –20 V (**e**) for 1 s each. The pulse was removed prior to KPFM scanning.

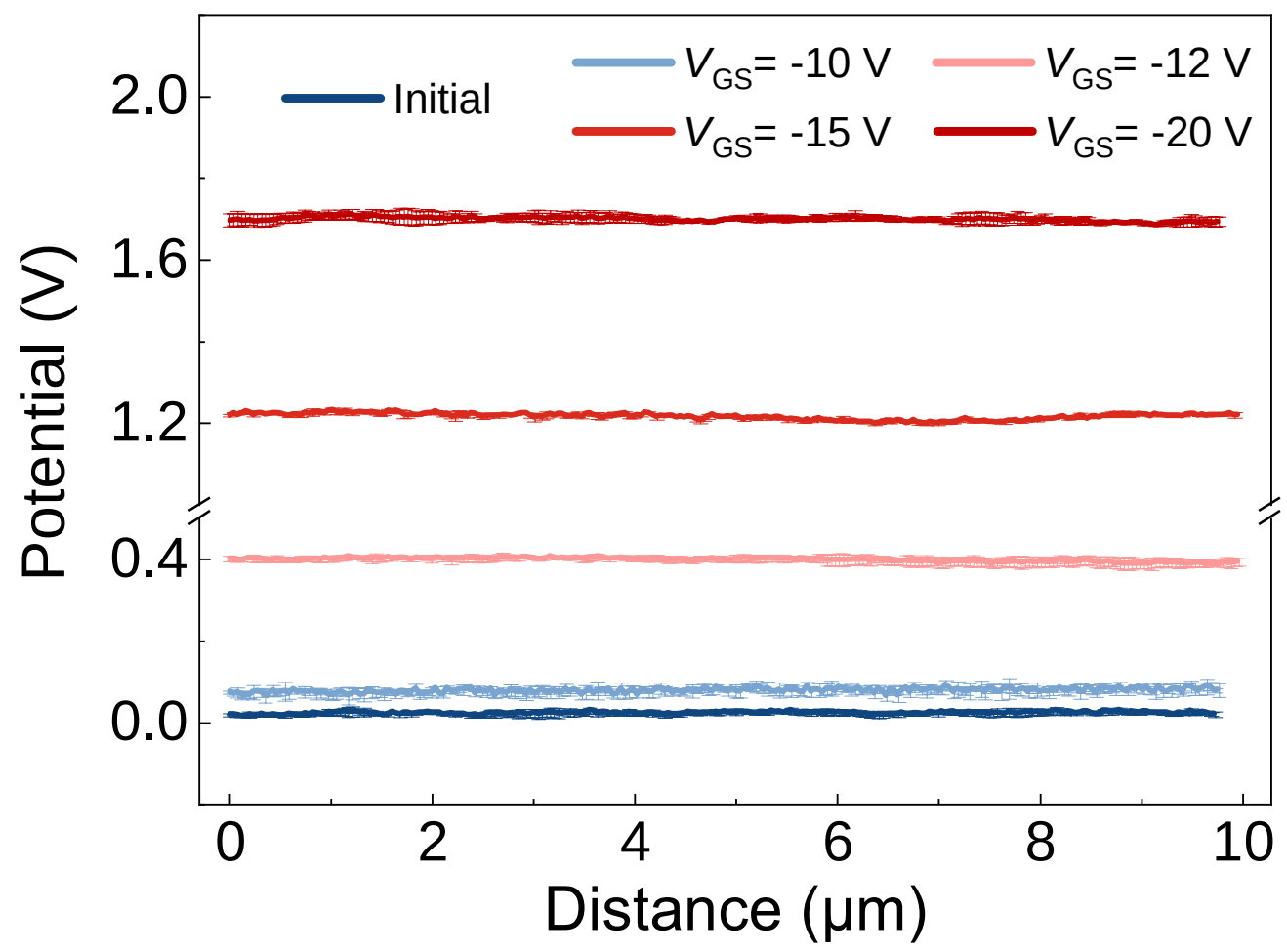

Supplementary Fig. 32 | Statistical summary of the average surface potential for transistors with an Al_2O_3 dielectric layer under various gate pre-bias conditions. The data points represent the mean surface potential values extracted from three distinct measurement positions for each condition; error bars denote the standard deviation.

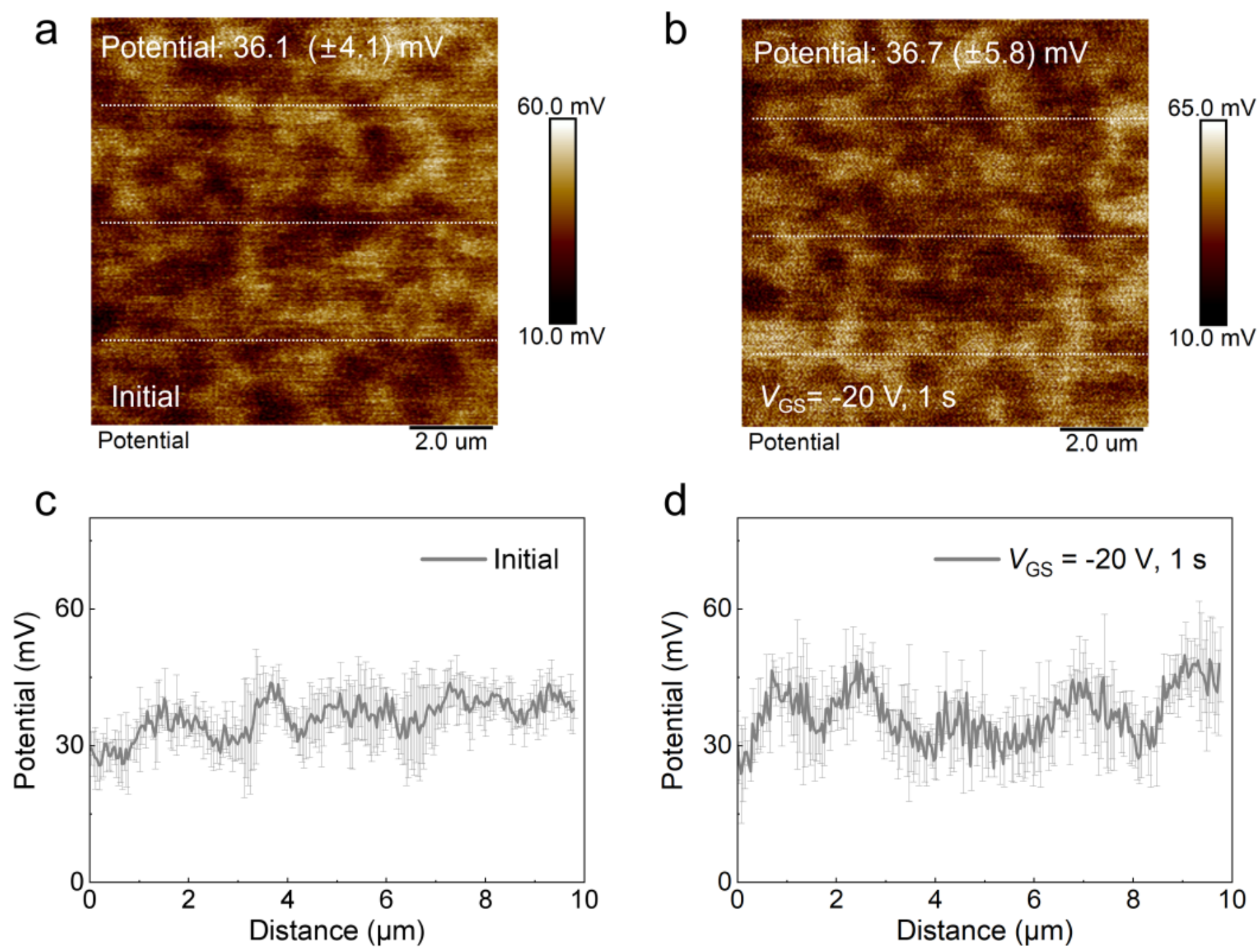

Supplementary Fig. 33 | Surface potential characteristics of transistors based on a 180 nm PAA dielectric layer before and after gate voltage pulsing. a, KPFM surface potential mapping of the semiconductor channel before gate voltage application. **b**, KPFM surface potential mapping after applying a –20 V, 1 s gate pulse. **c**, Statistical distribution of the initial surface potential (mean value: 36.1 ± 4.1 mV). **d**, Statistical distribution of the surface potential after pulsing (mean value: 36.7 ± 5.8

mV). The negligible change confirms that the single PAA layer exhibits very weak charge trapping capability.

Page 11: ...To directly probe the trap-induced energy level shift and the charge trapping role of oxygen vacancies, we performed Kelvin probe force microscopy (KPFM) on devices with different dielectric configurations. For single Al_2O_3 devices (Supplementary Figs. 31 and 32), the surface potential increases stepwise from 22.7 mV to 1.70 V as the programming voltage increases from –10 V to –20 V, confirming strong charge trapping; in contrast, single PAA devices (Supplementary Figs. 33) show negligible change (from 36.1 mV to 36.7 mV) under the same conditions. The Al_2O_3/PAA heterostructure (Supplementary Figs. 34 and 35) exhibits a similar stepwise increase (from 26.0 mV to 1.67 V), confirming that it inherits the strong trapping capability of Al_2O_3. These KPFM results provide direct evidence of trap-induced Fermi-level shifts and corroborate the charge storage mechanism inferred from the electrical measurements.

Q3. Besides the 450 nm illumination, how about the spectral response and the corresponding noise analysis?

A3. We fully agree that a comprehensive spectral response characterization, together with rigorous noise analysis, is essential to fully evaluate the photosensing capability of our devices. In response, we have performed additional measurements and analyses as detailed below.

(i) **Noise current measurement and specific detectivity calculation:** To accurately assess the detection limit of our devices, we directly measured the noise current (i_n) under dark conditions. The noise current was acquired by continuously sampling the drain current in the dark at a fixed bias (V_{DS}= -5 V, V_{GS}=0V), and the root-mean-square (RMS) value of the current fluctuation was taken as i_n. A representative noise spectrum is shown in **Supplementary Fig. 13**. Based on the measured noise current, we calculated the noise-equivalent power (NEP) and specific detectivity (D*) using the following standard formulae:

$$NEP=\frac{i_n}{R} \quad (4)$$

$$D^* = \frac{\sqrt{A}}{NEP} \quad (5)$$

where: i_n is the RMS noise current (A), R is the responsivity (A W^{-1}), A is the effective channel area (cm^2). The measured dark-state noise current of our device is i_n =2.05×10^{-13}A. Using this value, we recalculated the specific detectivity for all measured wavelengths to ensure a rigorous and consistent comparison.

(ii) Multi-band detection: Beyond the previously reported 450 nm response, we have now systematically characterized the photoresponse of our devices under monochromatic illumination at 290 nm, 365 nm, and 515 nm. As shown in **Supplementary Fig. 14** the device exhibits clear and reproducible photoresponses at all tested wavelengths, confirming its broadband detection capability. The corresponding *P* and *R* at 290 nm, 365 nm, and 515 nm have been calculated, and the complete results—including the noise-corrected *D**. Across the entire measured spectral range, the device maintains high *P* and *R* values, and the detectivity figures derived from directly measured noise current further corroborate its robust broadband performance.

Furthermore, to evaluate the optical neuromorphic functionality beyond 450 nm, we performed multi-pulse photoresponse tests at 290 nm, 365 nm, and 515 nm. As illustrated in **Supplementary Fig. 66**, the device shows reproducible excitatory postsynaptic current (EPSC) responses under 10, 20, 30, 40, and 50 consecutive light pulses at each wavelength. The EPSC amplitude increases progressively with the pulse number, clearly demonstrating a transition from short-term plasticity (STP) to long-term plasticity (LTP) across all tested spectral bands. These results confirm that the multi-wavelength optical modulation is a general and robust feature of our device, not limited to 450 nm excitation.

In summary, we have directly measured the dark-state noise current, extended the spectral response to multiple wavelengths with calculated detectivities, and verified multi-pulse synaptic plasticity behavior across the entire tested range. All these new data have been incorporated into the revised manuscript. (Page 9 and Page 15)

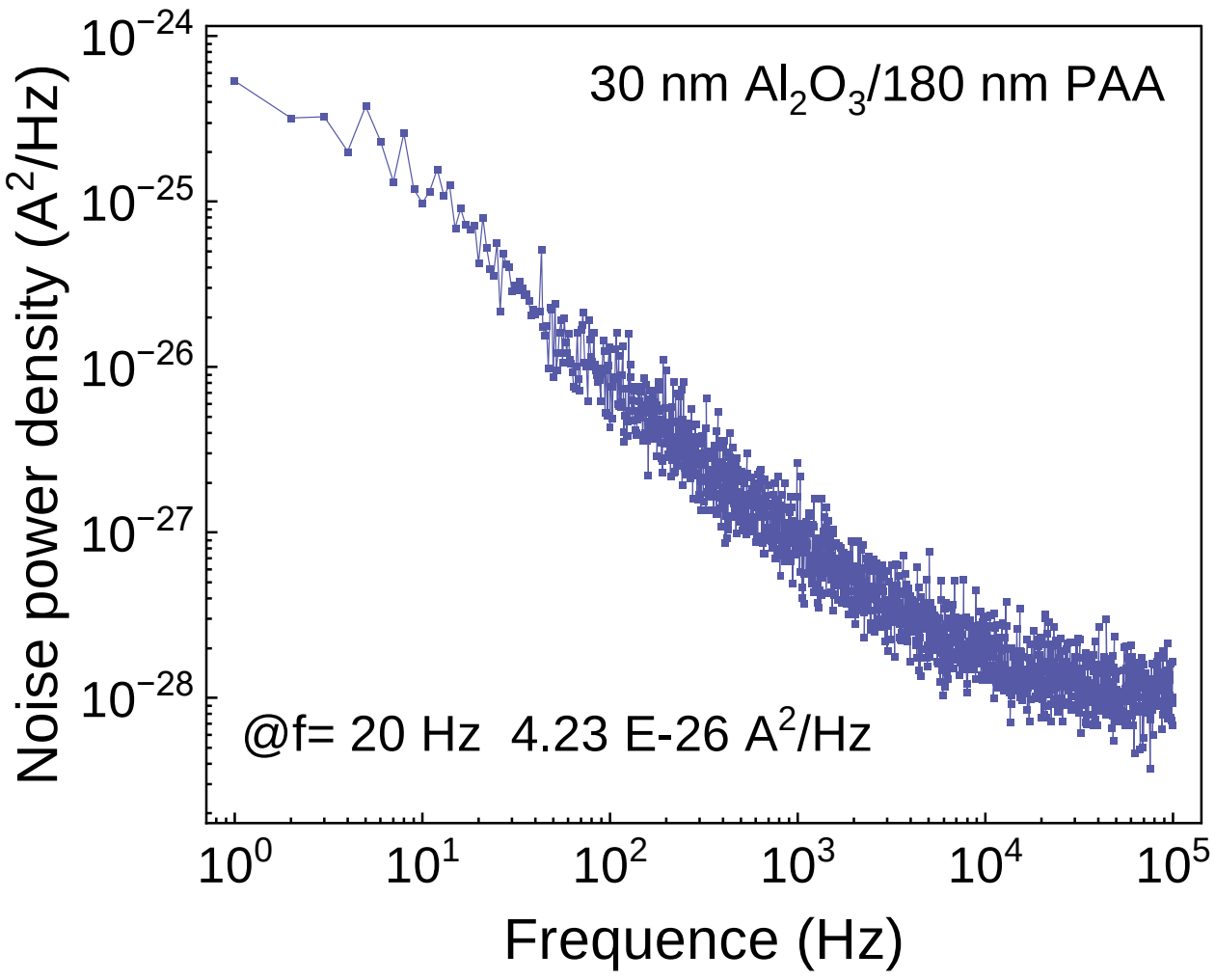

Supplementary Fig. 13 | Frequency-dependent noise current spectral density of the device measured under dark conditions. The noise power spectral density (S_i) is plotted as a function of frequency. The measurement was performed at a fixed bias of V_{DS}= -5 V and V_{GS}= 0 V in the dark. The noise current (i_n) used for detectivity calculations was extracted at 20 Hz, yielding a value of i_n= 2.05×10^{-13} A. (corresponding to S_n=i_n^2=4.23×10^{-26} A^2/Hz)

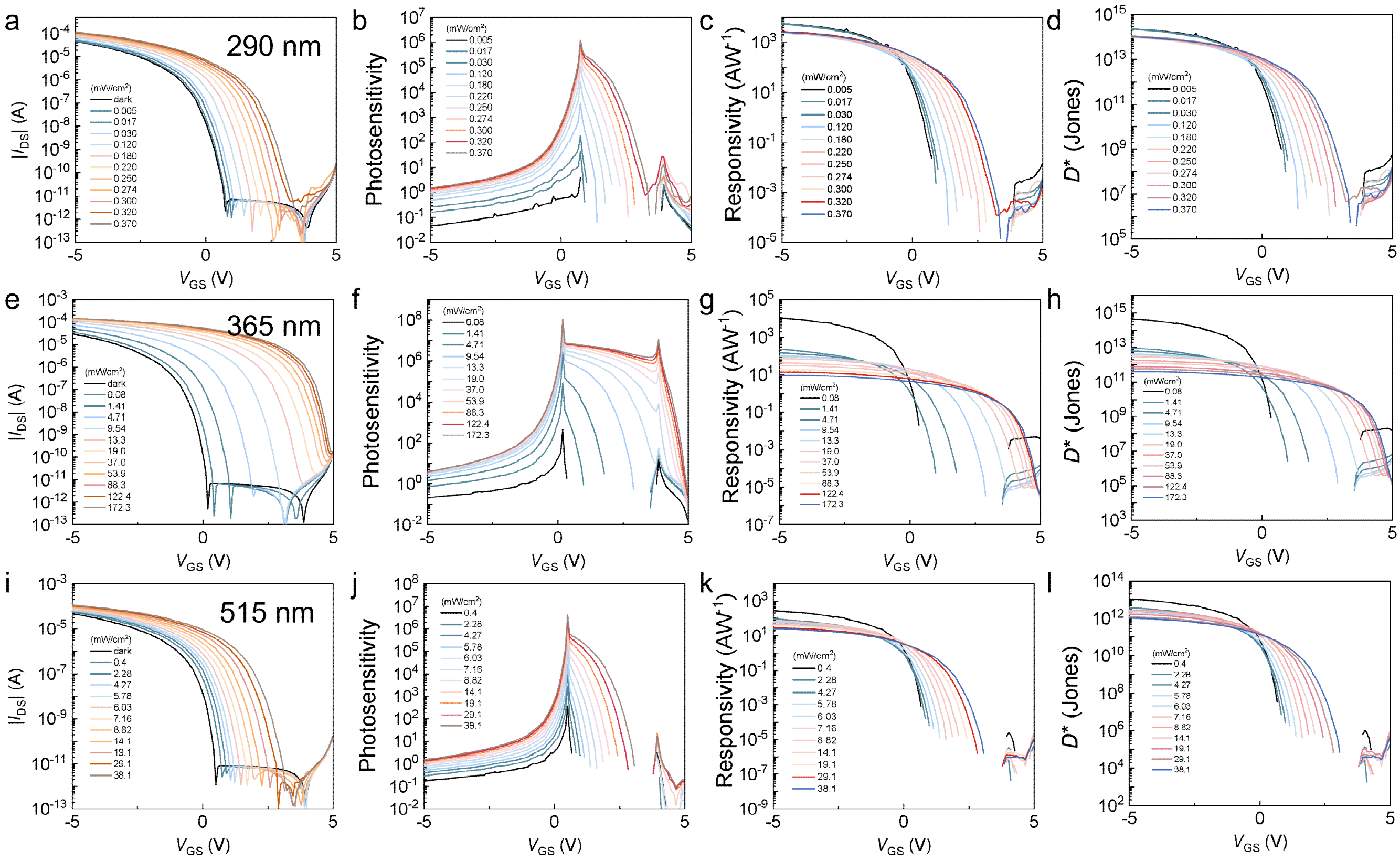

Supplementary Fig. 14 | Multi-wavelength photoresponse characteristics of the 30 nm Al_2O_3/180 nm PAA device. Photoresponse performance was systematically characterized under monochromatic illumination at 290 nm, 365 nm, and 515 nm. **a,e,i,** Transfer curves measured in the dark and under

illumination at 290 nm (**a**), 365 nm (**e**), and 515 nm (**i**). **b,f,j,** Corresponding *P* values extracted from the transfer curves. **c,g,k**, *R* values. **d,h,l,** Noise-corrected *D** values derived from the directly measured dark-state noise current (i_n= 2.05×10^{-13} A) The device exhibits clear photoresponse across all tested wavelengths.

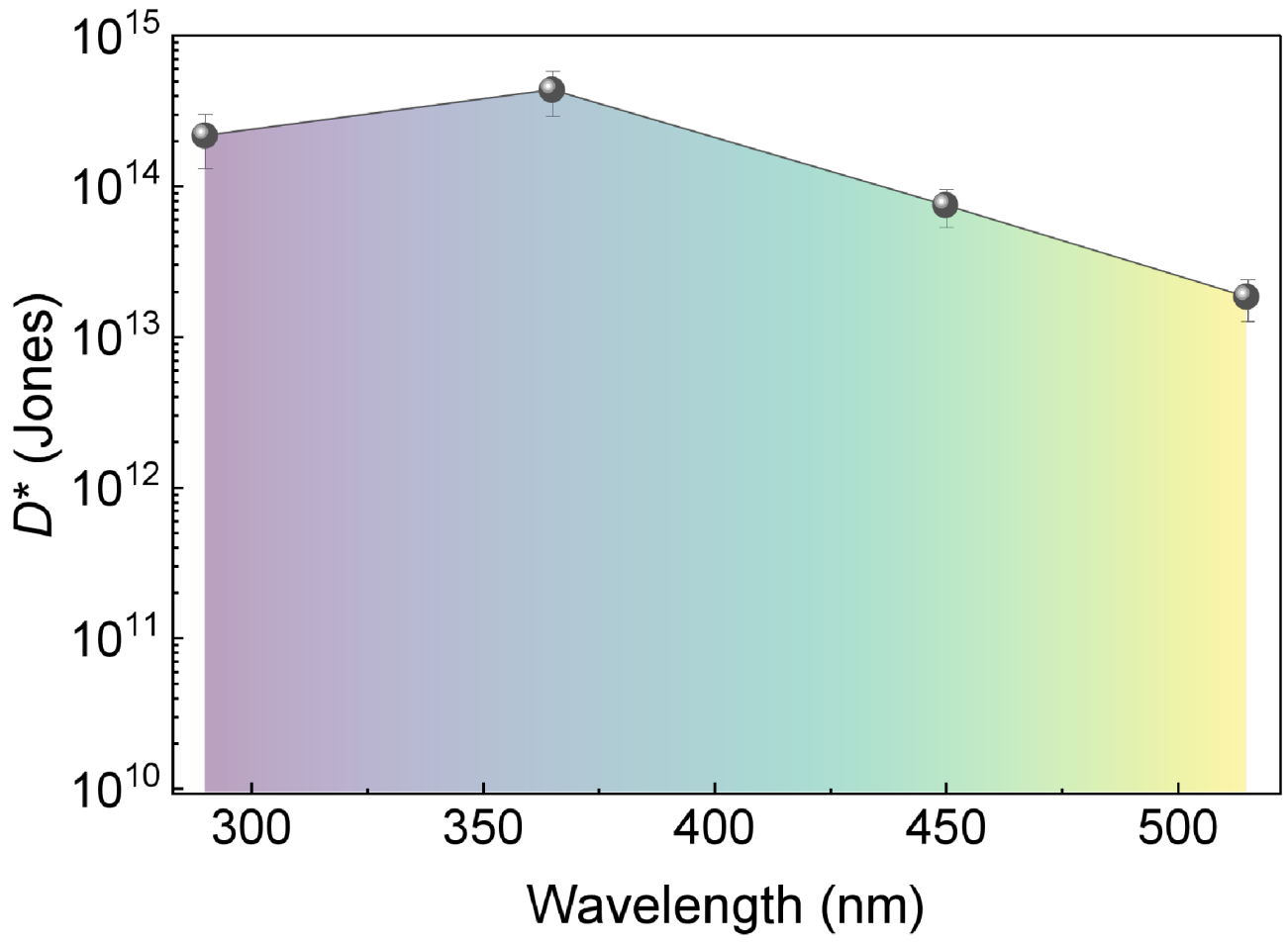

Supplementary Fig. 15 | Multi-device statistical summary of *D** at different wavelengths for the 30 nm Al_2O_3 / 180 nm PAA device.

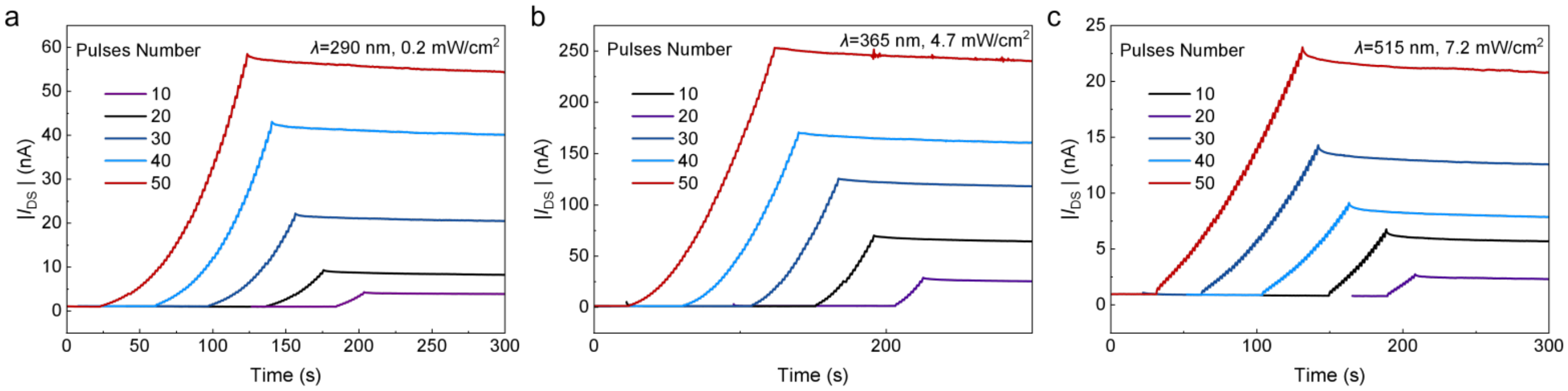

Supplementary Fig. 66 | Multi-pulse photoresponse behaviour of the 30 nm Al_2O_3/180 nm PAA device at different wavelengths. a–c, Excitatory postsynaptic current responses measured under consecutive light pulses (10, 20, 30, 40 and 50 pulses) at 290 nm (**a**), 365 nm (**b**) and 515 nm (**c**). At all tested wavelengths, the device exhibits reproducible pulse-number-dependent EPSC enhancement, mirroring the synaptic behaviour observed at 450 nm.

Page 9: The primary photoresponsive parameters of the device, derived from the photoresponse transfer curves, include a photosensitivity (*P*) value exceeding 10^7, a peak responsivity (*R*) value of 2.01×10^3 A W^{-1}, and a maximum specific detectivity (*D**) value approaching 10^{14} Jones, as illustrated in **Supplementary Fig. 12b-e**. This value is obtained from the directly measured noise current after noise correction (**Supplementary Fig. 13**). To further validate the multi-wavelength capability, extended the spectral response to 290 nm, 365 nm and 515 nm (**Supplementary Figs.**

14 and 15). The noise-corrected *D** values at these wavelengths reach up to 4.4×10^{14} Jones (365 nm), confirming that our device maintains robust photodetection across a multi-wavelength spectral range while preserving the outstanding performance at 450 nm...

Page 15: ... Beyond 450 nm, similar pulse-number-dependent EPSC enhancement was observed at 290 nm, 365 nm and 515 nm (**Supplementary Figs. 66**), confirming that the optical synaptic plasticity is a general feature across the tested spectral range...

Q4. Please consider including a table to benchmark the complete performance parameters of the reported heterogeneous dielectric layer and the resulting transistor against those in the literature to clearly demonstrate the advantages of this work.

A4. Thank you for your suggestion. To clearly benchmark the performance of our device against the state of the art, we have compiled a comprehensive comparison table summarizing the key parameters of recently reported organic and related neuromorphic transistors, including semiconductor, dielectric, operating voltage, mobility, retention time, and optical/electrical response speeds.

As summarized in Table R1 below, our device simultaneously achieves a high mobility of 22.65 $cm^2 V^{-1} s^{-1}$, an optical response time of 30 μs, an electrical pulse response down to 130 ns, a stable multilevel programming speed of 10 μs, and a retention time exceeding 10 years (extrapolated from >5 × 10^4 s measurements). This combination of fast sensing, fast programming, high mobility, and long retention is rarely achieved in a single organic device. Notably, while some reported devices show faster response in one specific metric (e.g., 3 ns optical response in Ref. 63 or 200 ns electrical response in Ref. 52), they suffer from significantly lower mobility (<0.1 $cm^2 V^{-1} s^{-1}$) or lack the integrated "sensing-memory-processing" functionality demonstrated in our work. Our Al_2O_3/PAA heterostructure design thus offers a unique balance of speed, mobility, and non-volatility, positioning it as a promising platform for flexible neuromorphic vision systems.

Supplementary Table 3: Performance comparison of representative non-volatile organic/2D optoelectronic memory devices.

Device Type	Semiconductor	Dielectric Layer	Operating Voltage	Mobility ($cm^2V^{-1}s^{-1}$)	Retention Time	Optical Response Speed	Electrical Response Speed	Ref.
FET	$ReSe_2$	hBN / SiO_2	0.5 V	—	>5,000 s (~3 months)	~1 ms	~1 ms	20
Fe-OL ET	C8-BTBT	P(VDF-TrFE) / PMMA	−40 V	8.1	—	—	0.5 s	28
OHTM	Pentacene / P13 / Pentacene	PVP / SiO_2	−30 V	0.23	>10^4 s (extrapolated to 10 yr)	—	1 s	29
FET	Pentacene	SiO_2+ PPyMA/TCNQ	−40 V	~0.01	>10^4 s	500 ms	1 s	30
FET	PDPP4T + p-NO_2-HABI	SiO_2	−80 V	0.4	>10^4 s	10 s	—	31
FET	$(POE)_2SnI_4$	SiO_2 + CL-PVP	−40 V	0.3	10^4 s	300 ms	5 s	32
OFET	HBP-H	SiO_2	−10 V	0.078	12 h	10 s	10 s	33
OFET	C8-BTBT + C8-PTCDI	FPS	−2 V	13.3	10^4 s (extrapolated to 30 yr)	300 ms	1 ms	53
OFET	BBTNDT	SiO_2 / Al_2O_3	−80 V	7.7	>2×10^4 s	10 s	10 s	54
OECT	PTBT-p	EMIMTFSI:PVDF-HFP	−0.8 V	—	>10^4 s	0.4 s	200 ns	55
OFET	Pentacene	SiO_2	−5 V	0.03	>10^5 s	1 s	5 s	56
OFET	Pentacene	PMMA / SiO_2	1.73 V	—	>10^4 s	1 s	1 s	57
OPT	BTBT	PMMA / SiO_2	−30 V	1.31	>10^4 s	1 s	10 µs	58
OFET	PTCDI-C13	pC1D1 / pV3D3	2.5 V	—	>10^5 s	—	100 µs	60
FET	IGZO	Ga_2O_3/Al_2O_3	−0.5 V	—	>72 h	50 ms	1 s	63
FET	IGZO	SiO_2	0.5 V	1.32	—	50 ms	—	64
OTFT	P3HT / DAE	SiO_2 / PMMA+PVP	−10 V	0.01	>4.3×10^7 s	3 ns	—	66
FET	WSe_2	Al_2O_3	0.05 V	—	>10^4 s	50 ms	—	67
Fe-FET	SnS_2	h-BN + CIPS	0.1 V	—	>10^4 s	0.5 s	0.1 s	68
OFET	Pentacene	SiO_2 + PAH	−40 V	0.013	10^4 s	—	10 µs	71
OFET	N2200 : TIPS-PEN	PS-brush	20 V	0.12	10^4 s (extrapolated to 10 yr)	—	1 µs	72
OFET	Pentacene	HfO_2 + DAE SAM	−0.1 V	0.7	>6×10^5 s	—	1 s	75
OTFT (This work)	C10-DNTT	Al_2O_3 / PAA	−5 V	22.65	>5×10^4 s (extrapolated to 10 yr)	30 µs	Response: 130 ns Storage: 10 µs	This work

Page 14: ... Compared to previously reported organic devices, our device shows a significant advantage in operating speed (Fig. 4f [28, 33, 53, 55, 60, 70, 71, 75]), with a distinction between the fastest electrical response (130 ns) and stable multilevel programming (10 µs). A comprehensive performance comparison is provided in Supplementary Table 3...

Q5. The use of machine learning models often faces the challenges of parameter overfitting, making them difficult to apply in new data sets or new human subjects. Could the authors provide

relevant validation and demonstration for the recognition of handwritten digits/gestures/faces from unseen human subjects?

A5. Thank you for your question. We agree that overfitting and subject-dependent bias are important issues in machine-learning-based recognition tasks. In our study, the training and testing datasets were strictly separated. For the handwritten digit, gesture, and face recognition tasks, the testing data were not used during either model training or parameter optimization. In particular, the testing sets were constructed from samples/subjects that were excluded from the corresponding training sets. Therefore, the reported recognition accuracies were obtained using unseen testing data/subjects, rather than by re-evaluating the model on the training data.

In response to this comment, we have revised the manuscript and Supporting Information explicitly describe the data partitioning strategy for each recognition task and to clarify that no testing samples were involved in the training process. These results support the generalization capability of the model under unseen-data/subject conditions. The related content has been added and marked in red on page 17 in the manuscript and **Supporting Note S1**.

Page 17: …For all recognition tasks, the training and testing datasets were strictly separated. The test samples/subjects for handwritten digit, gesture and face recognition were excluded from the training process and were not used for parameter optimization.

Q6. Besides the demonstration using a Zynq SoC, is it possible to provide relevant demonstrations on in-sensor computing?

A6. Thank you for this constructive suggestion. In response, we have further clarified the relevance of our optically modulated synaptic-device demonstration to in-sensor computing. In our device, optical stimuli can directly induce long-term potentiation, while electrical stimuli can induce long-term depression, enabling bidirectional conductance-state modulation in the same sensing/synaptic device. In this sense, the incident optical signal is not only sensed but also locally converted into a nonvolatile synaptic conductance state, providing a device-level basis for sensor-memory-computing integration.

Based on the experimentally measured optically induced LTP and electrically induced LTD characteristics, we further evaluated the potential of the device for visual recognition tasks, including MNIST handwritten digit, hand gesture, and Yale face recognition. An MLP network was

simulated using experimentally calibrated synaptic-device parameters, with strictly separated training and testing datasets. The simulated system achieved recognition accuracies of 94.78%, 99.83%, and 92.56% for the flat device, respectively, and retained robust performance of 93.88%, 96.67%, and 91.96% under mechanical bending with a radius of 3 mm.

We note that these recognition results were obtained from NeuroSim-based simulations parameterized by experimentally measured/fitted device characteristics, rather than from direct inference in a physical in-sensor array. Therefore, in the revised manuscript, we describe these results as an experimentally calibrated system-level evaluation toward flexible in-sensor neuromorphic computing, rather than as a complete hardware-level in-sensor computing demonstration. The corresponding revisions have been added and marked in red on page 17 of the manuscript.

Page 17: …Critically, the device conductance (G_{DS} = EPSC / V_{DS}) exhibited symmetrical and reversible LTP and LTD, which is essential for artificial synaptic weight updating.

Page 17:…Since optical stimuli can directly induce nonvolatile potentiation of the device conductance, the sensing input can be locally converted into a synaptic memory state, providing a device-level basis for sensor-memory-computing integration and future in-sensor neuromorphic computing. To further evaluate this potential at the system level, a multilayer perceptron (MLP) network was simulated using experimentally calibrated synaptic-device parameters.

Q7. The energy efficiency claim probably does not include the peripheral circuits (which often consume the most power). If so, the implication in the practical applications should be discussed.

A7. Thank you for this important comment. Peripheral circuits, such as drivers, readout/sensing circuits, ADC/DAC blocks, and control logic, can contribute substantially to the total power consumption in practical hardware implementations and therefore are essential for a rigorous system-level energy-efficiency evaluation. In the present work, the reported energy-efficiency metric mainly reflects the operation of the core synaptic computing module/accelerator and does not explicitly include the power consumption of all peripheral circuits. We acknowledge that, without these overheads, the reported TOPS/W value should not be interpreted as a complete system-level metric for practical deployment. In particular, the inclusion of peripheral circuitry would reduce the overall energy efficiency in a realized hardware system. At the current stage, we are not able to

accurately quantify the peripheral-circuit overhead, since it strongly depends on the specific circuit implementation, interface design, and system integration strategy. Following the reviewer's suggestion, we have revised the manuscript to explicitly clarify this limitation and to moderate the practical implication of the energy-efficiency claim. The revised text now emphasizes that the reported results mainly demonstrate the intrinsic energy-efficiency potential of the core sensing–memory–processing architecture, while a comprehensive system-level evaluation including peripheral overhead remains an important topic for future work.

In response, we have revised the manuscript to clarify that the reported energy-efficiency metric mainly reflects the core computing module and does not explicitly include peripheral-circuit overhead. The corresponding revisions have been added and marked in red on page 20 of the manuscript.

Page 19: …This design offers high intrinsic energy-efficiency potential within an ultra-low power envelope, offering a scalable solution for resource-constrained embedded and edge computing applications. It should be noted that the energy-efficiency analysis in this work mainly concerns the core computing module and does not explicitly include the overhead of peripheral circuits; therefore, the practical system-level efficiency may be lower and remains to be evaluated in future hardware implementations.

Page 20: …thereby demonstrating the feasibility of mapping algorithmic workloads onto synaptic hardware.

Page 20: …confirming that our synaptic architecture can provide reliable and accurate performance for complex visual detection tasks.

Q8. It is less convincing to use linear extrapolation for projecting a retention time exceeding ten years.

A8. Thank you for your question. In our original manuscript, we measured the retention characteristics for approximately 30,000 seconds and then performed a linear extrapolation to project a retention time exceeding 10 years. To further strengthen the credibility of this projection, we have now extended the retention measurement to 50,000 seconds. As shown in **Fig. 3b**, the device maintains an on/off current ratio > 10^4 over the entire 50,000 seconds measurement period, and the linear extrapolation from this extended dataset still projects a retention time exceeding 10

years with a comfortably maintained on/off ratio. The consistency between the projections from the 30,000 s and 50,000 s datasets confirms the robustness of our extrapolation.

We would also like to emphasize that linear extrapolation of retention time based on measured short-term data is a standard and well-accepted practice in the field of organic transistor memory. Numerous high-impact publications have employed this method to project long-term retention performance. For example: Lee et al.[1] reported organic flash memory with retention over 10 years using extrapolation from measured data (Nature Communications, 2017); Li et al.[2] demonstrated low-voltage ultrafast nonvolatile memory with a projected retention of 10 years via extrapolation (Nature Communications, 2022); and Wu et al.[3] achieved long-retention ferroelectric memory with retention exceeding 10 years based on extrapolated retention characteristics (Nature Communications, 2021). These examples demonstrate that linear extrapolation from experimentally measured retention data is a routine and scientifically accepted approach for estimating long-term retention in nonvolatile memory devices, particularly when direct measurement over the full 10-year timeframe is experimentally impractical. We have also added relevant statements on page 10 of the revised manuscript.

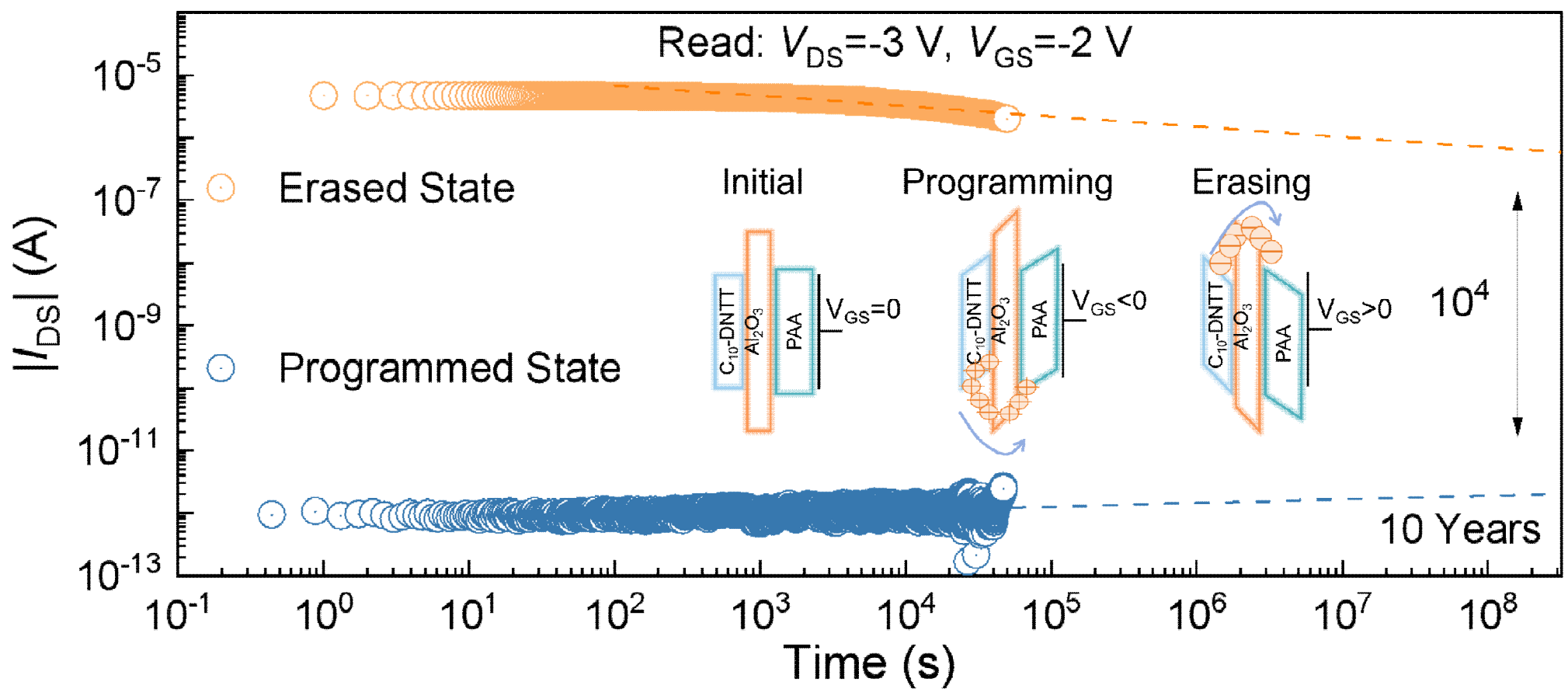

Fig. 3b. Device programming state and erase state currents for linear extrapolation. (Currents are read at V_{DS} = −3 V and V_{GS} = −2 V) and operating schematic of Al_2O_3/PAA based memory devices.

Page 10: ... Retention was evaluated by monitoring the read current after programming/erasing (Fig. 3b). The device sustains an on/off current ratio $>10^6$ for over 50,000 s. Linear extrapolation projects a retention time exceeding ten years with a ratio $>10^4$ meeting commercial standards...

Q9. The polymer polarity results are a bit under-leveraged in the demonstrations/applications.

A9. Thank you for your question. We agree that the polymer polarity results could be more explicitly connected to the subsequent device applications, and we appreciate the opportunity to clarify our rationale and strengthen the narrative.

In our original manuscript, the systematic comparison of dielectrics with varying polymer polarities was designed with a specific purpose: to elucidate the bottom-up modulation effect of the polar polymer layer on charge trapping efficiency and carrier transport at the dielectric/semiconductor interface. We compared five polymers—PAA, PAN, PVP, PS, and PPO—with decreasing polarity, and systematically characterized the resulting transistor performance, including carrier mobility (extracted from transfer curves) and memory window. The clear correlation between polymer polarity and device performance (higher polarity → larger memory window and higher mobility) confirmed that the interfacial electron-withdrawing strength plays a critical role in synergistically enhancing charge trapping while maintaining efficient transport. To further validate the generality of this conclusion, we replaced the semiconductor C10-DNTT with DNTT and observed the same polarity-dependent trends, confirming that this bottom-up modulation mechanism is not limited to a specific semiconductor material.

It is important to emphasize that this polarity screening was not an end in itself, but rather a systematic approach to identifying the optimal dielectric interface—specifically, the 30 nm Al_2O_3 / 180 nm PAA heterostructure, which exhibited the best balance of mobility and memory performance among all tested configurations. All subsequent demonstrations of integrated "sensing-memory-processing" functionality, including optical sensing, nonvolatile memory, synaptic plasticity, pattern recognition, and real-time face detection, were carried out exclusively on devices fabricated with this optimized interface. Similarly, our earlier thickness optimization (varying Al_2O_3 and PAA thicknesses) was also part of this screening process, aimed at identifying the most favorable dielectric architecture before proceeding to application-oriented experiments.

In other words, the polarity comparison served as a foundational screening tool that guided us toward the optimal interface. Without this systematic investigation, the high-performance neuromorphic applications we subsequently demonstrated would not have been possible. We have now revised the manuscript to more clearly articulate this logical flow. We have also added relevant statements on page 13 of the revised manuscript.

Page 13: ...Replacing C10-DNTT with DNTT preserved the same polarity-dependent trends, confirming the generality of this bottom-up modulation mechanism (**Supplementary Figs. 50-59**). This polarity screening guided us to identify the 30 nm Al_2O_3/PAA heterostructure as the optimal interface, which synergistically enhances charge trapping efficiency through PAA's strongly polar interface while maintaining high carrier mobility—providing an ideal platform for high-performance "sensing–memory–processing" integrated devices.

Reviewer #2 (Remarks to the Author):

This manuscript reports flexible organic thin-film transistors (OTFTs) employing an Al_2O_3/PAA heterogeneous dielectric to achieve integrated optical sensing, nonvolatile memory, and synaptic functions. The device-level performance, particularly the high carrier mobility, robust bending stability, and the implementation of LTP/LTD through synergistic photoelectric modulation—is noteworthy and potentially impactful for the flexible electronics community.

However, the central claims of the manuscript significantly exceed the provided experimental evidence. Several headline performance metrics appear to conflate qualitative observations or calibrated simulations with rigorous, direct device validation. In its current form, the manuscript lacks the technical depth and empirical certainty required for Nature Communications.

Response. We sincerely thank you for the recognition of our work and for the critical and thorough evaluation of our claims. In response, we have carefully addressed each concern through additional experiments, revised data analysis, and extensive rewrites to ensure that all claims are rigorously supported by direct experimental evidence. Specifically, we have: (1) clarified the respective roles of the OTFT synaptic devices and the FPGA in the inference pipeline, revising the relevant text and figures to avoid any ambiguity regarding hardware implementation; (2) explicitly stated that the MNIST, gesture, and Yale face recognition results were obtained from NeuroSim-based simulations parameterized by experimental data, rather than from physical array inference; (3) rigorously distinguished the fastest electrical pulse response (130 ns) from stable non-volatile programming (10 μs), providing post-pulse transfer curves, retention data, and transient current measurements to support this distinction; (4) corrected the optical response time from “< 10 μs” to the directly measured 30 μs (10%–90% rise time) and revised the Abstract and main text accordingly; (5) clarified the capacitance frequency used for each mobility extraction,

performed TLM measurements to rule out contact-resistance artefacts, and provided directly measured noise current for specific detectivity calculations; and (6) provided comprehensive EPR, XPS, and KPFM evidence to corroborate the oxygen-vacancy trapping mechanism in the Al_2O_3/PAA heterostructure. We believe that these revisions have fundamentally improved the technical depth and empirical certainty of our work.

Q1. The manuscript states that the synaptic devices “physically execute the computation-intensive convolutional and fully connected layers.” However, Fig. 6b indicates that convolution, activation and pooling are performed by the Programmable Logic of the Zynq SoC, while the OTFT devices are not clearly shown to be directly interfaced with the FPGA data path. The role of the devices in the inference pipeline is therefore unclear. The authors should provide the physical array size, weight-to-conductance mapping, device-in-the-loop MAC/VMM results, ADC/DAC specifications, latency, energy per inference, and comparison with a pure FPGA/software baseline.

A1: Thank you for your comment. We agree that the original wording may have caused ambiguity regarding the respective roles of the OTFT devices and the FPGA in the inference workflow.

In this work, the FPGA is not used to demonstrate a physical analog compute-in-memory (CIM) array. Instead, it serves as a reconfigurable hardware-level emulation and evaluation platform based on experimentally measured OTFT synaptic characteristics. Specifically, the OTFT devices provide the measured conductance modulation behavior, including programmable conductance states and device non-idealities associated with synaptic plasticity. Based on these experimental characteristics, we constructed a device-aware synaptic model and implemented it on FPGA to perform neural network inference under hardware-relevant constraints.

We used FPGA for three main reasons. First, FPGA allows the experimentally measured OTFT conductance plasticity to be embedded into a digital hardware inference flow in a controllable and reproducible manner. Compared with pure software simulation, FPGA implementation more faithfully reflects practical hardware constraints such as fixed-point quantization, parallel data paths, memory access behavior, and logic resource utilization. Second, direct integration of large-scale physical OTFT arrays with peripheral circuits such as DACs, ADCs, and control logic is beyond the scope of the present work. The objective here is to verify whether experimentally measured OTFT conductance plasticity can support neural network inference when mapped into a hardware-oriented computational framework. Third, FPGA offers high reconfigurability for

implementing convolution, activation, pooling, and fully connected operations while embedding the weight/conductance model derived from OTFT measurements into the inference process.

Therefore, the original statement that "synaptic devices physically execute the computation-intensive convolutional and fully connected layers" was not sufficiently accurate and may lead to misunderstanding. In response, we have revised the manuscript to clarify that the convolutional and fully connected layers are executed in the programmable logic of the Zynq FPGA, whereas the weight model is derived from experimentally measured OTFT conductance plasticity. In other words, the OTFT devices provide the physical basis for synaptic modeling, and the FPGA provides the hardware emulation platform for system-level inference evaluation.

Accordingly, we have revised the relevant text and figure description (including **Fig. 6b**) to clearly distinguish the roles of OTFT device data and FPGA-based hardware emulation in this work. The corresponding revisions have been added and marked in red on page 19 and page 20 of the manuscript.

Page 19: …we address by incorporating a device-aware synaptic weight model derived from experimentally measured OTFT conductance plasticity into an FPGA-based inference framework. By minimizing control logic and retaining only essential arithmetic and storage units, our dataflow-driven architecture establishes a hardware-oriented inference and evaluation pipeline that substantially reduces off-chip memory access overhead.

Page 20: …The accelerator leverages the intrinsic LTP/LTD characteristics of experimentally measured OTFT synaptic characteristics are used to construct a device-aware weight model, while the actual convolutional and fully connected operations are executed on FPGA, thereby demonstrating the feasibility of mapping algorithmic workloads onto synaptic hardware.

Q2. Supporting Note S1 states that the MLP network was designed using NeuroSim for simulation, with experimentally fitted conductance parameters. Thus, the MNIST, gesture and Yale face results appear to be device-parameter-calibrated simulations, not inference performed on a physical synaptic array. Describing these results as "implemented with synaptic devices" is misleading unless supported by device-in-the-loop measurements.

A2: We thank the reviewer for pointing out this important issue. We agree that the previous wording may have over-implied hardware implementation and could therefore be misleading. The MNIST,

gesture, and Yale face recognition results were not obtained by directly performing inference on a physical synaptic array, nor through device-in-the-loop measurements. Instead, these results were obtained from NeuroSim-based simulations parameterized by experimentally measured/fitted conductance characteristics of our synaptic devices. These simulations were intended to assess the potential system-level inference performance under experimentally calibrated device constraints, rather than to demonstrate a complete hardware-implemented synaptic-array system.

In response to this comment, we have revised the relevant descriptions throughout the Supporting Information to avoid ambiguity. The corresponding revisions have been added and marked in the **Supporting Note S1**.

Page S76: ...In this paper, an image-aware computational recognition system was evaluated through NeuroSim-based simulations using experimentally measured/fitted conductance parameters of the synaptic devices. The simulations were intended to assess the potential system-level inference performance under device-realistic conductance-update constraints. The MNIST, gesture, and Yale face recognition results were obtained from simulations rather than from inference directly performed on a physical synaptic array or device-in-the-loop measurements.

Page S77: ...During backpropagation, the error was propagated backward, and the network weights were updated using stochastic gradient descent. In the NeuroSim simulation, the calculated weight updates were mapped to equivalent LTP or LTD conductance-update steps according to the experimentally fitted device characteristics. These conductance updates were numerically simulated in NeuroSim, and no physical write pulses were applied to a real synaptic array during the recognition simulations.

Page S78: ...For all recognition tasks, the training and testing datasets were strictly separated. The testing samples/subjects were not used during training or parameter optimization. For the Yale face recognition task, data augmentation was applied only to the training set because of the limited number of available samples. The augmentation operations included image rotation, center cropping, and color-space enhancement. The testing set remained independent and was used only for final performance evaluation. Representative augmented images are shown in Supplementary Fig. 55.

Q3. Figs. 4b, c show memory-window formation under 10 μs gate pulses, whereas Fig. 4d shows “tunable suppression behavior” under 5 μs, 1 μs and 130 ns pulses. This appears to reflect

short-pulse synaptic inhibition rather than stable nonvolatile programming. No post-pulse transfer-curve shift, retention trace, read-current state or device statistics after a 130 ns pulse are provided. To claim 130 ns programming, the authors must show post-pulse memory states, retention data, terminal-level pulse waveforms and RC/parasitic analysis.

A3: We sincerely thank you for this comment. We agree that claiming "130 ns programming" requires robust evidence of post-pulse memory states and retention behaviour, and we appreciate the opportunity to clarify our experimental capabilities and to provide additional data to strengthen our claims. In our original manuscript, we aimed to explore the fastest possible electrical response of our devices.

For gate pulses longer than 10 μs, we used a semiconductor parameter analyser (FS-Pro) alone, which can deliver arbitrary gate voltages up to 60 V with a minimum pulse width of 10 μs. As shown in **Figs. 4b–c** of the original manuscript, the device exhibits clear memory-window formation under 10 μs programming pulses at voltages ranging from –10 V to –30 V. In the revised manuscript, we have included detailed retention traces and read-current states for six programming voltages under 10 μs pulses (**Fig. 4a**), directly demonstrating stable non-volatile storage at this timescale. These data confirm that our device can achieve robust multilevel storage at 10 μs speed. Our measurement setup requires the use of a fast pulse module (PRIMARIUS FS430-PAMUHP-FWGMK) in combination with a signal amplifier when dealing with pulses shorter than 10 μs. We must candidly acknowledge a technical limitation of this module: **it can only supply gate voltage pulses up to –10 V at the sub-microsecond pulse width.** Under this constraint, we are unable to apply higher-amplitude pulses within this time domain to drive the device into a stable non-volatile charge-trapping state. To verify the device's fast electrical response capability, we measured the post-pulse transfer-curve shift under –10 V, 1 μs pulse conditions (**Supplementary Fig. 60**). Although a certain memory window can be observed, its retention is limited, indicating that this behaviour corresponds to short-pulse synaptic inhibition rather than stable non-volatile programming. At the faster 130 ns pulse width (**Fig. 4d** of the original manuscript), the device only exhibits a clear instantaneous current suppression effect, confirming that it possesses a sub-microsecond electrical response speed, but without forming a persistent storage window.

To further corroborate the device's fast response with independent methods, we performed two additional experiments. First, we applied gate pulses of –3 V, –5 V and –10 V with a 1 μs width from a signal generator, while reading the drain current with a sourcemeter. The I–T characteristics

(**Supplementary Fig. 61**) clearly show that the channel current is modulated by the electrical pulses; notably, the device exhibits a retention ability on the second scale at a 1 μs electrical pulse speed. Second, we employed a current pre-amplifier to convert the drain current into a voltage signal, which was read out by an oscilloscope with a 10 ns sampling interval (**Supplementary Fig. 62**). This confirms that our devices do indeed exhibit an electrical response on the order of 1 μs, but that this response is insufficient to ensure long-term stable storage. **It should be noted that, in an effort to overcome the instrumental limitation of only being able to supply –10 V at sub-microsecond pulse widths, we have made collaborative attempts with multiple institutions (including the School of Materials Science and Engineering at Nankai University and the College of Materials Science and Engineering at Beijing University of Chemical Technology), but none have succeeded in achieving higher operating voltages in this time domain.** Should a compatible pulse generation system become available in the future, we believe that the stable non-volatile storage speed of our device could be advanced to the sub-microsecond level.

Based on the above results, we have revised our manuscript to draw a clearer and more rigorous distinction between the electrical response speed and the stable non-volatile programming speed of our device. Specifically, we now explicitly state that the fastest **electrical pulse response reaches 130 ns**, as evidenced by the distinct modulation observed in **Fig. 4d**, whereas the reliable multilevel non-volatile storage is achieved at a **programming speed of 10 μs**, supported by post-pulse transfer curves, retention characteristics, and read-current states measured under programming voltages from –10 V to –30 V. Corresponding modifications have been carefully made throughout the Abstract, main text, and figure captions to ensure this distinction is consistently reflected and to avoid any overstatement. We have also added relevant statements on pages 2, 4, 13, 14 and 20 of the revised manuscript.

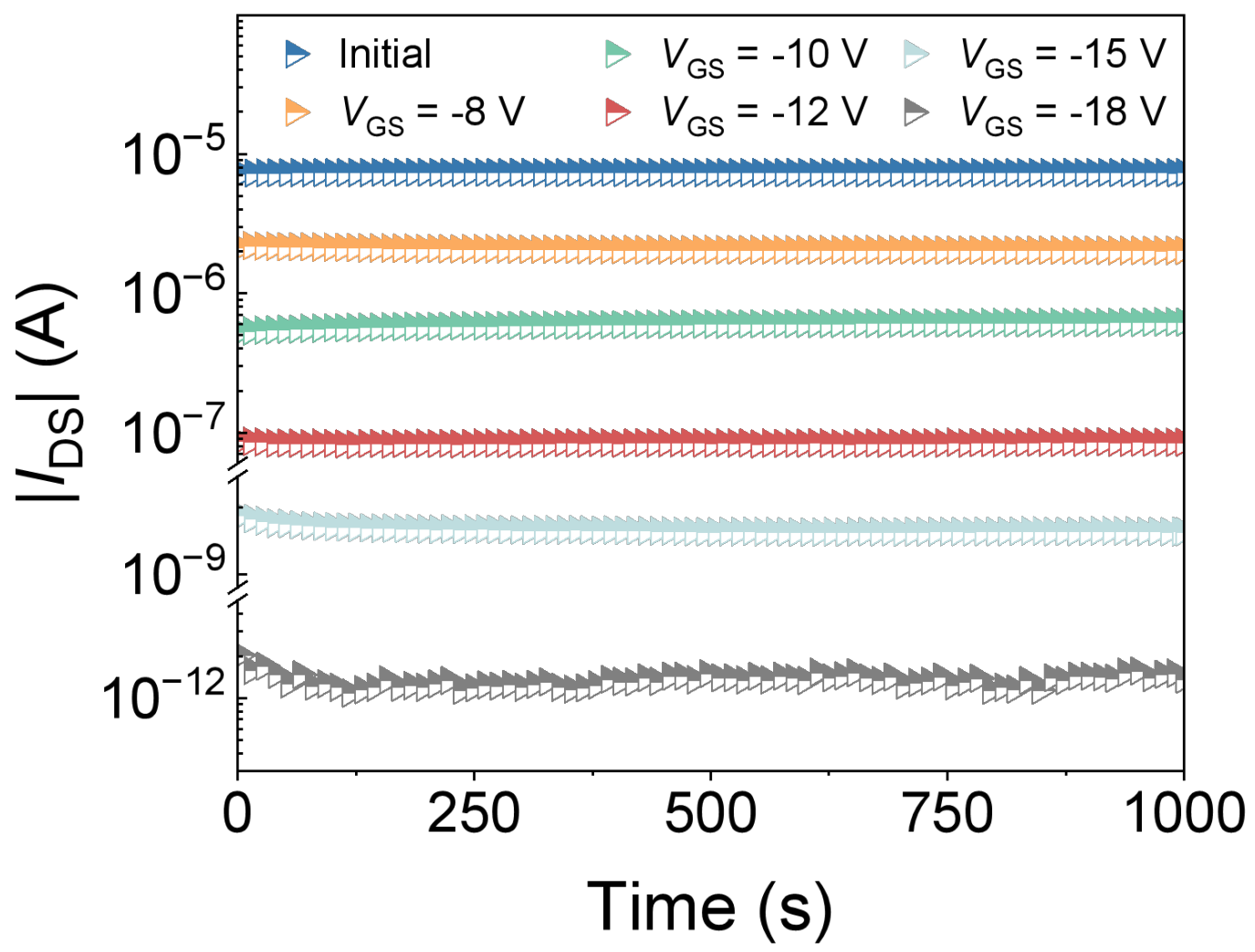

Fig. 4a, Retention characteristics of the device under different programming voltages. The read current is monitored as a function of time after applying 10 μs programming pulses at V_{GS}= −8 V, −10 V, −12 V, −15 V and −18 V (read at V_{DS}=-3V, V_{GS}=-2V).

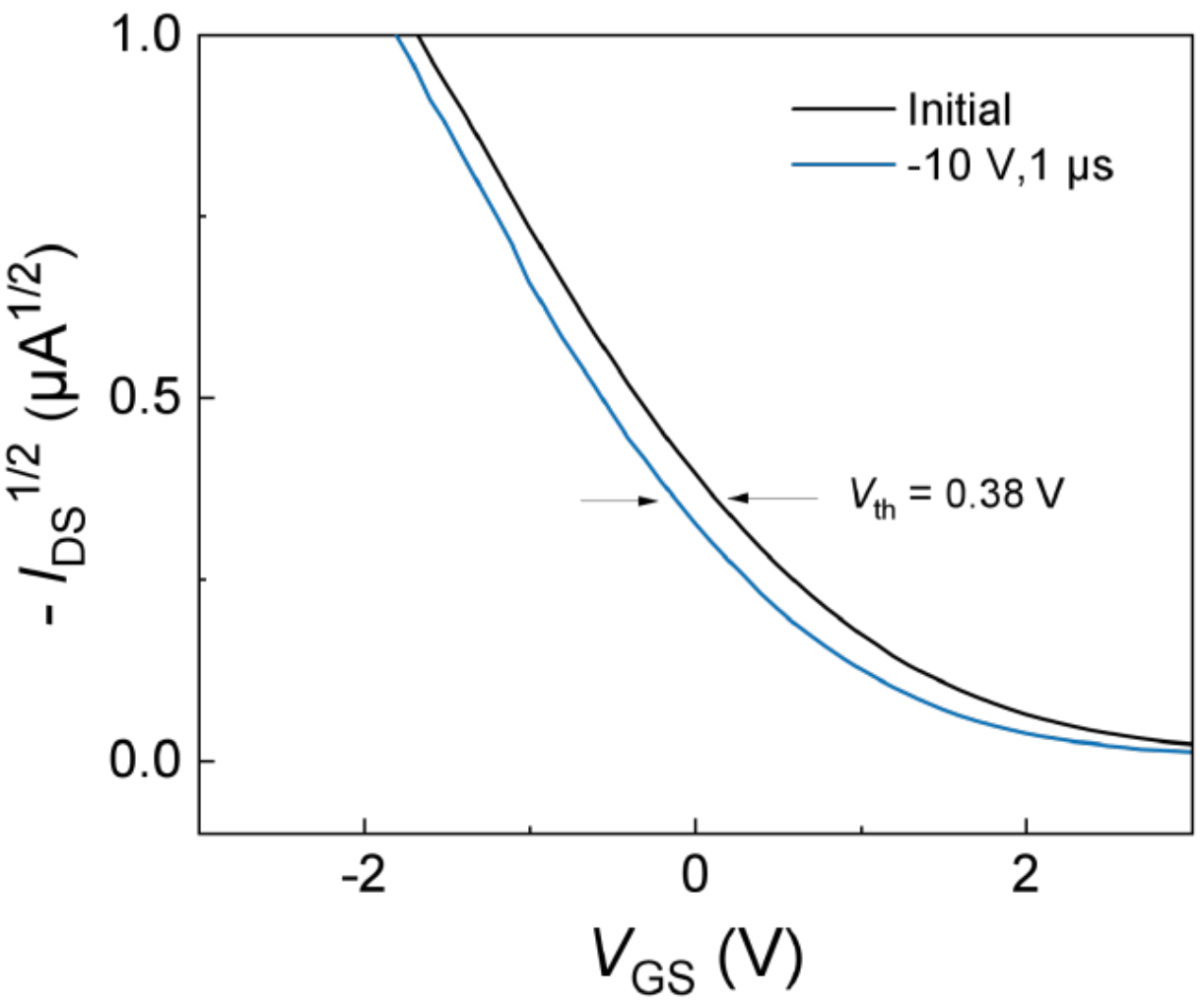

Supplementary Figure 60 | The storage window of the device following the application of a –10 V, 1 μs programming pulse. A slight negative shift in the transfer curve was observed, indicating limited charge retention capability over this short time scale.

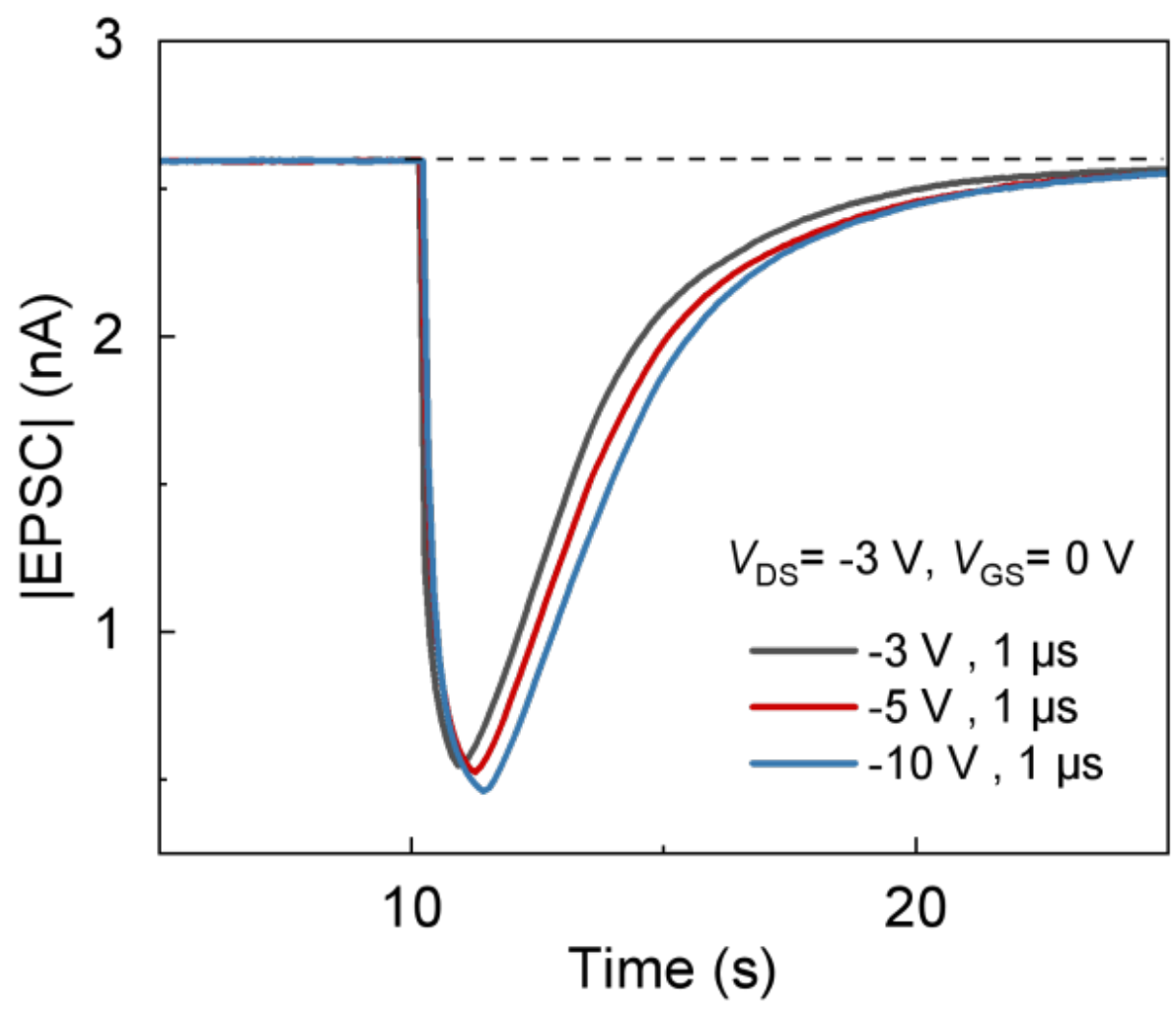

Supplementary Fig. 61 | Real-time drain current modulation under 1 μs gate pulses. The device exhibits clear current responses to –3 V, –5 V and –10 V pulses (1 μs width), confirming electrical modulation at sub-microsecond timescales, though the changes are transient rather than non-volatile.

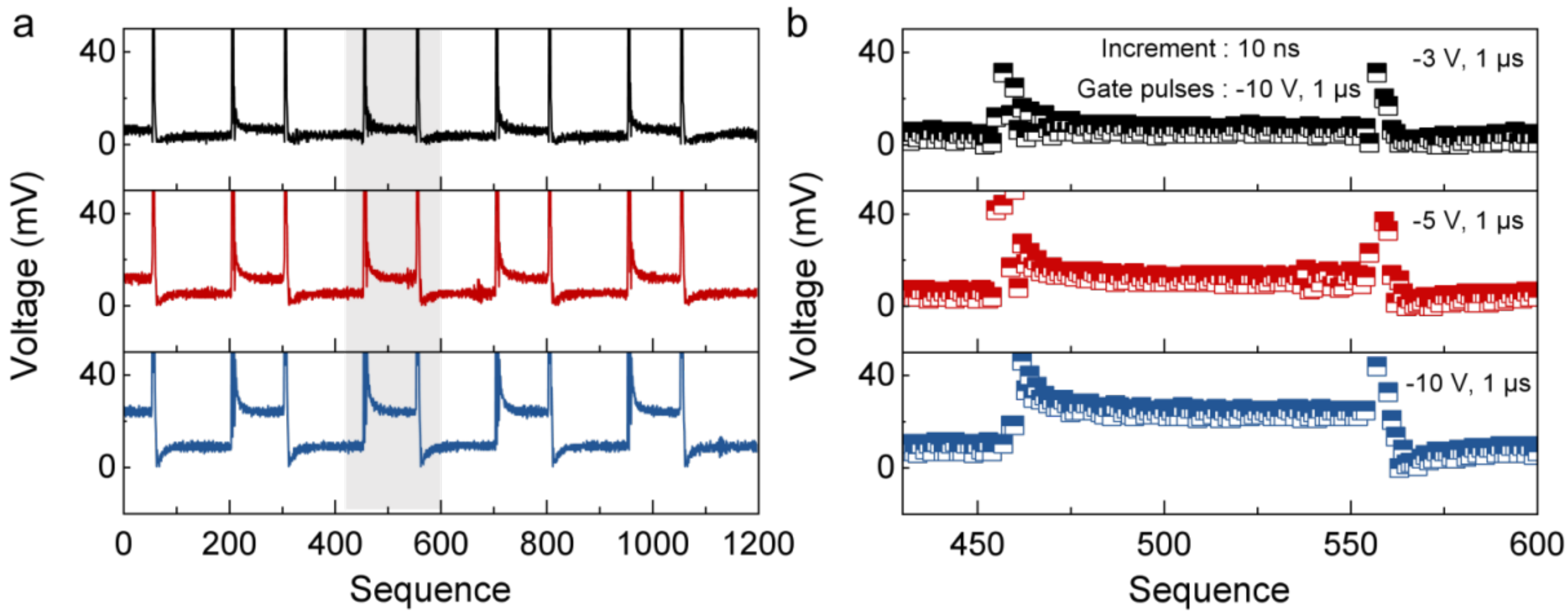

Supplementary Fig. 62 | Real-time electrical response to 1 μs gate pulses measured with a current pre-amplifier and oscilloscope (10 ns sampling interval). a, Transient drain current response to multiple 1 μs pulses at –3 V, –5 V and –10 V. **b**, Enlarged single-pulse view, resolving the current dynamics on the sub-microsecond scale. The clear modulation confirms the device's fast electrical responsiveness, though the response is transient rather than non-volatile.

Page 2: ...Concurrently, the device demonstrates outstanding stability, featuring a stable charge storage time of over ten years, a write/erase endurance exceeding 10,000 cycles, an electrical pulse response speed down to 130 ns, and a stable multilevel programming speed of 10 μs.

Page 4: For sensing performance, the device exhibits outstanding detection sensitivity for

monochromatic illumination at 450 nm and an optical response time (10%–90% rise time) of 30 μs. In memory applications, it demonstrates an electrical pulse response speed down to 130 ns, a stable multilevel programming speed of 10 μs, a memory window of 22.2 V, long-term retention stability over 10 years, and reliable cycling endurance exceeding 10^4 cycles.

Page 13: ...Applying distinct programming voltages (from −8 V to −18 V) with 10 μs pulses enables six well-separated storage states, which persist for over 10^3 s, demonstrating potential for multilevel memory applications (Fig. 4a)...

Page 13: ... However, due to the technical limitation of our high-speed pulse module, which cannot provide gate voltages exceeding –10 V at sub-microsecond pulse widths, the –10 V, 1 μs condition yields only a small memory window of 0.38 V (Supplementary Fig. 60). From another perspective, we characterised the rea-time I–T changes before and after applying 1 μs electrical pulses (Supplementary Fig. 61), which reveal a retention ability on the second scale, together with oscilloscope readout at 10 ns resolution to capture the instantaneous conductance change (Supplementary Fig. 62). Both measurements confirm the device's responsiveness to 1 μs electrical signals. Nevertheless, this response is more indicative of transient current suppression, because at such short pulse widths and with the limited voltage amplitude available, stable charge trapping cannot be established. Therefore, it is essential to distinguish the fastest electrical pulse response from stable non-volatile programming. Based on our results, we define 130 ns as the fastest electrical response speed and 10 μs as the programming speed for stable multilevel storage...

Page 20: ...The resulting flexible device integrates superior "sensing-memory-processing" capabilities: a fast optical response (30 μs), electrical response with a fast switching speed (130 ns) and a stable programming speed (10 μs)...

Q4. Fig. 2g shows current variation at 10 μs readout intervals during a 50 μs optical pulse, which does not by itself establish a sub-10 μs response time. Supplementary Fig. 11 only shows optical stimulation down to 100 μs. The manuscript does not provide 10–90% rise/fall times, optical pulse edge verification, amplifier bandwidth or RC-time analysis. The authors should provide bandwidth-limited transient photocurrent measurements and multi-device statistics. Without these data, the "<10 μs response" claim should be removed from the Abstract or replaced with the directly

measured timescale.

A4: We sincerely thank you for this rigorous correction. In our original experiments, we used a signal generator combined with a semiconductor laser to generate optical pulses. The shortest pulse width we could achieve with our current setup was 50 μs, with a readout interval of 10 μs. In our initial analysis, we mistakenly interpreted the current change observed within the first 10 μs as the response time. We now recognize that this interpretation was incorrect, and we sincerely apologies for this oversight.

To properly determine the optical response speed, we have now re-analyzed our transient photocurrent data using the standard 10%–90% rise-time definition. As shown in **Figure 2g**, the rise time (from 10% to 90% of the peak photocurrent) is approximately 30 μs. We have accordingly revised the Abstract, main text, and Figure captions to state that our device achieves an optical response time of 30 μs rather than the previously claimed "<10 μs".

We would also like to clarify a conceptual point regarding fall time. Our device is designed as a synaptic phototransistor for neuromorphic vision applications, not as a conventional optical switch. In synaptic devices, the sustained photocurrent after the optical pulse (i.e., the retention of the excited state) is not a drawback but rather a desirable feature, as it reflects the non-volatile memory characteristics essential for "sensing-memory-processing" integration. Therefore, we do not target a fast fall time; instead, the long retention of the photoresponse after optical stimulation is a key advantage for memory and neuromorphic computing functions. As demonstrated in our previous responses (and in the revised manuscript), the device can be effectively erased by applying an electrical gate pulse, returning the current to the baseline state when needed. This electrical erasure capability provides the necessary reset functionality without requiring a fast optical decay. All corresponding revisions have been made in the Abstract, main text, and Supplementary Information. We are deeply grateful to the reviewer for pointing out this error, which has allowed us to significantly improve the accuracy and credibility of our reporting. We have also added relevant statements on page 2, page 4, page 9, and page 20 of the revised manuscript.

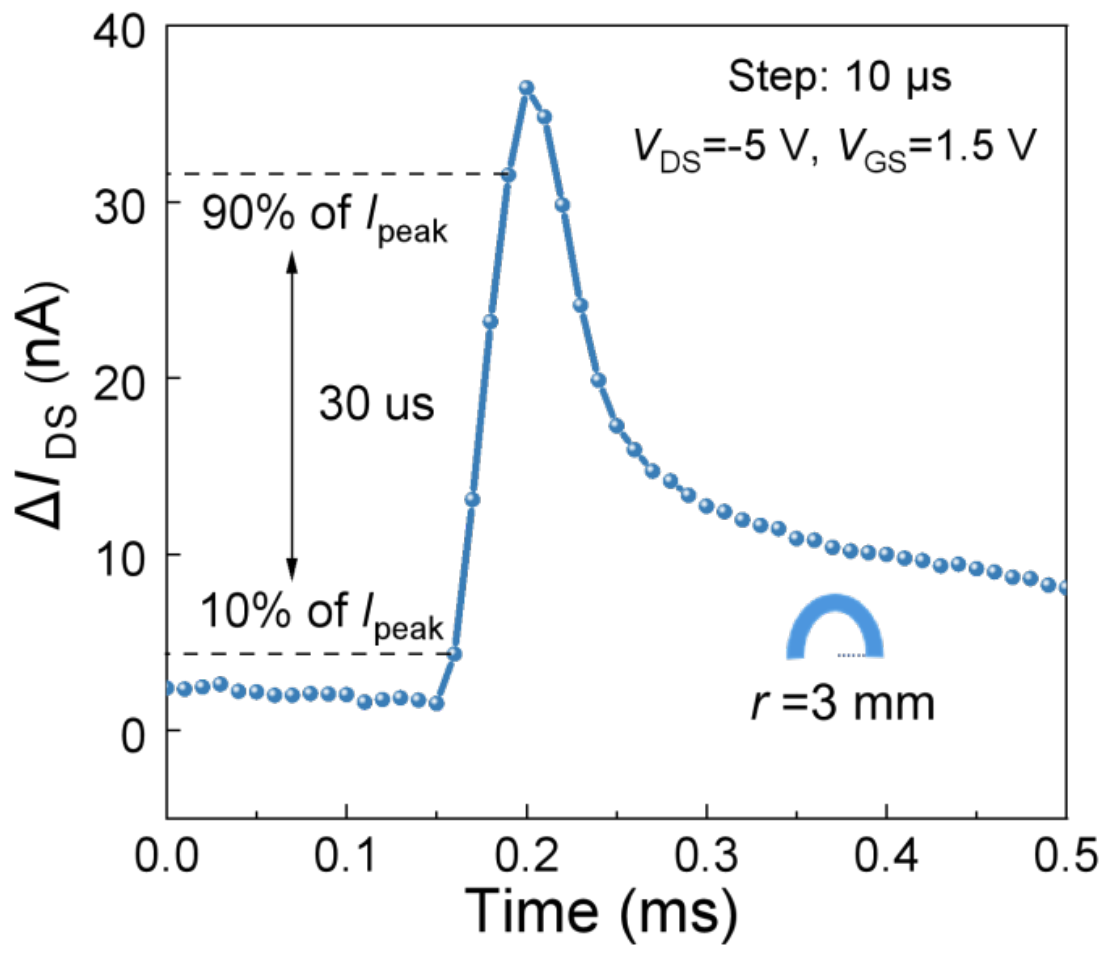

Fig. 2g | Photoresponse of the flexible device to a 50 μs laser pulse. The drain current is recorded during a 50 μs optical pulse with a readout interval of 10 μs. A clear current modulation is observed in response to the optical pulse, demonstrating the fast photoresponse capability of the device. The response time, defined by the standard 10%–90% rise time of the transient photocurrent, is approximately 30 μs.

Page 2: ... The mean mobility within the flexible device array is 22.65 (29.11) $cm^2\ V^{-1}\ s^{-1}$, demonstrating a rapid response time of 30 μs to 450 nm illumination.

Page 4: ... For sensing performance, the device exhibits outstanding detection sensitivity for monochromatic illumination at 450 nm and an optical response time (10%–90% rise time) of 30 μs. In memory applications, it demonstrates an electrical pulse response speed down to 130 ns, a stable multilevel programming speed of 10 μs, a memory window of 22.2 V, long-term retention stability over 10 years, and reliable cycling endurance exceeding 10^4 cycles.

Page 9: ... When subjected to laser pulses of 2 ms, 1 ms and 100 μs (Supplementary Fig. 17), the device exhibits consistent photoresponse down to 50 μs pulses (Figure 2g). The optical response time, defined by the standard 10%–90% rise time of the transient photocurrent, is approximately 30 μs. This experimental device achieves the highest response speed among comparable organic neuromorphic transistors (Fig. 2h[20, 30-33, 50, 56-65])

Page 20: ... The resulting flexible device integrates superior "sensing-memory-processing" capabilities: a fast optical response (30 μs),

Q5. Supplementary Table 2 shows that the selected 30 nm Al_2O_3/180 nm PAA stack has a memory window of 9.85 V, whereas the 30 nm PAA/30 nm Al_2O_3 stack reaches 12.8 V under the same

condition. Thus, the selected structure should be framed as a mobility–memory trade-off, not as the structure with superior charge-storage capability. In addition, the proposed mechanism—oxygen-vacancy trapping, interfacial charge accumulation and polar-PAA stabilization—is mainly inferred from polarity–memory correlations and EPR on single-layer Al_2O_3. More direct evidence, such as KPFM/Kelvin probe, temperature-dependent retention, C–V analysis or Fowler–Nordheim analysis of the Al_2O_3/PAA stack, is needed.

A5: Thank you for your comment. We acknowledge that the 30 nm PAA/30 nm Al_2O_3 stack exhibits a larger memory window (12.8 V) than our selected 30 nm Al_2O_3/180 nm PAA stack (9.85 V) under identical programming conditions. However, as we have consistently emphasized throughout our work, our primary objective was not to maximize the memory window alone, but rather to identify the dielectric configuration that offers the best comprehensive performance—specifically, the optimal trade-off between high carrier mobility (for fast signal response) and robust non-volatile charge trapping (for stable memory and neuromorphic functions). This balanced performance makes it the most suitable candidate for integrated "sensing-memory-processing" applications, where both fast response and reliable storage are required. We have now revised the manuscript to more clearly frame this as a mobility–memory trade-off optimization rather than a claim of superior charge storage alone, and we have added explicit discussion to this effect in the revised main text.

We also appreciate your suggestion to provide more direct evidence for the proposed charge trapping mechanism. In response, we have performed comprehensive EPR, XPS, and KPFM characterizations to corroborate our mechanistic model.

(1)**. Direct evidence of oxygen vacancies: EPR and XPS characterization.** To provide more direct evidence for the oxygen-vacancy trapping mechanism, we have performed EPR spectroscopy on three types of dielectric layers: single PAA, single Al_2O_3 (30 nm), and the Al_2O_3/PAA heterostructure (30 nm Al_2O_3/180 nm PAA). EPR is a powerful technique for detecting unpaired electrons associated with paramagnetic defects such as oxygen vacancies in oxide materials. As shown in **Supplementary Fig. 21**, the single PAA layer exhibits no discernible EPR signal at $g \approx 2.003$, confirming the absence of oxygen vacancies. In sharp contrast, both the single Al_2O_3 layer and the Al_2O_3/PAA heterostructure show a clear and distinct EPR signal at $g \approx 2.003$, which is characteristic of unpaired electrons trapped at oxygen vacancy sites in aluminum oxide. The signal intensity of the heterostructure is comparable to that of single Al_2O_3, indicating that the introduction of Al_2O_3 via LT-PEALD successfully incorporates oxygen vacancies into the dielectric

stack, providing abundant deep trapping sites for charge storage.

We have also performed high-resolution X-ray photoelectron spectroscopy (XPS) to further confirm the chemical states and oxygen vacancy content in the different dielectric layers. As shown in **Supplementary Figs. 22–24**, we analyzed the Al 2p, C 1s, N 1s, and O 1s core-level spectra for single PAA, single Al_2O_3, and the Al_2O_3-PAA heterostructure. Importantly, in the O 1s spectra, both the single Al_2O_3 and the heterostructure exhibit a distinguishable shoulder peak at a binding energy of approximately 531.5–532.0 eV, which is commonly assigned to oxygen vacancies (or defect-related oxygen species) in Al_2O_3 (**Supplementary Figs. 22 and 23**). This peak is absent in the single PAA layer (**Supplementary Figs. 24**). We note that because the Al_2O_3 layer is deposited on top of the PAA in our heterostructure, the XPS probing depth (typically 3–10 nm) primarily samples the Al_2O_3 surface; the N 1s signal from the underlying PAA is therefore relatively weak but does not compromise the identification of oxygen vacancies from the O 1s and Al 2p spectra. These XPS results are fully consistent with the EPR findings and collectively confirm the presence of oxygen vacancies in the Al_2O_3 dielectric stacks.

(2) **Direct evidence of charge trapping: KPFM measurements:** Beyond the structural evidence from EPR and XPS, we have performed KPFM to directly monitor the surface potential (work function) changes of the semiconductor layer under different programming conditions, which reflect the extent of charge trapping and the corresponding Fermi level shift. The KPFM measurements were carried out using a conductive tip with a work function of ϕtip = 5.1 eV (calibrated against HOPG). The contact potential difference VCPD is given by:

$$V_{CPD}=\frac{\phi_{sample}-\phi_{tip}}{e} \quad (6)$$

Where ϕ_{sample} is the work function of the semiconductor surface. The Fermi level of the sample is $E_{F,sample}$ = -ϕ_{sample}. Hence:

$$E_{F,sample}=-5.1eV-eV_{CPD} \quad (7)$$

For a p-type semiconductor (C10-DNTT), the hole concentration is

$$p=N_v exp(\frac{E_V-E_F}{k_B T}) \quad (8)$$

A decrease in E_F corresponds to a reduction in hole concentration, because the Fermi level moves away from the valence band edge. In our measurement, the observed V_{CPD} increase after negative gate pulsing reflects a decrease in hole density: after the negative gate pulse, holes are injected into the dielectric and trapped, leaving the semiconductor surface depleted of holes. This depletion

causes surface band bending, effectively raising the local vacuum level and increasing the measured V_{CPD}. The KPFM results are summarized as follows: For single PAA devices (**Supplementary Fig. 33**): Before programming, the surface potential is 36.1 (±4.1) mV. After applying a –20 V, 1 s gate pulse, the surface potential becomes 36.7 (±5.8) mV. The negligible change confirms that PAA alone has very weak charge trapping capability, consistent with the absence of oxygen vacancies. For single Al_2O_3 devices (**Supplementary Figs. 31 and 32**): Before programming, the surface potential is 22.7 (±2.8) mV. After applying gate pulses of –10 V, –12 V, –15 V, and –20 V for 1 s each, the surface potentials increase stepwise to 77.0 (±5.7) mV, 398.3 (±4.9) mV, 1.22 (±0.01) V, and 1.70 (±0.03) V, respectively. This monotonic and substantial increase demonstrates strong and tunable charge trapping in Al_2O_3., directly correlated with the oxygen vacancies confirmed by EPR and XPS. For the Al_2O_3/PAA heterostructure (**Supplementary Figs. 34 and 35**): A similar stepwise increase in surface potential was observed (from 26.0 mV up to 1.67 V under –20 V programming), confirming that the heterostructure retains the excellent trapping capability of Al_2O_3.

Taken together, the EPR and XPS data provide clear structural evidence for the presence of oxygen vacancies in the Al_2O_3 layer, which act as deep-level traps. The KPFM data, in turn, directly demonstrate that these traps are capable of trapping charge under a negative gate bias. We have also added relevant statements on page 10 and page 11 of the revised manuscript.

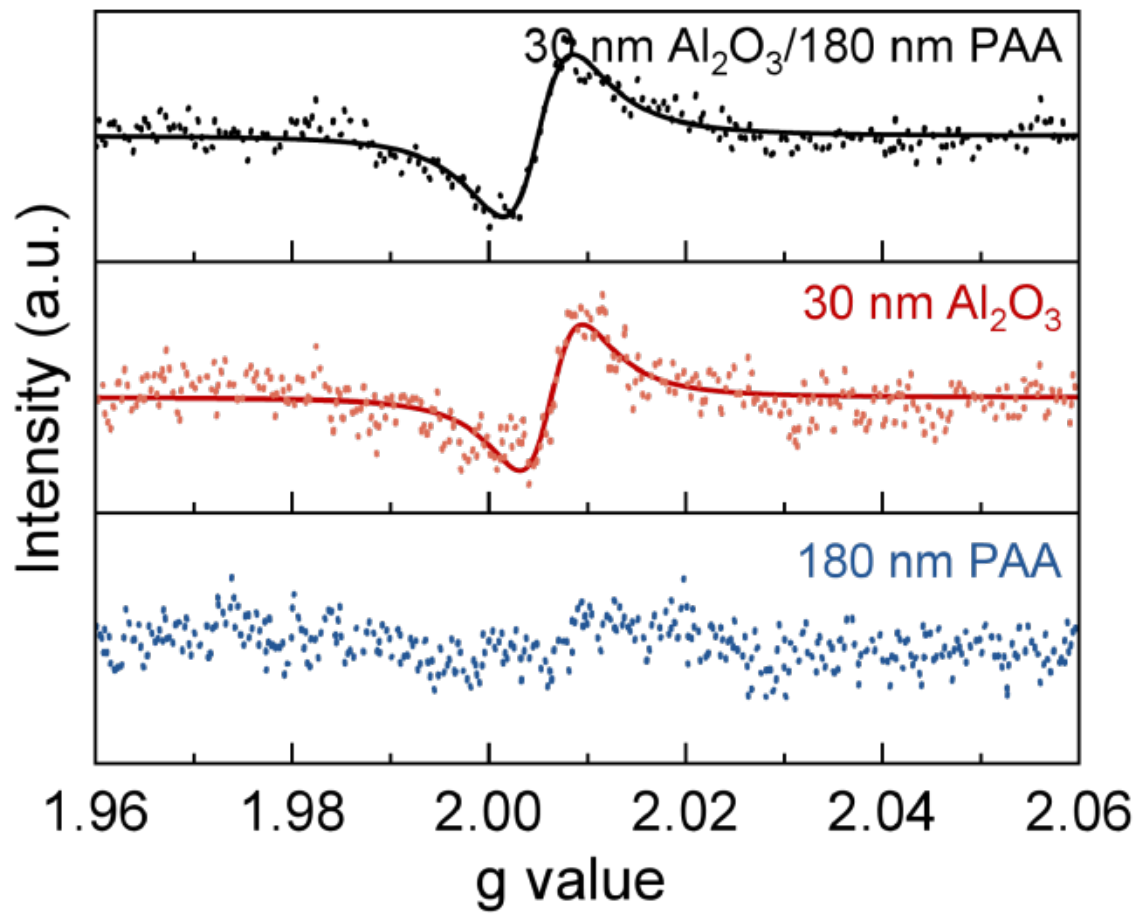

Supplementary Fig. 21 | Electron paramagnetic resonance (EPR) spectra of single PAA, single Al_2O_3 and Al_2O_3/PAA heterostructure. A clear signal at g≈2.003 is observed only for Al_2O_3-containing layers, confirming the presence of oxygen vacancies introduced by LT-PEALD Al_2O_3.

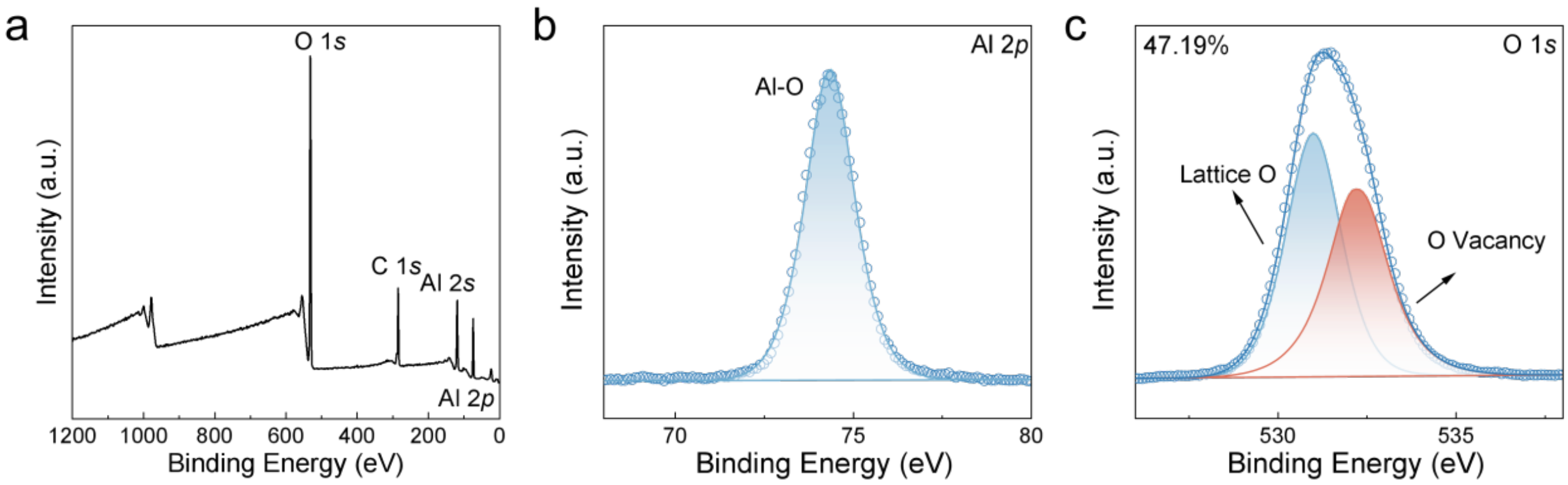

Supplementary Fig. 22 | XPS analysis of the single Al_2O_3 layer. a, Survey XPS spectrum of the Al_2O_3 layer, confirming the presence of Al, O and C elements. **b**, High-resolution Al 2p core-level spectrum, showing a single peak at 74.3 eV characteristic of Al–O bonding in Al_2O_3. **c**, High-resolution O 1s core-level spectrum with peak deconvolution. The main peak at 530.9 eV (lattice oxygen in Al–O), and a shoulder peak at approximately 531.8 eV (oxygen vacancies or defect-related oxygen species). The distinct shoulder peak at ~531.8 eV confirms the presence of oxygen vacancies in the Al_2O_3 layer, providing deep trapping sites for charge storage.

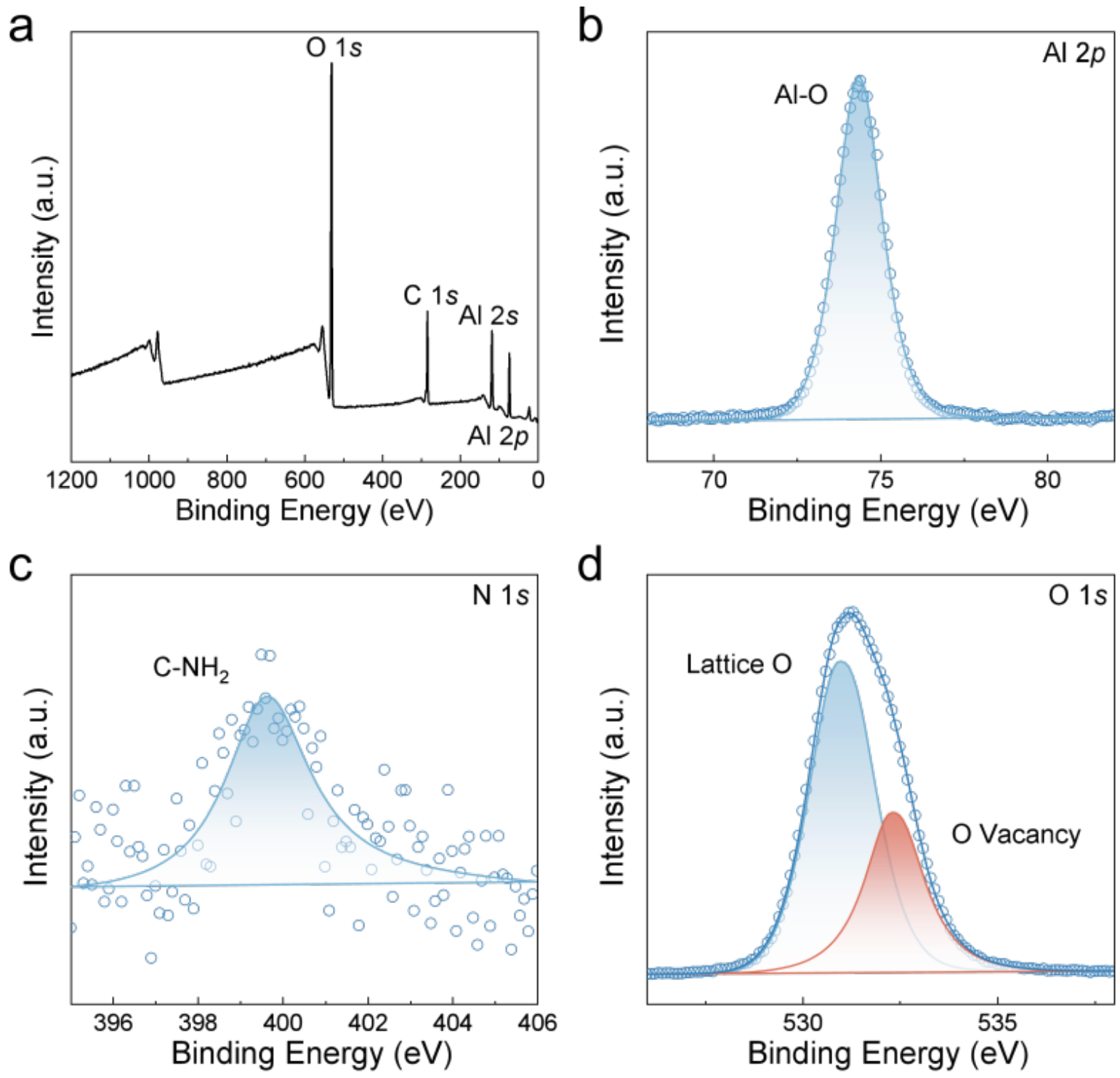

Supplementary Fig. 23 | XPS analysis of the single Al_2O_3/PAA layer. a, Survey XPS spectrum of the Al_2O_3 layer, confirming the presence of Al, O and C elements. **b**, High-resolution Al 2p core-level spectrum, showing a single peak at 74.3 eV characteristic of Al–O bonding in Al_2O_3. **c**, High-resolution N 1s core-level spectrum. A weak signal is observed due to the limited probing depth of XPS (typically 3-10 nm), which primarily samples the top Al_2O_3 layer rather than the underlying PAA. **d**, High-resolution O 1s core-level

spectrum with peak deconvolution. The main peak at 530.9 eV (lattice oxygen in Al–O), and a shoulder peak at approximately 531.8 eV (oxygen vacancies or defect-related oxygen species).

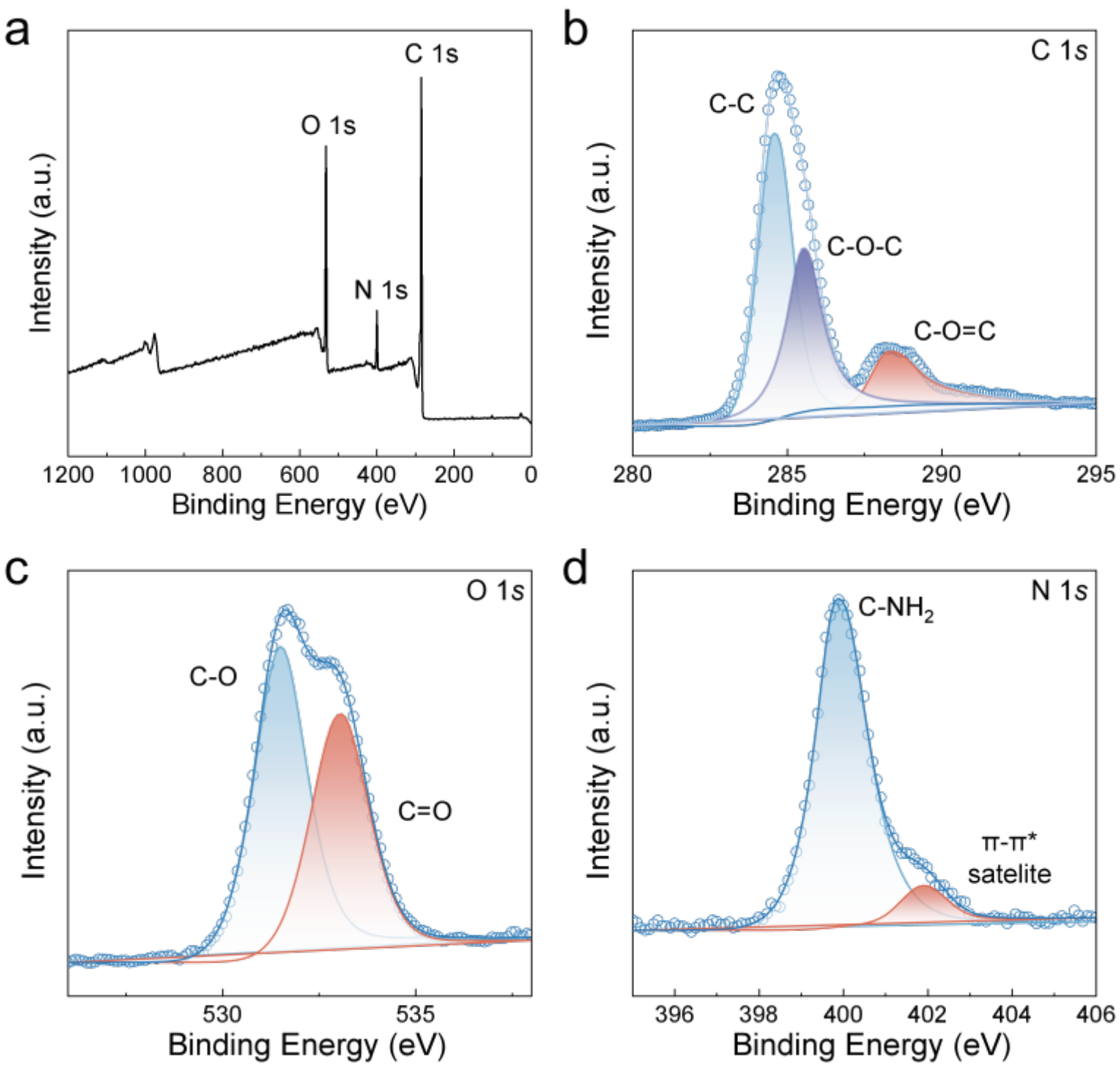

Supplementary Fig. 24 | XPS analysis of the single PAA layer. a, Survey XPS spectrum of the PAA layer, confirming the presence of C, O and N elements. **b**, High-resolution C 1s core-level spectrum with peak deconvolution. The C 1s spectrum can be fitted to three components at 284.8 eV (C–C/C–H), 286.0 eV (C–O), and 288.6 eV (O–C=O), characteristic of the poly(amic acid) structure. **c**, High-resolution O 1s core-level spectrum, fitted to two components at 531.5 eV (C–O) and 533.0 eV (C=O), consistent with the carboxyl and carbonyl groups in PAA. **d**, High-resolution N 1s core-level spectrum, showing a peak at approximately 400.1 eV corresponding to the amide groups (–NH–) in PAA. No signal at ~531.8 eV (oxygen vacancies) is observed in the O 1s spectrum, consistent with the EPR result confirming the absence of oxygen vacancies in the single PAA layer.

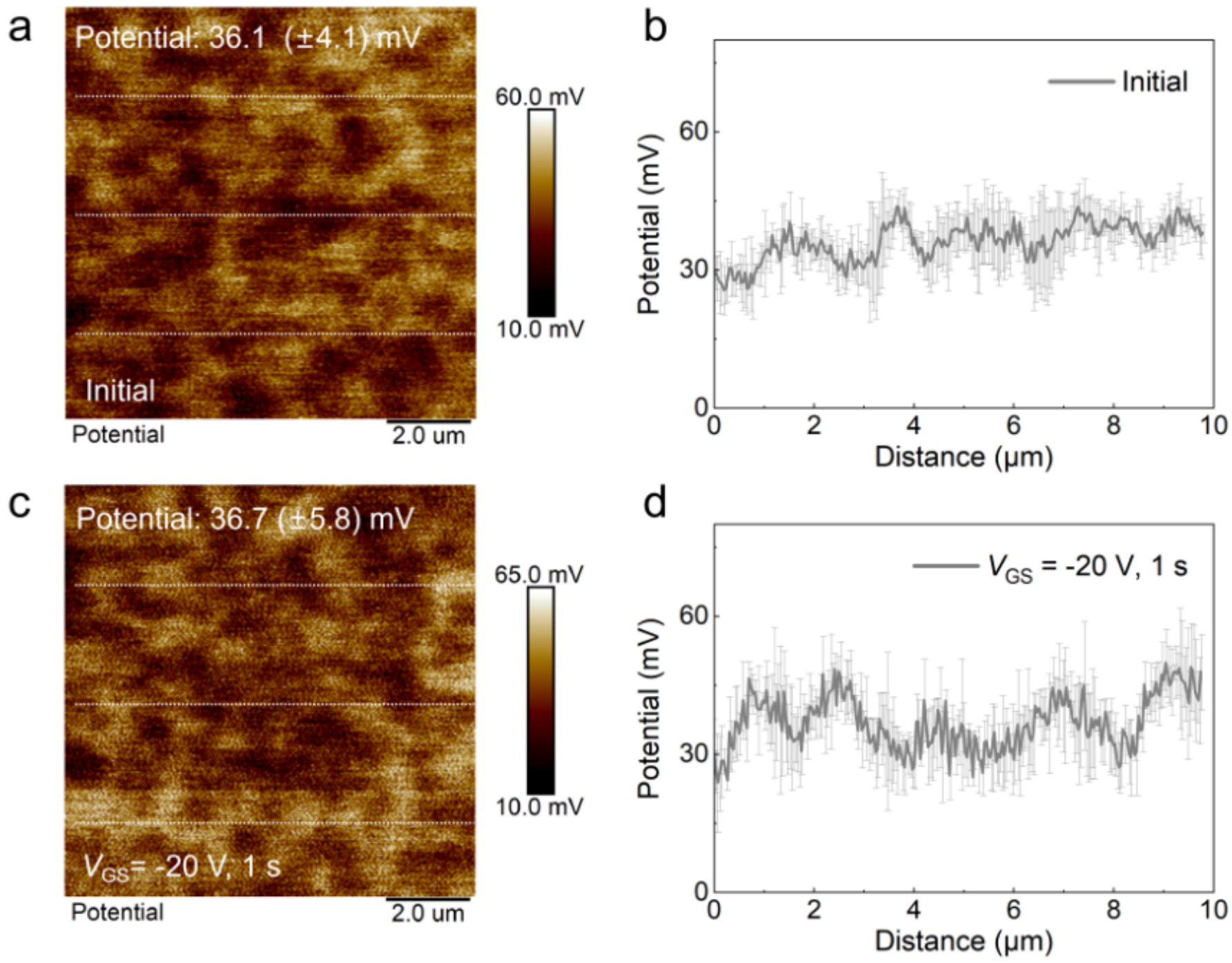

Supplementary Fig. 33 | Surface potential characteristics of transistors based on a 180 nm PAA dielectric layer before and after gate voltage pulsing. **a**, KPFM surface potential mapping of the semiconductor channel before gate voltage application. **b**, KPFM surface potential mapping after applying a –20 V, 1 s gate pulse. **c**, Statistical distribution of the initial surface potential (mean value: 36.1 ± 4.1 mV). **d**, Statistical distribution of the surface potential after pulsing (mean value: 36.7 ± 5.8 mV). The negligible change confirms that the single PAA layer exhibits very weak charge trapping capability.

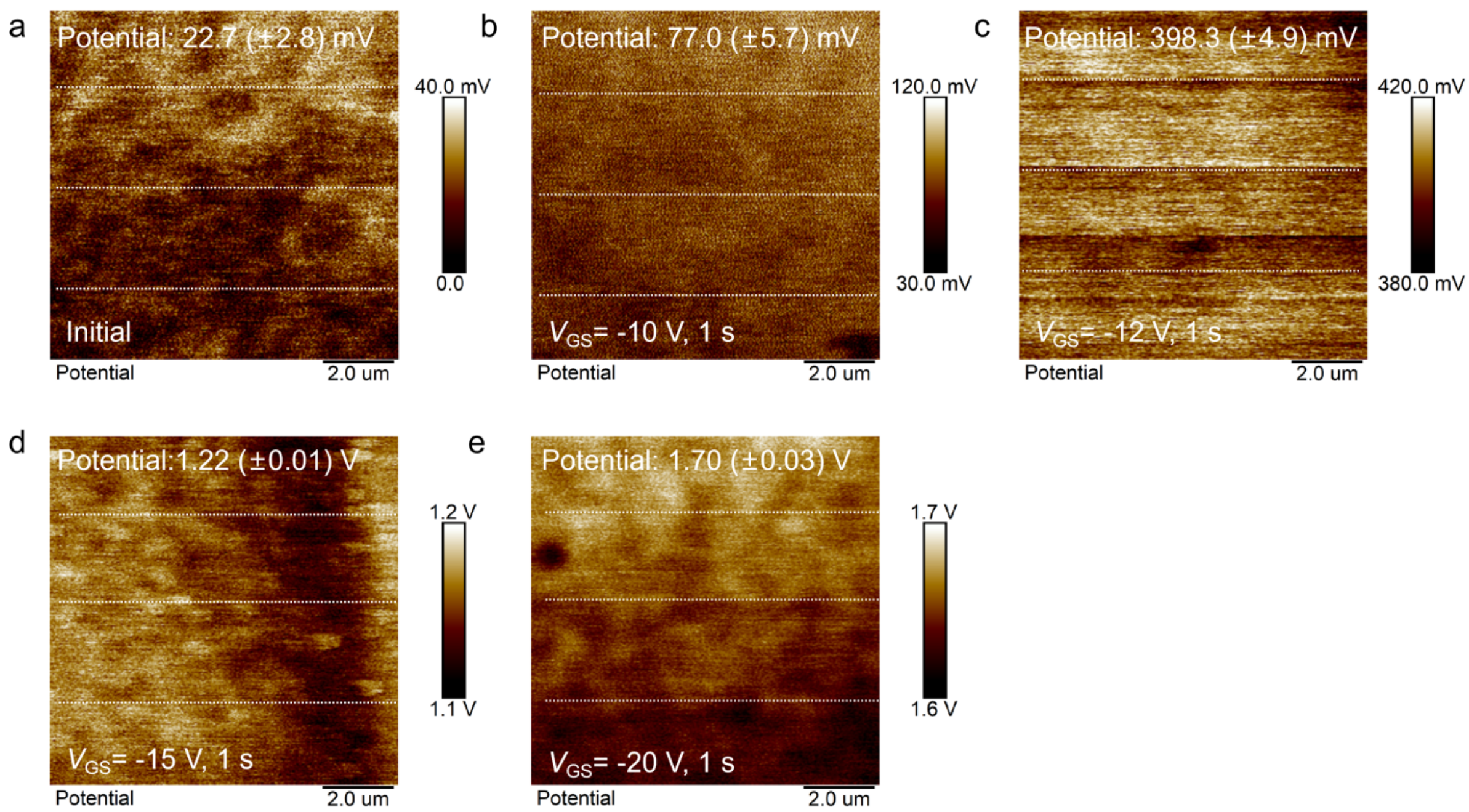

Supplementary Fig. 31 | Surface potential mapping of the transistor channel for a device with a 30

nm Al_2O_3 dielectric layer under different gate pulse conditions. a, KPFM surface potential image of the semiconductor channel in the pristine state (without gate voltage application). **b–e**, KPFM surface potential images after applying gate pulses of –10 V (**b**), –12 V (**c**), –15 V (**d**), and –20 V (**e**) for 1 s each. The pulse was removed prior to KPFM scanning.

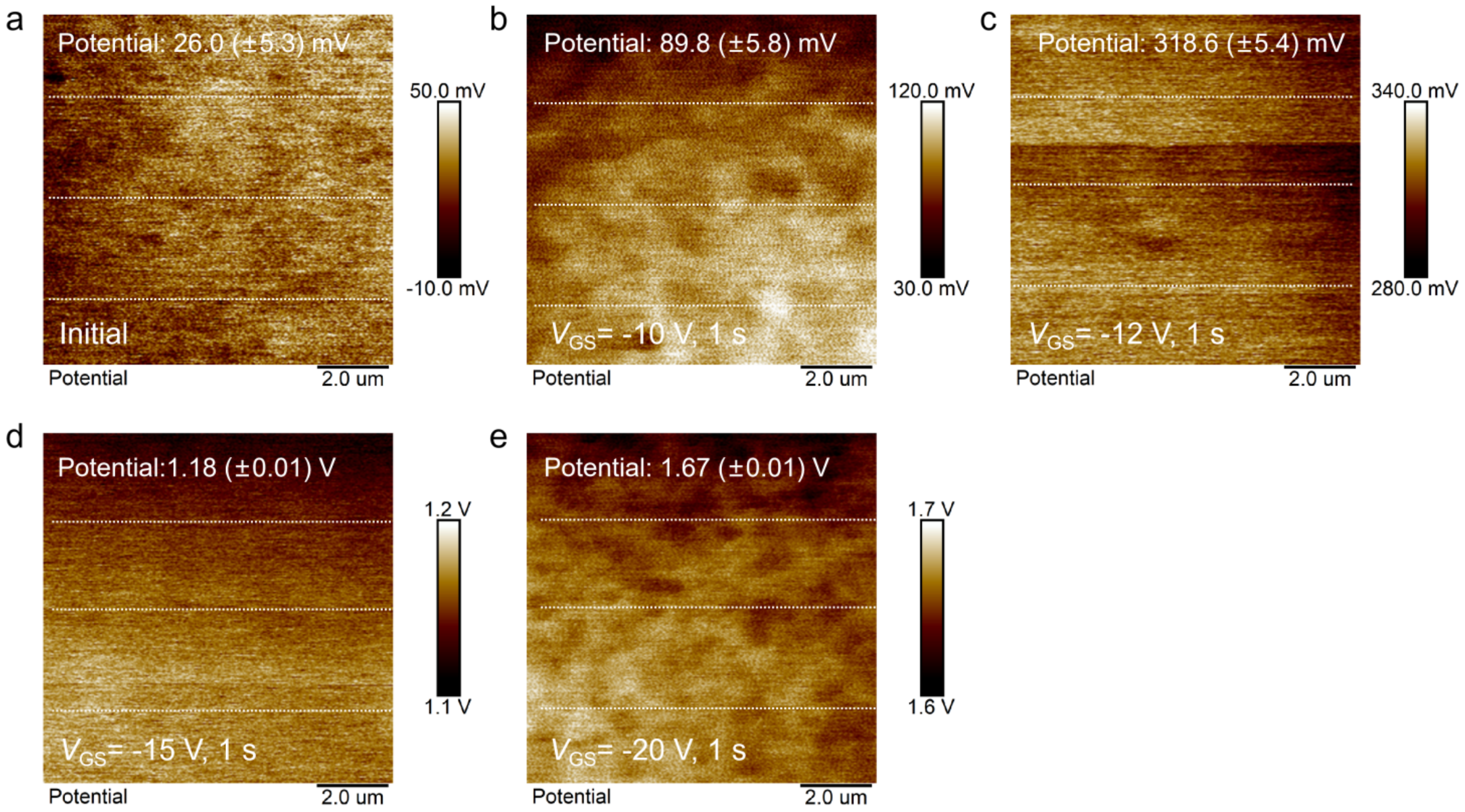

Supplementary Fig. 34 | Surface potential mapping of the transistor channel for a device with a 30 nm Al_2O_3/180 nm PAA dielectric layer under different gate pulse conditions. a, KPFM surface potential image of the semiconductor channel in the pristine state (without gate voltage application). **b–e**, KPFM surface potential images after applying gate pulses of –10 V (**b**), –12 V (**c**), –15 V (**d**), and –20 V (**e**) for 1 s each. The pulse was removed prior to KPFM scanning.

Page 10: ...EPR signals[70] (g = 2.003; Supplementary Fig. 21) confirm oxygen vacancies in Al_2O_3 as deep traps, which are present in both the single Al_2O_3 layer and the Al_2O_3/PAA heterostructure but absent in the single PAA layer (Supplementary Figs. 22–24). This assignment is further corroborated by XPS O 1s spectra, where a distinct shoulder peak at ~531.8 eV, characteristic of defect-related oxygen species, is observed exclusively in Al_2O_3-containing dielectrics (Supplementary Fig. 25)...

Page 11: ...To directly probe the trap-induced energy level shift and the charge trapping role of oxygen vacancies, we performed Kelvin probe force microscopy (KPFM) on devices with different dielectric configurations. For single Al_2O_3 devices (Supplementary Figs. 31 and 32), the surface potential increases stepwise from 22.7 mV to 1.70 V as the programming voltage increases from –

10 V to –20 V, confirming strong charge trapping; in contrast, single PAA devices (Supplementary Figs. 33) show negligible change (from 36.1 mV to 36.7 mV) under the same conditions. The Al_2O_3/PAA heterostructure (Supplementary Figs. 34 and 35) exhibits a similar stepwise increase (from 26.0 mV to 1.67 V), confirming that it inherits the strong trapping capability of Al_2O_3. These KPFM results provide direct evidence of trap-induced Fermi-level shifts and corroborate the charge storage mechanism inferred from the electrical measurements.....

Q6. The headline mobility values are difficult to assess because Supplementary Table 1 reports mobility at 20 Hz, Supplementary Fig. 7b reports 130-device statistics at 0.1 Hz, and Supplementary Fig. 2 shows strong frequency dependence of Ci. The authors should specify which Ci value was used for each mobility extraction, recalculate mobility using frequency-appropriate capacitance, and provide TLM or four-probe measurements to rule out contact-resistance artefacts. In addition, the basis of $D^* > 10^{14}$ Jones should be stated explicitly. If it is derived from dark-current or shot-noise assumptions rather than direct NEP/noise measurements, it should be reported as extrapolated detectivity, since such estimates can overstate actual faint-light detection performance.

A6: Thank you for your detailed comments. We agree that the capacitance of the dielectric layer is frequency-dependent, and that the extracted mobility values must correspond to the specific frequency used when measuring Ci; furthermore, our discussion of the capacitance frequency in the original manuscript was indeed somewhat disorganized.

(1). Clarification on capacitance values: for all dielectric configuration comparisons—including variations in PAA thickness, Al_2O_3 thickness, deposition sequence (PAA/Al_2O_3 vs. Al_2O_3/PAA), and different polar polymers (PS, PAN, PVP, PPO)—the capacitance values used for mobility extraction were all measured at 20 Hz. This frequency was chosen because it is the most reported condition in the literature for organic transistor mobility evaluation, allowing fair and direct comparison with previously published results. For the optimized heterostructure (30 nm Al_2O_3/180 nm PAA), we additionally characterized the capacitance at 0.1 Hz to verify the device stability under lower-frequency operation, which is relevant for long-term memory applications. The corresponding mobility extracted using the 0.1 Hz capacitance is reported in **Supplementary Fig. 10b** for the 130-device statistics. We have now revised the manuscript to clearly specify the capacitance

frequency alongside every reported mobility value (in the main text, figure captions, and Supplementary Tables), ensuring full transparency and ease of assessment. If the reviewer deems the 0.1 Hz data unnecessary, we are happy to retain only the 20 Hz dataset; however, we believe that including both frequencies demonstrates the robustness of our device performance across different operating conditions.

(2). TLM measurements to exclude contact-resistance artefacts. To rigorously rule out contact-resistance artefacts and confirm that the extracted mobility values reflect intrinsic channel transport rather than contact-limited injection, we performed TLM measurements on devices based on the optimized 30 nm Al_2O_3 / 180 nm PAA dielectric stack. TLM is a standard and widely accepted technique for extracting contact resistance in field-effect transistors. The measurement principle is as follows: we fabricated multiple devices with identical widths (W=1 mm) but varying channel lengths (L=30, 40, 50, 60, 70, 80, 90, 110, 130 μm) (**Supplementary Fig. 7**). The total resistance (R_{ON}) between source and drain in the linear regime is given by:

$$R_{ON}=\frac{V_{DS}}{I_{DS}}=R_{\mathrm{ch}}+R_c \qquad (9)$$

where R_{ch} is the channel resistance and R_c is the total contact resistance (including both source and drain contacts). The channel resistance can be expressed as:

$$R_{\mathrm{ch}}=\frac{L}{W\cdot C_i\cdot \mu\cdot (V_{GS}-V_{TH})} \qquad (10)$$

Thus, by plotting R_{ON} as a function of channel length L at a fixed gate overdrive (V_{GS}-V_{TH}) , the data points should fall on a straight line. The slope of this line yields the channel sheet resistance, while the intercept at L=0, gives the total contact resistance R_c. As shown in **Supplementary Figs. 8 and 9**, the TLM plot exhibits excellent linearity, confirming ohmic contact behaviour. From the y-axis intercept, we extracted a channel-width-normalized contact resistance of: R_cW= 420 Ω·cm. This value is within the range of reported values for high-mobility organic transistors (10–10^3 Ω·cm)[4,5,6] and is sufficiently low to ensure that the device performance is not contact-limited. This confirms that the high mobility values we report genuinely reflect the intrinsic charge transport properties of the C10-DNTT semiconductor on our optimized dielectric interface, rather than artefacts arising from contact resistance.

(3). Specific detectivity based on directly measured noise current: We thank the reviewer for pointing out the importance of rigorous noise characterization. In our original manuscript, the detectivity values were indeed based on dark-current or shot-noise assumptions, which, as the

reviewer correctly notes, can overestimate the actual faint-light detection performance. To address this, we have now directly measured the noise current (i_n) under dark conditions. The measurement was performed by continuously sampling the drain current in the dark at a fixed bias (V_{DS}= -5 V, V_{GS}= 0 V) over an extended period, and the root-mean-square (RMS) value of the current fluctuation was taken as i_n. The measured dark-state noise current of our device is:

$$i_n=2.05\times10^{-13}A$$

Using this directly measured noise current, we calculated the noise-equivalent power (NEP) and specific detectivity (D^*) with the standard formulae:

$$NEP=\frac{i_n}{R} \quad (11)$$

$$D^*=\frac{\sqrt{A}}{NEP}=\frac{R\cdot\sqrt{A}}{i_n} \quad (12)$$

where R is the responsivity ($A\cdot W^{-1}$) and A is the effective channel area (cm^2). This direct noise measurement provides a more reliable and conservative estimate of detectivity than assumptions based on shot noise. Using this method, we recalculated the specific detectivity for all measured wavelengths. We have also added relevant statements on page 7 and page 9 of the revised manuscript.

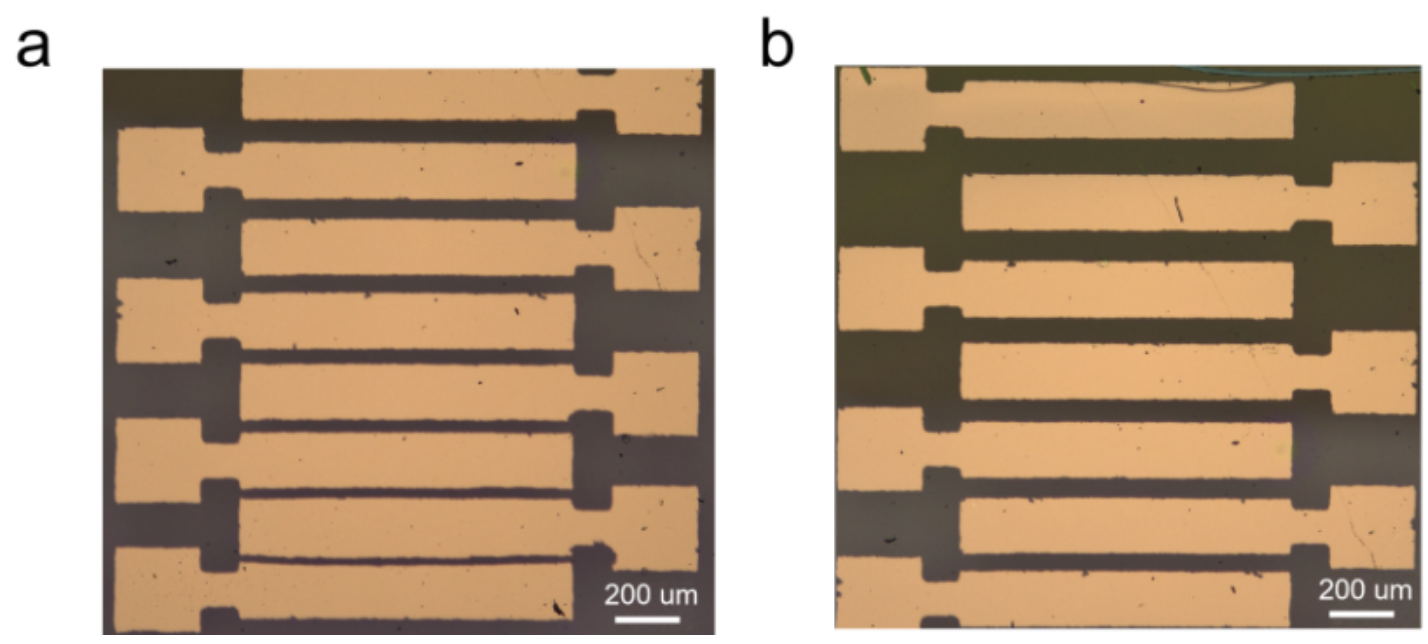

Supplementary Fig. 7 | Optical microscopy image of the TLM electrode array. Channel widths are fixed at W=1 mm, with channel lengths varying from L=30 μm to 130 μm (30, 40, 50, 60, 70, 80, 90, 110 and 130 μm) for contact resistance extraction.

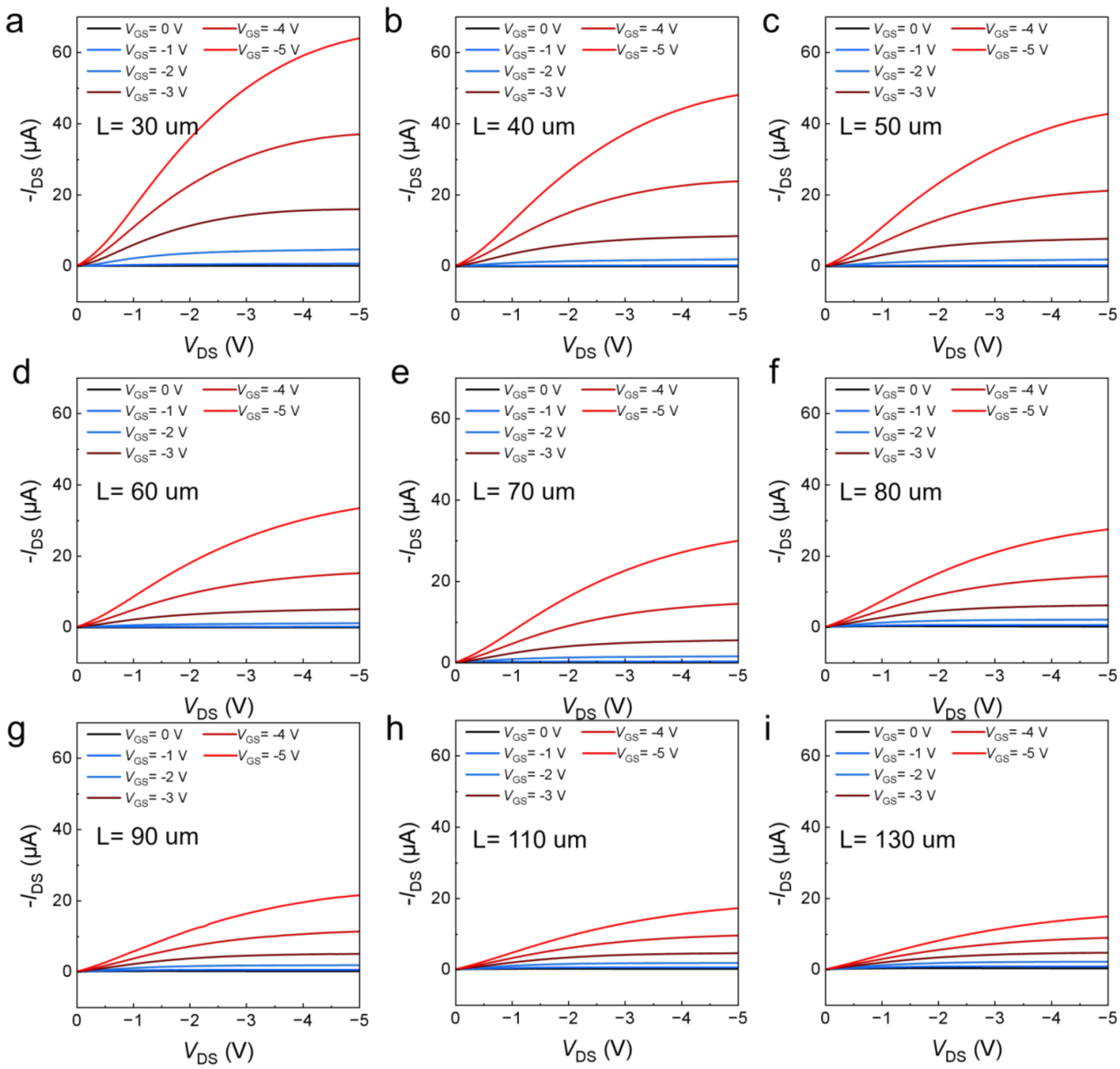

Supplementary Fig. 8 | Output characteristics of TLM devices with varying channel lengths (L=30–130 μm, W=1 mm). a-i, I_{DS}-V_{DS} curves measured at different V_{GS} for devices with channel lengths of 30 μm (**a**), 40 μm (**b**), 50 μm (**c**), 60 μm (**d**), 70 μm (**e**), 80 μm (**f**), 90 μm (**g**), 110 μm (**h**), and 130 μm (**i**). The linear behaviour in the low-bias regime confirms ohmic contacts, validating the subsequent TLM extraction of contact resistance.

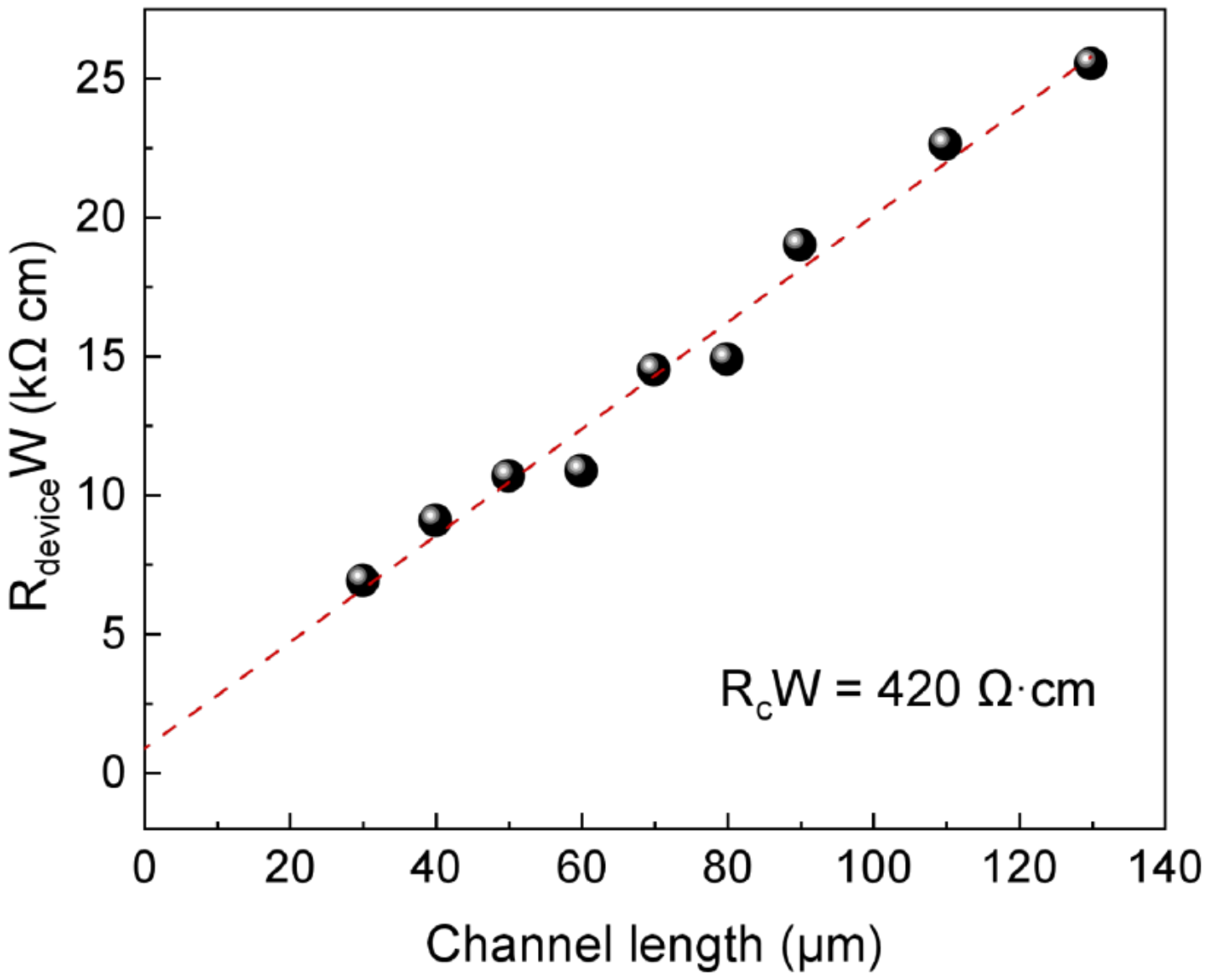

Supplementary Fig. 9 | Total on-resistance R_{ON} as a function of channel length L extracted from TLM measurements. The linear fit of R_{ON} versus L yields a channel-width-normalized contact resistance of R_{ON} = 420 Ω·cm, confirming ohmic contacts and validating the extracted mobility values.

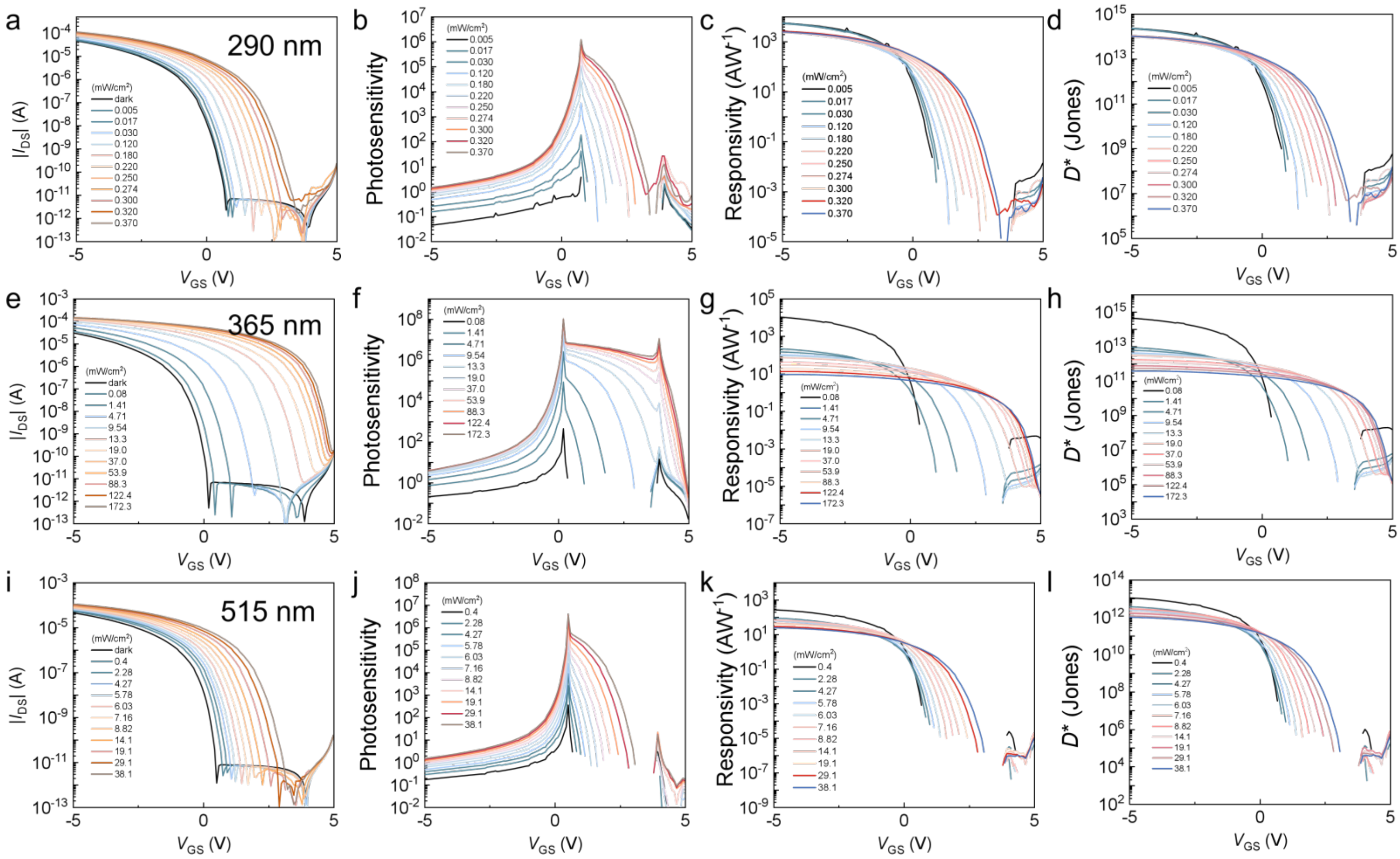

Supplementary Fig. 14 | Multi-wavelength photoresponse characteristics of the 30 nm Al_2O_3/180 nm PAA device. Photoresponse performance was systematically characterized under monochromatic illumination at 290 nm, 365 nm, and 515 nm. **a,e,i,** Transfer curves measured in the dark and under

illumination at 290 nm (**a**), 365 nm (**e**), and 515 nm (**i**). **b,f,j,** Corresponding *P* values extracted from the transfer curves. **c,g,k**, *R* values. **d,h,l,** Noise-corrected *D** values derived from the directly measured dark-state noise current (i_n= 2.05×10^{-13} A) The device exhibits clear photoresponse across all tested wavelengths.

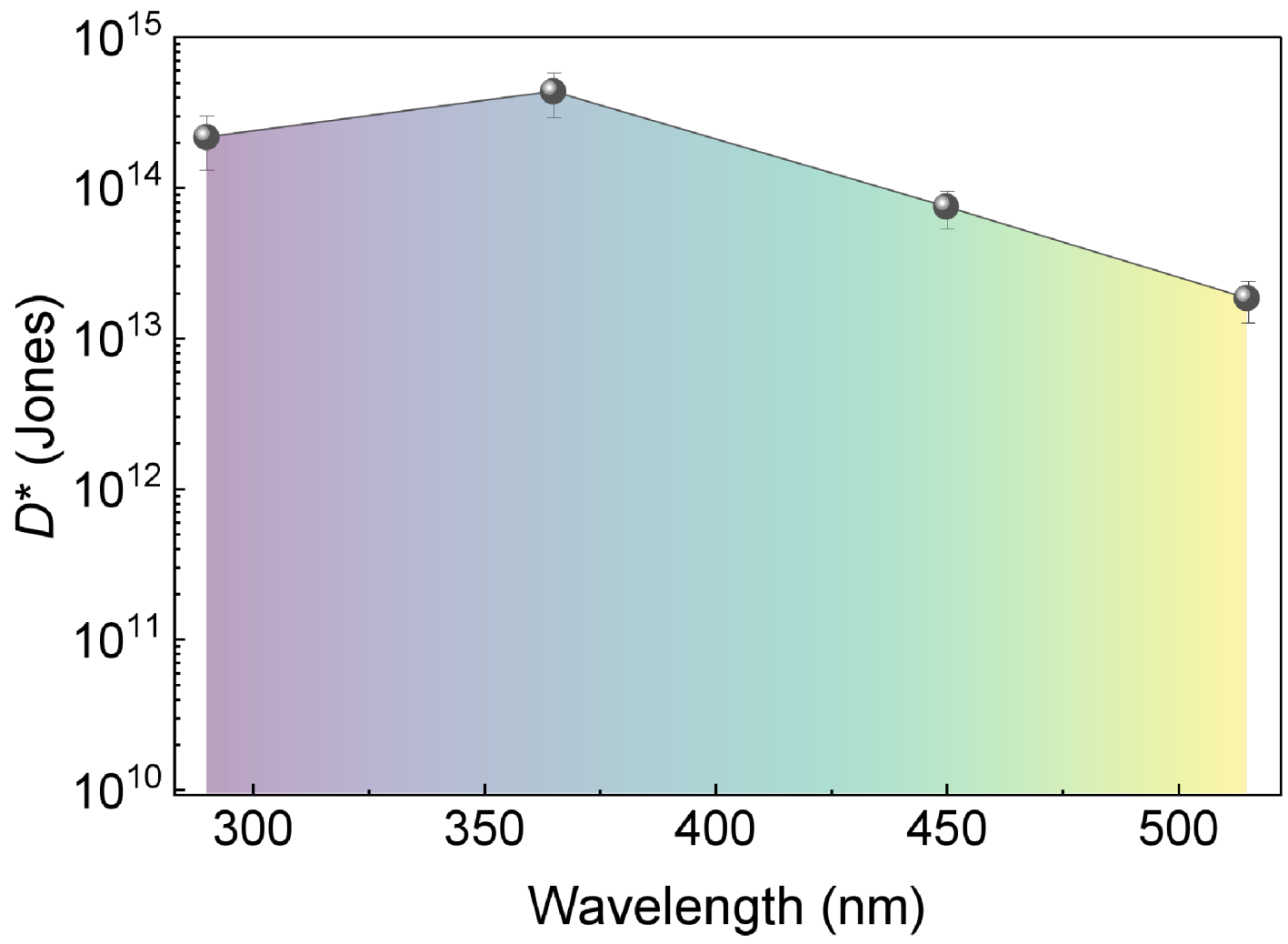

Supplementary Fig. 15 | Multi-device statistical summary of *D** at different wavelengths for the 30 nm Al_2O_3 / 180 nm PAA device.

Page 8: d, Mobility distribution map of 130 devices on a flexible substrate (*f*=0.1 Hz). e, Comparison of the mobility of the devices on flexible substrate bending for 1000 cycles at different bending radius (*f*=0.1 Hz).

Page S7: Supplementary Fig. 6 | Statistical summary of OTFT mobility for each dielectric configuration in Supplementary Fig. 4, with capacitance measured at *f*= 20 Hz....

Page S44: Supplementary Fig. 43 | Statistical comparison of field-effect mobility in Al_2O_3/polymer heterostructure dielectric-based OTFTs with C10-DNTT semiconductor, across different polymer configurations (*f* = 20 Hz).

Page S55: Supplementary Fig. 54 | Statistical comparison of field-effect mobility in Al_2O_3/polymer heterostructure dielectric-based OTFTs with DNTT semiconductor, across different polymer configurations (*f* = 20 Hz).

Page 7: ...To exclude contact-resistance artefacts, we performed transmission line method (TLM) measurements on devices with the optimized dielectric stack (Supplementary Figs. 7–9). The extracted channel-width-normalised contact resistance is R_cW=420 Ω·cm, which is within the typical range (10–10^3 Ω·cm) reported for high-mobility transistors[50-52]...

Page 9: ...The primary photoresponsive parameters of the device, derived from the photoresponse transfer curves, include a photosensitivity (*P*) value exceeding 10^7, a peak responsivity (*R*) value of 2.01×10^3 A W^{-1}, and a maximum specific detectivity (*D**) value approaching 10^{14} Jones, as illustrated in Supplementary Fig. 12b-e. This value is obtained from the directly measured noise current after noise correction (Supplementary Fig. 13). To further validate the multi-wavelength capability, extended the spectral response to 290 nm, 365 nm and 515 nm (Supplementary Figs. 14 and 15). The noise-corrected *D** values at these wavelengths reach up to 4.4×10^{14} Jones (365 nm), confirming that our device maintains robust photodetection across a multi-wavelength spectral range while preserving the outstanding performance at 450 nm.

References:

1. Lee, S. et al. Organic flash memory on various flexible substrates for foldable and disposable electronics. *Nat. Commun.* **8**, 725 (2017).
2. Li, Y. et al. Low-voltage ultrafast nonvolatile memory via direct charge injection through a threshold resistive-switching layer. *Nat. Commun.* **13**, 4591 (2022).
3. Wang, X. et al. Van der Waals engineering of ferroelectric heterostructures for long-retention memory. *Nat. Commun.* **12**, 2821 (2021).
4. Borchert, J. W. et al. A simple and robust approach to reducing contact resistance in organic transistors. *Nat. Commun.* **9**, 5130 (2018).
5. Paterson, A. F. et al. Addition of the Lewis Acid $Zn(C_6F_5)_2$ Enables Organic Transistors with a Maximum Hole Mobility in Excess of 20 cm^2 V^{-1} s^{-1}. *Adv. Mater.* **31**, 1900871 (2019).
6. He, D. et al. Ultrahigh mobility and efficient charge injection in monolayer organic thin-film transistors on boron nitride. *Sci. Adv.* **3**, e1701186 (2017).
